# Photocatalyzed Ring Expansion of α‐Ketosulfonylaziridines: Ready Access to δ‐Sultams

**DOI:** 10.1002/anie.202516731

**Published:** 2025-12-08

**Authors:** Marco M. Mastandrea, Stefania Perulli, Vittoria Martini, Ludovica Primitivo, Lei‐Jie Zhou, Christian Mück‐Lichtenfeld, Miquel A. Pericàs, Olga García Mancheño

**Affiliations:** ^1^ Organic Chemistry Institute University of Münster Corrensstraße 36/40 48149 Münster Germany; ^2^ Leibniz Institute for Catalysis Albert‐Einstein‐Straße 29A 18059 Rostock Germany; ^3^ Departament de Química Física i Inorgànica Universitat Rovira i Virgili Campus Sescelades, Marcel.lí Domingo s/n Tarragona 43007 Spain; ^4^ Royal Academy of Sciences and Arts of Barcelona Chemistry Section, La Rambla 115 Barcelona 08002 Spain; ^5^ Institute of Chemical Research of Catalonia (ICIQ) Av. Països Catalans, 16 Tarragona 43007 Spain

**Keywords:** Photocatalysis, Radical‐polar crossover, Ring expansion, Sultams, α‐Ketoaziridines

## Abstract

Herein, a novel photocatalytic ring expansion reaction of aziridines to δ‐sultams through intramolecular radical cyclization is reported. In particular, versatile *N*‐sulfonyl‐protected α‐ketoaziridines were envisioned to undergo a ring opening–expansion through a photoredox‐mediated radical–polar crossover (RPC) process to provide six‐membered sultams bearing an unprecedented 2,3‐disubstitution pattern. The generality of the reaction was explored with a wide variety of substrates, showing a good functional group tolerance. Moreover, the robustness of the method allows a 10‐fold upscaling, providing similar yield or diastereomeric preference for the *trans*‐disubstituted products. Mechanistic investigations supported the postulated radical–polar crossover pathway, involving aziridine ring opening by photocatalytic single‐electron reduction via a carbonyl radical anion, followed by intramolecular cyclization with the aryl unit of the *N*‐sulfonyl group and re‐aromatization. Thus, this strategy opens new possibilities for the construction of highly decorated heterocycles by photoredox‐mediated RPC ring expansion reactions.

## Graphical abstract

A novel radical–polar crossover (RPC) photoredox‐catalyzed reaction for the synthesis of δ‐sultams from *N*‐sulfonyl α‐ketoaziridines through a controlled ring opening–expansion process is presented. This method allows the easy access to sultams with an unprecedented substitution pattern in up to 85% yield and 2:1 d.r., and highlights the potential of photocatalytic RPC strategies for the construction of value‐added heterocyclic molecules.

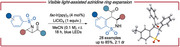

Visible‐light photoredox catalysis^[^ ^1^, ^2^, ^3^, ^4^, ^5^, ^6^ ^]^ has provided a way to harness the synthetic potential of radicals under mild conditions, while still retaining the benefits associated with odd‐electron reactivity. This characteristic of photoredox processes has promoted their rapid uptake in both academia and industry. Visible‐light‐mediated approaches have impacted an array of different reaction classes,^[^ ^1^, ^2^, ^3^, ^4^, ^5^, ^6^ ^]^ including radical‐polar crossover (RPC) processes that involve a transition from radical to polar (ionic) chemistry.^[^ ^7^, ^8^, ^9^ ^]^ Although the first RPC reaction was reported in 1993,^[^ ^10^ ^]^ this strategy has been only recently identified as a powerful synthetic tool in combination with visible‐light photoredox catalysis.^[^ ^11^, ^12^, ^13^ ^]^ In this case, a radical intermediate is initially generated through interaction of the substrate with a photocatalyst in its excited state, and, after being engaged in further transformations, undergoes an additional single electron (SET) reduction or oxidation to generate an ionic species. The fact that both radical and ionic intermediates participate in the RPC reaction pathway allows the rapid incorporation of diverse, reactive functional groups. Furthermore, photoredox‐mediated RPC processes offer new avenues for the synthesis of heterocyclic molecules.^[^ ^7^, ^8^, ^9^, ^14^, ^15^, ^16^ ^]^

In this context, the controlled ring expansion of aziridines remains an interesting strategy to access a broad variety of *N*‐heterocycles. Besides the intensively studied classical polar reactivity of aziridines,^[^ ^17^, ^18^, ^19^, ^20^ ^]^ SET^[^ ^21^ ^]^ and photocatalytic approaches^[^ ^22^, ^23^, ^24^, ^25^, ^26^, ^27^, ^28^, ^29^, ^30^ ^]^ for their ring expansion to form azetidines,^[^ ^26^ ^]^ or pyrrolidine and piperidine derivatives by dipolar cycloadditions^[^ ^27^, ^28^, ^29^, ^30^ ^]^ have been recently established. However, the development of mild and selective routes allowing ready access to important types of heterocycles from these precursors through new reactivities remains as an unmet, yet highly desirable need.

An interesting class of highly versatile building blocks for the synthesis of functionalized heterocycles through photocatalysis are the ring‐strained α‐ketoaziridines.^[^ ^31^, ^32^, ^33^ ^]^ In this regard, Louis Fensterbank, Cyril Ollivier, and co‐workers already showed the possibility of photocatalytic ring opening – ketyl radical‐anion formation – radical trapping of α‐ketoepoxides and α‐keto aziridines (Scheme 1a).^[^ ^34^ ^]^ However, to the best of our knowledge, no photocatalytic strategy for the ring expansion of such derivatives has been reported so far.

**Scheme 1:**
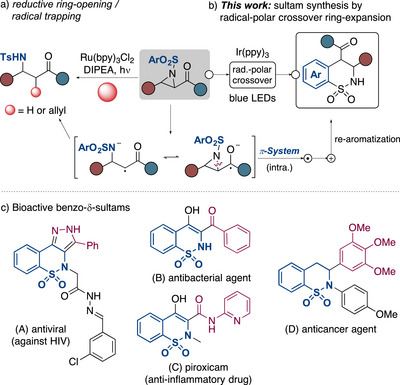
Photocatalytic reactions of α‐ketoaziridines: a) reductive ring‐opening/radical trapping, and b) *this work* on the RPC‐based ring expansion. c) Illustrative examples of biological relevant benzo‐δ‐sultams.

Encouraged by the rich opportunities offered by the operation of photoredox‐mediated RPC^[^ ^7^, ^8^, ^9^ ^]^ on α‐ketoaziridines,^[^ ^31^, ^32^, ^33^ ^]^ we envisaged the straightforward construction of sultams, an important class of cyclic sulfonamides presenting a broad spectrum of biological and therapeutical activities.^[^ ^35^, ^36^, ^37^, ^38^ ^]^ In particular, benzo‐δ‐sultams represent key target structures for drug discovery. They show promising antiviral (A),^[^ ^39^ ^]^ antimicrobial (B),^[^ ^40^ ^]^ anti‐inflammatory (C),^[^ ^41^ ^]^ or anticancer (D)^[^ ^42^ ^]^ activities, among others (Scheme 1c). Thus, oxicams, such as piroxicam (C),^[^ ^43^ ^]^ are currently the most prominent non‐steroidal anti‐inflammatory drugs on the market. Therefore, the development of new efficient routes that allow for different substitution patterns is still highly desirable.

We herein report the hitherto unknown RPC‐based ring expansion of *N*‐arylsulfonyl α‐ketoaziridines (Scheme 1b). The presence of the α‐carbonyl group allows a controlled photoredox catalyzed aziridine opening via SET reduction to form a separated radical anion species.^[^ ^34^, ^44^ ^]^ This intermediate could then undergo a radical cyclization with the arene of the *N*‐sulfonyl group, followed by RPC and final re‐aromatization to deliver the targeted benzo‐δ‐sultams.

To prove our hypothesis, we first chose *N*‐tosyl α‐ketoaziridine **1a** (*E*_1/2_(**1a**) = −1.34 V versus SCE, see Supporting Information. for details) as model substrate to investigate the RPC‐based ring expansion reaction (Table 1). Initially, *fac*‐Ir(ppy)_3_ (*E*_1/2_(Ir(III)*/Ir(IV)) = −1.73 V versus SCE)^[^ ^45^ ^]^ was used as suitable photoredox catalyst in anhydrous acetonitrile under blue light irradiation at room temperature for 18 h, leading to the desired product in a promising 27% yield (entry 1). In order to increase the yield of the reaction, different additives such as a Brønsted acid (entry 2), Brønsted base (entry 3) and Lewis base (entry 4) were next tested. However, instead of improving the desired reactivity, all these additives interfered with the reaction leading to side products. Moreover, the presence of moisture in the reaction media (use of wet MeCN) also resulted in a decrease in the yield of **2a** (entry 5). Next, we increased the catalyst loading from 1 to 4 mol% (entry 6), which resulted in a notable increase of the yield from 27% to 58%. Therefore, 4 mol% of photocatalyst was used as optimal loading for further optimization. Subsequently, different photocatalysts such as 5‐MeOCzBn (E*^ox^_1/2_ = −1.18 V versus SCE)^[^ ^46^ ^]^ or 4CzIPN (E*^ox^_1/2_ = −1.50 V versus SCE)^[^ ^46^ ^]^ (entries 7 and 8), and solvents (entries 9–12) were screened. Unfortunately, all these changes resulted in a significant drop of the yield respect to the previous conditions, while higher diastereoselectivities were generally obtained with more polar solvents. Similarly, the use of a less powerful light source (1 W versus 2 W; entry 13) also resulted in a less effective process. Eventually, we decided to screen a series of Lewis acids to induce a LUMO lowering of the substrate and favor the ring opening of the aziridine (entries 14–18). This revealed that LiClO_4_^[^ ^47^, ^48^ ^]^ (entry 15) gave a slightly improved yield and diastereoselectivity. Finally, the effect of the concentration was examined (entries 19 and 20). Both concentration and dilution led to lower yields, while the d.r. was maintained. In order to improve the diastereoselectivity, the reaction was run at 0 °C. However, the same d.r. was obtained, while showing low conversion (entry 21). It is also important to note that presence of oxygen shuts down drastically the photocatalytic activity, as only trace of product was observed with not degassed dry solvent (entry 22), while unreacted aziridine substrate could be mainly recovered.

**Table 1:** Optimization of the reaction conditions with α‐ketoaziridine **1a**.^a)^

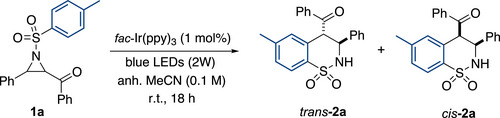			
Entry	Deviation from standard conditions	Yield (%)^b)^	d.r.^c)^ (*trans*/*cis*)
1	none	27	n.d.
2	+ PhCO_2_H (2 equiv.)	<5^d)^	–
3	+ Et_4_NHCO_3_ (2 equiv.)	<5^d)^	–
4	+ 2,6‐lutidine (2 equiv.)	<5^d)^	–
5	wet MeCN	18	n.d.
6	Ir(ppy)_3_ (4 mol%)	58	1.5:1
7	5‐MeOCzBn (4 mol%)	<5	–
8	4CzIPN (4 mol%)	<5	–
9	Ir(ppy)_3_ (4 mol%), in DMF	35	5.4:1
10	Ir(ppy)_3_ (4 mol%), in DMSO	26	6.7:1
11	Ir(ppy)_3_ (4 mol%), in DCE	<5	–
12	Ir(ppy)_3_ (4 mol%), in toluene	<5	–
13	Ir(ppy)_3_ (4 mol%), 1 W blue LED	40	n.d.
14	Ir(ppy)_3_ (4 mol%), LiOTf^e)^	59	1.5:1
15	Ir(ppy)_3_ (4 mol%), LiClO_4_ ^e)^	60(58)	1.8:1
16	Ir(ppy)_3_ (4 mol%), LiOAc^e)^	47	1.5:1
17	Ir(ppy)_3_ (4 mol%), Mg(OTf)_2_ ^e)^	46	n.d.
18	Ir(ppy)_3_ ( mol%), Sc(OTf)_3_ (20 mol%)	<5	–
19	Ir(ppy)_3_ (4 mol%), [0.2 M], LiClO_4_ ^e)^	39	1.8:1
20	Ir(ppy)_3_ (4 mol%), [0.05 M], LiClO_4_ ^e)^	36	1.8:1
21	Ir(ppy)_3_ (4 mol%), 0 °C, LiClO_4_ ^e)^	18	1.8:1
22	Ir(ppy)_3_ (4 mol%), LiClO_4_,^e)^ non‐degassed anh. MeCN	trace	n.d.

^a)^ Conditions: substrate **1a** (0.1 mmol) and *fac*‐Ir(ppy)_3_ (x mol%) in degassed anhydrous acetonitrile (0.1 M) under blue LED irradiation, 18 h at r.t. under Argon atmosphere.

^b)^ NMR‐yields were determined from the crude after workup of the reaction using 1,3,5‐trimethoxybenzene as internal standard. Isolated yields are given in brackets.

^c)^ The diastereomeric ratio was determined by NMR analysis of the crude reaction.

^d)^ The formation of a complex mixture of undesired side products mainly occurred.

^e)^ 1 equiv. used.

With the optimized conditions in hand – *fac*‐Ir(ppy)_3_ (4 mol%), LiClO_4_ (1 equiv.), in degassed, dry MeCN (0.1 M) – (entry 17), the scope of the reaction was explored (Scheme 2). The influence of different substituents at the arene ring on the sulfonyl group (Ar^1^ in Scheme 2) were initially tested (**2a** – **2h**). Different groups such as methyl, methoxy, chloro, bromo, and phenyl were easily tolerated, while the strong electron withdrawing nitro group only led to a low 10% yield (**2g**), which can be explained by the hampered radical cyclization step with the electrophilic key α‐carbonyl radical intermediate. However, the best results were obtained with the phenyl (**2b**) and tolyl (**2a** and **2c**) substitution. It is worth mentioning that for the other arylsulfonyl substituents (**2d‐2f**), a positive impact of LiClO_4_ on the yield was observed compared to the reaction without this additive, reinforcing its beneficial influence in the first SET step. Subsequently, the effect of the aryl substituent of the carbonyl group (R^2^ in Scheme 2) was investigated. In these experiments, it becomes clear that the presence of an aromatic group as substituent on the carbonyl to stabilize the ketyl radical‐anion formed during the SET is a strict requirement for the process to take place (**2i‐2r**). Thus, non‐stabilizing alkyl substituents of the keto group, such as methyl (**2u**) or *tert*‐butyl (**2v**), were not compatible with the process or only provided a decreased 6% NMR yield. Interestingly, rich heterocyclic aromatic systems such thiophene (**2t**) were also well accepted, while the substrate with a 2‐pyridylketone did not underwent the second radical cyclization step, providing the open product **2s’**. After all, different substituents on the aromatic ring were tolerated, providing the desired ring‐expanded products **2** in similar yields (51%–70%) and diastereoselectivities.

**Scheme 2:**
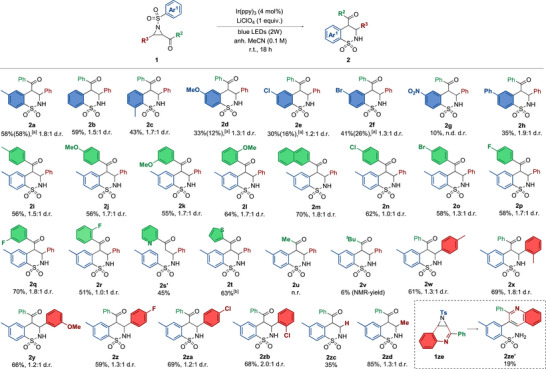
Scope of the reaction. Reactions were performed on a 0.1 mmol scale. Isolated yields are given. The d.r. was determined by NMR analysis of the crude. a) Yield of the reaction without LiClO_4_ as Lewis acid additive in brackets. b) d.r. could not be determined from the crude and only one isomer could be isolated.

We next explored diverse substituents on the aziridine (R^3^ in Scheme 2; **2w**‐**2ze**). Differently substituted aryl groups were well‐tolerated, including electron donating groups such as methyl (**2w** and **2x**) or methoxy (**2y**), as well as halogens (i.e., F, Cl) (**2z**‐**2zb**) in either *ortho*, *meta* or *para* positions, leading to homogeneous high yields (59%–69%). Moreover, an aryl group on the aziridine was not necessary to perform the reaction, as no substitution (**2zc**, 35%) or a methyl group (**2zd**, 85%) were also supported. Lastly, the method was tested with a fused 3,4‐dihydroquinoline‐aziridine **1ze** not presenting a ketone but a more challenging imine‐type unit. Interestingly, the dihydroquinoline functional group was tolerated, but the quinoline derivative **2ze’** was formed in an overall yield of 19%, following a similar ring‐opening/cyclization sequence to build the sultam, which suffers a final aromatization to gain the quinoline core with concomitant ring opening (see Supporting Information for details).

In order to illustrate the synthetic applicability of the methodology, a 10‐fold up‐scaling reaction (1 mmol scale) was performed (Scheme 3a). We could observe a comparable yield with the batch upscale to our standard reaction (54% versus 58%). Moreover, we could show exemplary derivatization of the **2a** product by *N*‐alkylation with benzyl bromide and K_2_CO_3_ as base, as well as with the Me_3_OBF_4_/DIPEA system, leading to the corresponding products as *trans*‐**3a** and *trans*‐**4a** in a 52% and 70% yield, respectively. In the case of *N*‐arylation attempts, we observed an intriguing reactivity, in which a double *N*‐arylation with an in situ formed aryne is favored by the enolizable carbonyl unit assisted ring opening. Based on the observed enrichment in the *trans*‐isomer during *N*‐alkylation reactions, we explored the possible epimerization upon treatment of a *cis*/*trans* product mixture with a base such as *^t^* BuOK (Scheme 3b). To our delight, a sample of **2a** with a 1.3:1 d.r. (57:43 *trans*/*cis*) could be efficiently epimerized in DMSO at room temperature towards the diastereoisomer *trans*‐**2a**, reaching a 92:8 *trans*/*cis* ratio (see Supporting Information. for details). Finally, the two diastereoisomers of **2a** could be crystallized separately. Thereby, the x‐ray structural analysis of both isomers^[^ ^49^ ^]^ corroborated the favored formation of the product with the *trans* configuration during the photocatalytic reaction (Scheme 3c).

**Scheme 3:**
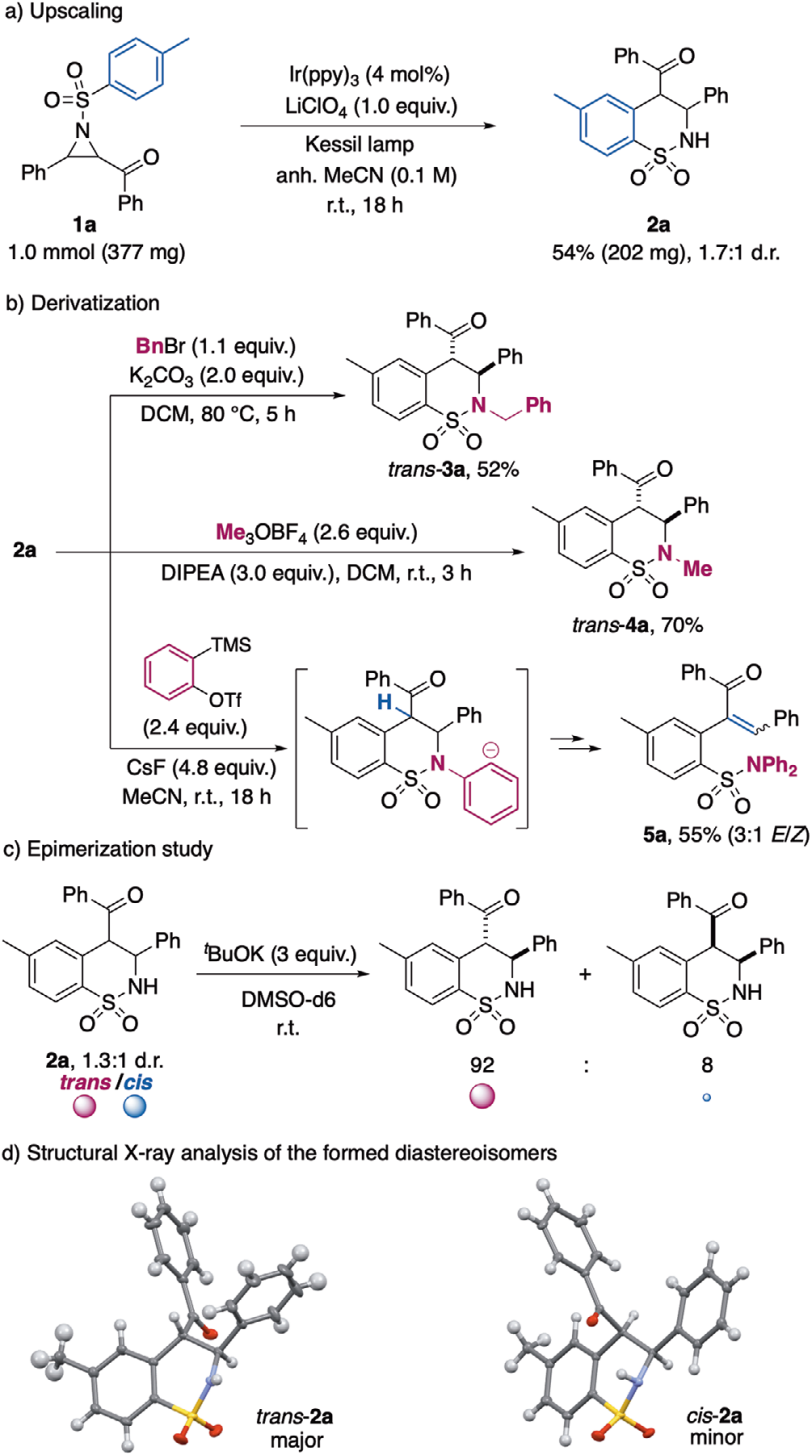
a) Upscaling of the model reaction, b) derivatization of **2a**, c) epimerization to the *trans*‐**2a** product upon treatment with base, and d) x‐ray analysis of the relative configuration of the **2a**‐isomers.

To gain more insights into the mechanism of the process, several experiments were carried out. In first place, we performed Stern–Volmer quenching and cyclic voltammetry studies (see Supporting Information for details) to evaluate the proposed initial reduction of the substrate **1a** by the iridium photocatalyst in the absence and presence of LiClO_4_ as Lewis acid. The photocatalyst's fluorescence quenching was not significantly affected by the presence of the Lewis acid (see Figures S14 and S15), while this additive influences the reduction potential of **1a**. As anticipated, the presence and LUMO lowering effect of the Lewis acid affects the redox potential of **1a** and makes its reduction more favorable (*E*_1/2_(**1a**‐LiClO_4_) = −1.20 V versus *E*_1/2_(**1a**) = −1.34 V versus SCE), whereas the direct reduction of the substrate by *fac*‐Ir(ppy)_3_ is thermodynamically favorable as it presents a less negative potential than this catalyst (*E*_1/2_(Ir(III)*/Ir(IV) = −1.73 V versus SCE).^[^ ^45^ ^]^ This supports the hypothesized single electron transfer nature of the initial interaction between the catalyst and the substrate to generate a ketyl radical‐anion.

Next, a radical trapping experiment with 2,6,6‐tetramethylpiperidine 1‐oxyl radical (TEMPO) and benzoic acid as proton source was performed aiming at determining key carbon‐radical intermediates and the site of the ring opening of the aziridine (Scheme 4a). The trapped product **6a** was isolated in 62% yield (≥90%, detected by LC‐MS), showing that the ring opening is occurring by radical generation in alpha to the carbonyl group.

**Scheme 4:**
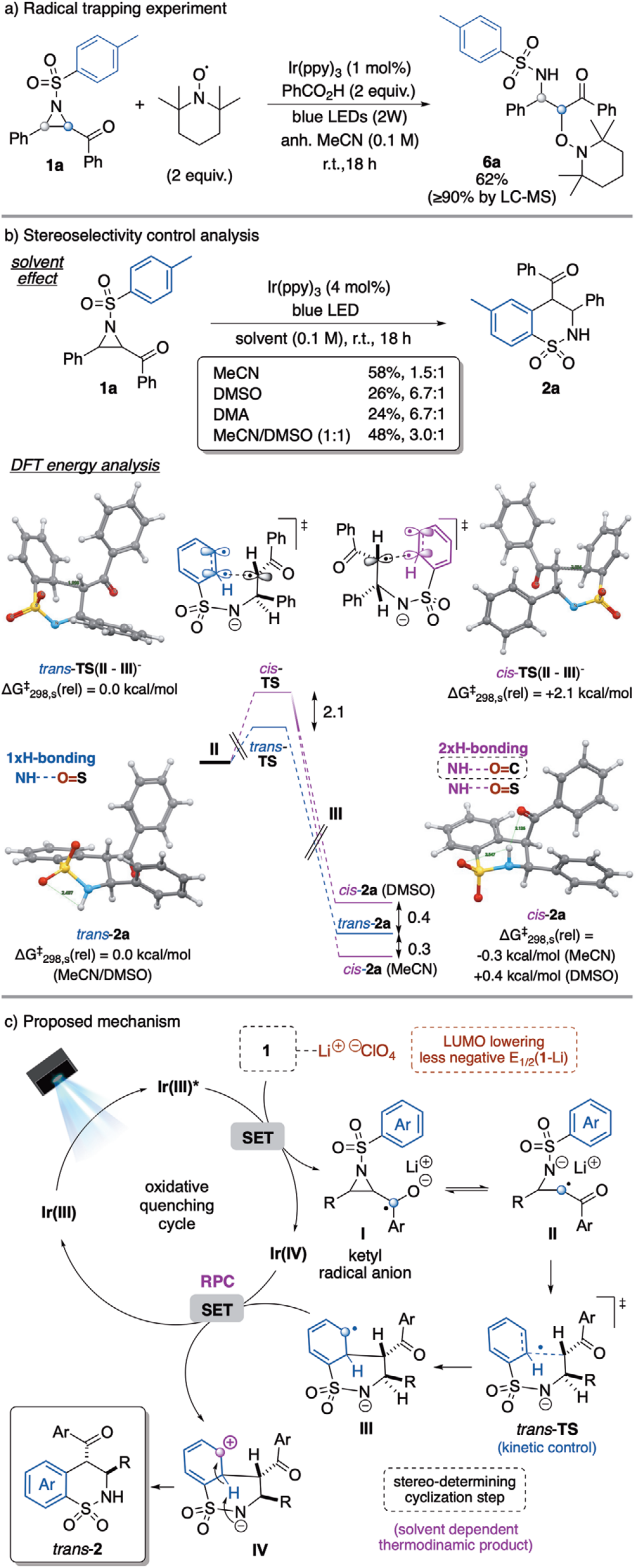
Mechanistic investigations: a) radical trapping experiment, and b) stereochemical outcome evaluation by solvent effect study and DFT calculated energy analysis of *trans*‐**2a** and *cis*‐**2a** products and transition states (**TS**) for the cyclization step. All energies in kcal/mol. c) Proposed plausible mechanism.

Finally, seeking a better understanding of the factors responsible of the diastereoselectivity outcome of the reaction, experimental and computational studies were carried out (Scheme 4b). We considered more carefully the solvent effect as an enhanced selectivity towards the *trans*‐isomer in more polar and greater hydrogen bond acceptors than acetonitrile such as DMSO or DMA was recorded. Interestingly, the reaction run in a co‐solvent mixture of MeCN/DMSO (1:1) led to a diastereoselectivity lying in between the ones in the pure solvents. This might indicate the ability of the solvent system to stabilize or destabilize one of the diastereoisomers by hydrogen bonding (HB) interactions with the sultam NH group, which might be crucial for the stereochemical outcome. Next, computational DFT calculations on the relative energies of the products **2a** and transition states (TS) of the stereo‐determining radical cyclization step were performed (see Supporting Information for details).

Analysis of the computed optimized *trans*‐**TS** and *cis*‐**TS** revealed a more favorable *trans*‐pathway, presenting a 2.1 kcal/mol lower activation energy (kinetic control). However, in acetonitrile the *cis*‐**2a** product is slightly thermodynamically more stable (Δ*G* ^‡^_298,s_(rel) = −0.3 kcal/mol). This can be explained by an additional stabilization of this isomer by HB between the NH group of the sultam and the carbonyl group, which is not present in the *trans* isomer. This situation is inverted in DMSO in which this interaction is disrupted, becoming *trans*‐**2a** the thermodynamic product. This is in accordance with the observed enhanced *trans*‐selectivity in DMSO. Nonetheless, the differences in energy of the two isomers are very small, leading to the low to moderate diastereoselectivity obtained.

Based on all these observations, as well as on previous reports on reductive ring opening of α‐ketoaziridines^[^ ^34^ ^]^ and radical intramolecular radical cyclization with aromatic units,^[^ ^23^ ^]^ we propose a mechanism involving a radical‐polar crossover (RPC) reaction pathway, as shown in Scheme 4c. According to it, the iridium catalyst is excited upon visible light irradiation, leading to an excited state able to reduce the substrate (**1**) through a SET process to the carbonyl group, favored by the Lewis acid additive. The newly formed radical‐anion undergoes a radical ring opening driven by ring‐strain release to build the key radical‐amidate intermediate **II** presenting the C‐centered radical at the alpha position of the carbonyl group. This species is then involved in an intramolecular radical cyclization with the *N*‐arylsulfonyl group, creating the sultam six‐member cycle. The so‐formed dearomatized intermediate **III** undergoes a second SET with the oxidized Ir(IV) species, leading by RPC to the polar intermediate **IV** and closing the catalytic cycle by regeneration of the Ir(III) photocatalyst. The zwitterionic intermediate **IV** is then evolved to the final product (**2**) by either stepwise re‐aromatization–amidate protonation or through a direct intramolecular 1,5‐hydrogen shift.

In conclusion, we successfully developed a novel photoredox‐catalyzed methodology for the ring expansion reaction of α‐ketoaziridines, providing readily access to δ‐sultams. This process shows a high functional group tolerance, as well as a broad variety of substitution patterns at the aryl sulfonyl unit or aziridine core, allowing the ring expansion of a wide range of substrates. Additionally, we demonstrated the synthetic utility of our method by 10‐fold up‐scaling of the model reaction and exemplary derivatization of **2a**. The epimerization of the product mixture under basic conditions towards the more favorable *trans* isomer was achieved, as well as the crystallization of the two obtained diastereoisomers of **2a**. This allowed the determination of the relative configuration of the isomers. Furthermore, mechanistic investigations revealed that the transformation is initiated by a reductive SET process to the carbonyl of the α‐ketoaziridine, followed by ring‐opening and intramolecular radical cyclization of the *N*‐arylsulfonyl group with the α‐carbonyl radical arising from the ring opening of the aziridine. The latter is the stereo‐determining step, in which the formation for the kinetic *trans*‐**2** product (lower TS energy) competes with the thermodynamically more stable *cis*‐**2** in acetonitrile, which explains the moderate stereoselectivites observed. However, increasing the HB acceptor ability of the solvent inverts the stability of the products by disfavoring intramolecular HB‐interactions in the *cis*‐isomer and leads to an appreciable enhancement of selectivity towards the *trans*‐isomer. Finally, after RPC by a second SET to build the corresponding aryl cation and rearomatization, the sultam product is formed. This work provides valuable synthetic alternative to the synthesis of sultams, and offers new directions towards photoredox‐mediated ring opening‐expansion strategies towards to the construction of a broad variety of heterocyclic molecules.

## Supporting Information

The authors have cited additional references within the Supporting Information.^[^ ^50^, ^51^, ^52^, ^53^, ^54^, ^55^, ^56^, ^57^, ^58^, ^59^, ^60^, ^61^, ^62^, ^63^, ^64^, ^65^, ^66^, ^67^, ^68^ ^]^

## Conflict of Interests

The authors declare no conflict of interest.

## Supporting information



Supporting Information

Supporting Information

## Data Availability

The data that support the findings of this study are available in the supplementary material of this article. Part of the research data is openly available in ChemRxiv at 10.26434/chemrxiv‐2025‐mfkrt.
