# Supplementary material for: Photocatalyzed Ring Expansion of α‐Ketosulfonylaziridines: Ready Access to δ‐Sultams
Source: Angew Chem Int Ed Engl. 2025 Dec 8;65(4):e16731. doi: 10.1002/anie.202516731 (PMC12828454; doi:10.1002/anie.202516731)

Photocatalyzed Ring Expansion of α-Ketosulfonylaziridines: Ready Access to δ-Sultams

Marco M. Mastandrea,†[e] Stefania Perulli,†[a] Vittoria Martini,[a] Ludovica Primitivo,[e] Lei-Jie Zhou,[e] Christian Mück-Lichtenfeld,[a] Miquel A. Pericàs*[c,d] and Olga García Mancheño*[a,b]

[a] S. Perulli, Dr. V. Martini, Dr. C. Mück-Lichtenfeld, Prof. Dr. O. García Mancheño
Organic Chemistry Institute, University of Münster, Corrensstraße 36/40, Münster 48149, Germany
E-mail: olga.garcia@uni-muenster.de

[b] Prof. Dr. O. García Mancheño
Leibniz Institute for Catalysis
Albert-Einstein-Straße 29A, 18059 Rostock, Germany

[c] Prof. Dr. M. A. Pericàs
Departament de Química Física i Inorgànica, Universitat Rovira i Virgili, Campus Sescelades, Marcel.lí Domingo s/n, 43007 Tarragona, Spain
E-mail: miquelangel.pericas@urv.cat

[d] Prof. Dr. M. A. Pericàs
Royal Academy of Sciences and Arts of Barcelona, Chemistry Section
La Rambla 115, 08002 Barcelona, Spain

[e] Dr. M. M. Mastandrea, Dr. L. Primitivo, Dr. L.-J. Zhou
Institute of Chemical Research of Catalonia (ICIQ) Av. Països Catalans, 16, 43007, Tarragona, Spain

† These authors contributed equally to this work.

CONTENTS

1. General Information 2
2. Control Experiments 4
3. Synthesis of α-Ketoaziridines 7
4. Photocatalyzed Radical-Polar Crossover Ring-Expansion and Derivatization of **2a** 16
5. Mechanistic Studies 27
6. X-Ray Structural Analysis for **2a** 33
7. DFT calculations.. 35
8. References 46
9. NMR Spectra 47

1. General Information

If not specified, all reagents were purchased and used without any further purification. The reaction progress was checked by thin layer chromatography (TLC). Therefore aluminium-foil backed silica TLC plates with a fluorescent indicator (TLC Silica gel 60 F254) from Merck were used. Compounds were detected either with a UV lamp (λ = 254 nm) or by employing a *p*-anisaldehyde stain in sulfuric acid, acetic acid and ethanol. For column chromatography, Aldrich flash grade silica gel (230-400 mesh) was used as a stationary phase.

Fluorescence quenching experiments were performed on a Jasco FP-8500 spectrofluorometer with self-dried MeCN (MeCN was dried by distilling the solvent over CaH_2).High-resolution mass spectra (HRMS) were carried out using High Resolution Mass Spectrometry on Waters GCT gas chromatograph coupled time-of-flight mass spectrometer (GC/MS-TOF) with electron ionization (EI) or alternately were recorded on a Bruker MicroTof ESI spectrometer or a Thermo Fisher Scientific Orbitrap LTQ XL spectrometer using electrospray ionization (ESI) techniques.

^{1}H and ^{13}C and spectra were recorded in $CDCl_3$ (reference signals: ^{1}H = 7.26 ppm, ^{13}C = 77.16 ppm, $CDCl_3$), on a Bruker Avance II 300, Bruker Avance II 400 NMR, Bruker Avance 400 or Bruker Avance 500 Ultrashield. The chemical shifts (δ) were given in ppm and the coupling constants (*J*) are reported in Hz. Multiplicities were abbreviated by s (singlet), bs (broad singlet), d (doublet), t (triplet), m (multiplet), bm (broad multiplet). All products that are known were characterized by comparison of their physical and spectroscopic properties with those described in the literature.

Irradiation setup 1: *small-scale reactions*: a five single blue LEDs photoreactor has been used (Figure S1). The overall setup allows for the simultaneous irradiation of five 10 mL reaction vessels under magnetic stirring at constant temperature (internal measured temperature of 25 ºC). The measured emission intensity of the blue LEDs at 700 mA is 75 mW/cm^2. LEDs were purchased from Digi-Key.

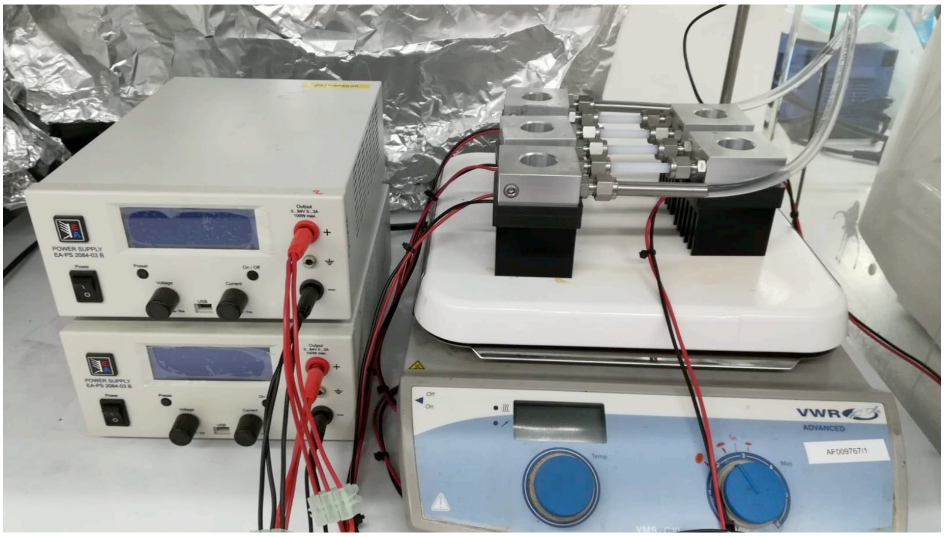

Figure S1. *Irradiation Setup 1 used in intramolecular aziridine ring expansion reactions.*

Irradiation setup 2: *small-scale reactions*: Reactions were performed employing a custom-built photoreactor, manufactured by the precision engineering workshop of the organic chemistry institute of the University of Münster (Figure S2). Headspace vials with crimp neck (10 mL) were used and sealed with aluminum crimp caps with septum. The vials were irradiated from the bottom by a 5 W LED 455-460 nm (purchased from Avonec; product nr. 5W455460m) under defined temperature (20 °C) adjusted by a Huber Minichiller 280.

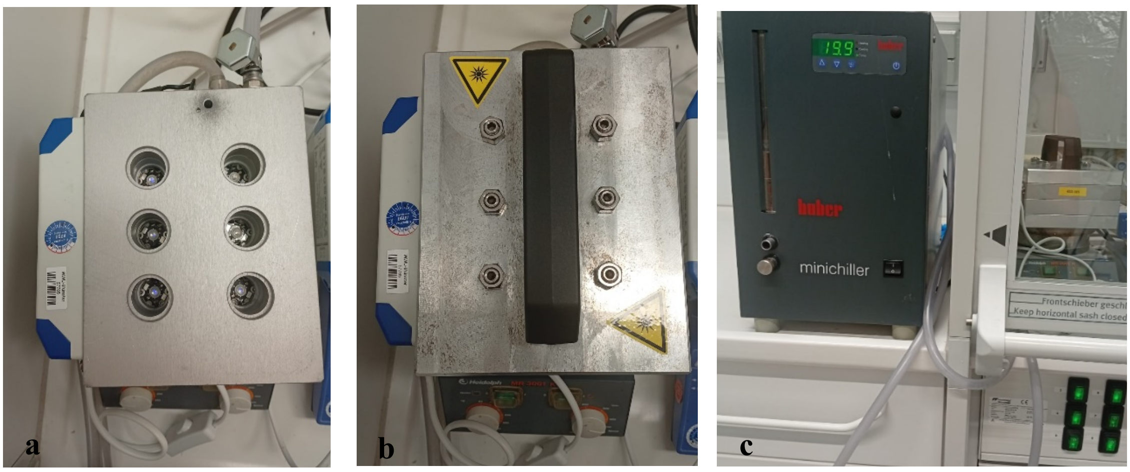

Figure S2. *a) and b) custom-build photoreactor for small scale; c) water cooling system.*

Irradiation setup 3: *gram scale reaction.* A 9 cm crystallizing dish with a 1-meter blue LED strip wrapped around it was used as photoreactor, and the reactor was cooled using a fan.

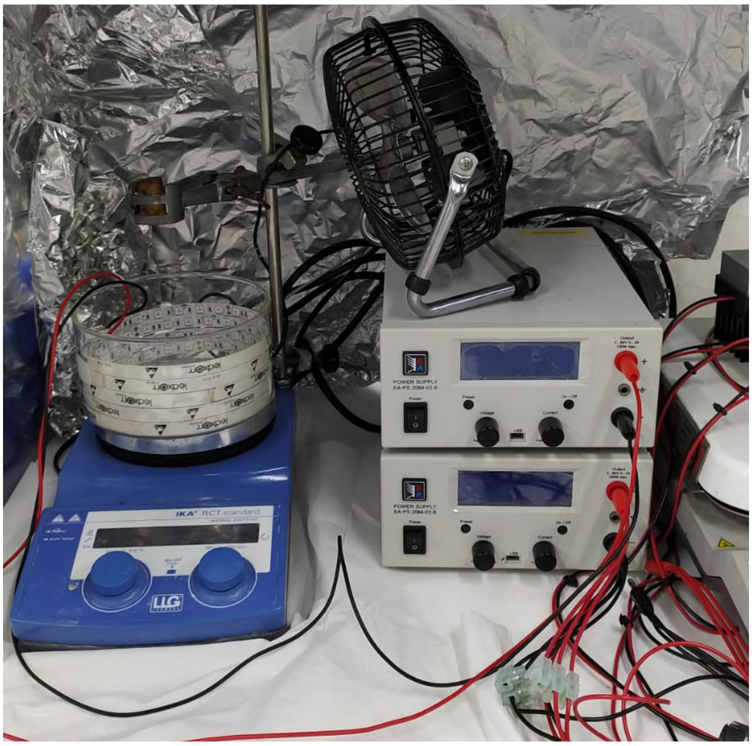

Figure S3. *Irradiation setup 2 used in gram scale intramolecular aziridine ring expansion reaction.*

Irradiation setup 4: *gram scale reaction.* The reaction mixture was placed in a finger Schlenk tube and stirred under the irradiation of two Kessil H150 Blue lamps with fans to cool down the system.

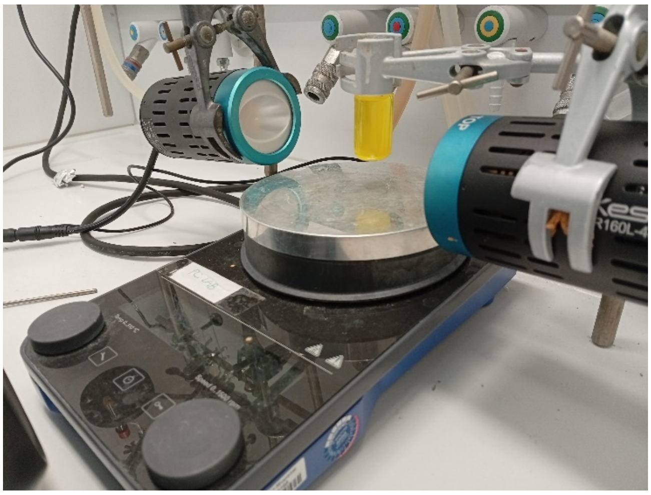

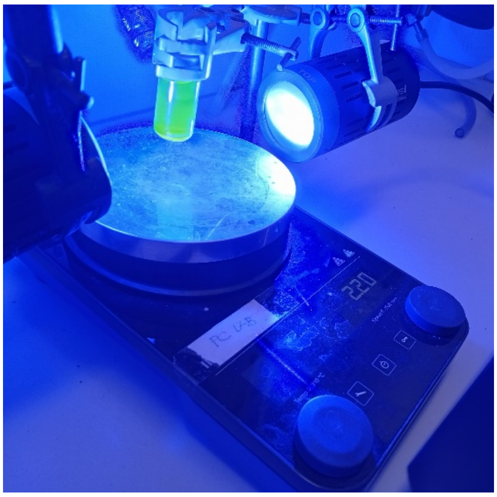

Figure S4. *Irradiation for big scale reactions.*

2. Control Experiments

Table S1. *Control experiments for the model reaction with **1a**.*

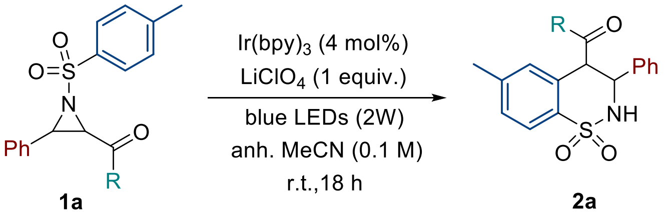

Entry	Deviation from standard conditions	Yield
1	no catalyst (R = Ph)	0%
2	no light (R =Ph)	0%
3	R = Me (1q)	0%
4	R = *t*-Bu (1r)	6%*

* NMR yield

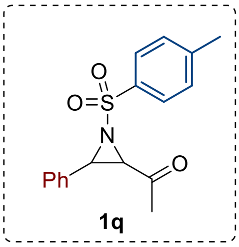

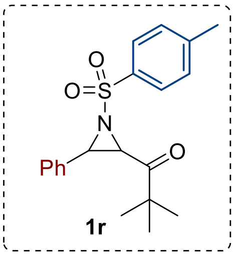

Monitoring of the intramolecular aziridine ring expansion reaction

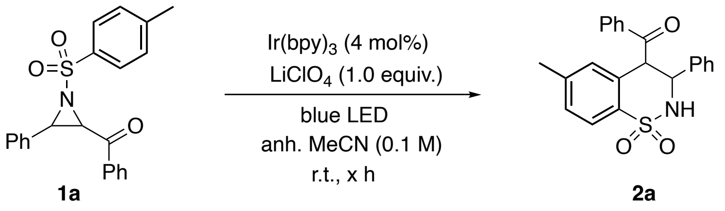

Entry	Time	Conversion	NMR-yield
1	2 h	77%	41%
2	3 h	89%	50%
3	4 h	89%	50%
4	24 h	99%	58%

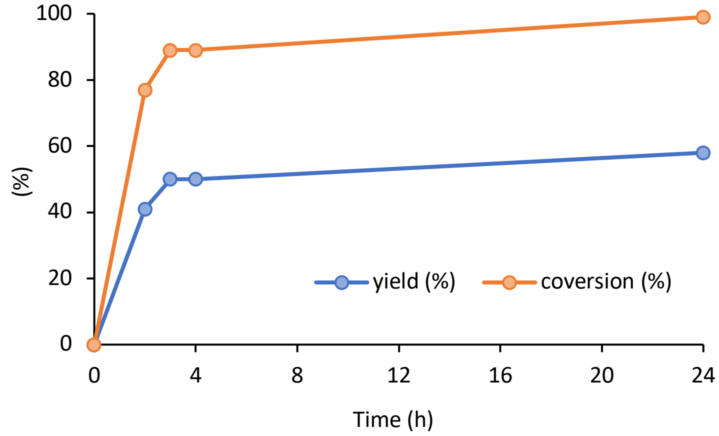

Diastereoselectivity monitoring:

Entry	Time	d.r. (*cis*:*trans*)
1	1 h	1 : 2.3
2	2 h	1 : 1.8
3	3 h	1 : 1.8
4	4 h	1 : 1.5

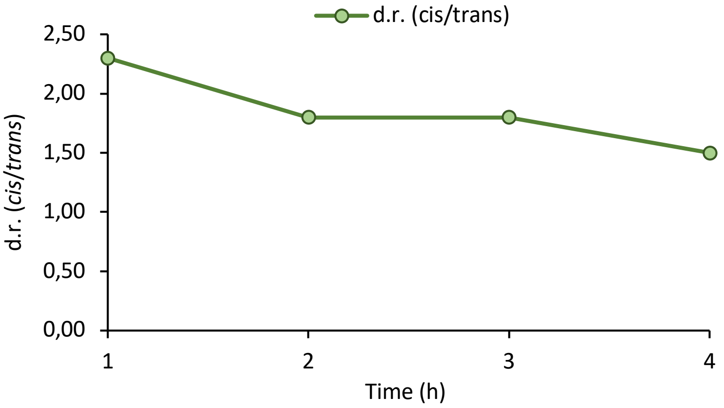

Study of diastereoselectivity photodegradation with 2a and proposed mechanism

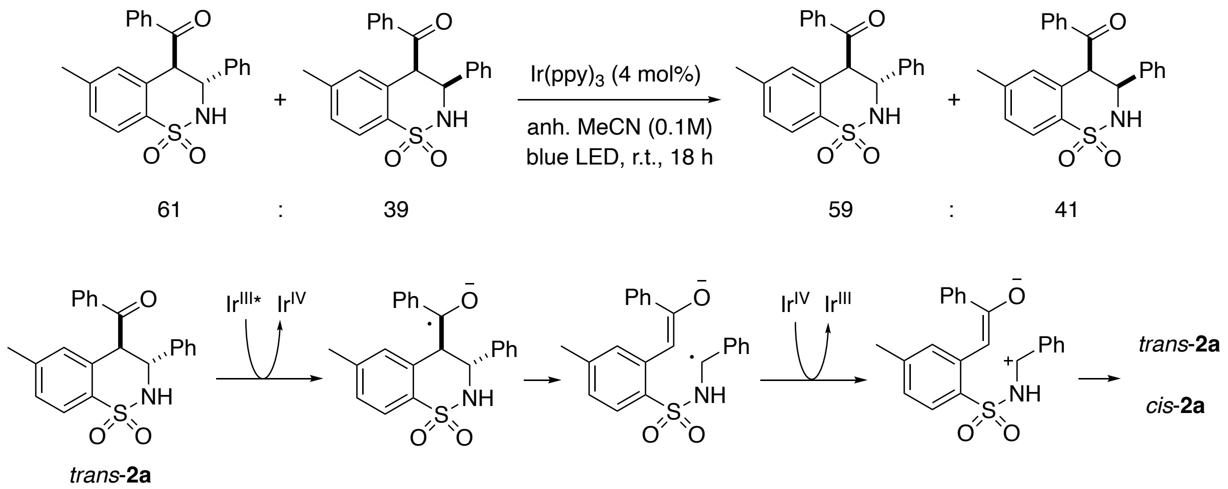

Epimerization study of a 1.3:1 *trans*/*cis*-mixture of 2a under basic conditions

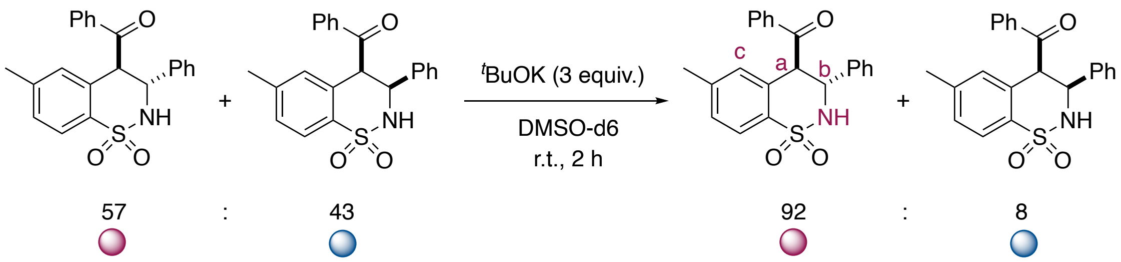

^{1}H NMR (DMSO-d6, 400 MHz)

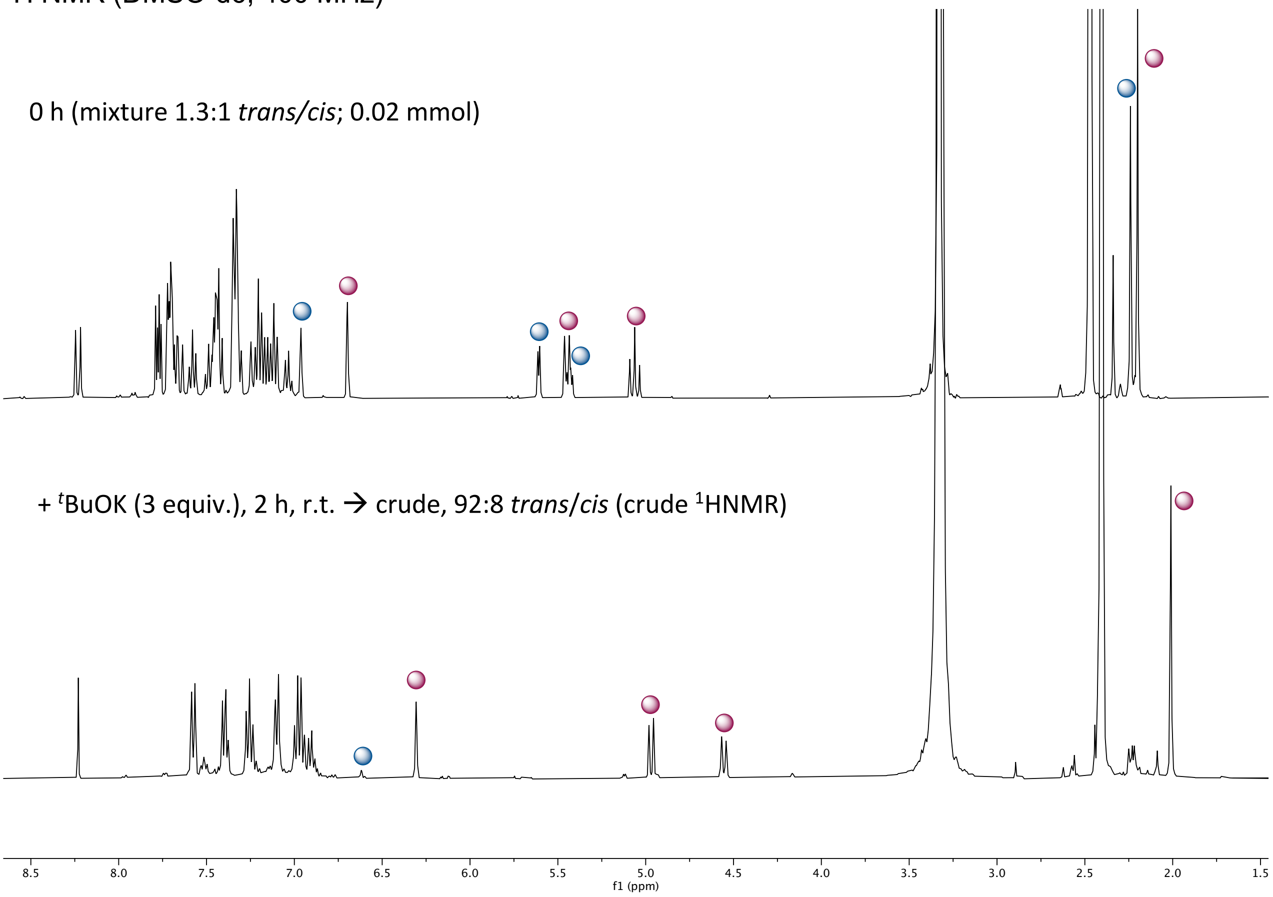

After extraction with Et_2O/H_2O: ^{1}H NMR ($CDCl_3$, 400 MHz)

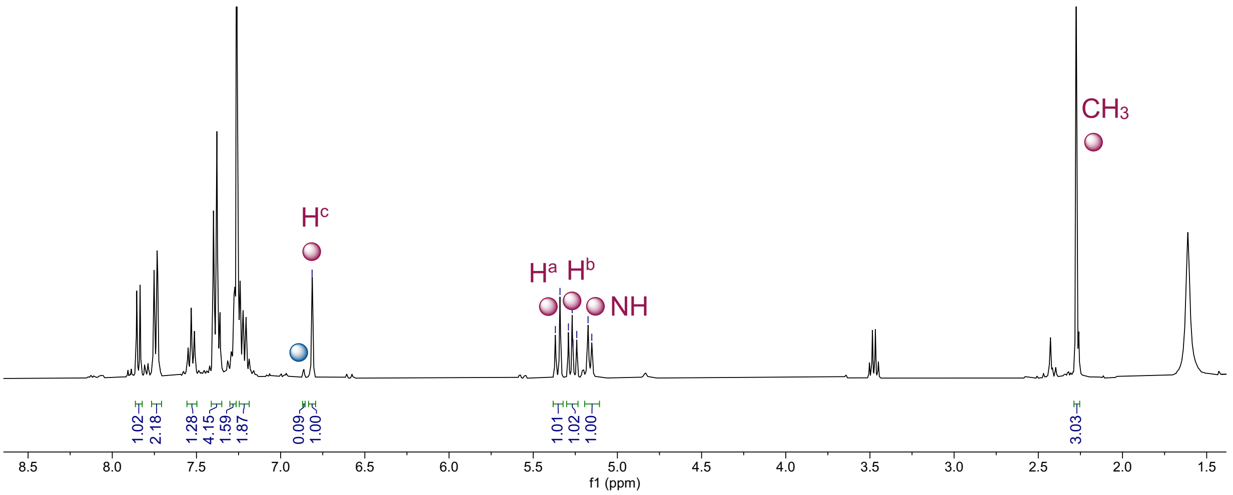

3. Synthesis of α-Ketoaziridines

General procedure for the synthesis of aziridines 1a-g, 1i-zb and 1ze (GP1)

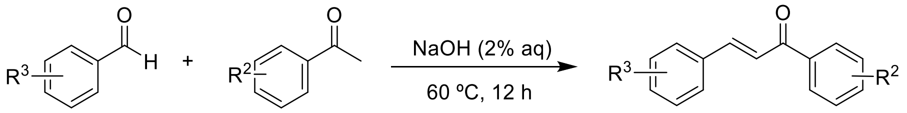

Scheme S1. *Synthesis of not commercially available chalcones.*

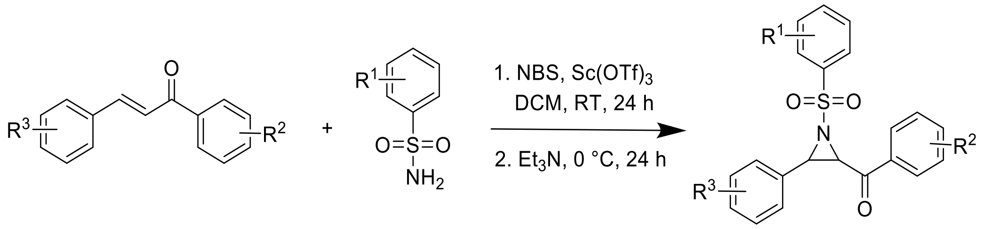

Scheme S2. *Synthesis of aziridines **1a-g, 1i-zb and 1ze** from chalcones.*

The not commercially available chalcones, were synthetized according to following synthetic procedure: a mixture of the corresponding acetophenone (1.02 equiv.) and the corresponding aromatic aldehyde (1.00 equiv.) in NaOH (2% aq) (3 mL/mmol of aldehyde) was stirred at room temperature for 30 minutes. Then, the reaction mixture was stirred at 60 °C overnight. Afterwards, the precipitated chalcones were filtered out from the reaction mixture, dried under vacuum and taken to the next step without further purification.

To a solution of chalcone (5.80 mmol) and 4Å molecular sieves (500.0 mg) in dry DCM (11 mL) was added, under argon atmosphere, the corresponding arylsulfonamide (6.30 mmol), NBS (1.228 g, 6.90 mmol) and $Sc(OTf)_3$ (143.0 mg, 0.29 mmol). Reaction mixture was stirred at r.t. overnight. Then, anhydrous Et_3N (970 µL, 6.96 mmol) was added at 0 ºC and reaction mixture was stirred at room temperature overnight. Afterwards, reaction mixture was filtered and concentrated in vacuo. The desired product was isolated by column-flash chromatography (Cyclohexane:EtOAc/ 9:1).

Phenyl(3-phenyl-1-tosylaziridin-2-yl)methanone (1a)

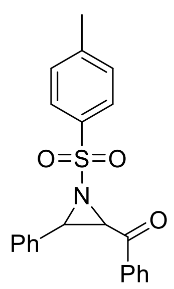

Following GP1, **1a** was prepared starting from (*E*)-chalcone and *p*-toluenesulfonamide. It was obtained as a 1.9:1 *cis/trans* mixture and isolated as a white solid (1.970 g, 90% yield). **^{1}H NMR** (400 MHz, $CDCl_3$) (*cis/ trans* mixture) δ 8.07 – 8.03 (m, 2H *trans*), 7.98 (d, *J* = 8.3 Hz, 2H *cis*), 7.88 – 7.83 (m, 2H *cis*), 7.72 (d, *J* = 8.3 Hz, 2H *trans*), 7.62 (ddt, *J* = 8.1, 6.9, 1.3 Hz, 1H *trans*), 7.55 – 7.45 (m, 3H *cis* + *trans*), 7.42 – 7.32 (m, 9H *cis* + *trans*), 7.25 – 7.14 (m, 8H *cis* + *trans*), 4.52 (d, *J* = 4.2 Hz, 1H *trans*), 4.41 (d, *J* = 7.7 Hz, 1H *cis*), 4.36 (d, *J* = 7.7 Hz, 1H *cis*), 4.29 (d, *J* = 4.2 Hz, 1H *trans*), 2.44 (s, 3H *cis*), 2.40 (s, 3H *trans*). **^{13}C NMR** (100 MHz, $CDCl_3$) (*cis/ trans* mixture) δ 190.5, 189.1, 145.3, 144.5, 136.8, 136.2, 135.8, 134.6, 134.2, 133.9, 133.1, 131.3, 130.0, 129.6, 129.1, 129.0, 129.0, 128.9, 128.8, 128.8, 128.6, 128.5, 128.4, 128.2, 127.9, 127.7, 127.5, 50.3, 48.3, 47.7, 46.7, 21.8, 21.7. These data are in agreement with those reported in literature.[50]

Phenyl(3-phenyl-1-(phenylsulfonyl)aziridin-2-yl)methanone (1b)

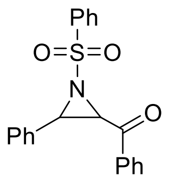

Following GP1, **1b** was prepared starting from (*E*)-chalcone and benzenesulfonamide. It was obtained as a 1.7:1 *cis/trans* mixture and isolated as a white solid (1.876 g, 89% yield). **^{1}H NMR** (400 MHz, $CDCl_3$) (*cis/ trans* mixture) δ 8.12 – 8.10 (m, 2H *cis*), 8.07 – 8.04 (m, 2H *trans*), 7.87 – 7.84 (m, 4H *cis* + *trans*), 7.68 – 7.37 (m, 16H *cis* + *trans*), 7.24 – 7.16 (m, 6H *cis* + *trans*), 4.54 (d, *J* = 4.2 Hz, 1H *trans*), 4.45 (d, *J* = 7.7 Hz, 1H *cis*), 4.40 (d, *J* = 7.7 Hz, 1H *cis*), 4.32 (d, *J* = 4.2 Hz, 1H *trans*). **^{13}C NMR** (100 MHz, $CDCl_3$) (*cis/ trans* mixture) δ 190.4, 189.0, 139.8, 137.7, 136.1, 135.8, 134.3, 134.2, 134.0, 133.5, 133.0, 131.2, 129.4, 129.1, 129.1, 129.0, 129.0, 128.8, 128.8, 128.8, 128.7, 128.5, 128.5, 128.1, 127.8, 127.6, 127.4, 50.4, 48.4, 47.8, 46.7. These data are in agreement with those reported in literature.[50]

Phenyl(3-phenyl-1-(*o*-tolylsulfonyl)aziridin-2-yl)methanone (1c)

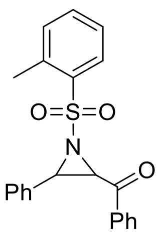

Following GP1, **1c** was prepared starting from (*E*)-chalcone and 2-methylbenzenesulfonamide. It was obtained as a 1.7:1 *cis*/*trans* mixture and isolated as a white solid (1.751 g, 80% yield). **^{1}H NMR** (400 MHz, $CDCl_3$) (*cis/trans* mixture) δ 8.04 (ddd, *J* = 8.6, 4.8, 1.5 Hz, 2H *cis*), 7.83 (dd, *J* = 8.4, 1.3 Hz, 2H *cis*), 7.72 (dd, *J* = 7.9, 1.4 Hz, 1H *trans*), 7.61 (ddt, *J* = 7.8, 6.9, 1.3 Hz, 1H *trans*), 7.52 (ddt, J = 8.5, 5.7, 1.3 Hz, 2H *cis*), 7.48 – 7.29 (m, 14H, *cis* + *trans*), 7.25 – 7.23 (m, 2H *cis*), 7.20 – 7.17 (m, 4H, *cis* + *trans*), 4.61 (d, *J* = 4.2 Hz, 1H *trans*), 4.47 – 4.41 (m, 2H *cis*), 4.28 (d, *J* = 4.2 Hz, 1H *trans*), 3.00 (s, 3H *cis*), 2.76 (s, 3H *trans*). **^{13}C NMR** (100 MHz, $CDCl_3$) (*cis/trans* mixture) δ 190.57, 189.1, 139.8, 138.7, 138.1, 136.2, 135.9, 135.8, 134.2, 134.0, 133.5, 133.4, 133.0, 132.5, 131.5, 129.5, 129.2, 129.1, 129.0, 128.9, 128.8, 128.7, 128.5, 128.4, 127.6, 127.5, 126.2, 126.0, 50.6, 48.6, 47.5, 46.6, 20.9, 20.8. **HRMS** (ESI) m/z calculated for ($C_{22}H_{19}NNaO_3S$) 400.0978 $[M+Na]^+$; found 400.0983.

(1-((4-Methoxyphenyl)sulfonyl)-3-phenylaziridin-2-yl)(phenyl)methanone (1d)

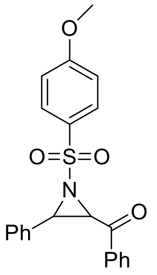

Following GP1, **1d** was prepared starting from (*E*)-chalcone and 4-methoxybenzenesulfonamide. The *cis* isomer was isolated as a white solid (1.414 g, 62% yield). **^{1}H NMR** (400 MHz, $CDCl_3$) (*cis* stereoisomer) δ 8.02 (d, *J* = 8.9 Hz, 2H), 7.88 – 7.83 (m, 2H), 7.56 – 7.50 (m, 1H), 7.42 – 7.36 (m, 2H), 7.24 – 7.14 (m, 5H), 7.02 (d, *J* = 9.0 Hz, 2H), 4.39 (d, *J* = 7.7 Hz, 1H), 4.34 (d, *J* = 7.7 Hz, 1H), 3.87 (s, 3H). **^{13}C NMR** (100 MHz, $CDCl_3$) (*cis* stereoisomer) δ 189.2, 164.1, 135.8, 133.9, 131.4, 130.5, 128.8, 128.7, 128.6, 128.5, 128.4, 127.5, 114.6, 55.8, 48.3, 46.7. **HRMS** (ESI) m/z calculated for ($C_{22}H_{19}NNaO_4S$) 416.0927 $[M+Na]^+$; found 416.0927.

(1-((4-Chlorophenyl)sulfonyl)-3-phenylaziridin-2-yl)(phenyl)methanone (1e)

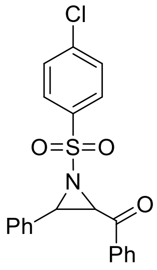

Following GP1, **1e** was prepared starting from (*E*)-chalcone and 4-chlorobenzenesulfonamide. The *cis* isomer was isolated as a white solid (992.0 mg, 43% yield). **^{1}H NMR** (500 MHz, $CDCl_3$) (*cis* diastereomer) δ 8.05 (d, *J* = 8.7 Hz, 2H), 7.85 (dd, *J* = 8.4, 1.3 Hz, 2H), 7.57 – 7.51 (m, 3H), 7.40 (dd, *J* = 8.2, 7.4 Hz, 2H), 7.25 – 7.16 (m, 5H), 4.45 (d, *J* = 7.7 Hz, 1H), 4.41 (d, *J* = 7.8 Hz, 1H). **^{13}C NMR** (126 MHz, $CDCl_3$) (*cis* diastereomer) δ 188.7, 140.9, 136.2, 135.6, 134.1, 131.0, 129.8, 129.6, 128.9, 128.8, 128.5, 128.4, 127.4, 48.7, 46.9. **HRMS** (ESI) m/z calculated for ($C_{21}H_{16}ClNNaO_3S$) 420.0429 $[M+Na]^+$; found 420.0432.

(1-((4-Bromophenyl)sulfonyl)-3-phenylaziridin-2-yl)(phenyl)methanone(1f)

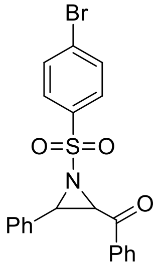

Following GP1, **1f** was prepared starting from (*E*)-chalcone and 4-bromobenzenesulfonamide. The *cis* isomer was isolated as a white solid (1.077 g, 42% yield). **^{1}H NMR** (500 MHz, $CDCl_3$) (*cis* diastereomer) δ 7.97 (d, *J* = 8.7 Hz, 2H), 7.88 – 7.83 (m, 2H), 7.71 (d, *J* = 8.8 Hz, 2H), 7.56 – 7.51 (m, 1H), 7.43 – 7.37 (m, 2H), 7.25 – 7.16 (m, 5H), 4.45 (d, *J* = 7.8 Hz, 1H), 4.41 (d, *J* = 7.8 Hz, 1H). **^{13}C NMR** (126 MHz, $CDCl_3$) (*cis* diastereomer) δ 188.7, 136.8, 135.6, 134.1, 132.8, 131.0, 129.6, 129.5, 128.9, 128.9, 128.6, 128.5, 127.4, 48.7, 46.9. **HRMS** (ESI) m/z calculated for ($C_{21}H_{16}BrNNaO_3S$) 463.9907 $[M+Na]^+$; found 463.9926.

(1-((4-Nitrophenyl)sulfonyl)-3-phenylaziridin-2-yl)(phenyl)methanone (1g)

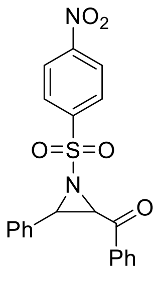

Adapting GP1 on 8.08 mmol scale, **1g** was prepared starting from (*E*)-chalcone (1.680 g, 8.08 mmol, 1 equiv.) and 4-nitrobenzenesulfonamide. The *cis* isomer was obtained and isolated as an orange foamy solid (230.0 mg, 9% yield). **^{1}H NMR** (400 MHz, $CDCl_3$) (*cis* diastereomer) δ 8.45 – 8.39 (m, 2H), 8.37 – 8.30 (m, 2H), 7.89 – 7.82 (m, 2H), 7.47 – 7.37 (m, 3H), 7.28 – 7.15 (m, 5H), 4.54 – 4.50 (two overlapped doublets, d, *J* = 7.8 Hz, 2H). **^{13}C NMR** (101 MHz, $CDCl_3$) (*cis* diastereomer) δ 188.2, 151.0, 143.7, 135.3, 134.3, 130.6, 129.4, 129.1, 129.0, 128.7, 128.4, 127.3, 124.6, 49.4, 47.1. **HRMS** (ESI) m/z calculated for ($C_{21}H_{16}N_{25}SNa$) 431.0672 $[M+Na]^+$; found 431.0672.

(3-Phenyl-1-tosylaziridin-2-yl)(*p*-tolyl)methanone (1i)

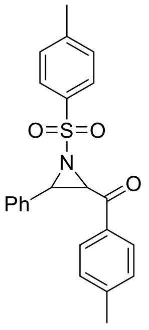

Following GP1, **1i** was prepared starting from 1-(2-methoxyphenyl)-3-phenylprop-2-en-1-one and *p*-toluenesulfonamide. It was obtained as a 1:2 *cis/trans* mixture and isolated as a white solid (1.907 g, 84% yield). **^{1}H NMR** (500 MHz, $CDCl_3$) (*cis/trans* mixture) δ 7.99 – 7.94 (m, 4H, *cis* + *trans*), 7.77 (d, *J* = 8.3 Hz, 2H *cis*), 7.72 (d, *J* = 8.3 Hz, 2H *trans*), 7.36 – 7.33 (m, 9H, *cis* + *trans*), 7.28 (d, *J* = 7.9 Hz, 2H *trans*), 7.24 – 7.22 (m, 4H, *cis* + *trans*), 7.20 – 7.14 (m, 3H, *cis* + *trans*), 4.50 (d, *J* = 4.3 Hz, 1H *trans*), 4.39 (d, *J* = 7.8 Hz, 1H *cis*), 4.35 (d, *J* = 7.8 Hz, 1H *cis*), 4.28 (d, *J* = 4.3 Hz, 1H *trans*), 2.43 (s, 6H, *cis* + *trans*), 2.40 (s, 3H *trans*), 2.36 (s, 3H *cis*). **^{13}C NMR** (126 MHz, $CDCl_3$) (*cis/trans* mixture) δ 189.9, 188.5, 145.3, 145.2, 145.0, 144.4, 136.8, 134.6, 133.7, 133.3, 133.1, 131.4, 131.4, 130.0, 129.6, 129.6, 129.5, 129.3, 129.2, 129.0, 128.7, 128.6, 128.6, 128.4, 128.2, 127.8, 127.7, 127.5, 50.3, 48.4, 47.6, 46.6, 21.9, 21.9, 21.8, 21.7. These data are in agreement with those reported in literature.[51]

(4-Methoxyphenyl)(3-phenyl-1-tosylaziridin-2-yl)methanone (1j)

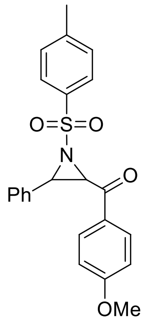

Following GP1, **1j** was prepared starting from 1-(4-methoxyphenyl)-3-phenylprop-2-en-1-one (5.8 mmol) and *p*-toluenesulfonamide. It was obtained as a 1:1.6 *cis/trans* mixture and isolated as white foamy solid (1.867 g, 79% yield). **^{1}H NMR** (500 MHz, $CDCl_3$) (*cis/ trans* mixture) δ 8.05 (d, *J* = 8.9 Hz, 2H *trans*), 7.97 (d, *J* = 8.3 Hz, 2H *cis*), 7.87 (d, *J* = 9.0 Hz, 2H *cis*), 7.72 (d, *J* = 8.3 Hz, 2H *trans*), 7.36 – 7.31 (m, 8H, *cis* + *trans*), 7.24 – 7.20 (m, 4H, *cis* + *trans*), 7.17 – 7.14 (m, 2H, *cis* + *trans*), 6.95 (d, *J* = 8.9 Hz, 2H, *trans*), 6.85 (d, *J* = 8.9 Hz, 2H *cis*), 4.50 (d, *J* = 4.2 Hz, 1H *trans*), 4.36 (d, *J* = 7.7 Hz, 1H *cis*), 4.32 (d, *J* = 7.8 Hz, 1H *cis*), 4.26 (d, *J* = 4.2 Hz, 1H *trans*), 3.88 (s, 3H *trans*), 3.82 (s, 3H *cis*), 2.43 (s, 3H *cis*), 2.39 (s, 3H *trans*). **^{13}C NMR** (126 MHz, $CDCl_3$) (*cis/ trans* mixture) *δ* 188.6, 187.4, 164.5, 164.2, 145.2, 144.4, 136.8, 134.6, 133., 131.5, 130.9, 130.0, 129.6, 129.3, 128.9, 128.9, 128.7, 128.5, 128.4, 128.2, 127.8, 127.7, 127.4, 114.2, 114.0, 55.7, 55.6, 50.2, 48.3, 47.5, 46.4, 21.8, 21.7. These data are in agreement with those reported in literature.[52]

(3-Methoxyphenyl)(3-phenyl-1-tosylaziridin-2-yl)methanone (1k)

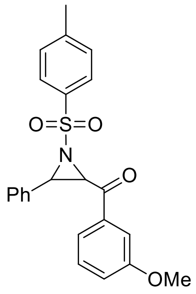

Following GP1, **1k** was prepared starting from 1-(3-methoxyphenyl)-3-phenylprop-2-en-1-one and *p*-toluenesulfonamide. It was obtained as a 3.6:1 *cis/trans* mixture and isolated as white foamy solid (1.465 g, 62% yield). **^{1}H NMR** (400 MHz, $CDCl_3$) (*cis/trans* mixture) δ 7.97 (d, *J* = 8.3 Hz, 2H *cis*), 7.72 (d, *J* = 8.3 Hz, 2H *trans*), 7.63 (ddd, *J* = 7.7, 1.6, 1.0 Hz, 1H *trans*), 7.56 (dd, *J* = 2.7, 1.6 Hz, 1H *trans*), 7.49 (ddd, *J* = 7.7, 1.6, 1.0 Hz, 1H *cis*), 7.39 – 7.29 (m, 9H, *cis* + *trans*), 7.24 – 7.15 (m, 9H, *cis* + *trans*), 7.07 (ddd, *J* = 8.3, 2.6, 1.0 Hz, 1H *cis*), 4.52 (d, *J* = 4.2 Hz, 1H *trans*), 4.39 (d, *J* = 7.7 Hz, 1H *cis*), 4.34 (d, *J* = 7.7 Hz, 1H *cis*), 4.27 (d, *J* = 4.2 Hz, 1H *trans*), 3.84 (s, 3H *trans*), 3.78 (s, 3H *cis*), 2.44 (s, 3H *cis*), 2.40 (s, 3H *trans*). **^{13}C NMR** (100 MHz, $CDCl_3$) (*cis/ trans* mixture) δ 190.3, 188.9, 160.1, 159.9, 145.3, 137.4, 137.1, 136.8, 134.5, 133.1, 131.3, 130.0, 129.9, 129.8, 129.7, 129.6, 129.0, 128.8, 128.7, 128.7, 128.5, 128.2, 127.9, 127.7, 127.5, 121.8, 121.2, 120.7, 112.8, 112.4, 55.6, 55.6, 50.5, 48.3, 47.6, 46.7, 21.8, 21.7. **HRMS** (ESI) m/z calculated for ($C_{23}H_{21}NNaO_4S$) 430.1078 $[M+Na]^+$; found 430.1083.

(2-Methoxyphenyl)(3-phenyl-1-tosylaziridin-2-yl)methanone (1l)

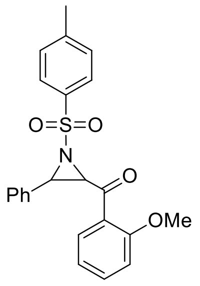

Following GP1, **1l** was prepared starting from 1-(2-methoxyphenyl)-3-phenylprop-2-en-1-one and *p*-toluenesulfonamide. It was obtained as a 3:1 *cis/trans* mixture and isolated as a white solid (1.985 g, 84% yield). **^{1}H NMR** (400 MHz, $CDCl_3$) (*cis/trans* mixture) δ 7.96 (d, *J* = 8.3 Hz, 2H *cis*), 7.84 (dd, *J* = 7.8, 1.8 Hz, 1H *trans*), 7.70 (d, *J* = 8.3 Hz, 2H *trans*), 7.53 (tdd, *J* = 8.0, 1.8, 0.6 Hz, 1H *trans*), 7.48 – 7.41 (m, 2H *cis*), 7.36 – 7.31 (m, 8H, *cis* + *trans*), 7.24 – 7.16 (m, 6H, *cis* + *trans*), 7.05 (t, *J* = 7.5 Hz, 1H *trans*), 6.96 – 6.85 (m, 3H, *cis* + *trans*), 4.59 (d, *J* = 4.4 Hz,1H *trans*), 4.56 (d, *J* = 7.9 Hz, 1H *cis*), 4.34 – 4.28 (m, 2H, *cis* + *trans*), 3.97 (s, 3H *cis*), 3.67 (s, 3H *trans*), 2.43 (s, 3H *cis*),

2.39 (s, 3H *trans*). **^{13}C NMR** (100 MHz, $CDCl_3$) (*cis/trans* mixture) δ 192.1, 189.8, 159.4, 159.2, 144.9, 144.0, 137.5, 135.2, 135.0, 134.9, 133.1, 131.8, 131.4, 130.9, 129.9, 129.5, 128.9, 128.5, 128.3, 128.2, 128.2, 128.1, 127.7, 127.7, 126.5, 126.3, 121.1, 120.9, 111.7, 111.5, 55.8, 55.7, 53.5, 51.8, 49.4, 47.2, 27.0, 21.8, 21.7. **HRMS** (ESI) m/z calculated for ($C_{23}H_{21}NNaO_4S$) 430.1076 $[M+Na]^+$; found 430.1083.

Naphthalen-2-yl(3-phenyl-1-tosylaziridin-2-yl)methanone (1m)

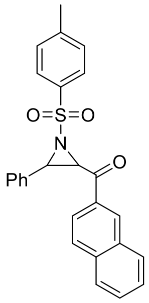

Following GP1, **1m** was prepared starting from (*E*)-1-(naphthalen-2-yl)-3-phenylprop-2-en-1-one and *p*-toluenesulfonamide. A *cis/trans* mixture was obtained and isolated as white foamy solid (1.170 g, 47% yield, *cis/trans* 7:1 *dr*). **^{1}H NMR** (400 MHz, $CDCl_3$) (*cis/trans dr* 7:1) δ 8.51 (s, 1H, *cis*), 8.49 (s, 1H, *trans*), 8.10 (dd, *J* = 8.6, 1.8 Hz, 1H, *trans*), 8.01 (d, *J* = 8.4 Hz, 2H, *cis*), 7.96 – 7.90 (m, 1H, *cis*), 7.91 – 7.86 (m, 3H, *trans*), 7.87 – 7.77 (m, 3H, *cis*), 7.71 – 7.65 (m, 2H, *trans*), 7.64 – 7.51 (m, 2H, *cis,* 2H *trans*), 7.53 – 7.45 (m, 2H, *trans*), 7.45 – 7.39 (m, 2H, *trans*), 7.39 – 7.33 (m, 2H, *cis*), 7.33 – 7.28 (m, 1H, *trans*), 7.25 – 7.20 (m, 2H, *cis*), 7.17 – 7.11 (m, 3H, *cis*), 7.09 – 7.03 (m, 2H *trans*), 4.62 (d, *J* = 4.3 Hz, 1H, *trans*), 4.55 (d, *J* = 7.8 Hz, 1H, *cis*), 4.43 (d, *J* = 7.7 Hz, 1H, *cis*), 4.39 (d, *J* = 4.3 Hz, 1H, *trans*), 2.44 (s, 3H, *cis*), 2.32 (s, 3H, *trans*). **^{13}C NMR** (101 MHz, $CDCl_3$) δ 189.0, 145.3, 136.0, 134.6, 133.2, 132.4, 131.4, 130.9, 130.1, 129.9, 129.1, 128.8, 128.6, 128.5, 128.2, 128.0, 127.5, 127.1, 123.7, 48.4, 46.7, 21.9. **HRMS** (ESI) m/z calculated for ($C_{26}H_{21}NO_3SNa$) 450.1134 $[M+Na]^+$; found 450.1136. These data are in agreement with those reported in literature.[53]

(4-Chlorophenyl)(3-phenyl-1-tosylaziridin-2-yl)methanone (1n)

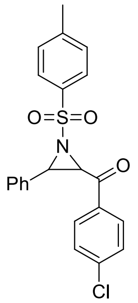

Following GP1, **1n** was prepared starting from 1-(4-chlorophenyl)-3-phenylprop-2-en-1-one and *p*-toluenesulfonamide. The *cis* isomer was isolated as white foamy solid (2.102 g, 88% yield). **^{1}H NMR** (500 MHz, $CDCl_3$) (*cis* stereoisomer) δ 7.97 (d, *J* = 8.3 Hz, 2H), 7.80 (d, *J* = 8.6 Hz, 2H), 7.36 (d, *J* = 8.6 Hz, 4H), 7.21 – 7.14 (m, 5H), 4.35 (d, *J* = 7.7 Hz, 1H), 4.33 (d, *J* = 7.7 Hz, 1H), 2.44 (s, 3H). **^{13}C NMR** (126 MHz, $CDCl_3$) (*cis* stereoisomer) δ 188.3, 145.4, 140.5, 134.4, 134.1, 131.1, 130.1, 129.9, 129.2, 128.7, 128.5, 128.2, 127.3, 48.1, 46.6, 21.8. These data are in agreement with those reported in literature.[52]

(4-Bromophenyl)(3-phenyl-1-tosylaziridin-2-yl)methanone (1o)

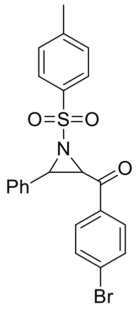

Following GP1, **1o** was prepared starting from 1-(4-bromophenyl)-3-phenylprop-2-en-1-one and *p*-toluenesulfonamide. The *cis* isomer was isolated as a white foamy solid (2.329 g, 88% yield). **^{1}H NMR** (500 MHz, $CDCl_3$) (*cis* stereoisomer) δ 7.96 (d, *J* = 8.3 Hz, 2H), 7.74 – 7.70 (m, 2H), 7.55 – 7.50 (m, 2H), 7.36 (d, *J* = 7.9 Hz, 2H), 7.19 – 7.15 (m, 5H), 4.34 (d, *J* = 7.7 Hz, 1H), 4.32 (d, *J* = 7.7 Hz, 1H), 2.44 (s, 3H). **^{13}C NMR** (126 MHz, $CDCl_3$) (*cis* stereoisomer) δ 188.6, 145.4, 134.5, 134.4, 132.2, 131.8, 131.1, 130.9, 130.8, 130.1, 130.0, 129.7, 129.4, 128.9, 128.8, 128.5, 128.2, 127.7, 127.3, 48.0, 46.6, 21.9. These data are in agreement with those reported in literature.[52]

(4-Fluoroophenyl)(3-phenyl-1-tosylaziridin-2-yl)methanone (1p)

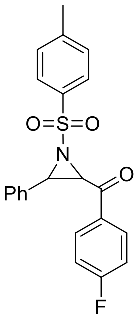

Following GP1, **1p** was prepared starting from 1-(4-fluorophenyl)-3-phenylprop-2-en-1-one and *p*-toluenesulfonamide. The *cis* isomer was isolated as a white foamy solid (2.064 g, 90% yield). **^{1}H NMR** (500 MHz, $CDCl_3$) (*cis* stereoisomer) δ 7.97 (d, *J* = 8.3 Hz, 2H), 7.94 – 7.87 (m, 2H), 7.36 (d, *J* = 7.8 Hz, 2H), 7.21 – 7.15 (m, 5H), 7.08 – 7.03 (m, 2H), 4.35 (d, *J* = 7.7 Hz, 1H), 4.33 (d, *J* = 7.7 Hz, 1H), 2.44 (s, 3H). **^{1}H{^{19}F} NMR** (500 MHz, $CDCl_3$) (*cis* stereoisomer) δ 7.97 (d, *J* = 8.3 Hz, 2H), 7.90 (d, *J* = 8.9 Hz, 2H), 7.38 – 7.34 (m, 2H), 7.20 – 7.14 (m, 5H), 7.06 (d, *J* = 8.9 Hz, 2H), 4.35 (d, *J* = 7.7 Hz, 1H), 4.32 (d, *J* = 7.8 Hz, 1H), 2.44 (s, 3H). **^{13}C NMR** (126 MHz, $CDCl_3$) (*cis* stereoisomer) δ 187.8, 167.2, 165.2, 145.4, 134.4, 131.3, 131.3, 131.2, 130.1, 128.7, 128.5, 128.2, 127.4, 116.1, 116.0, 48.1, 46.5, 21.8. **^{19}F NMR**

(471 MHz, $CDCl_3$) (*cis* stereoisomer) δ (-103.08) – (-103.22) (m). **^{19}F{^{1}H} NMR** (471 MHz, $CDCl_3$) (*cis* stereoisomer) δ (-103.15) (s). These data are in agreement with those reported in literature.[52]

(3-Fluoroophenyl)(3-phenyl-1-tosylaziridin-2-yl)methanone (1q)

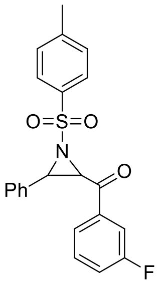

Following GP1, **1q** was prepared starting from 1-(3-fluorophenyl)-3-phenylprop-2-en-1-one and *p*-toluenesulfonamide. It was obtained as a >20:1 *cis/trans* mixture, and isolated as white foamy solid (1.858 g, 81% yield). **^{1}H{^{19}F} NMR** (400 MHz, $CDCl_3$) (*cis* stereoisomer) δ 7.97 (d, *J* = 8.4 Hz, 2H), 7.67 (ddd, *J* = 7.8, 1.6, 1.0 Hz, 1H), 7.49 (dd, *J* = 2.6, 1.5 Hz, 1H), 7.40 – 7.33 (m, 3H), 7.24 – 7.15 (m, 6H), 4.35 (s, 2H), 2.44 (s, 3H). **^{13}C NMR** (100 MHz, $CDCl_3$) (*cis* stereoisomer) δ 188.3, 188.3, 164.2, 161.6, 145.4, 137.8, 137.7, 134.4, 131.1, 130.6, 130.5, 130.1, 129.7, 128.8, 128.8, 128.5, 128.2, 127.9, 127.6, 127.4, 124.4, 124.3, 121.2, 120.9, 115.3, 115.1, 48.2, 46.7, 21.9. **^{19}F{^{1}H} NMR** (*cis* stereoisomer) (376 MHz, $CDCl_3$) δ -111.19 (s). **HRMS** (ESI) m/z calculated for ($C_{22}H_{18}FNO_3S$) 418.0884 $[M+Na]^+$; found 418.0885.

(2-Fluorophenyl)(3-phenyl-1-tosylaziridin-2-yl)methanone (1r)

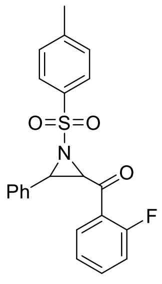

Following GP1, **1r** was prepared starting from 1-(2-fluorophenyl)-3-phenylprop-2-en-1-one and *p*-toluenesulfonamide. It was obtained as a 2:1 *cis/trans* mixture and isolated as a white solid (1.949 g, 85% yield). **^{1}H NMR** (400 MHz, $CDCl_3$) (*cis/trans* mixture) δ 8.03 – 7.93 (m, 3H, *cis* + *trans*), 7.74 (d, *J* = 8.3 Hz, 2H *trans*), 7.64 – 7.55 (m, 2H, *cis* + *trans*), 7.49 (dddd, *J* = 8.4, 7.1, 5.1, 1.9 Hz, 1H, *cis*), 7.38 – 7.06 (m, 18H, *cis* + *trans*), 4.49 (d, *J* = 4.2 Hz, 1H *trans*), 4.45 (dd, *J* = 7.8, 4.4 Hz, 1H, *cis*), 4.42 – 4.35 (m, 2H, *cis* + *trans*), 2.44 (s, 3H, *cis*), 2.40 (s, 3H, *trans*). **^{1}H{^{19}F} NMR** (400 MHz, $CDCl_3$) (*cis/trans* mixture) δ 7.97 – 7.94 (m, 3H, *cis* + *trans*), 7.74 (d, *J* = 8.4 Hz , 2H *trans*), 7.63 – 7.56 (m, 2H, *cis* + *trans*), 7.48 (ddd, *J* = 8.2, 7.3, 1.8 Hz, 1H *cis*), 7.41 – 7.10 (m, 18H, *cis* + *trans*), 4.49 (d, *J* = 4.2 Hz, 1H *trans*), 4.45 (d, *J* = 7.8 Hz, 1H *cis*), 4.40 (d, *J* = 7.8 Hz, 1H *cis*), 4.36 (d, *J* = 4.2 Hz, 1H *trans*), 2.44 (s, 3H *cis*), 2.40 (s, 3H *trans*). **^{13}C NMR** (100 MHz, $CDCl_3$) (*cis/trans* mixture) δ 189.2, 189.2, 187.1, 187.0, 163.6, 163.4, 161.1, 160.9, 145.2, 144.3, 137.1, 136.0, 135.9, 135.7, 135.6, 134.7, 133.0, 131.4, 131.4, 131.3, 131.2, 131.1, 130.8, 130.7, 130.0, 129.6, 129.0, 128.7, 128.6, 128.4, 128.2, 127.8, 127.8, 127.7, 127.6, 125.0, 124.9, 124.9, 124.8, 124.7, 124.3, 124.2, 117.0, 116.8, 116.7, 116.6, 53.0, 52.9, 51.2, 51.1, 48.7, 48.7, 47.0, 47.0, 21.8, 21.7. **^{19}F NMR** (376 MHz, $CDCl_3$) (*cis/trans* mixture) δ (-108.57) – (-108.83) (m, *cis* + *trans*). **^{19}F{^{1}H} NMR** (376 MHz, $CDCl_3$) (*cis/trans* mixture) δ -108.66 (s, *trans*), -108.75 (s, *cis*). **HRMS** (ESI) m/z calculated for ($C_{22}H_{18}FNNaO_3S$) 418.0883 $[M+Na]^+$; found 418.0884.

(3-Phenyl-1-tosylaziridin-2-yl)(pyridin-2-yl)methanone (1s)

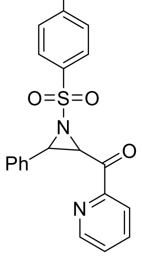

Adapting GP1 on 11.6 mmol scale, **1s** was prepared starting from (*E*)-3-phenyl-1-(pyridin-2-yl)prop-2-en-1-one (2.430 g, 11.6 mmol, 1 equiv.) and *p*-toluenesulfonamide. It was obtained as a *cis* isomer and isolated as a blue foamy solid (0.527 g, 12% yield, *cis* stereoisomer). **^{1}H NMR** (400 MHz, $CDCl_3$) (*cis* stereoisomer) δ 8.61 (d, *J* = 4.8 Hz, 1H), 7.90 (d, *J* = 8.4 Hz, 2H), 7.72 – 7.56 (m, 2H), 7.41 – 7.33 (m, 1H), 7.26 (d, *J* = 8.5 Hz, 2H), 7.21 – 7.15 (m, 2H), 7.12 – 7.04 (m, 3H), 5.11 (d, *J* = 7.9 Hz, 1H), 4.38 (d, *J* = 7.9 Hz, 1H), 2.35 (s, 3H). **^{13}C NMR** (101 MHz, $CDCl_3$) (*cis* stereoisomer) δ 189.6, 152.6, 149.3, 145.0, 137.0, 134.7, 131.6, 129.9, 128.4, 128.2, 128.2, 127.8, 127.8, 122.2, 47.6, 47.5, 21.8. **HRMS** (ESI) m/z calculated for ($C_{21}H_{18}N_{23}SNa$) 401.0941 $[M+Na]^+$; found 401.0930.

(3-Phenyl-1-tosylaziridin-2-yl)(thiophen-2-yl)methanone (1t)

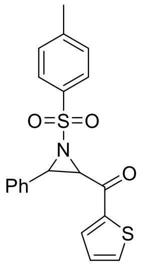

Adapting GP1 on 11.6 mmol scale, **1t** was prepared starting from (*E*)-3-phenyl-1-(thiophen-2-yl)prop-2-en-1-one (2.500 g, 11.6 mmol, 1 equiv.) and *p*-toluenesulfonamide. It was obtained as a *cis* isomer and isolated as a yellow solid (1.880 g, 42% yield, *cis* stereoisomer). **^{1}H NMR** (400 MHz, $CDCl_3$) (*cis* stereoisomer) δ 7.89 (d, *J* = 8.4 Hz, 2H), 7.86 (d, *J* = 3.9 Hz, 1H), 7.54 (d, *J* = 6.1 Hz, 1H), 7.29 (d, *J* = 8.0 Hz, 2H), 7.17 – 7.06 (m, 5H), 7.05 – 6.94 (m, 1H), 4.20 – 4.16 (two overlapped doublets, d, *J* = 8.2, 2H), 2.37 (s, 3H). **^{13}C NMR** (101 MHz, $CDCl_3$) (*cis* stereoisomer) δ 182.2, 145.4, 142.2, 135.2, 134.2, 134.0, 131.2, 130.1, 128.6, 128.4, 128.4, 128.3, 127.5, 47.9, 46.5, 21.8. **HRMS** (ESI) m/z calculated for ($C_{20}H_{17}NO_3S_2Na$) 406.0542 $[M+Na]^+$; found 406.0544.

1-(3-Phenyl-1-tosylaziridin-2-yl)ethan-1-one (1u)

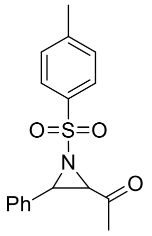

Following GP1, **1u** was prepared starting from 4-phenylbut-3-en-2-one and *p*-toluenesulfonamide. The *cis* isomer was isolated as a white foamy solid (402.0 mg, 22% yield). **^{1}H NMR** (400 MHz, $CDCl_3$) (*cis* diastereoisomer) δ 7.95 (d, *J* = 8.3 Hz, 2H), 7.43 – 7.39 (m, 2H), 7.32 – 7.23 (m, 5H), 4.18 (d, *J* = 7.9 Hz, 1H), 3.64 (d, *J* = 7.9 Hz, 1H), 2.49 (s, 3H), 1.76 (s, 3H). **^{13}C NMR** (100 MHz, $CDCl_3$) (*cis* diastereoisomer) δ 200.9, 145.5, 134.0, 131.5, 130.2, 128.8, 128.8, 128.4, 127.5, 49.2, 45.9, 28.7, 21.9. **HRMS** (ESI) m/z calculated for ($C_{17}H_{17}NNaO_3S$) 338.0821 $[M+Na]^+$; found 338.0822.

2,2-Dimethyl-1-(3-phenyl-1-tosylaziridin-2-yl)propan-1-one (1v)

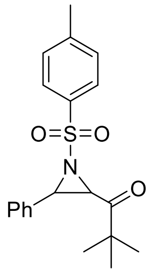

Following GP1, **1v** was prepared starting from 4,4-dimethyl-1-phenylpent-1-en-3-one and *p*-toluenesulfonamide. It was obtained as a 1:3.5 *cis/trans* mixture and the enriched product in the *trans* isomer was isolated as white foamy solid (431.0 mg, 27% yield, 1:5.2 d.r.). **^{1}H NMR** (400 MHz, $CDCl_3$) (*trans* diastereoisomer) δ 7.75 (d, *J* = 8.3 Hz, 2H), 7.38 – 7.29 (m, 5H), 7.30 – 7.24 (m, 2H), 4.32 (d, *J* = 4.1 Hz, 1H), 4.01 (d, *J* = 4.2 Hz, 1H), 2.43 (s, 3H), 1.30 (s, 9H). **^{13}C NMR** (100 MHz, $CDCl_3$) (*trans* diastereoisomer) δ 205.8, 144.1, 137.6, 132.6, 129.5, 128.9, 128.6, 127.7, 127.4, 77.3, 77.0, 76.7, 49.7, 47.2, 44.7, 25.8, 25.7, 21.6. These data are in agreement with those reported in literature.[50]

Phenyl(3-(*p*-tolyl)-1-tosylaziridin-2-yl)methanone (1w)

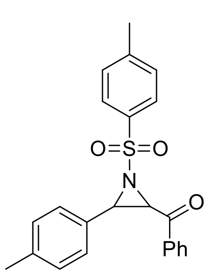

Following GP1, **1w** was prepared starting from 1-phenyl-3-(*p*-tolyl)prop-2-en-1-one and *p*-toluenesulfonamide. It was obtained as a 1:1 *cis/trans* mixture and isolated as white foamy solid (1.884 g, 83% yield). **^{1}H NMR** (500 MHz, $CDCl_3$) (*cis/trans* mixture) δ 8.04 (dd, *J* = 8.4, 1.3 Hz, 2H *trans*), 7.96 (d, *J* = 8.3 Hz, 2H *cis*), 7.86 (dd, *J* = 8.4, 1.3 Hz, 2H *trans*), 7.72 (d, *J* = 8.3 Hz, 2H *cis*), 7.63 – 7.59 (m, 1H *cis*), 7.54 – 7.46 (m, 3H, *cis* + *trans*), 7.40 – 7.36 (m, 2H, *cis* + *trans*), 7.35 – 7.33 (m, 2H, *cis* + *trans*), 7.26 – 7.21 (m, 4H, *cis* + *trans*), 7.13 (dd, *J* = 16.9, 7.9 Hz, 4H, *cis* + *trans*), 6.96 (d, *J* = 7.7 Hz, 2H *cis*), 4.46 (d, *J* = 4.2 Hz, 1H *trans*), 4.39 (d, *J* = 7.7 Hz, 1H, *cis*), 4.34 – 4.31 (m, 2H, *cis* + *trans*), 2.42 (s, 3H *cis*), 2.39 (s, 3H *trans*), 2.34 (s, 3H *trans*), 2.20 (s, 3H *cis*). **^{13}C NMR** (126 MHz, $CDCl_3$) (*cis*/ *trans* mixture) δ 190.6, 189.2, 145.2, 144.4, 139.0, 138.5, 136.9, 136.1, 135.8, 134.6, 134.2, 133.9, 130.0, 129.8, 129.6, 129.4, 129.1, 129.0, 128.9, 128.8, 128.5, 128.3, 128.2, 127.8, 127.7, 127.3, 49.9, 48.3, 48.0, 46.7, 21.8, 21.7, 21.4, 21.2. These data are in agreement with those reported in literature.[52]

Phenyl(3-(*o*-tolyl)-1-tosylaziridin-2-yl)methanone (1x)

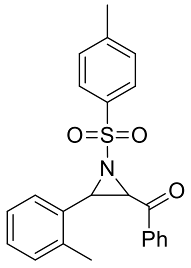

Following GP1, **1x** was prepared starting from 1-phenyl-3-(*o*-tolyl)prop-2-en-1-one and *p*-toluenesulfonamide. The *cis* isomer was isolated as a white foamy solid (1.703 g, 75% yield). **^{1}H NMR** (500 MHz, $CDCl_3$) (*cis* stereoisomer) δ 7.99 (d, *J* = 8.3 Hz, 2H), 7.86 (dd, *J* = 8.4, 1.3 Hz, 2H), 7.56 – 7.50 (m, 1H), 7.41 – 7.34 (m, 4H), 7.16 (dd, *J* = 7.7, 1.5 Hz, 1H), 7.08 (td, *J* = 7.4, 1.5 Hz, 1H), 7.02 – 6.96 (m, 2H), 4.53 (d, *J* = 7.6 Hz, 1H), 4.36 (d, *J* = 7.6 Hz, 1H), 2.44 (s, 3H), 2.31 (s, 3H). **^{13}C NMR** (126 MHz, $CDCl_3$) (*cis* stereoisomer) δ 189.2, 145.3, 136.3, 135.9, 134.5, 133.8, 130.1, 129.9, 129.3, 128.7, 128.5, 128.4, 128.2, 127.5, 125.8, 46.8, 45.7, 21.8, 19.2. These data are in agreement with those reported in literature.[51]

(3-(3-Methoxyphenyl)-1-tosylaziridin-2-yl)(phenyl)methanone (1y)

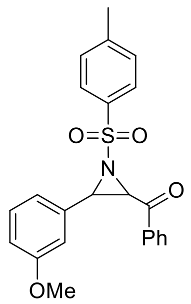

Following GP1, **1y** was prepared starting from 3-(3-methoxyphenyl)-1-phenylprop-2-en-1-one and *p*-toluenesulfonyl amine. It was obtained as a 2.4:1 *cis/trans* mixture and isolated as white foamy solid (1.938 g, 82% yield). **^{1}H NMR (**500 MHz, $CDCl_3$) (*cis*/*trans* mixture) δ 8.05 (dd, *J* = 8.4, 1.3 Hz, 2H *trans*), 7.97 (d, *J* = 8.3 Hz, 2H *cis*), 7.88 – 7.84 (m, 2H *cis*), 7.74 (d, *J* = 8.3 Hz, 2H *trans*), 7.64 – 7.60 (m, 1H *trans*), 7.55 – 7.45 (m, 4H, *cis* + *trans*), 7.42 – 7.33 (m, 4H, *cis* + *trans*), 7.27 – 7.22 (m, 2H, *cis* + *trans*), 7.07 (t, *J* = 7.9 Hz, 1H *cis*), 6.94 (dt, *J* = 7.8, 1.5 Hz, 1H *trans*), 6.86 (ddd, *J* = 8.3, 2.6, 0.9 Hz, 1H *trans*), 6.84 – 6.80 (m, 2H, *cis* + *trans*), 6.74 – 6.71 (m, 1H *cis*), 6.69 (ddd, *J* = 8.2, 2.6, 1.0 Hz, 1H *cis*), 4.51 (d, *J* = 4.2 Hz, 1H *trans*), 4.39 (d, *J* = 7.7 Hz, 1H *cis*), 4.32 (d, *J* = 7.7 Hz, 1H *cis*), 4.23 (d, *J* = 4.2 Hz, 1H *trans*), 3.75 (s, 3H *trans*), 3.63 (s, 3H *cis*), 2.43 (s, 3H *cis*), 2.40 (s, 3H *trans*). **^{13}C NMR** (126 MHz, $CDCl_3$) (*cis*/*trans* mixture) δ 190.3, 189.1, 159.9, 159.5, 145.3, 144.5, 136.8, 136.1, 135.8, 134.8, 134.5, 134.2, 133.9, 132.8, 130.0, 129.9, 129.6, 129.5, 129.1, 128.9, 128.8, 128.5, 128.2, 127.9, 119.9, 119.7, 114.7, 114.7, 112.8, 112.5, 55.4, 55.2, 50.8, 48.3, 47.3, 46.6, 27.0, 21.8. **HRMS** (ESI) m/z calculated for ($C_{23}H_{21}NNaO_4S$) 430.1083 $[M+Na]^+$; found 430.1088.

(3-(4-Fluorophenyl)-1-tosylaziridin-2-yl)(phenyl)methanone (1z)

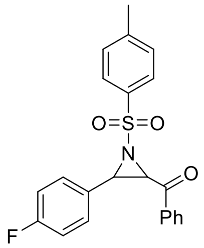

Following GP1, **1z** was prepared starting from 3-(4-fluorophenyl)-1-phenylprop-2-en-1-one and *p*-toluenesulfonamide. It was obtained as a 1.8:1 *cis/trans* mixture and isolated as white foamy solid (1.858 g, 81% yield). **^{1}H NMR** (500 MHz, $CDCl_3$) (*cis/trans* mixture) δ 8.04 (d, *J* = 7.0 Hz, 2H *trans*), 7.96 (d, *J* = 8.3 Hz, 2H *cis*), 7.85 (dd, *J* = 8.3, 1.3 Hz, 2H *cis*), 7.71 (d, *J* = 8.3 Hz, 2H *trans*), 7.62 (t, *J* = 7.4 Hz, 1H *trans*), 7.54 (t, *J* = 7.5 Hz, 1H *cis*), 7.50 – 7.45 (m, 2H *trans*), 7.42 – 7.31 (m, 6H, *cis* + *trans*), 7.25 – 7.19 (m, 4H, *cis* + *trans*), 7.03 (d, *J* = 8.8 Hz, 2H *trans*), 6.85 (d, *J* = 8.8 Hz, 2H *cis*), 4.49 (d, *J* = 4.2 Hz, 1H *trans*), 4.40 (d, *J* = 7.7 Hz, 1H *cis*), 4.34 (d, *J* = 7.7 Hz, 1H *cis*), 4.28 (d, *J* = 4.2 Hz, 1H *trans*), 2.44 (s, 3H *cis*), 2.40 (s, 3H *trans*). **^{13}C NMR** (126 MHz, $CDCl_3$) (*cis*/*trans* mixture) δ 190.3, 188.9, 164.1, 163.8, 162.1, 161.9, 145.4, 144.7, 136.6, 136.0, 135.7, 134.4, 134.3, 134.1, 130.1, 129.7, 129.6, 129.6, 129.6, 129.3, 129.2, 129.1, 129.0, 128.9, 128.8, 128.7, 128.4, 128.2, 127.8, 127.1, 127.1, 115.9, 115.7, 115.6, 115.4, 50.1, 48.3, 46.9, 45.9, 21.8, 21.7. **^{19}F{^{1}H} NMR** (471 MHz, $CDCl_3$) (*cis*/ *trans* mixture) δ (-112.25) (s, *trans*), (-112.81) (s, *cis*). **^{19}F NMR** (471 MHz, $CDCl_3$) (*cis*/ *trans* mixture) δ (-112.21) – (-112.31) (m, *trans*), (-112.75) – (-112.89) (m, *cis*). These data are in agreement with those reported in literature.[52]

(3-(4-Chlorophenyl)-1-tosylaziridin-2-yl)(phenyl)methanone (1za)

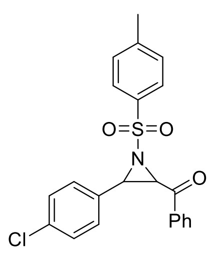

Following GP1, **1za** was prepared starting from 3-(4-chlorophenyl)-1-phenylprop-2-en-1-one and *p*-toluenesulfonamide. It was obtained as a 3.9:1 *cis/trans* mixture and isolated as white foamy solid (1.887 g, 79% yield). **^{1}H NMR** (500 MHz, $CDCl_3$) (*cis/trans* mixture) δ 8.06 – 8.01 (m, 2H *trans*), 7.96 (d, *J* = 8.3 Hz, 2H *cis*), 7.87 – 7.83 (m, 2H *cis*), 7.71 (d, *J* = 8.3 Hz, 2H *trans*), 7.65 – 7.60 (m, 1H *trans*), 7.57 – 7.52 (m, 1H *cis*), 7.51 – 7.46 (m, 2H *trans*), 7.44 – 7.37 (m, 2H *cis*), 7.36 (d, *J* = 8.0 Hz, 2H *cis*), 7.32 – 7.27 (m, 4H *trans*), 7.24 (d, *J* = 7.9 Hz, 2H *trans*), 7.21 – 7.11 (m,

4H *cis*), 4.49 (d, *J* = 4.2 Hz, 1H *trans*), 4.42 (d, *J* = 7.7 Hz, 1H *cis*), 4.32 (d, *J* = 7.7 Hz, 1H *cis*), 4.25 (d, *J* = 4.2 Hz, 1H *trans*), 2.44 (s, 3H *cis*), 2.40 (s, 3H *trans*). **^{13}C NMR** (126 MHz, $CDCl_3$) (*cis/trans* mixture) δ 190.2, 188.8, 145.4, 144.7, 136.6, 136.0, 135.6, 135.0, 134.6, 134.4, 134.3, 134.1, 131.7, 130.1, 129.9, 129.7, 129.1, 129.0, 129.0, 129.0, 128.9, 128.9, 128.7, 128.5, 128.2, 127.8, 50.4, 48.2, 46.7, 45.9, 21.8, 21.8. These data are in agreement with those reported in literature.[52]

(3-(2-Chlorophenyl)-1-tosylaziridin-2-yl)(phenyl)methanone (1zb)

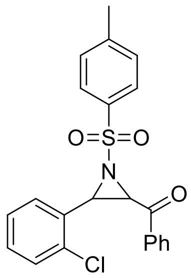

Following GP1, **1zb** was prepared starting from 3-(2-chlorophenyl)-1-phenylprop-2-en-1-one and *p*-toluenesulfonamide. It was obtained as a 1:2.4 *cis/trans* mixture and isolated as white foamy solid (1.959 g, 82% yield). **^{1}H NMR (**500 MHz, $CDCl_3$) (*cis*/*trans* mixture) δ 8.06 (dd, *J* = 8.4, 1.3 Hz, 2H *trans*), 7.98 (d, *J* = 8.3 Hz, 2H *cis*), 7.91 (dd, *J* = 8.4, 1.3 Hz, 2H *cis*), 7.74 (d, *J* = 8.3 Hz, 2H *trans*), 7.64 – 7.60 (m, 1H *trans*), 7.56 – 7.52 (m, 1H *cis*), 7.51 – 7.46 (m, 2H *trans*), 7.43 – 7.33 (m, 4H, *cis* + *trans*), 7.29 – 7.17 (m, 8H, *cis* + *trans*), 7.14 – 7.10 (m, 2H *cis*), 4.78 (d, *J* = 4.2 Hz, 1H *trans*), 4.60 (d, *J* = 7.6 Hz, 1H *cis*), 4.57 (d, *J* = 7.6 Hz, 1H *cis*), 4.20 (d, *J* = 4.2 Hz, 1H *trans*), 2.44 (s, 3H *cis*), 2.41 (s, 3H *trans*). **^{13}C NMR** (126 MHz, $CDCl_3$) (*cis/trans* mixture) δ 189.9, 188.4, 145.4, 144.7, 136.4, 136.1, 135.6, 134.9, 134.3, 134.2, 134.0, 133.6, 131.5, 130.1, 130.1, 129.8, 129.7, 129.6, 129.5, 129.2, 129.1, 129.1, 128.9, 128.7, 128.6, 128.3, 128.2, 128.0, 127.0, 126.8, 49.6, 47.2, 45.7, 45.2, 21.8, 21.8. These data are in agreement with those reported in literature.[52]

(3-Methyl-1-tosylaziridin-2-yl)(phenyl)methanone (1zd)

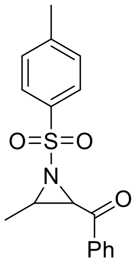

Following GP1, **1zd** was prepared starting from 1-phenylbut-2-en-1-one and *p*-toluenesulfonamide. It was obtained as a 1:2.5 *cis/trans* mixture and the *trans* isomer was isolated as white foamy solid (177.3 mg, 59% yield). **^{1}H NMR** (400 MHz, $CDCl_3$) (*trans* diastereoisomer) δ 8.00 (d, *J* = 8.5 Hz, 2H), 7.90 (d, *J* = 8.4 Hz, 2H), 7.62 (td, *J* = 7.2, 1.5 Hz, 1H), 7.49 (t, *J* = 7.8 Hz, 2H), 7.35 (d, *J* = 8.4 Hz, 2H), 7.27 (s, 1H), 4.14 (d, *J* = 7.7 Hz, 1H), 3.36 (dt, *J* = 7.9, 5.8 Hz, 1H), 2.45 (s, 3H), 1.21 (d, *J* = 5.9 Hz, 3H). **^{13}C NMR** (100 MHz, $CDCl_3$) (*trans* diastereoisomer) δ 190.6, 144.9, 135.8, 134.7, 134.1, 129.9, 128.9, 128.5, 128.0, 77.3, 77.0, 76.7, 45.7, 41.1, 21.7, 12.6. These data are in agreement with those reported in literature.[52]

2-Phenyl-1-tosyl-1a,7b-dihydro-1*H*-azirino[2,3-c]quinoline (1ze)

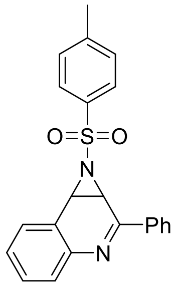

Adapting GP1 on 11.6 mmol scale, **1ze** was prepared starting from (*E*)-3-(2-aminophenyl)-1-phenylprop-2-en-1-one and *p*-toluenesulfonamide. It was obtained as *cis* isomer and isolated as a yellow solid (0.434 g, 10% yield). **^{1}H NMR** (400 MHz, $CDCl_3$) (*cis* stereoisomer) δ 7.98 – 7.92 (m, 2H), 7.88 (d, *J* = 8.4 Hz, 2H), 7.67 – 7.62 (m, 1H), 7.53 – 7.40 (m, 5H), 7.33 (d, *J* = 8.6 Hz, 2H), 7.29 (dd, *J* = 7.4, 1.3 Hz, 1H), 4.47 (d, *J* = 6.9 Hz, 1H), 4.40 (d, *J* = 6.9 Hz, 1H), 2.43 (s, 3H). **^{13}C NMR** (101 MHz, $CDCl_3$) (*cis* stereoisomer) δ 158.3, 145.3, 142.8, 137.4, 135.0, 131.3, 130.6, 130.3, 130.0, 128.9, 128.8, 128.5, 128.1, 127.8, 121.1, 44.3, 37.6, 21.8. **HRMS** (ESI) m/z calculated for ($C_{22}H_{18}N_2O_2SNa$) 397.0987 $[M+Na]^+$; found 397.0981.

Synthesis of (1-([1,1'-biphenyl]-4-ylsulfonyl)-3-phenylaziridin-2-yl)(phenyl)methanone (1h):

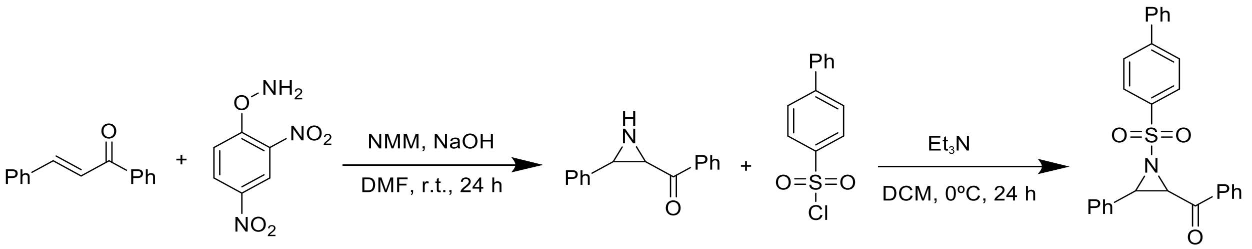

Scheme S3. *Synthesis of aziridine **1h**.*

N-Methylmorpholine (0.11 mL, 1.04 mmol, 1.04 equiv.) was added to a solution of *O*-(2,4-dinitrophenyl) hydroxylamine (303.0 mg, 1.52 mmol, 1.52 equiv.) in DMF (1 mL). The mixture was stirred for 15 minutes and then NaOH (80.0 mg, 2 mmol, 2.00 equiv.) and (*E*)-chalcone (208.3 mg, 1.00 mmol, 1 equiv.) were added. The mixture was stirred until the thin-layer chromatography (TLC) analysis indicated the complete consumption of the substrate. The reaction mixture was diluted with EtOAc and washed with brine. The organic layer was dried over anhydrous Na_2SO_4 and concentrated in vacuo. Crude aziridine was used for the further reaction without purification.
To a solution of the aziridine (1.00 mmol, 1.00 equiv.) and Et_3N (418 µL, 3.00 mmol) in DCM (3.5 mL), biphenyl-4-sulfonyl chloride (379.0 mg, 1.5 mmol, 1.50 equiv.) was added at 0 ºC, and reaction mixture was stirred for 24 h at the same temperature. Afterwards, it was quenched with NH_4Cl saturated solution and diluted with EtOAc. The phases were separated and aqueous phase was extracted with EtOAc three times. Combined organic layers were washed with brine and dried over anhydrous Na_2SO_4. After evaporation of the solvent under reduced pressure, the crude product was purified by crystallization (Cyclohexane/ EtOAc) to afford the desired product as the *cis* isomer (369.0 mg, 84% yield over two steps). **^{1}H NMR** (400 MHz, $CDCl_3$) (*cis* diastereomer) δ 8.19 (d, *J* = 8.6 Hz, 2H), 7.93 – 7.86 (m, 2H), 7.83 – 7.77 (m, 2H), 7.67 – 7.61 (m, 2H), 7.57 – 7.39 (m, 6H), 7.31 – 7.26 (m, 2H), 7.24 – 7.17 (m, 3H), 4.50 (d, *J* = 7.7 Hz, 1H), 4.46 (d, *J* = 7.7 Hz, 1H). **^{13}C NMR** (100 MHz, $CDCl_3$) (*cis* diastereomer) δ 189.0, 147.1, 139.2, 136.1, 135.8, 134.0, 131.2, 129.2, 128.9, 128.7, 128.7, 128.5, 128.0, 127.6, 127.5, 48.5, 46.7. **HRMS** (ESI) m/z calculated for ($C_{27}H_{21}NNaO_3S$) 462.1124 $[M+Na]^+$; found 462.1134.

Synthesis of phenyl(1-tosylaziridin-2-yl)methanone (1zc):

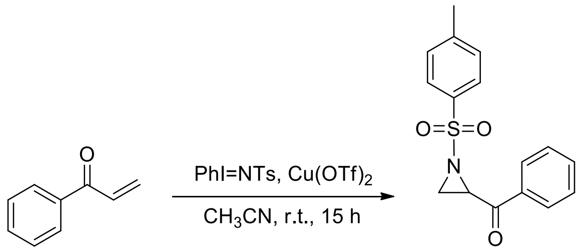

***Scheme S4**. Synthesis of aziridine **1zc**.*

PhI=NTs (5.600 g, 15 mmol, 15 equiv.) was added, under argon and at room temperature, to a solution of phenyl vinyl ketone (996 µL, 10 mmol) and $Cu(OTf)_2$ (362.0 mg, 1 mmol, 1 equiv.) in dry acetonitrile (30 mL), and reaction mixture was stirred at room temperature overnight. Afterwards, it was concentrated in vacuo. The crude was purified by flash chromatography (Cyclohexane: EtOAc / 8:2) to give pure product as a yellow solid (1.055 g, 35% yield). **^{1}H NMR** (300 MHz, $CDCl_3$) δ 8.09 – 8.01 (m, 2H), 7.88 (d, *J* = 8.4 Hz, 2H), 7.67 – 7.59 (m, 1H), 7.53 – 7.45 (m, 2H), 7.37 – 7.31 (m, 2H), 4.17 (dd, *J* = 6.9, 4.2 Hz, 1H), 2.87 (d, *J* = 7.0 Hz, 1H), 2.75 (d, *J* = 4.2 Hz, 1H), 2.44 (s, 3H). **^{13}C NMR** (126 MHz, $CDCl_3$) δ 191.5, 145.4, 135.7, 134.4, 134.3, 130.0, 129.0, 128.9, 128.3, 37.9, 32.9, 21.8. These data are in agreement with those reported in literature.[54]

4. Photocatalyzed Radical-Polar Crossover Ring-Expansion and Derivatization of 2a

General procedure for the intramolecular aziridine ring expansion (GP2)

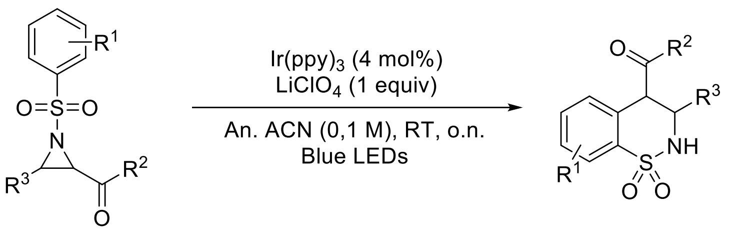

***Scheme S5**. Intramolecular aziridine ring expansion.*

The corresponding aziridine (0.1 mmol, 1 equiv.) was mixed in a 10 mL vial with $LiClO_4$ (11.0 mg, 0.1 mmol, 1 equiv.) and $Ir(ppy)_3$ (3.0 mg, 0.04 mmol, 4 mol%). Then, the vial was sealed and three cycles of vacuum-refill with Ar were performed. After adding degassed acetonitrile (1 mL), the mixture was stirred under blue LEDs irradiation overnight. Afterwards, the vial was opened, 10 mL of EtOAc were added and the mixture was washed with water three times (3 x 10 mL) and brine (10 mL). The separated organic phase was then dried over $MgSO_4$, filtered, and concentrated under vacuum. The desired product was purified by silica gel column chromatography (EtOAc/Petroleum ether 0% to 30%).

General procedure for the 1 mmol scale intramolecular ring expansion (GP3)

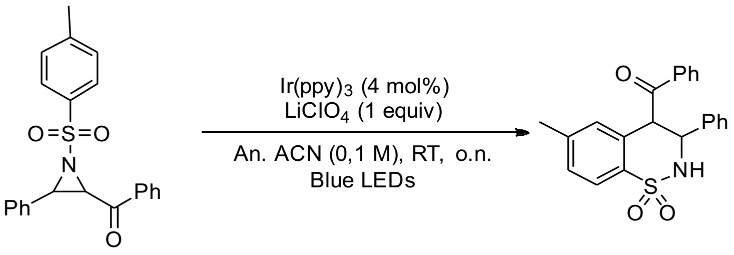

***Scheme S6**. Intramolecular aziridine ring expansion.*

The corresponding aziridine (1 mmol, 1 equiv.) was mixed in a 50 mL Schlenk tube with $LiClO_4$ (106.0 mg, 1 mmol, 1 equiv.) and $Ir(ppy)_3$ (26.0 mg, 0.4 mmol, 4 mol%). Then, the tube was sealed and three cycles of vacuum-refill with Ar were performed. After adding degassed anhydrous acetonitrile (10 mL), the mixture was stirred under blue LEDs irradiation overnight. Afterwards, the tube was opened, 100 mL of EtOAc were added and the mixture was washed with water three times (3x100 mL) and brine (100 mL). The separated organic phase was then dried over $MgSO_4$, filtered, and concentrated under vacuum. The crude was purified by silica gel column chromatography (EtOAc/Petroleum ether 0% to 30%).

(6-Methyl-1,1-dioxido-3-phenyl-3,4-dihydro-2*H*-benzo[*e*][1,2]thiazin-4-yl)(phenyl)methanone (2a)

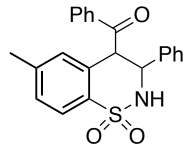

Following GP2, **2a** was prepared starting from phenyl(3-phenyl-1-tosylaziridin-2-yl)methanone (**1a**) (38.0 mg, 0.1 mmol, 1 equiv.) and it was obtained as a 1.8:1 *trans*/*cis* mixture and isolated as white amorphous solid (22.0 mg, 58% yield, 1.8:1 d.r.).

Following GP3, **2a** was prepared starting from 1.0 mmol (377.0 mg) of phenyl(3-phenyl-1-tosylaziridin-2-yl)methanone (**1a**) and it was obtained as a 1.7:1 *trans*/*cis* mixture and isolated as white amorphous solid (202.0 mg, 54% yield, 1.7:1 d.r.). **^{1}H NMR** (400 MHz, $CDCl_3$) (*cis*/*trans* mixture) δ 7.88 (d, *J* = 8.1 Hz, 1H *cis*), 7.80 (d, *J* = 8.0 Hz, 1H *trans*), 7.76 – 7.71 (m, 4H, *cis* + *trans*), 7.58 – 7.49 (m, 2H, *cis* + *trans*), 7.43 – 7.34 (m, 6H, *cis* + *trans*), 7.32 – 7.27 (m, 4H, *cis* + *trans*), 7.25 – 7.13 (m, 6H, *cis* + *trans*), 6.87 (t, *J* = 1.1 Hz, 1H *cis*), 6.80 (d, *J* = 1.6 Hz, 1H *trans*), 6.59 (d, *J* = 12.7 Hz, 1H *cis*), 5.55 (dd, *J* = 12.7, 3.6 Hz, 1H *cis*), 5.36 (d, *J* = 10.9 Hz, 1H *trans*), 5.26 (dd, *J* = 11.0, 9.2 Hz, 1H *trans*), 5.22 (d, *J* = 3.7 Hz, 1H *cis*), 5.17 (d, *J* = 9.2 Hz, 1H *trans*), 2.26 (s, 3H *trans*), 2.25 (s, 3H *cis*).

^{13}**C NMR** (126 MHz, $CDCl_3$) (*cis*/*trans* mixture) δ 200.4, 199.2, 143.4, 143.1, 138.2, 137.2, 136.9, 136.7, 135.4, 135.1, 135.0, 134.3, 134.0, 133.8, 133.5, 130.0, 129.6, 129.0, 129.0, 129.0, 128.9, 128.7, 128.7, 128.2, 127.6, 125.9, 125.6, 123.8, 115.7, 112.4, 60.3, 58.8, 51.9, 47.2, 21.8, 21.5. **HRMS** (ESI) m/z calculated for ($C_{22}H_{19}NNaO_3S$) 400.0978 $[M+Na]^+$; found 400.0978.

Crystallization: Fractions enriched in one diastereomer were obtained through further flash chromatography purification. Single crystals suitable for X-ray analysis and melting point measurement were grown by slow diffusion of *n*-hexane into the dichloromethane solution of **2a**. **Melting point** (*trans*): 205.5 ºC. **Melting point** (*cis*): 214.8 ºC.

Epimerization: To a solution of **2a** (38.1 mg, 0.1 mmol, 1 equiv.) in DMSO-d6 (1 mL), ^{t}BuOK (33.7 mg, 0.3 mmol, 3 equiv.) were added and the mixture was let stirred at room temperature. The reaction was monitor by ^{1}H NMR until reaching a 92:8 d.r (*trans*:*cis*) after 6 h (2h for a 0.02 mmol scale, see page S6 epimerization study). Afterwards, water (8 mL), followed by sat. NH_4Cl (5 mL) were added and the mixture was extracted with Et_2O (5x6 mL). The separated organic phase was then dried over $MgSO_4$, filtered, and concentrated under vacuum. The crude was purified by silica gel column chromatography (EtOAc/Pentane 0% to 30%), providing *trans*-**2a** as white solid (20.9 mg, 0.055 mmol, 55%). ^{1}**H NMR** (400 MHz, $CDCl_3$) (*trans*) δ 7.84 – 7.78 (m, 1H), 7.78 – 7.71 (m, 2H), 7.53 (t, *J* = 7.5 Hz, 1H), 7.44 – 7.34 (m, 4H), 7.28 – 7.18 (m, 4H), 6.97 – 6.89 (m, 0.5H NH), 6.80 (s, 1H), 5.35 (d, *J* = 10.3 Hz, 0.4H NH), 5.25 (d, *J* = 10.0 Hz, 1H), 5.18 (dd, *J* = 10.0, 8.3 Hz, 1H), 2.26 (s, 3H). ^{13}**C NMR** (100 MHz, $CDCl_3$) (*trans*) δ 199.1, 143.5, 138.3, 137.2, 135.5, 135.0, 134.0, 129.7, 129.2, 129.1, 129.0, 128.9, 128.7, 127.6, 123.8, 116.0, 115.8, 60.3, 51.9, 21.8. **HRMS** (ESI) m/z calculated for ($C_{22}H_{20}NNaO_3S$) 401.1056 $[M+H+Na]^+$; found 401.1036.

(1,1-Dioxido-3-phenyl-3,4-dihydro-2*H*-benzo[*e*][1,2]thiazin-4-yl)(phenyl)methanone (2b)

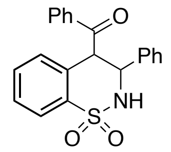

Following GP2, **2b** was prepared starting from phenyl(3-phenyl-1-(phenylsulfonyl)aziridin-2-yl)methanone (**1b**) (36.0 mg, 0.1 mmol, 1 equiv.) and it was obtained as a 1.5:1 *trans*/*cis* mixture and isolated as white amorphous solid (21.0 mg, 59% yield, 1.5:1 d.r.). ^{1}**H NMR** (500 MHz, $CDCl_3$) (*cis*/*trans* mixture) δ 8.01 (d, *J* = 7.0 Hz, 1H *cis*), 7.95 (dd, *J* = 7.6, 1.4 Hz, 1H *trans*), 7.77 – 7.73 (m, 4H, *cis* + *trans*), 7.59 – 7.50 (m, 2H, *cis* + *trans*), 7.50 – 7.26 (m, 16H, *cis* + *trans*), 7.24 – 7.16 (m, 2H, *cis* + *trans*), 7.10 (d, *J* = 7.8 Hz, 1H *cis*), 7.03 (d, *J* = 7.6 Hz, 1H *trans*), 6.67 (d, *J* = 12.7 Hz, 1H *cis*), 5.59 (dd, *J* = 12.7, 3.6 Hz, 1H *cis*), 5.42 (d, *J* = 11.1 Hz, 1H *trans*), 5.32 – 5.24 (m, 2H, *cis* + *trans*), 5.09 (d, *J* = 9.0 Hz, 1H *trans*). ^{13}**C NMR** (126 MHz, $CDCl_3$) (*cis*/*trans* mixture) δ 200.5, 199.0, 138.7, 138.6, 138.1, 137.3, 137.1, 136.8, 135.3, 134.5, 134.3, 133.6, 132.9, 132.5, 129.6, 129.5, 129.3, 129.2, 129.2, 129.2, 128.9, 128.5, 128.5, 128.4, 127.9, 126.1, 126.0, 124.0, 60.4, 59.0, 51.6, 47.4. **HRMS** (ESI) m/z calculated for ($C_{21}H_{17}NNaO_3S$) 386.0821 $[M+Na]^+$; found 386.0827.

(8-Methyl-1,1-dioxido-3-phenyl-3,4-dihydro-2*H*-benzo[*e*][1,2]thiazin-4-yl)(phenyl)methanone (2c)

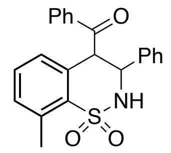

Following GP2, **2c** was prepared starting from phenyl(3-phenyl-1-(o-tolylsulfonyl)aziridin-2-yl)methanone (**1c**) (38.0 mg, 0.1 mmol, 1 equiv.) and it was obtained as a 1.7:1 *trans*/*cis* mixture and isolated as white amorphous solid (16.0 mg, 43% yield, 2.0:1 d.r.). ^{1}**H NMR** (500 MHz, $CDCl_3$) (*cis*/*trans* mixture) δ 7.74 – 7.69 (m, 4H, *cis* + *trans*), 7.57 – 7.50 (m, 2H, *cis* + *trans*), 7.41 – 7.36 (m, 4H, *cis* + *trans*), 7.34 – 7.27 (m, 4H, *cis* + *trans*), 7.25 – 7.15 (m, 10H, *cis* + *trans*), 6.91 (dd, *J* = 7.0, 1.9 Hz, 1H cis), 6.85 – 6.80 (m, 2H, *cis* + *trans*), 5.53 (dd, *J* = 12.9, 3.5 Hz 1H, *cis*), 5.45 (d, *J* = 9.6 Hz, 1H *trans*), 5.38 – 5.29 (m, 2H *trans*), 5.24 (d, *J* = 3.5 Hz, 1H *cis*), 2.80 (s, 3H *cis*), 2.74 (s, 3H *trans*). ^{13}**C NMR** (126 MHz, $CDCl_3$) (*cis*/*trans* mixture) δ 200.8, 199.3, 138.6, 137.3, 137.2, 137.1, 137.0, 136.8, 136.6, 135.9, 134.4, 134.3, 134.1, 132.4, 132.2, 131.8, 129.3, 129.2, 129.2, 129.2, 129.1, 129.1, 129.0, 128.4, 127.6, 127.4, 126.7, 126.1, 59.5, 58.3, 53.5, 48.3, 27.2, 20.8. **HRMS** (ESI) m/z calculated for ($C_{22}H_{19}NNaO_3S$) 400.0978 $[M+Na]^+$; found 400.0971.

(6-Methoxy-1,1-dioxido-3-phenyl-3,4-dihydro-2*H*-benzo[*e*][1,2]thiazin-4-yl)(phenyl)methanone (2d)

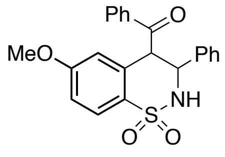

Following GP2, **2d** was prepared starting from (1-((4-methoxyphenyl)sulfonyl)-3-phenylaziridin-2-yl)(phenyl)methanone (**1d**) (39.0 mg, 0.1 mmol, 1 equiv.) and it was obtained as a 1.3:1 *trans*/*cis* mixture and isolated as white amorphous solid (13.0 mg, 33% yield, 1.3:1 d.r.). **^{1}H NMR** (500 MHz, $CDCl_3$) (*cis*/*trans* mixture) δ 7.94 (d, *J* = 8.8 Hz, 1H *cis*), 7.87 (d, *J* = 8.7 Hz, 1H *trans*), 7.77 – 7.71 (m, 4H, *cis* + *trans*), 7.57 –7.50 (m, 2H, *cis* + *trans*), 7.43 – 7.36 (m, 6H, *cis* + *trans*), 7.34 – 7.30 (m, 2H *cis*), 7.28 – 7.24 (m, 4H, *trans* + *cis*), 7.23 – 7.19 (m, 1H *trans*), 7.17 (dd, *J* = 10.4, 4.2 Hz, 1H *cis*), 6.95 (dd, *J* = 8.8, 2.5 Hz, 1H *cis*), 6.90 (ddd, *J* = 8.8, 2.5, 0.6 Hz, 1H *trans*), 6.61 (d, *J* = 12.7 Hz, 1H *cis*), 6.54 (d, *J* = 2.5 Hz, 1H *cis*), 6.48 (dd, *J* = 2.5, 0.6 Hz, 1H *trans*), 5.56 (dd, *J* = 12.7, 3.6 Hz, 1H *cis*), 5.35 – 7.28 (m, 2H *trans*), 5.20 (d, *J* = 3.6 Hz, 1H *cis*), 5.14 (d, *J* = 7.9 Hz, 1H *trans*), 3.69 (s, 6H, *cis* + *trans*). **^{13}C NMR** (126 MHz, $CDCl_3$) (*cis*/*trans* mixture) δ 200.3, 198.9, 162.6, 162.2, 138.4, 137.2, 137.2, 136.9, 135.6, 134.5, 134.3, 130.6, 130.3, 129.3, 129.2, 129.1, 129.1, 129.0, 128.4, 127.9, 127.8, 126.1, 126.1, 115.0, 114.5, 114.4, 113.2, 60.4, 59.0, 55.8, 52.4, 47.7. **HRMS** (ESI) m/z calculated for ($C_{22}H_{19}NNaO_4S$) 416.0927 $[M+Na]^+$; found 416.0930.

(6-Chloro-1,1-dioxido-3-phenyl-3,4-dihydro-2*H*-benzo[*e*][1,2]thiazin-4-yl)(phenyl)methanone (2e)

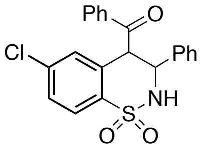

Following GP2, **2e** was prepared starting from (1-((4-chlorophenyl)sulfonyl)-3-phenylaziridin-2-yl)(phenyl)methanone (**1e**) (40.0 mg, 0.1 mmol, 1 equiv.) and it was obtained as a 1.2:1 *trans*/*cis* mixture and isolated as white amorphous solid (12.0 mg, 30% yield,1.2:1 d.r.). **^{1}H NMR** (500 MHz, $CDCl_3$) (*cis*/*trans* mixture) δ 7.94 (d, *J* = 8.5 Hz, 1H *cis*), 7.86 (d, *J* = 8.4 Hz, 1H *trans*), 7.77 – 7.68 (m, 4H, *cis* + *trans*), 7.60 – 7.52 (m, 2H, *cis* + *trans*), 7.46 – 7.37 (m, 8H, *cis* + *trans*), 7.31 – 7.15 (m, 8H, *cis* + *trans*), 7.07 (d, *J* = 2.0 Hz, 1H *cis*), 7.00 (dd, *J* = 1.7, 1.0 Hz, 1H *trans*), 6.59 (d, *J* = 12.7 Hz, 1H *cis*), 5.57 (dd, *J* = 12.7, 3.6 Hz, 1H *cis*), 5.40 (d, *J* = 10.1 Hz, 1H *trans*), 5.27– 5.21 (m, 3H, *cis* + *trans*). **^{13}C NMR** (126 MHz, $CDCl_3$) (*cis*/*trans* mixture) δ 199.8, 198.5, 139.1, 138.6, 138.1, 137.2, 137.1, 136.6, 136.6, 136.5, 135.6, 134.8, 134.6, 129.8, 129.4, 129.3, 129.2, 129.2, 129.1, 128.9, 128.8, 128.6, 127.8, 127.4, 126.1, 125.4, 60.6, 59.0, 51.3, 47.3. **HRMS** (ESI) m/z calculated for ($C_{21}H_{16}ClNNaO_3S$) 420.0432 $[M+Na]^+$; found 420.0432.

(6-Bromo-1,1-dioxido-3-phenyl-3,4-dihydro-2*H*-benzo[*e*][1,2]thiazin-4-yl)(phenyl)methanone (2f)

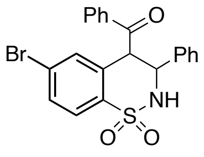

Following GP2, **2f** was prepared starting from (1-((4-bromophenyl)sulfonyl)-3-phenylaziridin-2-yl)(phenyl)methanone (**1f**) (44.0 mg, 0.1 mmol, 1 equiv.) and it was obtained as a 1.3:1 *trans*/*cis* mixture and isolated as white amorphous solid (18.0 mg, 41% yield, 3.3:1 d.r.). **^{1}H NMR** (500 MHz, $CDCl_3$) (*cis*/*trans* mixture) δ 7.88 (d, *J* = 8.4 Hz, 1H *trans*), 7.82 (d, *J* = 8.3 Hz, 1H *cis*), 7.75 – 7.69 (m, 4H, *cis* + *trans*), 7.61 (dd, *J* = 8.4, 1.9 Hz, 2H, *cis* + *trans*), 7.59 – 7.53 (m, 2H, *cis* + *trans*), 7.43 – 7.37 (m, 6H, *cis* + *trans*), 7.30 – 7.26 (m, 4H, *cis* + *trans*), 7.25 – 7.23 (m, 4H, *cis* + *trans*), 7.19 – 7.14 (m, 2H, *cis* + *trans*), 6.57 (d, *J* = 12.7 Hz, 1H *trans*), 5.56 (dd, *J* = 12.7, 3.6 Hz, 1H *trans*), 5.41 (d, *J* = 11.0 Hz, 1H *cis*), 5.24 (dd, *J* = 11.1, 8.7 Hz, 1H *cis*), 5.21 (d, *J* = 3.7 Hz, 1H *trans*), 5.05 (d, *J* = 8.7 Hz, 1H *cis*). **^{13}C NMR** (126 MHz, $CDCl_3$) (*cis*/*trans* mixture) δ 199.8, 198.5, 138.1, 137.8, 137.2, 137.1, 136.6, 136.5, 135.8, 134.8, 134.6, 132.8, 132.3, 131.8, 131.5, 129.4, 129.3, 129.2, 129.2, 129.1, 128.9, 128.6, 127.8, 127.5, 126.9, 126.1, 125.4, 60.6, 59.0, 51.2, 47.3. **HRMS** (ESI) m/z calculated for ($C_{21}H_{16}BrNNaO_3S$) 463.9926 $[M+Na]^+$; found 463.9916.

(6-Nitro-1,1-dioxido-3-phenyl-3,4-dihydro-2*H*-benzo[*e*][1,2]thiazin-4-yl)(phenyl)methanone (2g)

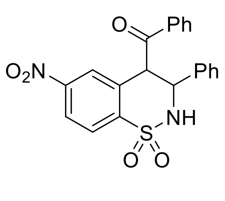

Following GP2, **2g** was prepared starting from (1-((4-nitrophenyl)sulfonyl)-3-phenylaziridin-2-yl)(phenyl)methanone (**1g**) (41.0 mg, 0.1 mmol, 1 equiv.) and it was isolated as yellow oil (4.0 mg, 10% yield, *trans*). **^{1}H NMR** (400 MHz, $CDCl_3$) (*trans*) δ 8.14 – 8.08 (m, 2H), 7.79 – 7.73 (m, 3H), 7.63 – 7.52 (m, 2H), 7.47 – 7.37 (m, 2H), 7.21 – 7.11 (m, 4H), 5.66 (d, J = 5.5 Hz, 1H), 5.40 (d, J = 7.9 Hz, 1H), 5.12 (dd, J = 7.8, 5.5 Hz, 1H). **^{13}C NMR** (101 MHz, $CDCl_3$) (*trans*) δ 191.0,

160.9, 147.8, 145.9, 137.8, 136.0, 134.5, 129.1, 129.0, 128.8, 128.7, 128.7, 128.2, 123.9, 59.1, 50.3. **HRMS** (ESI) m/z calculated for ($C_{21}H_{16}N_2O_5SNa$) 431.0672 $[M+Na]^+$; found 431.0671.

(1,1-Dioxido-3,6-diphenyl-3,4-dihydro-2*H*-benzo[*e*][1,2]thiazin-4-yl)(phenyl)methanone (2h)

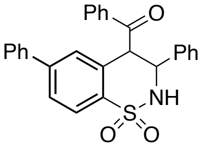

Following GP2, **2h** was prepared starting from (1-([1,1'-biphenyl]-4-ylsulfonyl)-3-phenylaziridin-2-yl)(phenyl)methanone (**1h**) (44.0 mg, 0.1 mmol, 1 equiv.) and it was obtained as a 1.9:1 *trans*/*cis* mixture and isolated as white amorphous solid (15.0 mg, 35% yield, 1.4:1 d.r.). **^{1}H NMR** (500 MHz, $CDCl_3$) (*cis*/*trans* mixture) δ 8.06 (d, *J* = 8.2 Hz, 1H, *cis*), 7.97 (d, *J* = 8.1 Hz, 1H, *trans*), 7.78 – 7.74 (m, 4H, *cis* + *trans*), 7.64 (dd, *J* = 8.2, 1.8 Hz, 1H *cis*), 7.60 (ddd, J = 8.1, 1.7, 0.7 Hz, 1H *trans*), 7.58 – 7.50 (m, 2H, *cis* + *trans*), 7.43 – 7.33 (m, 18H, *cis* + *trans*), 7.29 – 7.15 (m, 8H, *cis* + *trans*), 6.66 (d, *J* = 12.7 Hz, 1H *cis*), 5.63 (dd, *J* = 12.7, 3.5 Hz, 1H *cis*), 5.45 (d, *J* = 10.3 Hz, 1H *trans*), 5.35 – 5.29 (m, 3H, *cis* + *trans*). **^{13}C NMR** (126 MHz, $CDCl_3$) (*cis*/*trans* mixture) δ 200.5, 199.1, 145.9, 145.6, 139.3, 139.2, 138.4, 137.4, 137.1, 136.8, 136.6, 135.8, 134.6, 134.3, 134.2, 129.3, 129.3, 129.3, 129.3, 129.2, 129.2, 129.0, 128.9, 128.8, 128.5, 128.3, 128.1, 127.8, 127.8, 127.6, 127.5, 127.2, 127.1, 126.4, 126.1, 124.5, 60.6, 59.1, 52.2, 47.8. **HRMS** (ESI) m/z calculated for ($C_{27}H_{21}NNaO_3S$) 462.1134 $[M+Na]^+$; found 462.1125.

(6-Methyl-1,1-dioxido-3-phenyl-3,4-dihydro-2*H*-benzo[*e*][1,2]thiazin-4-yl)(*p*-tolyl)methanone (2i)

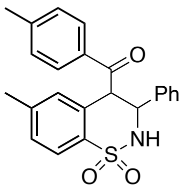

Following GP2, **2i** was prepared starting from (3-phenyl-1-tosylaziridin-2-yl)(p-tolyl)methanone (**1i**) (39.0 mg, 0.1 mmol, 1 equiv.) and it was obtained as a 1.5:1 *trans*/*cis* mixture and isolated as white amorphous solid (22.0 mg, 56% yield, 1.5:1 d.r.). **^{1}H NMR** (500 MHz, $CDCl_3$) (*cis*/*trans* mixture) δ 7.87 (d, *J* = 8.1 Hz, 1H *cis*), 7.80 (d, *J* = 8.0 Hz, 1H *trans*), 7.66 (dd, *J* = 8.4, 2.3 Hz, 4H, *cis* + *trans*), 7.41 – 7.36 (m, 2H, *cis* + *trans*), 7.30 (dd, *J* = 8.0, 1.5 Hz, 2H *trans*), 7.27 – 7.15 (m, 12H, *cis* + *trans*), 6.90 – 6.83 (m, 1H *cis*), 6.79 (d, *J* = 1.6 Hz, 1H *trans*), 6.66 (d, *J* = 12.6 Hz, 1H *cis*), 5.54 (dd, *J* = 12.7, 3.6 Hz, 1H *cis*), 5.33 (d, *J* = 11.0 Hz, 1H *trans*), 5.26 (dd, *J* = 11.1, 9.1 Hz, 1H *trans*), 5.18 (d, *J* = 3.7 Hz, 1H *cis*), 5.16 (d, *J* = 9.1 Hz, 1H *trans*), 2.38 (s, 3H *cis*), 2.36 (s, 3H *trans*), 2.25 (s, 6H, *cis* + *trans*). **^{13}C NMR** (126 MHz, $CDCl_3$) δ 200.0, 198.7, 145.8, 145.4, 143.6, 143.2, 138.5, 137.2, 135.6, 135.4, 135.3, 134.9, 134.3, 133.9, 130.2, 123.0, 129.8, 129.4, 129.2, 129.1, 129.1, 128.9, 128.8, 128.3, 127.8, 126.1, 125.8, 124.0, 60.5, 59.1, 51.8, 47.1, 22.0, 22.0, 22.0, 21.8. **HRMS** (ESI) m/z calculated for ($C_{23}H_{21}NNaO_3S$) 414.1134 $[M+Na]^+$; found 414.1137.

(4-Methoxyphenyl)(6-methyl-1,1-dioxido-3-phenyl-3,4-dihydro-2*H*-benzo[*e*][1,2]thiazin-4-yl)methanone (2j)

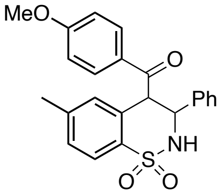

Following GP2, **2j** was prepared starting from (4-methoxyphenyl)(3-phenyl-1-tosylaziridin-2-yl)methanone (**1j**) (41.0 mg, 0.1 mmol, 1 equiv.) and it was obtained as a 1.7:1 *trans*/*cis* mixture and isolated as white amorphous solid (23.0 mg, 56% yield, 1.7:1 d.r.). **^{1}H NMR** (500 MHz, $CDCl_3$) (*cis*/*trans* mixture) δ 7.86 (d, *J* = 8.0 Hz, 1H *cis*), 7.78 (d, *J* = 8.1 Hz, 1H *trans*), 7.77 – 7.74 (m, 4H, *cis* + *trans*), 7.40 – 7.36 (m, 2H, *cis* + *trans*), 7.30 – 7.13 (m, 10H, *cis* + *trans*), 6.90 – 6.79 (m, 6H, *cis* + *trans*), 6.71 (d, *J* = 12.6 Hz, 1H *cis*), 5.52 (dd, *J* = 12.6, 3.6 Hz, 1H *cis*), 5.30 (d, *J* = 10.9 Hz, 1H *trans*), 5.27 (d, *J* = 8.8 Hz, 1H *trans*), 5.25 – 5.22 (m, 1H *trans*), 5.15 (d, *J* = 3.6 Hz, 1H *cis*), 3.84 (s, 3H *cis*), 3.82 (s, 3H *trans*), 2.25 (s 3H, *cis*), 2.25 (s, 3H *trans*). **^{13}C NMR** (126 MHz, $CDCl_3$) (*cis*/*trans* mixture) δ 198.6, 197.4, 164.8, 164.5, 143.6, 143.2, 138.6, 137.3, 135.6, 135.3, 134.2, 131.7, 131.5, 130.4, 130.1, 130.0, 129.8, 129.7, 129.1, 129.0, 128.9, 128.8, 128.3, 127.8, 126.7, 126.1, 125.7, 123.9, 114.5, 114.4, 60.6, 59.1, 55.9, 55.9, 51.6, 46.8, 22.0, 21.8. **HRMS** (ESI) m/z calculated for ($C_{23}H_{21}NNaO_4S$) 430.1083 $[M+Na]^+$; found 430.1082.

(3-Methoxyphenyl)(6-methyl-1,1-dioxido-3-phenyl-3,4-dihydro-2*H*-benzo[*e*][1,2]thiazin-4-yl)methanone (2k)

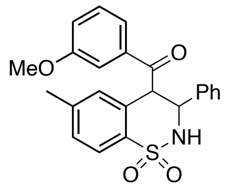

Following GP2, **2k** was prepared starting from (3-methoxyphenyl)(3-phenyl-1-tosylaziridin-2-yl)methanone (**1k**) (41.0 mg, 0.1 mmol, 1 equiv.) and it was obtained as a 1.7:1 *trans*/*cis* mixture and isolated as white amorphous solid (23.0 mg, 55% yield, 1.7:1 d.r.). ^{1}**H NMR** (500 MHz, $CDCl_3$) (*cis*/*trans* mixture) δ 7.88 (d, *J* = 8.1 Hz, 1H *cis*), 7.80 (d, *J* = 8.0 Hz, 1H *trans*), 7.38 – 7.33 (m, 4H, *cis* + *trans*), 7.32 – 7.29 (m, 4H, *cis* + *trans*), 7.28 – 7.24 (m, 6H, *cis* + *trans*), 7.24 – 7.17 (m, 4H, *cis* + *trans*), 7.11 – 7.05 (m, 2H, *cis* + *trans*), 6.88 (s, 1H *cis*), 6.80 (s, 1H *trans*), 6.59 (d, *J* = 12.8 Hz, 1H *cis*), 5.54 (dd, *J* = 12.7, 3.6 Hz, 1H *cis*), 5.32 – 5.26 (m, 2H *trans*), 5.21 (dd, *J* = 7.1, 2.1 Hz, 1H *trans*), 5.18 (d, *J* = 3.6 Hz, 1H *cis*), 3.77 (s, 6H, *cis* + *trans*), 2.26 (s, 6H, *cis* + *trans*). ^{13}**C NMR** (126 MHz, $CDCl_3$) (*cis*/*trans* mixture) δ 200.4, 199.2, 160.2, 143.6, 143.3, 138.6, 138.2, 137.1, 135.3, 135.2, 133.7, 130.2, 130.2, 130.1, 129.9, 129.2, 129.2, 129.1, 128.9, 128.4, 127.8, 126.1, 125.8, 124.1, 121.8, 121.7, 121.1, 121.0, 113.2, 112.8, 60.5, 59.0, 55.7, 52.4, 47.5, 22.0, 21.7. **HRMS** (ESI) m/z calculated for ($C_{23}H_{21}NNaO_4S$) 430.1083 $[M+Na]^+$; found 430.1082.

(2-Methoxyphenyl)(6-methyl-1,1-dioxido-3-phenyl-3,4-dihydro-2*H*-benzo[*e*][1,2]thiazin-4-yl)methanone (2l)

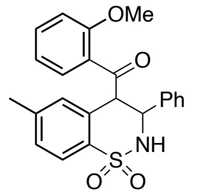

Following GP2, **2l** was prepared starting from (2-methoxyphenyl)(3-phenyl-1-tosylaziridin-2-yl)methanone (**1l**) (41.0 mg, 0.1 mmol, 1 equiv.) and it was obtained as a 1.7:1 *trans*/*cis* mixture and isolated as white amorphous solid (26.0 mg, 64% yield, 1.3:1 d.r.). ^{1}**H NMR** (500 MHz, $CDCl_3$) (*cis*/*trans* mixture) δ 7.85 (d, *J* = 8.0 Hz, 1H *cis*), 7.77 (d, *J* = 8.0 Hz, 1H *trans*), 7.46 – 7.37 (m, 2H, *cis* + *trans*), 7.37 – 7.32 (m, 2H *cis*), 7.30 – 7.14 (m, 12H, *cis* + *trans*), 6.97 (dd, *J* = 9.4, 1.4 Hz, 2H, *cis* + *trans*), 6.92 (dd, *J* = 8.4, 1.0 Hz, 1H *cis*), 6.87 – 6.83 (m, 2H *trans*), 6.84 – 6.79 (m, 1H *cis*), 6.43 (d, *J* = 12.6 Hz, 1H *cis*), 5.66 (d, *J* = 3.7 Hz, 1H *cis*), 5.51 – 5.46 (m, 2H, *cis* + *trans*), 5.18 (t, *J* = 9.8 Hz, 1H *trans*), 5.01 (d, *J* = 9.4 Hz, 1H *trans*), 3.91 (s, 3H *cis*), 3.67 (s, 3H *trans*), 2.31 (s, 3H *trans*), 2.25 (s, 3H *cis*). ^{13}**C NMR** (126 MHz, $CDCl_3$) (*cis*/*trans* mixture) δ 202.6, 201.2, 158.3, 143.1, 142.9, 138.5, 137.5, 135.8, 135.4, 135.1, 135.0, 134.7, 134.3, 131.7, 131.2, 130.3, 130.0, 129.3, 128.9, 128.7, 128.7, 128.7, 128.5, 128.0, 128.0, 127.5, 126.4, 125.5, 123.9, 121.4, 121.2, 112.0, 111.8, 60.4, 58.9, 55.9, 55.9, 55.8, 51.6, 22.0, 21.8. **HRMS** (ESI) m/z calculated for ($C_{23}H_{21}NNaO_4S$) 430.1083 $[M+Na]^+$; found 430.1067.

(6-Methyl-1,1-dioxido-3-phenyl-3,4-dihydro-2*H*-benzo[*e*][1,2]thiazin-4-yl)(naphthalen-2-yl)methanone (2m)

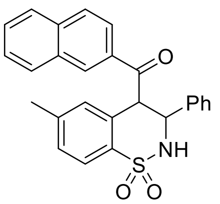

Following GP2, **2m** was prepared starting from naphthalen-2-yl(3-phenyl-1-tosylaziridin-2-yl)methanone (**1m**) (43.0 mg, 0.1 mmol, 1 equiv.) and it was obtained as a 1.8:1 *trans/cis* mixture and isolated as yellow oil (30.0 mg, 70% yield, 1.8:1 d.r.). ^{1}**H NMR** (400 MHz, $CDCl_3$) (*trans/cis* mixture) δ 8.29 – 8.25 (m, 1H *trans*, 1H *cis*), 7.95 – 7.75 (m, 8H *trans*), 7.67 – 7.50 (m, 8H *cis*), 7.43 (d, *J* = 8.1 Hz, 2H *trans*), 7.35 (d, *J* = 7.5 Hz, 2H *cis*), 7.30 – 7.19 (m, 3H trans), 7.19 – 7.07 (m, 3H *cis*), 6.92 (s, 1H *cis*), 6.85 (s, 1H *trans*), 6.66 (d, *J* = 12.7 Hz, 1H *cis*), 5.62 (dd, *J* = 12.7, 3.6 Hz, 1H *cis*), 5.53 (d, *J* = 11.0 Hz, 1H *trans*), 5.37 (d, *J* = 4.4 Hz, 1H *cis*), 5.34 (d, *J* = 11.4 Hz, 1H *trans*), 5.14 (d, *J* = 9.2 Hz, 1H *trans*), 2.24 (s, 3H *trans*), 2.22 (s, 3H *cis*). ^{13}**C NMR** (101 MHz, $CDCl_3$) (*trans/cis* mixture) δ 200.2, 198.8, 143.5, 143.1, 138.5, 137.0, 136.0, 136.0, 135.6, 135.3, 135.2, 134.5, 134.1, 133.7, 132.5, 132.4, 131.3, 131.1, 130.1, 130.0, 129.6, 129.5, 129.3, 129.1, 129.0, 129.0, 128.9, 128.8, 128.7, 128.3, 127.9, 127.9, 127.6, 127.3, 127.2, 126.0, 125.7, 124.0, 123.9, 123.8, 60.5, 59.0, 51.8, 47.4, 21.8, 21.6. **HRMS** (ESI) m/z calculated for ($C_{26}H_{21}NO_3SNa$) 450.1134 $[M+Na]^+$; found 450.1137.

(3-(4-Chlorophenyl)-6-methyl-1,1-dioxido-3,4-dihydro-2*H*-benzo[*e*][1,2]thiazin-4-yl)(phenyl)methanone (2n)

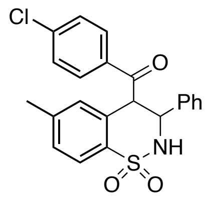

Following GP2, **2n** was prepared starting from (3-(4-chlorophenyl)-1-tosylaziridin-2-yl)(phenyl)methanone (**1n**) (41.0 mg, 0.1 mmol, 1 equiv.) and it was obtained as a 1:1 *trans/cis* mixture and isolated as white amorphous solid (26.0 mg, 62% yield, 1.25:1 d.r.). **^{1}H NMR** (500 MHz, $CDCl_3$) (*cis/trans* mixture) δ 7.88 (d, *J* = 8.1 Hz, 1H *cis*), 7.81 (d, *J* = 8.0 Hz, 1H *trans*), 7.67 (dd, *J* = 11.0, 8.6 Hz, 4H, *cis* + *trans*), 7.38 – 7.33 (m, 6H, *cis* + *trans*), 7.29 – 7.16 (m, 10H, *cis* + *trans*), 6.83 (bs, 1H *cis*), 6.76 (bs, 1H *trans*), 6.49 (d, *J* = 12.8 Hz, 1H *cis*), 5.55 (dd, *J* = 12.8, 3.7 Hz, 1H *cis*), 5.30 (d, *J* = 11.0 Hz, 1H *trans*), 5.25 – 5.21 (m, 1H *trans*), 5.18 – 5.12 (m, 2H, *cis* + *trans*), 2.29 – 2.25 (m, 6H, *cis* + *trans*). **^{13}C NMR** (126 MHz, $CDCl_3$) (*cis/trans* mixture) δ 199.2, 197.9, 143.6, 143.2, 141.1, 140.8, 138.1, 136.8, 135.5, 135.4, 135.1, 135.0, 134.8, 133.3, 130.3, 130.2, 130.1, 129.5, 129.4, 129.4, 129.1, 129.0, 128.9, 128.6, 128.4, 127.6, 125.9, 125.7, 123.9, 60.4, 58.8, 52.0, 47.5, 21.8, 21.6. **HRMS** (ESI) m/z calculated for ($C_{22}H_{18}ClNNaO_3S$) 434.0588 $[M+Na]^+$; found 434.0577.

(4-Bromophenyl)(6-methyl-1,1-dioxido-3-phenyl-3,4-dihydro-2*H*-benzo[*e*][1,2]thiazin-4-yl)methanone (2o)

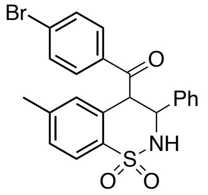

Following GP2, **2o** was prepared starting from (4-bromophenyl)(3-phenyl-1-tosylaziridin-2-yl)methanone (**1o**) (45.0 mg, 0.1 mmol, 1 equiv.) and it was obtained as a 1.3:1 *trans/cis* mixture and isolated as white amorphous solid (22.0 mg, 58% yield, 2.0:1 d.r.). **^{1}H NMR** (400 MHz, $CDCl_3$) (*cis/ trans* mixture) δ 7.88 (d, *J* = 8.1 Hz, 1H *cis*), 7.82 (d, *J* = 8.0 Hz, 1H *trans*), 7.65 – 7.58 (m, 3H *trans*), 7.58 – 7.51 (m, 4H, *cis* + *trans*), 7.41 – 7.37 (m, 4H, *cis* + *trans*), 7.31 – 7.30 (m, 4H, *cis* + *trans*), 7.28 – 7.22 (m, 5H, *cis* + *trans*), 6.85 (s, 1H *cis*), 6.75 (s, 1H *trans*), 6.49 (d, *J* = 12.8 Hz, 1H *cis*), 5.55 (dd, *J* = 12.8, 3.7 Hz, 1H *cis*), 5.28 – 5.11 (m, 4H, *cis* + *trans*), 2.28 (s, 6H, *cis* + *trans*). **^{13}C NMR** (100 MHz, $CDCl_3$) (*cis/trans* mixture) δ 199.4, 198.1, 143.6, 143.2, 138.1, 136.8, 135.7, 135.5, 134.7, 133.3, 132.4, 132.4, 130.3, 130.2, 129.6, 129.5, 129.1, 129.0, 128.9, 128.5, 127.6, 125.9, 125.7, 123.9, 60.3, 58.8, 52.0, 47.5, 21.8, 21.6. **HRMS** (ESI) m/z calculated for ($C_{22}H_{18}BrNNaO_3S$) 478.0089 $[M+Na]^+$; found 478.0091.

(4-Fluorophenyl)(6-methyl-1,1-dioxido-3-phenyl-3,4-dihydro-2*H*-benzo[*e*][1,2]thiazin-4-yl)methanone (2p)

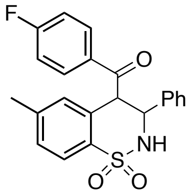

Following GP2, **2p** was prepared starting from (4-fluorophenyl)(3-phenyl-1-tosylaziridin-2-yl)methanone (**1p**) (39.5 mg, 0.1 mmol, 1 equiv.) and it was obtained as a 1.7:1 *trans/cis* mixture and isolated as white amorphous solid (23.0 mg, 58 % yield, 1.7:1 d.r.). **^{1}H NMR** (400 MHz, $CDCl_3$) (*cis/trans* mixture) δ 7.87 (d, *J* = 8.1 Hz, 1H *cis*), 7.77 (m, 6H, *cis* + *trans*), 7.39 – 7.34 (m, 2H *trans*), 7.29 – 7.15 (m, 8H *cis* + *trans*), 7.09 – 6.99 (m, 5H, *cis* + *trans*), 6.85 (bs, 1H *cis*), 6.77 (bs, 1H *trans*), 6.52 (d, *J* = 12.8 Hz, 1H *cis*), 5.54 (dd, *J* = 12.8, 3.7 Hz, 1H *cis*), 5.32 (d, *J* = 10.0 Hz, 1H *trans*), 5.28 – 5.19 (m, 2H *trans*), 5.15 (d, *J* = 3.7 Hz, 1H *cis*), 2.27 (s, 6H, *cis* + *trans*). **^{13}C NMR** (100 MHz, $CDCl_3$) (*cis/trans* mixture) δ 198.7, 197.5, 167.7, 167.5, 165.2, 165.0, 143.5, 143.2, 138.1, 136.8, 135.4, 135.08, 134.90, 133.58, 133.55, 133.47, 131.77, 131.67, 131.58, 131.49, 130.2, 129.5, 129.1, 129.0, 128.8, 128.7, 128.6, 128.3, 127.6, 125.9, 125.7, 123.9, 116.4, 116.2, 60.4, 58.8, 51.9, 47.4, 21.8, 21.6. **HRMS** (ESI) m/z calculated for ($C_{22}H_{18}FNNaO_3S$) 418.0884 $[M+Na]^+$; found 418.0881.

(3-Fluorophenyl)(6-methyl-1,1-dioxido-3-phenyl-3,4-dihydro-2*H*-benzo[*e*][1,2]thiazin-4-yl)methanone (2q)

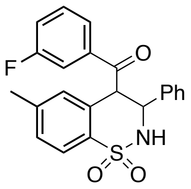

Following GP2, **2q** was prepared starting from 2(3-fluorophenyl)(3-phenyl-1-tosylaziridin-2-yl)methanone (**1q**) (39.5 mg, 0.1 mmol, 1 equiv.) and it was obtained as a 1.8:1 *trans/cis* mixture and isolated as white amorphous solid (28.0 mg, 70% yield, 5.4:1 d.r.). **^{1}H NMR** (500 MHz, $CDCl_3$) (*cis/trans* mixture) δ 7.90 (d, *J* = 8.1 Hz, 1H *cis*), 7.84 (d, *J* = 8.0 Hz, 1H *trans*), 7.51 – 7.49 (m, 2H, *cis* + *trans*), 7.46 – 7.43 (m, 2H, *cis* + *trans*), 7.40 – 7.33 (m, 6H, *cis* + *trans*), 7.31 – 7.20 (m, 10H, *cis* + *trans*), 6.85 (bs, 1H *cis*), 6.78 (bs, 1H *trans*), 6.45 (d, *J* = 12.8 Hz, 1H *cis*), 5.57 (dd, *J* = 12.8, 3.7 Hz, 1H *cis*), 5.33 – 5.31

(m, 1H *trans*), 5.26 – 5.21 (m, 1H *trans*), 5.13 (d, *J* = 3.7 Hz, 1H *cis*), 5.02 (d, *J* = 8.9 Hz, 1H *trans*), 2.30 – 2.28 (m, 6H, *cis* + *trans*). **^{13}C NMR** (126 MHz, $CDCl_3$) (*cis/trans* mixture) δ 198.0, 198.0, 164.0, 162.0, 143.6, 139.2, 139.1, 138.2, 135.7, 134.7, 130.8, 130.8, 129.1, 129.1, 129.1, 128.9, 128.5, 127.6, 125.9, 124.5, 124.5, 123.9, 121.3, 121.1, 115.4, 115.3, 60.4, 58.8, 52.1, 47.9, 21.8, 21.6. **^{19}F NMR** (376 MHz, $CDCl_3$) (*cis/trans* mixture) δ -110.71 (s, *cis*), -110.81 (s, *trans*). **HRMS** (ESI) m/z calculated for ($C_{22}H_{18}FNNaO_3S$) 418.0884 $[M+Na]^+$; found 418.0890.

(2-Fluorophenyl)(6-methyl-1,1-dioxido-3-phenyl-3,4-dihydro-2*H*-benzo[*e*][1,2]thiazin-4-yl)methanone (2r)

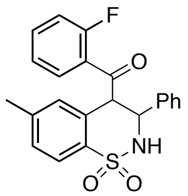

Following GP2, **2r** was prepared starting from (2-fluorophenyl)(3-phenyl-1-tosylaziridin-2-yl)methanone (**1r**) (40.0 mg, 0.1 mmol, 1 equiv.) and it was obtained as a 1:1 *trans*/*cis* mixture and isolated as white amorphous solid (20.0 mg, 51% yield, 1:1 d.r.). **^{1}H NMR** (400 MHz, $CDCl_3$) (*cis*/*trans* mixture) δ 7.90 (d, *J* = 8.1 Hz, 1H *cis*), 7.81 (d, *J* = 8.0 Hz, 1H *trans*), 7.54 – 7.46 (m, 2H, *cis* + *trans*), 7.46 – 7.40 (m, 2H, *cis* + *trans*), 7.38 – 7.27 (m, 8H, *cis* + *trans*), 7.25 – 7.14 (m, 4H, *cis* + *trans*), 7.13 – 7.06 (m, 4H, *cis* + *trans*), 7.05 (s, 1H *cis*), 6.91 (s, 1H, *trans*), 6.28 (d, *J* = 12.8 Hz, 1H, *cis*), 5.57 (dd, *J* = 12.8, 3.7 Hz, 1H, *cis*), 5.39 (d, *J* = 3.5 Hz, 1H, *cis*), 5.37 (d, *J* = 8.2 Hz, 1H, *trans*), 5.24 – 5.17 (m, 2H, *trans*), 2.33 (s, 3H, *cis*), 2.31 (s, 3H, *trans*). **^{13}C NMR** (100 MHz, $CDCl_3$) δ 199.1 (d, *J* = 4.0 Hz), 198.5 (d, *J* = 3.2 Hz), 161.5 (d, *J* = 255.1 Hz) 161.3 (d, *J* = 255.1 Hz), 143.5, 143.4, 138.0 136.8, 136.0 (d, *J* = 9.5 Hz), 135.6, 135.5 (d, *J* = 9.1 Hz), 135.1, 135.0, 131.6 (d, *J* = 1.7 Hz), 130.9 (d, *J* = 1.6 Hz), 130.3, 130.0 (d, *J* = 2.4 Hz), 128.8 (d, *J* = 23.8 Hz), 129.1 (d, *J* = 21.2 Hz), 128.2, 127.8, 126.9 (d, *J* = 11.2 Hz), 126.0, 125.7, 125.1 (d, *J* = 3.3 Hz), 125.0 (d, *J* = 3.5 Hz), 124.1, 117.0 (d, *J* = 23.1 Hz), 117.0 (d, *J* = 23.9 Hz), 60.6, 58.7, 55.9 (d, *J* = 6.2 Hz), 52.2 (d, *J* = 8.5 Hz), 22.0, 21.8. **^{19}F NMR** (376 MHz, $CDCl_3$) (*cis*/*trans* mixture) δ -110.35 (ddd, *J* = 15.1, 7.7, 4.0 Hz), -110.92 -111.02 (m). **HRMS** (ESI) m/z calculated for ($C_{22}H_{18}$ $FNNaO_3S$) 418.0889 $[M+Na]^+$; found 418.0868.

4-Methyl-*N*-(3-oxo-1-phenyl-3-(pyridin-2-yl)propyl)benzenesulfonamide (2s')

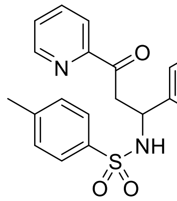

Following GP2, **2s'** was prepared starting from (3-phenyl-1-tosylaziridin-2-yl)(pyridin-2-yl)methanone (**1s**) (38.0 mg, 0.1 mmol, 1 equiv.) and isolated as yellow oil (17.0 mg, 45% yield). **^{1}H NMR** (600 MHz, $CDCl_3$) δ 8.69 – 8.64 (m, 1H), 7.89 (d, *J* = 7.8 Hz, 1H), 7.81 (td, *J* = 7.7, 1.8 Hz, 1H), 7.51 (d, *J* = 8.3 Hz, 2H), 7.49 (ddd, *J* = 7.5, 4.7, 1.3 Hz, 1H), 7.24 – 7.22 (m, 2H), 7.22 – 7.14 (m, 3H), 7.09 (d, *J* = 7.9 Hz, 2H), 6.16 (d, *J* = 6.5 Hz, 1H), 4.92 – 4.86 (m, 1H), 3.78 (dd, *J* = 16.1, 7.5 Hz, 1H), 3.47 (dd, *J* = 16.1, 5.3 Hz, 1H), 2.34 (s, 3H). **^{13}C NMR** (151 MHz, $CDCl_3$) δ 199.1, 152.8, 149.0, 143.1, 140.7, 137.5, 137.2, 129.4, 128.6, 127.7, 127.6, 127.2, 126.7, 122.3, 54.9, 44.7, 21.6. **HRMS** (ESI) m/z calculated for ($C_{21}H_{18}N_2O_3SNaH_2$) 403.1086 $[M+Na]^+$; found 403.1086.

(6-Methyl-1,1-dioxido-3-phenyl-3,4-dihydro-2*H*-benzo[*e*][1,2]thiazin-4-yl)(thiophen-2-yl)methanone (2t)

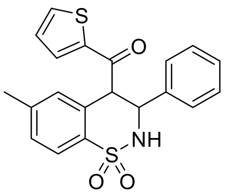

Following GP2, **2t** was prepared starting from (3-phenyl-1-tosylaziridin-2-yl)(thiophen-2-yl)methanone (**1t**) (38.3 mg, 0.1 mmol, 1 equiv.) and isolated as light brown solid (24.0 mg, 63% yield, *trans*). **^{1}H NMR** (600 MHz, $CDCl_3$) (*trans*) δ 7.83 (d, *J* = 8.0 Hz, 1H), 7.64 (dd, *J* = 4.9, 1.1 Hz, 1H), 7.53 (dd, *J* = 3.9, 1.1 Hz, 1H), 7.40 (d, *J* = 12.2 Hz, 2H), 7.30 (d, *J* = 0.9 Hz, 4H), 7.03 (dd, *J* = 4.9, 3.9 Hz, 1H), 6.91 (d, *J* = 1.7 Hz, 1H), 5.28 (dd, *J* = 11.2, 9.2 Hz, 1H), 5.11 (d, *J* = 11.1 Hz, 1H), 4.99 (d, *J* = 9.3 Hz, 1H), 2.31 (s, 3H). **^{13}C NMR** (101 MHz, $CDCl_3$) (*trans*) δ 190.7, 144.1, 143.4, 137.9, 135.8, 135.3, 134.4, 133.5, 129.0, 128.9, 128.7, 128.7, 127.4, 123.7, 60.2, 53.8, 21.7. **HRMS** (ESI) m/z calculated for (C_{20} $H_{17}NO_{32}Na$) 406.0542 $[M+Na]^+$; found 406.0541.

(6-Methyl-1,1-dioxido-3-(*p*-tolyl)-3,4-dihydro-2*H*-benzo[*e*][1,2]thiazin-4-yl)(phenyl)methanone (2w)

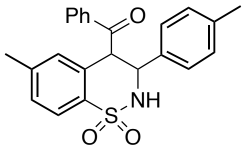

Following GP2, **2w** was prepared starting from phenyl(3-(*p*-tolyl)-1-tosylaziridin-2-yl)methanone (**1w**) (39.1 mg, 0.1 mmol, 1 equiv.) and it was obtained as a 1.3:1 *trans/cis* mixture and isolated as white amorphous solid (24.0 mg, 61% yield, 1.6:1 d.r.). **^{1}H NMR** (500 MHz, $CDCl_3$) (*cis*/*trans* mixture) δ 7.88 (d, *J* = 8.1 Hz, 1H *cis*), 7.82 (d, *J* = 8.0 Hz, 1H *trans*), 7.77 – 7.73 (m, 4H, *cis* + *trans*), 7.58 – 7.51 (m, 2H *trans*), 7.43 – 7.37 (m, 4H, *cis* + *trans*), 7.25 – 7.22 (m, 4H, *cis* + *trans*), 7.18 (d, *J* = 8.1 Hz, 2H *cis*), 7.07 – 7.03 (m, 4H, *cis* + *trans*), 6.86 (bs, 1H *cis*), 6.80 (bs, 1H *trans*), 6.57 (d, *J* = 12.7 Hz, 1H *cis*), 5.51 (dd, *J* = 12.7, 3.6 Hz, 1H *cis*), 5.33 – 5.23 (m, 2H *trans*), 5.19 (d, *J* = 3.6 Hz, 1H *cis*), 5.04 (d, *J* = 8.9 Hz, 1H *trans*), 2.27 – 2.24 (m, 9H, *cis* + *trans*), 2.22 (s, 3H *cis*). **^{13}C NMR** (126 MHz, $CDCl_3$) (*cis/trans* mixture) δ 200.5, 199.0, 143.4, 143.0, 138.6, 137.9, 137.1, 136.8, 135.4, 135.2, 135.1, 135.0, 134.2, 134.0, 134.0, 133.6, 130.0, 129.7, 129.6, 129.6, 129.1, 129.0, 129.0, 129.0, 128.8, 128.7, 127.5, 125.8, 125.7, 123.9, 60.0, 58.7, 52.2, 47.3, 21.8, 21.6, 21.2, 21.1. **HRMS** (ESI) m/z calculated for ($C_{23}H_{21}NNaO_3S$) 414.1134 $[M+Na]^+$; found 414.1122.

(6-Methyl-1,1-dioxido-3-(*o*-tolyl)-3,4-dihydro-2*H*-benzo[*e*][1,2]thiazin-4-yl)(phenyl)methanone (2x)

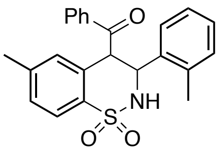

Following GP2, **2x** was prepared starting from 3-(*o*-tolyl)-1-tosylaziridin-2-yl)methanone (**1x**) (39.1 mg, 0.1 mmol, 1 equiv.) and it was obtained as a 1.8:1 *trans/cis* mixture and isolated as white amorphous solid (27.0 mg, 69% yield, 3.3:1 d.r.). **^{1}H NMR** (300 MHz, $CDCl_3$) (*cis*/*trans* mixture) δ 7.88 (d, *J* = 8.1 Hz, 1H *cis*), 7.81 – 7.72 (m, 4H, *cis* + *trans*), 7.62 – 7.50 (m, 3H, *cis* + *trans*), 7.49 – 7.27 (m, 7H, *cis* + *trans*), 7.18 (d, *J* = 8.2 Hz, 1H *trans*), 7.16 – 6.96 (m, 5H, *cis* + *trans*), 6.95 (td, *J* = 7.4, 1.8 Hz, 1H *cis*), 6.83 (br s, 2H *cis* + *trans*), 6.54 (d, *J* = 12.7 Hz, 1H *cis*), 5.70 – 5.60 (m, 1H, *cis* + *trans*), 5.46 (d, *J* = 11.0 Hz, 1H *trans*), 5.16 – 5.07 (m, 1H, *cis* + *trans*), 2.51 (s, 3H *cis*), 2.27 – 2.25 (m, 9H, *cis* + *trans*). **^{13}C NMR** (75 MHz, $CDCl_3$) (*cis*/*trans* mixture) δ 200.3, 199.0, 143.4, 143.1, 137.0, 136.9, 135.4, 135.3, 135.1, 134.7, 134.2, 133.9, 131.1, 130.9, 130.1, 129.5, 129.2, 129.1, 128.9, 128.9, 128.8, 128.8, 128.6, 128.2, 126.8, 126.1, 125.6, 124.4, 56.3, 55.4, 51.2, 45.9, 21.7, 21.5, 19.6, 19.3. **HRMS** (ESI) m/z calculated for ($C_{23}H_{21}NNaO_3S$) 414.1134 $[M+Na]^+$; found 414.1138.

(3-(3-Methoxyphenyl)-6-methyl-1,1-dioxido-3,4-dihydro-2*H*-benzo[*e*][1,2]thiazin-4-yl)(phenyl)methanone (2y)

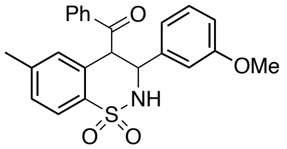

Following GP2, **2y** was prepared starting from (3-(3-methoxyphenyl)-1-tosylaziridin-2-yl)(phenyl)methanone (**1y**) (39.2 mg, 0.1 mmol, 1 equiv.) and it was obtained as a 1.2:1 *trans/cis* mixture and isolated as white amorphous solid (27.0 mg, 66% yield, 1.2:1 d.r.). **^{1}H NMR (**500 MHz, $CDCl_3$) (*cis*/*trans* mixture) δ 7.87 (d, *J* = 8.1 Hz, 1H cis), 7.82 – 7.76 (m, 5H, *cis* + *trans*), 7.60 – 7.52 (m, 2H *trans*), 7.45 – 7.37 (m, 4H, *cis* + *trans*), 7.27 – 7.21 (m, 2H, *cis* + *trans*), 7.19 – 7.11 (m, 2H, *cis* + *trans*), 6.95 – 6.92 (m, 2H, *cis* + *trans*), 6.90 – 6.84 (m, 3H, *cis* + *trans*), 6.80 (bs, 1H *trans*), 6.74 (ddd, *J* = 8.3, 2.3, 1.2 Hz, 1H *trans*), 6.69 (dd, *J* = 8.1, 2.5 Hz, 1H *cis*), 6.58 (d, *J* = 12.7 Hz, 1H *cis*), 5.52 (dd, *J* = 12.7, 3.6 Hz, 1H *cis*), 5.34 (d, *J* = 11.0 Hz, 1H *trans*), 5.28 – 5.21 (m, 2H, *cis* + *trans*), 5.14 (d, *J* = 9.3 Hz, 1H *trans*), 3.73 – 3.70 (m, 6H, *cis* + *trans*), 2.27 – 2.24 (m, 6H, *cis* + *trans*). **^{13}C NMR** (126 MHz, $CDCl_3$) (*cis/trans* mixture) δ 200.3, 199.1, 160.0, 160.0, 143.5, 143.1, 139.7, 138.5, 137.2, 136.7, 135.4, 135.1, 135.0, 134.3, 134.0, 133.5, 130.1, 130.1, 130.0, 129.7, 129.1, 129.0, 129.0, 129.0, 128.8, 128.7, 125.6, 123.9, 119.7, 118.2, 114.6, 113.9, 113.0, 111.7, 60.3, 58.8, 55.4, 55.4, 51.9, 47.1, 21.8, 21.6. **HRMS** (ESI) m/z calculated for ($C_{23}H_{21}NNaO_4S$) 430.1083 $[M+Na]^+$; found 430.1089.

(3-(4-Fluorophenyl)-6-methyl-1,1-dioxido-3,4-dihydro-2*H*-benzo[*e*][1,2]thiazin-4-yl)(phenyl)methanone (2z)

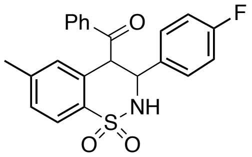

Following GP2, **2z** was prepared starting from 2-benzoyl-3 (3-(4-fluorophenyl)-1-tosylaziridin-2-yl)(phenyl)methanone (**1z**) (39.5 mg, 0.1 mmol, 1 equiv.) and it was obtained as a 1.3:1 *trans/cis* mixture and isolated as white amorphous solid (23.0 mg, 59% yield, 1.5:1 d.r.). **^{1}H NMR** (400 MHz, $CDCl_3$) (*cis*/*trans* mixture) δ 7.87 (d, *J* = 8.1 Hz, 1H *cis*), 7.81 – 7.73 (m, 4H, *cis* + *trans*), 7.61 – 7.52 (m, 2H, *cis* + *trans*), 7.46 – 7.37 (m, 6H, *cis* + *trans*), 7.31 – 7.21 (m, 4H, *cis* + *trans*), 6.93 (td, *J* = 8.7, 2.3 Hz,

4H, *cis* + *trans*), 6.87 (dt, 1H *cis*), 6.79 (m, 1H, *trans*), 6.59 (d, *J* = 12.6 Hz, 1H *cis*), 5.53 (dd, *J* = 12.5, 3.7 Hz, 1H *cis*), 5.36 (d, *J* = 9.4 Hz, 1H *trans*), 5.27 – 5.17 (m, 3H *cis* + *trans*), 2.26 (s, 3H *trans*), 2.25 (s, 3H *cis*). **^{13}C NMR** (100 MHz, $CDCl_3$) (*cis*/*trans* mixture) δ 200.3, 199.0, 163.9, 163.6, 161.4, 161.1, 143.5, 143.2, 137.1, 136.6, 135.6, 135.0, 135.0, 134.5, 134.5, 134.5, 134.3, 133.3, 132.9, 130.1, 129.7, 129.6, 129.6, 129.2, 129.0, 129.0, 128.7, 128.6, 127.8, 127.7, 125.7, 123.7, 116.0, 115.8, 59.7, 58.3, 53.6, 51.6, 47.1, 21.8, 21.6. **^{19}F NMR** (376 MHz, $CDCl_3$) (*cis/trans* mixture) δ -113.10 (d, *J* = 2.2 Hz, *trans*), -113.62 – -113.66 (m, *cis*). **HRMS** (ESI) m/z calculated for ($C_{22}H_{18}FNNaO_3S$) 418.0884 $[M+Na]^+$; found 418.0882.

(3-(4-Chlorophenyl)-6-methyl-1,1-dioxido-3,4-dihydro-2*H*-benzo[e][1,2]thiazin-4-yl)(phenyl)methanone (2za)

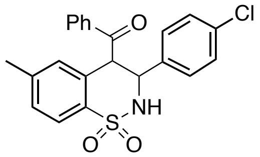

Following GP2, **2za** was prepared starting from (3-(4-chlorophenyl)-1-tosylaziridin-2-yl)(phenyl)methanone (**1za**) (41.0 mg, 0.1 mmol, 1 equiv.) and it was obtained as a 1.2:1 *trans*/*cis* mixture and isolated as white amorphous solid (28.0 mg, 69% yield, 1.2:1 d.r.). **^{1}H NMR** (500 MHz, $CDCl_3$) (*cis*/*trans* mixture) δ 7.85 (d, *J* = 8.1 Hz, 1H *cis*), 7.79 – 7.75 (m, 5H, *cis* + *trans*), 7.63 – 7.53 (m, 2H, *cis* + *trans*), 7.47 – 7.38 (m, 4H, *cis* + *trans*), 7.37 – 7.33 (m, 2H, *cis* + *trans*), 7.27 – 7.18 (m, 8H, *cis* + *trans*), 6.88 (s, 1H *cis*), 6.78 (d, *J* = 0.4 Hz, 1H trans), 6.62 (d, *J* = 12.6 Hz, 1H *cis*), 5.51 (dd, *J* = 12.6, 3.6 Hz, 1H *cis*), 5.35 (d, *J* = 11.1 Hz, 1H *trans*), 5.32 (d, *J* = 9.3 Hz, 1H *trans*), 5.23 – 5.18 (m, 2H, *cis* + *trans*), 2.25 (s, 3H *trans*), 2.24 (s, 3H *cis*). **^{13}C NMR** (126 MHz, $CDCl_3$) (*cis*/*trans* mixture) δ 200.4, 199.0, 143.7, 143.4, 137.4, 137.2, 136.6, 135.8, 135.7, 135.1, 134.8, 134.6, 134.5, 134.3, 133.3, 130.3, 129.9, 129.4, 129.4, 129.4, 129.3, 129.3, 129.2, 129.1, 128.9, 128.8, 127.5, 125.8, 123.9, 59.8, 58.5, 51.4, 46.9, 22.0, 21.8. **HRMS** (ESI) m/z calculated for ($C_{22}H_{18}ClNNaO_3S$) 434.0588 $[M+Na]^+$; found 434.0585.

(3-(2-Chlorophenyl)-6-methyl-1,1-dioxido-3,4-dihydro-2*H*-benzo[e][1,2]thiazin-4-yl)(phenyl)methanone (2zb)

Following GP2, **2zb** was prepared starting from (3-(2-chlorophenyl)-1-tosylaziridin-2-yl)(phenyl)methanone (**1zb**) (41.2 mg, 0.1 mmol, 1 equiv.) and it was obtained as a 2.0:1 *trans/cis* mixture. Both *cis* (9.3 mg, 23%) and *trans* (18.7 mg, 45%) stereoisomers were isolated as white amorphous solids (28.0 mg, 68% combined yield).

(3*S*,4*R*)/(3*R*,4*S*)-(*cis*-2zb)

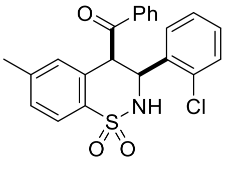

^{1}H NMR (400 MHz, $CDCl_3$) (*cis* stereoisomer) δ 7.91 (d, *J* = 8.1 Hz, 1H), 7.72 (dd, *J* = 8.5, 1.3 Hz, 2H), 7.58 – 7.49 (m, 1H), 7.42 – 7.34 (m, 2H), 7.34 – 7.26 (m, 3H), 7.17 – 7.06 (m, 3H), 6.86 (bs, 1H), 6.58 (d, *J* = 12.9 Hz, 1H), 5.82 (dd, *J* = 12.8, 3.7 Hz, 1H), 5.52 (d, *J* = 3.7 Hz, 1H), 2.28 (s, 3H). **^{13}C NMR** (100 MHz, $CDCl_3$) (*cis* stereoisomer) δ 200.3, 143.3, 136.6, 135.1, 134.5, 134.2, 133.7, 131.7, 130.1, 129.8, 129.7, 129.6, 129.0, 128.8, 128.0, 127.6, 125.6, 56.4, 44.7, 21.6.

(3*R*,4*R*)/(3*S*,4*S*)-(*trans*-2zb)

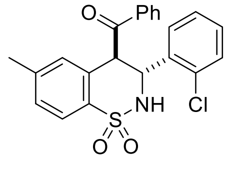

^{1}H NMR (400 MHz, $CDCl_3$) (*trans* stereoisomer) δ 7.85 – 7.76 (m, 3H), 7.55 (ddt, *J* = 8.7, 7.0, 1.3 Hz, 1H), 7.43 – 7.35 (m, 3H), 7.30 – 7.23 (m, 2H), 7.18 – 7.13 (m, 2H), 6.81 (bs, 1H), 5.75 – 5.64 (m, 2H), 5.49 (d, *J* = 9.9 Hz, 1H), 2.27 (s, 3H). **^{13}C NMR** (100 MHz, $CDCl_3$) (*trans* stereoisomer) δ 198.5, 143.5, 136.9, 135.2, 135.0, 134.1, 133.2, 130.6, 130.2, 129.2, 129.1, 129.0, 128.8, 127.8, 124.3, 49.8, 21.8.

HRMS (ESI) m/z calculated for ($C_{22}H_{18}ClNNaO_3S$) 434.0588 $[M+Na]^+$; found 434.0591.

(6-Methyl-1,1-dioxido-3,4-dihydro-2*H*-benzo[e][1,2]thiazin-4-yl)(phenyl)methanone (2zc)

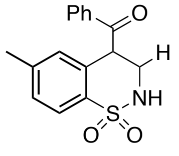

Following GP2, **2zc** was prepared starting from phenyl(1-tosylaziridin-2-yl)methanone (**1zc**) (30.1 mg, 0.1 mmol, 1 equiv.) and it was isolated as white amorphous solid (11.0 mg, 35% yield). **^{1}H NMR** (300 MHz, $CDCl_3$) δ 8.09 – 7.99 (m, 2H), 7.81 (d, *J* = 8.1 Hz, 1H), 7.74 – 7.67 (m, 1H), 7.62 – 7.54 (m, 2H), 7.26 – 7.20 (m, 1H), 6.87 (bs, 1H), 5.45 (dd, *J* = 11.8, 3.4 Hz, 1H), 4.80 (dd, *J* = 4.7, 2.2 Hz, 1H), 4.27 (ddd, *J* = 15.2, 12.0, 4.6 Hz, 1H), 3.84 (ddd, *J* = 15.2, 3.5, 2.2 Hz, 1H), 2.26 (s, 3H). **^{13}C NMR** (100 MHz, $CDCl_3$) δ 199.9,

143.0, 135.7, 135.5, 134.7, 133.2, 130.0, 129.8, 129.4, 129.3, 125.2, 44.9, 42.2, 21.6. **HRMS** (ESI) m/z calculated for ($C_{16}H_{15}NNaO_3S$) 324.0665 $[M+Na]^+$; found 324.0666.

(3,6-Dimethyl-1,1-dioxido-3,4-dihydro-2*H*-benzo[*e*][1,2]thiazin-4-yl)(phenyl)methanone (2zd)

Following GP2, **2zd** was prepared starting from (3-methyl-1-tosylaziridin-2-yl)(phenyl)methanone (**1zd**) (30.1 mg, 0.1 mmol, 1 equiv.) and it was obtained as a 1.3:1 *trans/cis* mixture. The *cis* (11.7 mg, 37%) and *trans* (15.0 mg, 48%) steroisomers were isolated as white amorphous solids (26.7 mg, 85% combined yield).

(3*S*,4*R*)/(3*R*,4*S*)-(*cis*-2zd)

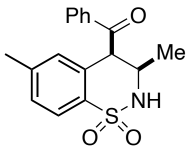

^{1}H NMR (400 MHz, $CDCl_3$) (*trans* stereoisomer) δ 8.11 – 8.01 (m, 2H), 7.83 (d, *J* = 8.0 Hz, 1H), 7.75 – 7.67 (m, 1H), 7.60 (t, *J* = 7.8 Hz, 1H), 7.22 (dd, *J* = 8.1, 1.7 Hz, 1H), 6.83 (br s, 1H), 5.77 (d, *J* = 12.5 Hz, 1H), 4.83 (d, *J* = 3.6 Hz, 1H), 4.43 (dqd, *J* = 12.5, 6.9, 3.5 Hz, 1H), 2.24 (s, 3H), 1.34 (d, *J* = 7.0 Hz, 3H). **^{13}C NMR** (100 MHz, $CDCl_3$) (*trans* stereoisomer) δ 200.4, 142.9, 137.0, 134.8, 133.9, 129.9, 129.5, 129.4, 129.3, 129.2, 125.5, 52.1, 45.9, 21.5, 19.7.

(3*R*,4*R*)/(3*S*,4*S*)-(*trans*-2zd)

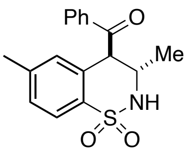

^{1}H NMR (400 MHz, $CDCl_3$) (*cis* stereoisomer) δ 7.96 (d, *J* = 7.1 Hz, 2H), 7.78 (d, *J* = 8.0 Hz, 1H), 7.64 (t, *J* = 7.4 Hz, 1H), 7.51 (t, *J* = 7.7 Hz, 2H), 7.21 (d, *J* = 8.1 Hz, 1H), 6.77 (s, 1H), 4.80 (d, *J* = 10.7 Hz, 1H), 4.63 (d, *J* = 9.7 Hz, 1H), 4.47 – 4.33 (m, 1H), 2.24 (s, 3H), 1.34 (d, *J* = 6.6 Hz, 3H). **^{13}C NMR** (100 MHz, $CDCl_3$) (*cis* stereoisomer) δ 199.3, 143.3, 136.2, 134.5, 134.3, 134.2, 129.2, 129.2, 129.2, 129.2, 128.9, 124.4, 77.4, 77.0, 76.7, 53.6, 52.1, 21.6, 20.7.
HRMS (ESI) m/z calculated for ($C_{17}H_{17}NNaO_3S$) 338.0827 $[M+Na]^+$; found 338.0811.

4-Methyl-2-(2-phenylquinolin-3-yl)benzenesulfonamide (2ze')

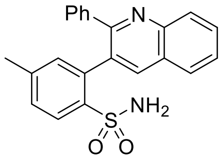

Following GP2, **2ze'** was prepared starting from 2-phenyl-1-tosyl-1a,7b-dihydro-1*H*-azirino[2,3-c]quinoline (**1ze**) (38.0 mg, 0.1 mmol, 1 equiv.) and isolated as light brown solid (7.1 mg, 19% yield). **^{1}H NMR** (400 MHz, $CDCl_3$) δ 8.48 (s, 1H), 8.05 (d, *J* = 9.0 Hz, 1H), 7.87 (d, *J* = 8.3 Hz, 1H), 7.67 (d, *J* = 16.9 Hz, 1H), 7.58 (d, *J* = 15.0 Hz, 1H), 7.53 – 7.41 (m, 4H), 7.22 – 7.08 (m, 3H), 6.78 (s, 1H), 2.38 (s, 3H). **^{13}C NMR** (101 MHz, $CDCl_3$) δ 153.3, 151.4, 144.6, 136.0, 130.0, 129.7, 129.6, 129.5, 129.4, 129.2, 128.6, 128.6, 127.9, 127.6, 127.6, 127.3, 126.5, 21.7. **HRMS** (ESI) m/z calculated for ($C_{22}H_{19}N_2O_2S$) 375.1162 $[M+H]^+$; found 375.1161.

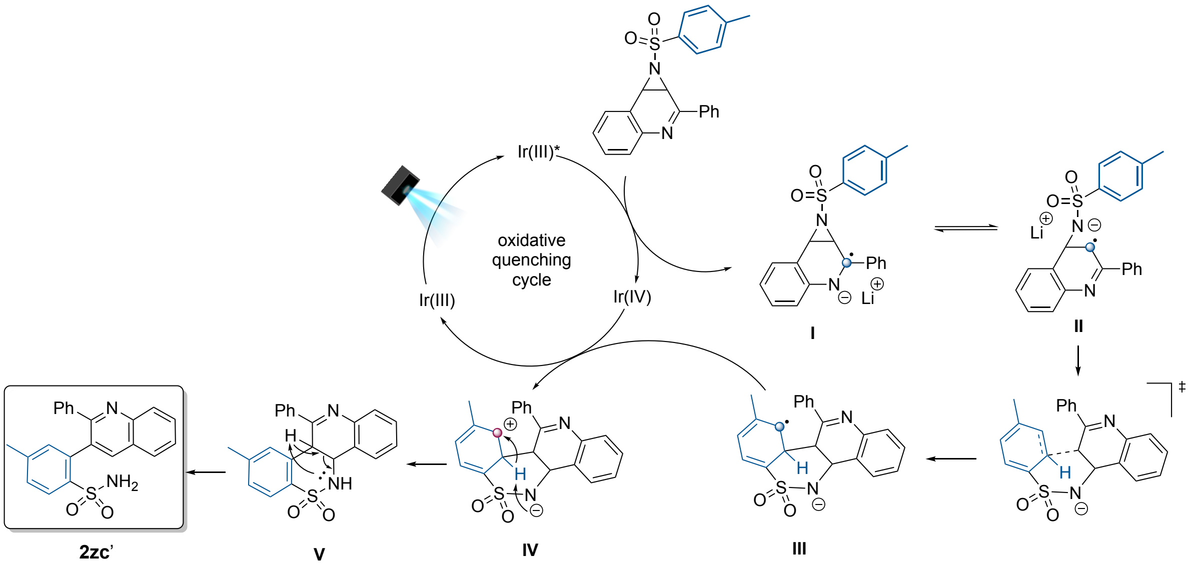

***Scheme S7**. Proposed mechanism for obtaining **2ze'**.*

(3*R*,4*R*)/(3*S*,4*S*)-2-Benzyl-6-methyl-1,1-dioxido-3-phenyl-3,4-dihydro-2*H*-benzo[*e*][1,2]thiazin-4-yl)(phenyl)methanone (*trans*-3a)

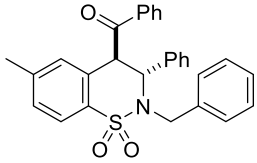

The sultam **2a** (190.0 mg, 0.5 mmol, 1.00 equiv.; 1.7:1 d.r. *trans/cis*) was dissolved in DCM (0.5 M). Benzylbromide (90.7 mg, 0.53 mmol, 1.05 equiv.) and K_2CO_3 (138.2 mg, 1.0 mmol, 2.00 equiv.) were added and the mixture was heated to 80 ºC and the mixture stirred for 5 hours. The reaction was cooled to r.t., filtered and concentrated under reduced pressure. The crude product obtained as a *trans*-enriched mixture (6:1 *trans/cis*) was purified by flash column chromatography (Hexane/EtOAc 10:1) and the *trans* isomer of the product **3a** was isolated as a white solid (121.5 mg, 52%, >20:1 d.r.). **^{1}H NMR** (400 MHz, $CDCl_3$) (*trans* stereoisomer) δ 7.78 (d, *J* = 7.9 Hz, 1H), 7.70 – 7.65 (m, 2H), 7.42 (t, *J* = 7.4 Hz, 1H), 7.37 – 7.34 (m, 2H), 7.29 – 7.24 (m, 2H), 7.18 – 7.00 (m, 7H), 6.90 (dd, *J* = 7.8, 1.7 Hz, 2H), 6.64 (s, 1H), 5.64 (d, *J* = 11.5 Hz, 1H), 4.65 (d, *J* = 11.6 Hz, 1H), 4.49 (d, *J* = 15.1 Hz, 1H), 4.09 (d, *J* = 15.1 Hz, 1H), 2.20 (s, 3H). **^{13}C NMR** (100 MHz, $CDCl_3$) (*trans* stereoisomer) δ 198.5, 142.8, 138.5, 137.4, 136.8, 135.2, 134.5, 133.9, 128.8, 128.8, 128.5, 128.4, 128.3, 128.3, 128.0, 128.0, 127.9, 127.6, 122.7, 77.3, 77.0, 76.7, 64.2, 52.3, 49.7, 21.7. **HRMS** (ESI) m/z calculated for ($C_{29}H_{25}NNaO_3S$) 490.1453 $[M+Na]^+$; found 490.1440.

(2,6-Dimethyl-1,1-dioxido-3-phenyl-3,4-dihydro-2*H*-benzo[*e*][1,2]thiazin-4-yl)(phenyl)methanone (*trans*-4a)

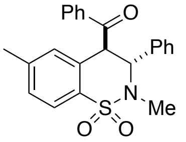

To a solution of sultam **2a** (19.0 mg, 0.050 mmol, 1.0 equiv.) in dry DCM under argon atmosphere at room temperature, Me_3OBF_4 (19.0 mg, 0.128 mmol, 2.6 equiv.) and DIPEA (26 µL, 0.150 mmol, 3 equiv.) were added. The reaction was stirred for 3 h. Then, the solvent was evaporated and the crude mixture purified by column chromatography (gradient EA/PE 1:10 to 1:4), providing the product **5a** as yellowish oil (13.7 mg, 70%, *trans*). **^{1}H NMR** (400 MHz, $CDCl_3$) δ 7.80 (dd, *J* = 10.3, 7.6 Hz, 3H), 7.53 (dd, *J* = 16.9, 7.7 Hz, 3H), 7.37 (t, *J* = 7.7 Hz, 2H), 7.30 – 7.24 (m, 4H), 7.23 – 7.17 (m, 1H), 6.83 (s, 1H), 5.63 (d, *J* = 11.7 Hz, 1H), 4.74 (d, *J* = 11.7 Hz, 1H), 2.74 (s, 3H), 2.29 (s, 3H). **^{13}C NMR** (101 MHz, $CDCl_3$) δ 198.9, 143.2, 138.1, 137.3, 134.8, 134.6, 134.1, 129.0, 128.8, 128.6, 128.5, 128.5, 128.5, 128.1, 123.9, 67.4, 49.3, 36.2, 21.9. **HRMS** (ESI) m/z calculated for ($C_{23}H_{21}NO_3SNa$) 414.1145 $[M+Na]^+$; found 414.1133.

(*E*/*Z*)-4-Methyl-2-(3-oxo-1,3-diphenylprop-1-en-2-yl)-*N,N*-diphenylbenzenesulfonamide (5a)

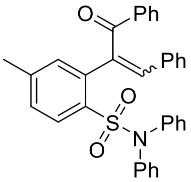

To a solution of sultam **2a** (12.0 mg, 0.030 mmol mmol, 1 equiv.; 1.7:1 d.r. *trans/cis*) in dry MeCN under argon atmosphere at room temperature, 2-(trimethylsilyl)phenyl trifluoromethanesulfonate (18 µL, 0.072 mmol, 2.4 equiv.) and CsF (22.0 mg, 0.144 mmol, 4.8 equiv.) were added. The reaction was stirred for 24 h. Then, the solvent was evaporated and the crude mixture purified by column chromatography (gradient EA/PE 1:15 to 1:4), providing the product **5a** as a white solid (8.7 mg, 55%, *E/Z* mixture 3:1). **^{1}H NMR** (400 MHz, $CDCl_3$) (*E/Z)* δ 7.72 – 7.66 (m, 2H), 7.63 (d, *J* = 8.2 Hz, 1H), 7.47 – 7.40 (m, 1H), 7.30 – 7.23 (m, 3H), 7.23 – 7.14 (m, 7H), 7.14 – 7.01 (m, 7H), 7.00 – 6.92 (m, 2H), 6.88 – 6.78 (m, 1H), 2.36 (s, 3H). **^{13}C NMR** (151 MHz, $CDCl_3$) δ 196.9, 196.8, 144.5, 144.4, 143.4, 142.2, 141.5, 141.5, 139.4, 139.2, 137.9, 137.9, 137.0, 136.7, 135.9, 135.9, 134.5, 134.3, 134.2, 132.7, 132.6, 131.7, 131.6, 131.4, 130.8, 130.5, 130.4, 129.5, 129.1, 129.0, 128.9, 128.8, 128.8, 128.8, 128.5, 128.5, 128.0, 120.0, 127.1, 127.1, 115.7, 115.6, 21.6, 21.6. **HRMS** (ESI) m/z calculated for ($C_{34}H_{27}NO_3SNa$) 552.1609 $[M+Na]^+$; found 552.1605.

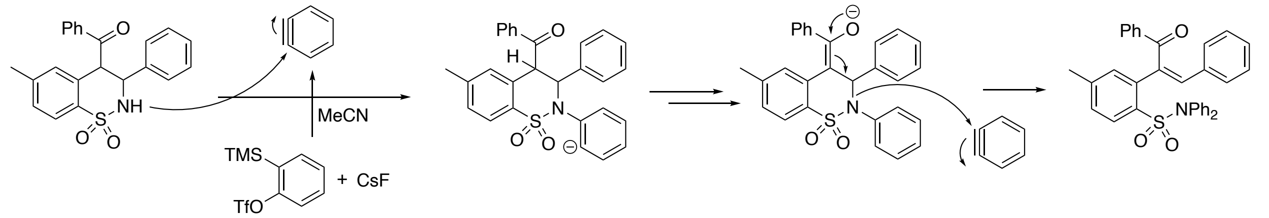

***Scheme S8**. Proposed mechanism for obtaining **5a**.*

5. Mechanistic Studies

CV experiments

Cyclic voltammetry measurements were performed with a Potentiostat PARSTAT 2273 equipped with a glassy carbon electrode as working electrode and platinum wire as counter electrode. Silver wire (Ag/AgCl/NaCl 3M, 0.220 V vs NHE) was used as pseudo reference electrode and a 0.1 M solution of tetrabutylammonium tetrafluoroborate in dry and degassed acetonitrile was applied as electrolyte. Cyclic voltammetry of **1a** was measured in absence and presence of $LiClO_4$, and in the second case every voltammogram at a different scan rate was recorder with a new freshly prepared solution of **1a** and $LiClO_4$ in dry and degassed acetonitrile.

Solutions were degassed with argon prior measurement and experiments were performed under argon atmosphere. Every scan was measured in positive direction first. Reduction potentials (E_{red}) were extrapolated considering pick potentials (E_p). The final potential was converted from Ag/AgCl to SCE.[55]

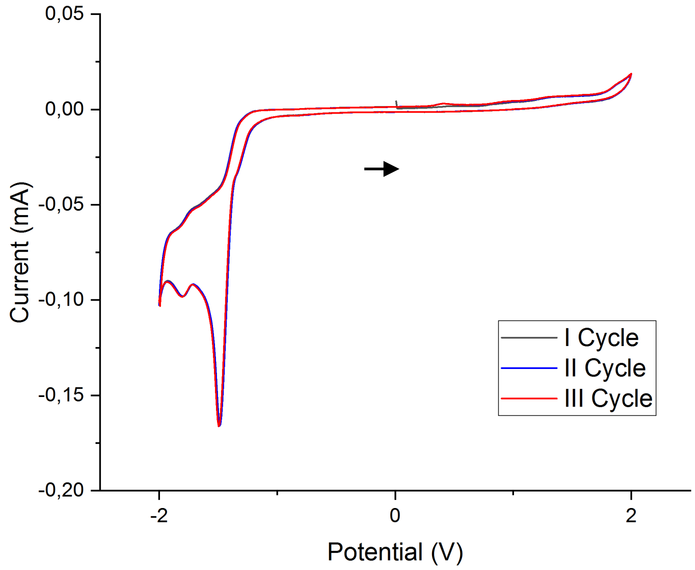

Figure S5. *Voltammograms of **1a** (4.2 mM). Scan rate= 40 mV/s; 3 consecutive cycles.* E_{red}^{I} *= -1.34 V vs SCE;* E_{red}^{II} *= -1.79 V vs SCE.*

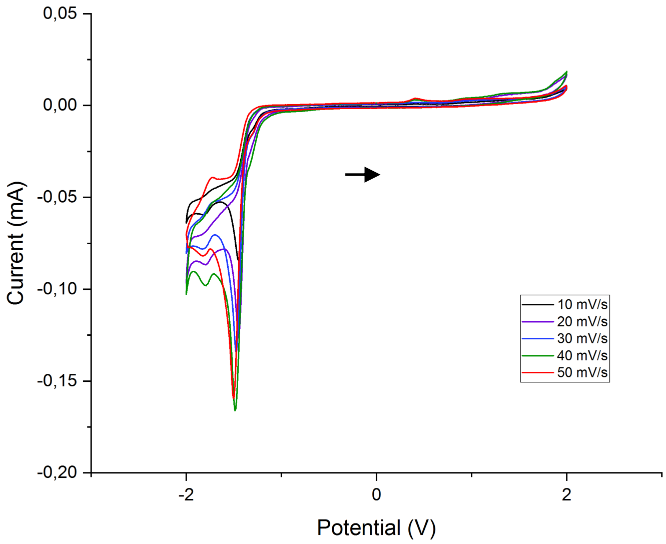

Figure S6. *Voltammograms of **1a** (4.2 mM) at different scan rates.*

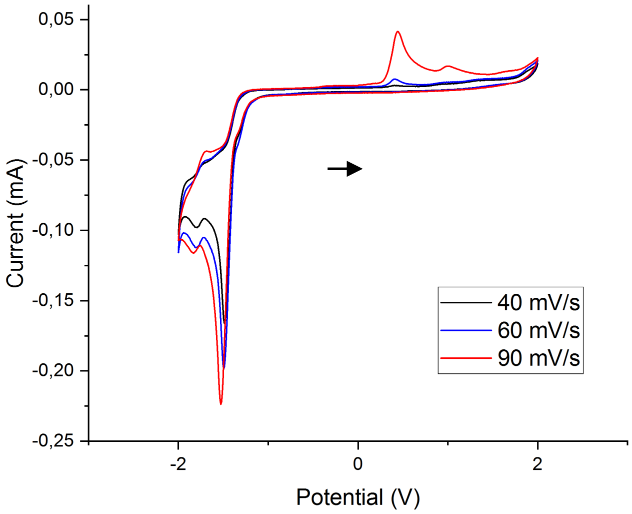

Figure S7. *Voltammograms of* ***1a*** *(4.2 mM) at different scan rates. From scan rates higher than 40 mV/s it is observable the cathodic picks (E_{ox}^{I}= 0.29 V vs SCE; E_{ox}^{II}=0.90 vs SCE).*

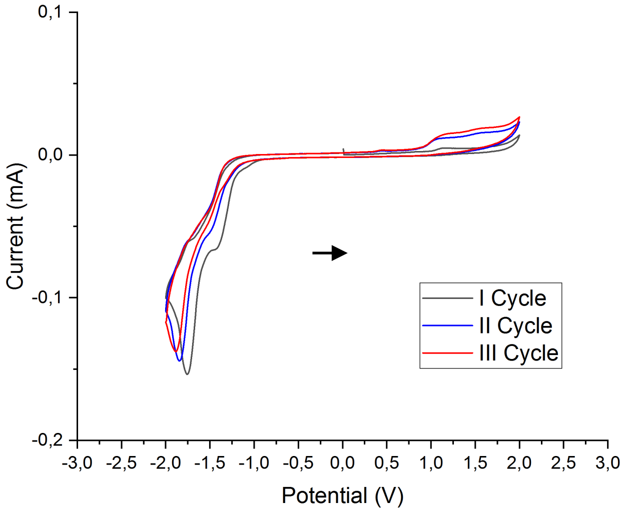

Figure S8. *Voltammograms of* ***1a*** *(6.4 mM) in presence of $LiClO_4$ (10 mM). Scan rate= 40 mV/s. 3 scan cycles scan. E_{red}^{I}= -1.20 V vs SCE; E_{red}^{II}= -1.65 V vs SCE.*

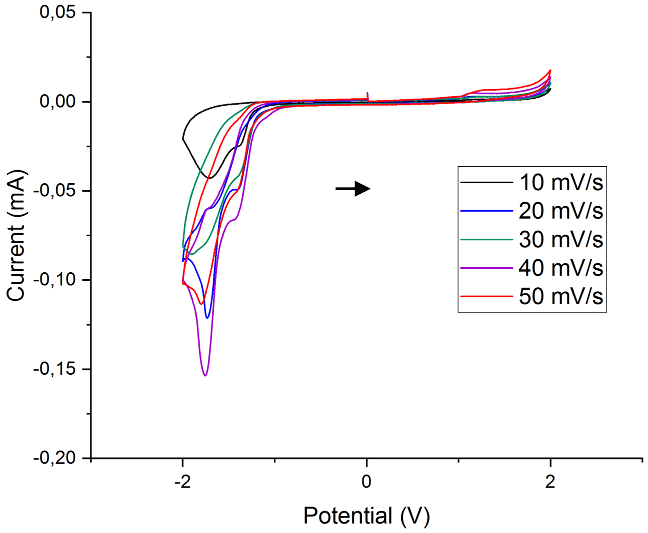

Figure S9. *Voltammograms of* ***1a*** *and $LiClO_4$ at different scan rates. [**1a**] 6.1 mM, [$LiClO_4$] 9.4 mM, scan rate 10 mV/s (black trace); [**1a**] 6.6 mM, [$LiClO_4$] 9.0 mM, scan rate 20 mV/s (blue trace); [**1a**] 6.1 mM, [$LiClO_4$] 9.4 mM, scan rate 30 mV/s (green trace); [**1a**] 6.4 mM, [$LiClO_4$] 10 mM, scan rate 40 mV/s (purple trace); [**1a**] 5.8 mM, [$LiClO_4$] 9.4 mM, scan rate 10 mV/s (red trace).*

Stern-Volmer studies

The emission spectra were conducted on a Jasco FP-8500 spectrofluorometer and with a 1 cm cuvette. To perform Stern-Volmer experiments three stock solution with dry and degassed MeCN were prepared: 1) a 10 mL, 0.2 mM solution of $Ir(ppy)_3$; 2) a 3.5 mL, 86 mM solution of substrate **1a**; 3) a 3.5 mL, 86 mM solution of substrate **1a** and 86 mM of $LiClO_4$. The fluorescence measurements were conducted at an excitation wavelength of 450 nm and the emission was detected in a range from 460 nm to 800 nm.

To tack the quenching of the phosphorescence of $Ir(ppy)_3$, the quenchers were added with progressively higher concentration, keeping constant the concentration of $Ir(ppy)_3$ to 0.1 mM. The Stern-Volmer quenching constant K_{SV} was calculated according to the Stern-Volmer equation:

$$\frac{I_0}{I} = 1 + Ksv\tau_o[Q]$$

where I_0 is the luminescence without the quencher, I is the intensity with the quencher, τ_0 is the lifetime of the excited photocatalyst (1.6 μs for $Ir(ppy)_3$)[56] and [Q] is the concentration of the quencher.

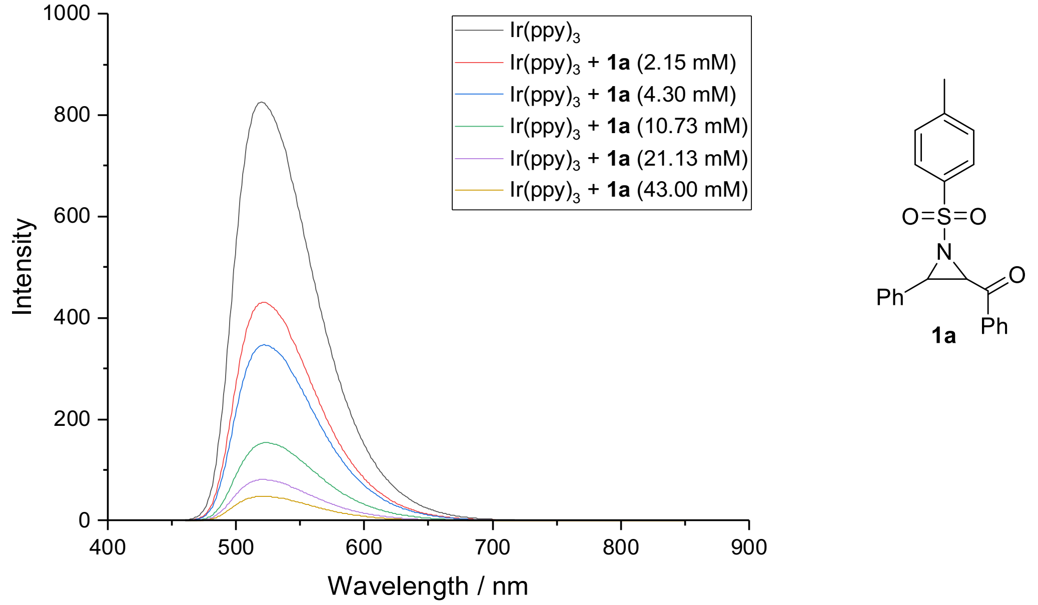

Figure S10. *Emission spectra (λ_{ex}= 450 nm) of the Stern-Volmer quenching with substrate **1a**.*

Entry	Quencher [mM]	I(0)/I
1	0	1
2	2.15	1.9163
3	4.3	2.3786
4	10.73	5.3478
5	21.13	10.1022
6	43	16.8491

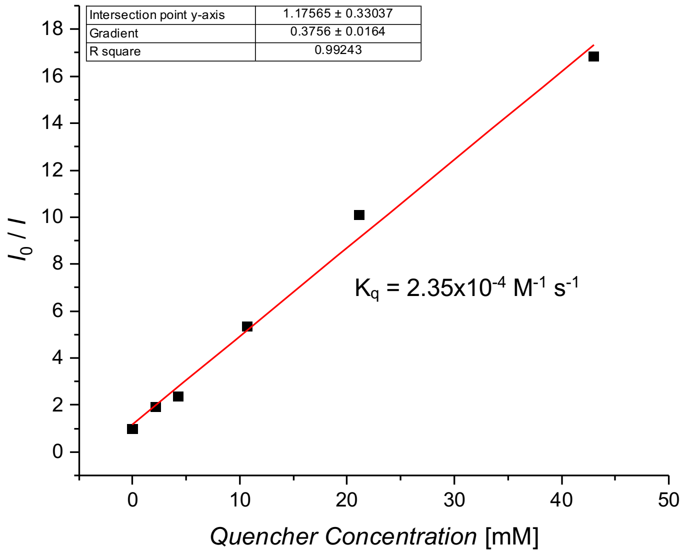

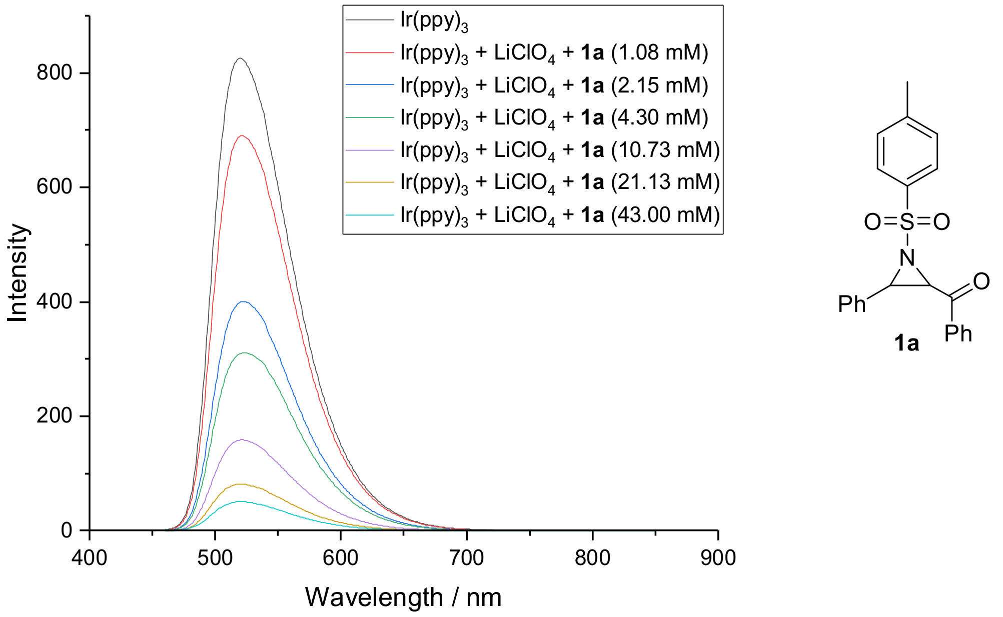

Figure S11. *Emission spectra (λ_{ex}= 450 nm) of the Stern-Volmer quenching with substrate **1a** and $LiClO_4$.*

Entry	Quencher [mM]	I(0)/I
1	0	1
2	2.15	1.9163
3	4.3	2.3786
4	10.73	5.3478
5	21.13	10.1022
6	43	16.8491

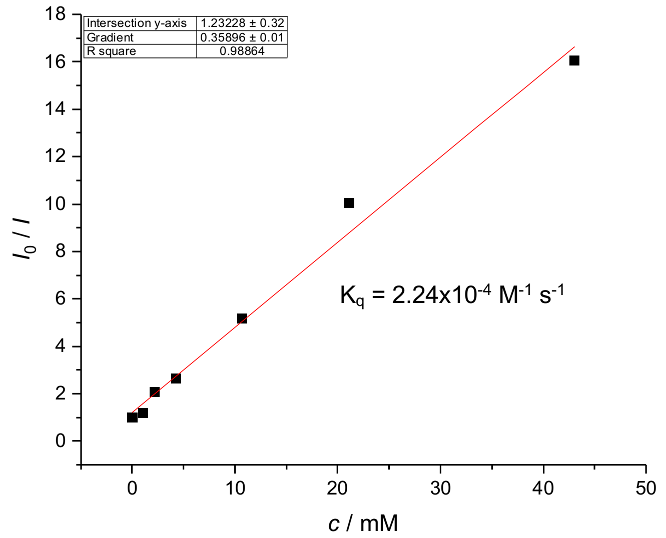

Radical trapping experiment

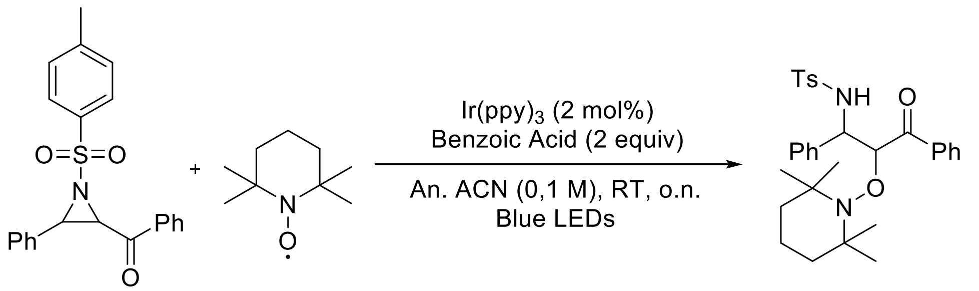

1a **6a**

Scheme S9*. Radical trapping experiment with TEMPO.*

Aziridine **1a** (38.0 mg, 0.1 mmol, 1 equiv.) was mixed in a 10 mL vial with $Ir(ppy)_3$ (1.0 mg, 0.02 mmol, 2 mol%), TEMPO (31.0 mg 0.2 mmol, 2 equiv.) and benzoic acid (24.0 mg, 0.2 mmol, 2 equiv.). Then, the vial was sealed and three cycles

of vacuum-refill with Ar were performed. After adding degassed acetonitrile (1 mL), the mixture was stirred under blue LEDs irradiation overnight. Afterwards, the vial was opened, 10 mL of EtOAc were added and the mixture was washed with water three times (3 x 10 mL) and brine (10 mL). The separated organic phase was then dried over $MgSO_4$, filtered, and concentrated under vacuum. The fraction containing **6a** was filtrated through silica gel (EtOAc) and analysed by HPLC-MS (Column: Zorbax Eclipse C18 (100x4.6mm,3.5µm); Mobile phase: H_2O:MeOH 30:70 →70% MeOH up to 100% in 10', hold 5'; Flow: 1mL/min; APCI).

```
Sample Info     : Zorbax Eclipse  C18 100 x 4.6mm,3.5µm
                  H2O / MeOH 30:70
                  70% MeOH up to 100% in 10', hold 5
                  1mL/min
                  APCI +/-
                  Sample: 1mg/mL in MeOH
                  HPLCMS1

Additional Info : Peak(s) manually integrated
```

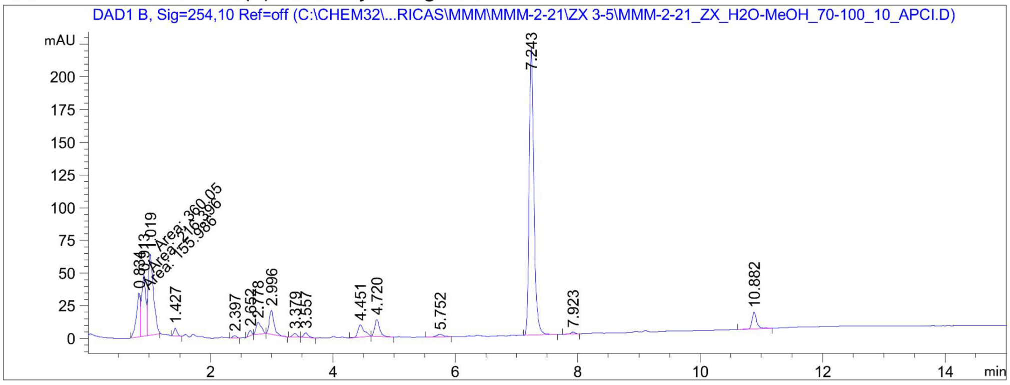

MS Spectrum

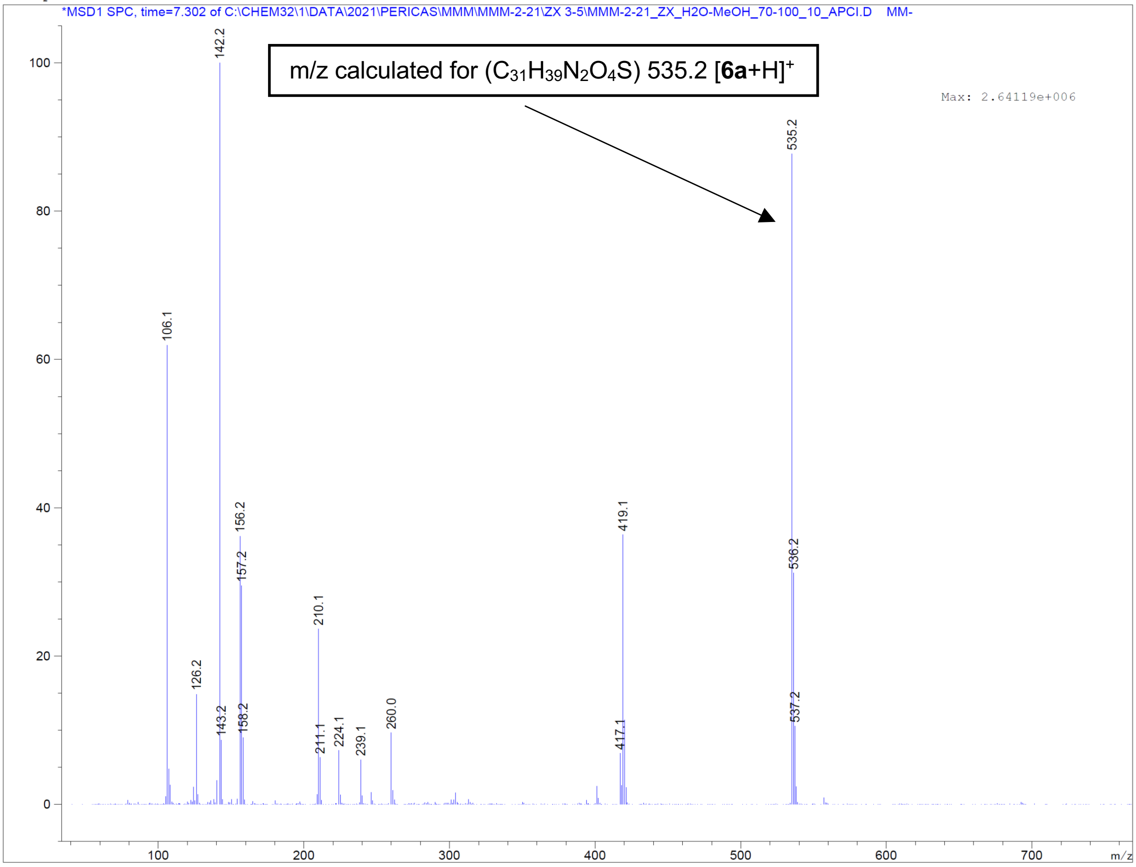

4-Methyl-*N*-(3-oxo-1,3-diphenyl-2-((2,2,6,6-tetramethylpiperidin-1-yl)oxy)propyl) benzenesulfonamide (6a)

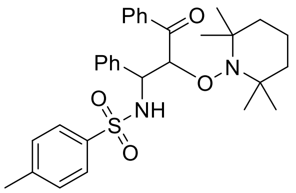

The same protocol has been adapted on 0.2 mmol scale (aziridine **1a,** 76.0 mg, 0.2 mmol, 1 equiv.). The crude mixture was purified by column chromatography on silica gel (Pentane/EtOAc 0% to 30%), providing the isolated compound **6a** as a white amorphous solid (66.0 mg, 62%, 3.6:1 d.r.). **^{1}H NMR** (400 MHz, $CDCl_3$) (*major/minor* 3.6:1 d.r.) δ 7.74 – 7.67 (m, 2H, *minor*), 7.57 – 7.52 (m, 2H, *major*), 7.52 – 7.46 (m, 2H, *major*), 7.46 – 7.37 (m, 1H *major*, 1H *minor*), 7.36 – 7.32 (m, 1H, *minor*), 7.28 – 7.21 (m, 2H *major*, 2H *minor*), 7.20 – 7.14 (m, 1H, *major*), 7.12 (d, *J* = 8.0 Hz, 2H, *major*), 7.08 – 7.02 (m, 2H *major*, 2H *minor*), 7.02 – 6.94 (m, 2H major, 6H minor), 6.62 (d, *J* = 5.7 Hz, 1H, *minor*), 6.54 (d, *J* = 3.0 Hz, 1H, *major*), 5.39 (d, *J* = 4.4 Hz, 1H, *minor*), 5.34 – 5.28 (m, 1H, *major*), 5.23 (t, *J* = 5.1 Hz, 1H, *minor*), 4.86 (dd, *J* = 7.9, 3.0 Hz, 1H, *major*), 2.36 (s, 3H, *major*), 2.34 (s, 3H, *minor*), 1.57 – 1.16 (m, 12H minor, 12H major), 1.06 – 0.94 (m, 3H *major,* 3H *minor*), 0.87 (s, 3H *major,* 3H *minor*). **^{13}C NMR** (101 MHz, $CDCl_3$) (*mixture of rotamers*, *major/minor* 3.6:1 *dr)* δ 202.9, 199.7, 143.1, 142.8, 138.5, 137.9, 137.5, 137.3, 136.9, 135.6, 133.2, 133.0, 129.5, 129.3, 128.8, 128.4, 128.3, 128.2, 128.1, 128.0, 127.7, 127.5, 127.2, 126.9, 85.4, 82.5, 61.3, 60.9, 60.7, 59.9, 59.8, 59.6, 40.8, 40.7, 40.4, 34.3, 34.1, 34.0, 33.8, 21.6, 21.5, 21.0, 20.3, 19.9, 17.1, 17.0. **HRMS** (ESI) m/z calculated for ($C_{31}H_{38}N_2O_4SNa$) 557.2444 $[M+Na]^+$; found 557.2445.

6. X-Ray Structural Analysis for 2a.

X-Ray diffraction: Data sets for compounds **2a_cis** and **2a_trans** were collected with an APEX DUO Kappa 4-axis goniometer Diffractometer. Programs used: data collection: *APEX2* Version 2014.9-0; cell refinement: *SAINT* Version 8.34A; data reduction: *SAINT* Version 8.34A; absorption correction, *SADABS* Version 2014/4; structure solution *SHELXT*-Version 2014-7; structure refinement *SHELXL*- Version 2014-7 and graphics, *XP* (Version 5.1, Bruker AXS Inc., Madison, Wisconsin, USA, **1998**). *R*-values are given for observed reflections, and wR^2 values are given for all reflections.

Exceptions and special features: For compound **2a_trans** one SO_2 unit and one tolyl group were found disordered over two positions in the asymmetric unit. Several restraints (SADI, SAME, ISOR and SIMU) were used in order to improve refinement stability. Moreover, for this compound a badly disordered CH_2Cl_2 molecule was found in the asymmetrical unit and could not be satisfactorily refined. The program SQUEEZE (Spek, A.L. (**2015**). Acta Cryst. C71, 9-18) was therefore used to remove mathematically the effect of the solvent. The quoted formula and derived parameters are not included the squeezed solvent molecule.

X-ray crystal structure analysis of 2a_cis - CCDC number: 2456137:

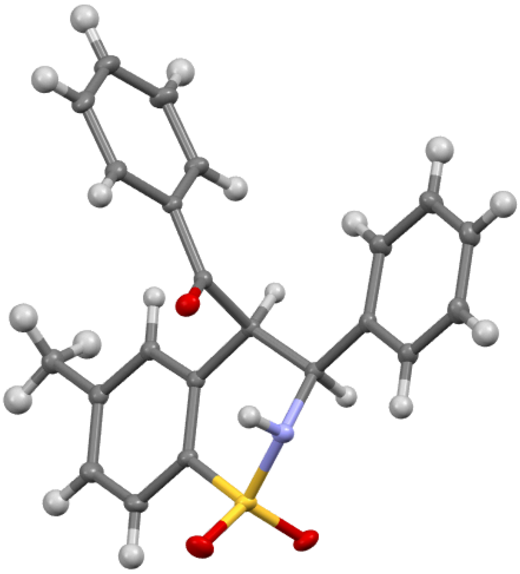

Figure S12. Crystal structure of compound **2a_cis**.
Thermal ellipsoids are shown at 50% probability.

A colorless, block-like specimen of $C_{22}H_{19}NO_3S$, approximate dimensions 0.400 mm x 0.500 mm x 0.500 mm, was used for the X-ray crystallographic analysis. The X-ray intensity data were measured on a single crystal diffractometer Bruker D8 Venture Photon III system equipped with a micro focus tube Mo ImS (MoKα, λ = 0.71073 Å) and a MX mirror monochromator. The integration of the data using a monoclinic unit cell yielded a total of 22742 reflections to a maximum θ angle of 25.35° (0.83 Å resolution), of which 3225 were independent (average redundancy 7.052, completeness = 99.9%, R_{int} = 3.63%, R_{sig} = 2.08%) and 2937 (91.07%) were greater than $2\sigma(F^2)$. The final cell constants of a = 9.1302(4) Å, b = 20.4004(8) Å, c = 9.9268(4) Å, β = 106.5007(10)°, volume = 1772.82(13) Å^3, are based upon the refinement of the XYZ-centroids of reflections above 20 σ(I). Data were corrected for absorption effects using the Multi-Scan method (SADABS). The calculated minimum and maximum transmission coefficients (based on crystal size) are 0.9040 and 0.9220. The structure was solved and refined using the Bruker SHELXTL Software Package,

using the space group $P2_1/n$, with Z = 4 for the formula unit, $C_{22}H_{19}NO_3S$. The final anisotropic full-matrix least-squares refinement on F^2 with 249 variables converged at R1 = 3.82%, for the observed data and wR2 = 9.46% for all data. The goodness-of-fit was 1.030. The largest peak in the final difference electron density synthesis was 1.013 $e^-/Å^3$ and the largest hole was -0.437 $e^-/Å^3$ with an RMS deviation of 0.057 $e^-/Å^3$. On the basis of the final model, the calculated density was 1.414 g/cm^3 and F(000), 792 e^-. The hydrogen atom at NH group was refined freely.

X-ray crystal structure analysis of 2a_trans - CCDC number: 2456138:

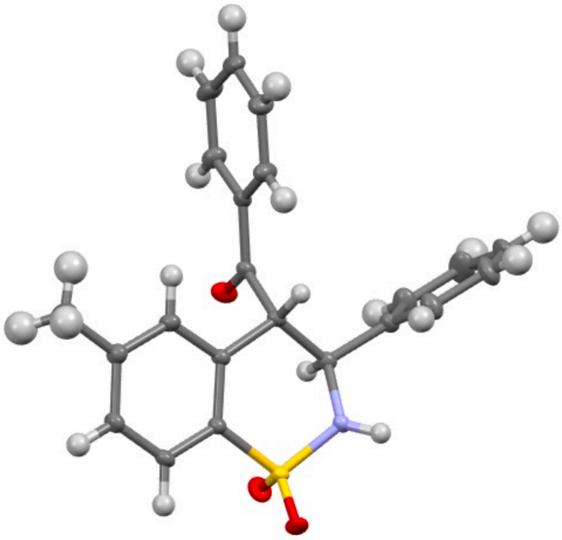

Figure S13: Crystal structure of compound **2a_trans**.
Thermal ellipsoids are shown at 50% probability. Only one independent molecule (molecule named with suffix A) of two found in the asymmetric unit is shown.

A colorless, block-like specimen of $C_{22}H_{19}NO_3S$, approximate dimensions 0.200 mm x 0.200 mm x 0.300 mm, was used for the X-ray crystallographic analysis. The X-ray intensity data were measured on a single crystal diffractometer Bruker D8 Venture Photon III system equipped with a micro focus tube Mo ImS (MoKα, λ = 0.71073 Å) and a MX mirror monochromator. The integration of the data using a triclinic unit cell yielded a total of 25228 reflections to a maximum θ angle of 25.35° (0.83 Å resolution), of which 7274 were independent (average redundancy 3.468, completeness = 98.8%, R_{int} = 2.18%, R_{sig} = 2.08%) and 6416 (88.20%) were greater than $2\sigma(F^2)$. The final cell constants of a = 10.3860(6) Å, b = 10.4561(7) Å, c = 19.9148(12) Å, α = 82.937(2)°, β = 78.805(2)°, γ = 71.377(2)°, volume = 2006.1(2) $Å^3$, are based upon the refinement of the XYZ-centroids of reflections above 20 σ(I). Data were corrected for absorption effects using the Multi-Scan method (SADABS). The calculated minimum and maximum transmission coefficients (based on crystal size) are 0.9470 and 0.9640. The structure was solved and refined using the Bruker SHELXTL Software Package, using the space group *P*-1, with Z = 4 for the formula unit, $C_{22}H_{19}NO_3S$. The final anisotropic full-matrix least-squares refinement on F^2 with 547 variables converged at R1 = 4.22%, for the observed data and wR2 = 10.58% for all data. The goodness-of-fit was 1.058. The largest peak in the final difference electron density synthesis was 0.743 $e^-/Å^3$ and the largest hole was -0.453 $e^-/Å^3$ with an RMS deviation of 0.049 $e^-/Å^3$. On the basis of the final model, the calculated density was 1.250 g/cm^3 and F(000), 792 e^-. The hydrogen atoms at NH groups (N1A and N1B atoms) were refined freely.

7. DFT calculations

Methods

The structures were optimized without geometry constraints using the PBE0 hybrid functional[57,58] and an atom-pairwise dispersion correction (D3).[59,60] A flexible triple zeta basis set (def2-TZVP)[61] was used in all calculations. The nature of the optimized stationary points was proven by the presence of either 0 (minimum) or 1 (transition structure) imaginary vibrational frequency. For the determination of free enthalpy contributions at 298 K (G^{RRHO}), the rigid-rotor-harmonic-oscillator model and a rotor approximation for vibrational modes with wave numbers below 100 cm^{-1} was applied.[62] Electronic energies of the structures optimized with PBE0-D3 were recalculated with the hybrid functional PW6B95(-D3)[63] using the def2-TZVP basis set.
Solvation free energies (G_{solv}) for T = 298.15 K in acetonitrile and DMSO were obtained with COSMO-RS.[64,65] Relative values of free enthalpy in solution ($\Delta G^{s}{}_{298}$) were obtained using the sum of the differences in PW6B95-D3 electronic energies, G^{RRHO}(298K), and G_{solv} as

$$\Delta G_{s,298} = \Delta E(\text{PW6B95-D3}) + \Delta G^{rrho}(298\text{K}) + \Delta G_{solv} \qquad (1)$$

All DFT calculations were performed with the TURBOMOLE program.[66]

Conformer ensembles of *trans*-**2** and *cis*-**2** were obtained with CREST[67,68] and then reoptimized with the DFT procedure as described before. Only one prevailing conformer was obtained within a $\Delta G_{s,298}$ window of 2 kcal/mol. *trans*-**2'** was optimized starting from a conformation resembling the solid state structure of *trans*-**2a**.

DFT calculated energies

Table S2: DFT-calculates energies and thermostatistical contributions of species

Structure	E(PBE0-D3)	$G^{RRHO}{}_{298}$	E(PW6B95-D3)[a]	G_{solv} (CH_3CN) COSMO-RS	G_{solv} (DMSO) COSMO-RS
	[E_h]	[kcal/mol]	[E_h]	[kcal/mol]	[kcal/mol]
**II**	-1495.681493	178.571	-1498.814252	-21.758	-22.810
trans-**TS(II→III)$^-$**	-1488.131392	178.137	-1491.221452	-60.323	-59.600
cis-**TS(II→III)$^-$**	-1488.124711	177.471	-1491.214284	-62.105	-61.368
trans-**TS(II→III)-$LiClO_4$**	-1495.660446	180.951	-1498.791143	-21.520	-22.466
cis-**TS(II→III)-$LiClO_4$**	-1495.662083	180.678	-1498.792601	-22.170	-23.262
trans-**2a**	-1488.157409	182.451	-1491.246489	-17.834	-19.622
trans-**2a'**[b]	-1488.152038	182.680	-1491.240204	-19.369	-20.217
cis-**2a**	-1488.157672	182.457	-1491.245688	-18.684	-19.716

[a] Single point electronic energy using the structures optimized with PBE0-D3.
[b] Conformation as found in the solid state (*trans*-**2a**, Figure S13).

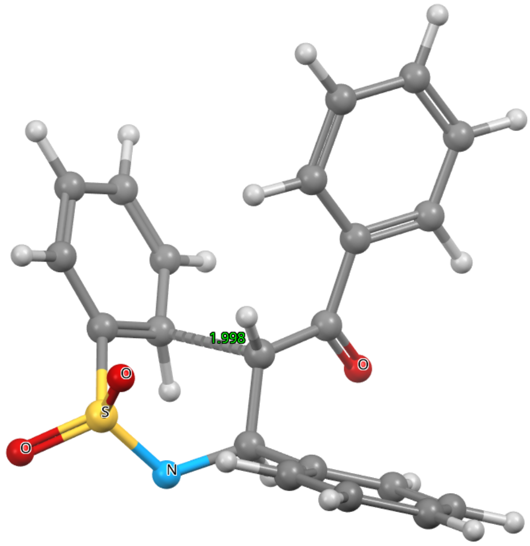

trans-**TS(II→III)**$^-$

$\Delta G^{\ddagger}_{298,s}$(rel) = 0.0 kcal/mol

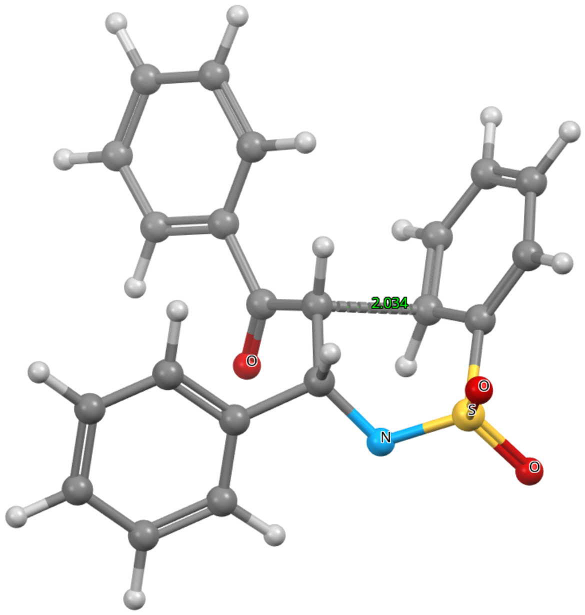

cis-**TS(II→III)**$^-$

$\Delta G^{\ddagger}_{298,s}$(rel) = +2.1 kcal/mol

Figure S14. *Transition states of ring closure of anion* ***II***$^-$ *(**TS(II$^-$→III$^-$)**); R, Ar = Ph)*

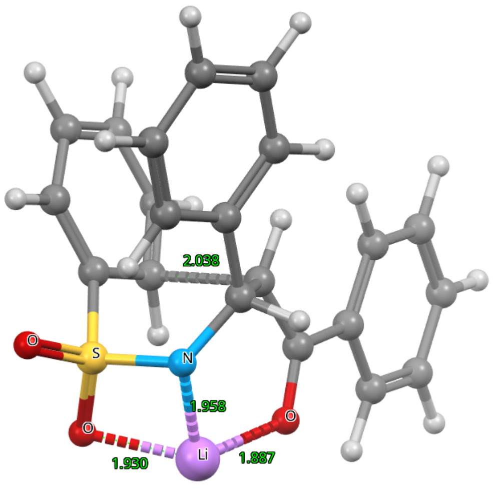

trans-**TS(II→III)-LiClO$_4$**

$\Delta G^{\ddagger}_{298,s}$ = 17.2 kcal/mol

$\Delta G^{\ddagger}_{298,s}$(rel) = 0.0 kcal/mol

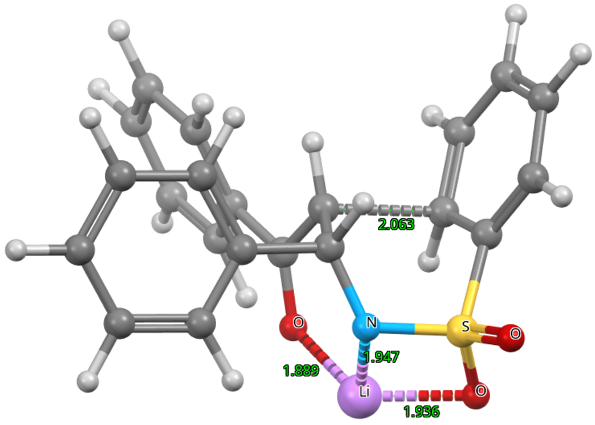

cis-**TS(II→III)-LiClO$_4$**

$\Delta G^{\ddagger}_{298,s}$ = 15.4 kcal/mol

$\Delta G^{\ddagger}_{298,s}$(rel) = -1.8 kcal/mol

Figure S15. *Transition states of ring closure of* ***II*** *(**TS(II→III)-LiClO$_4$** (chelate); R, Ar = Ph)*

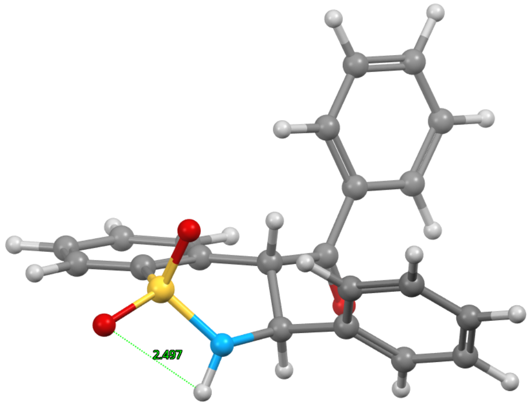

trans-**2a**

$\Delta G^{\ddagger}_{298,s}$(rel) = 0.0 kcal/mol (MeCN, DMSO)

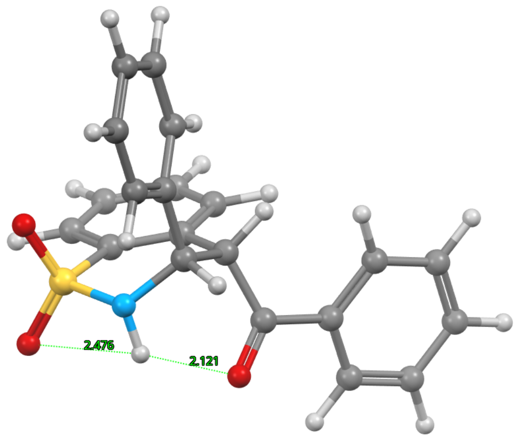

trans-**2a'**
(conformation like *trans*-**2a** in the solid state)

$\Delta G^{\ddagger}_{298,s}$(rel) = +2.6 kcal/mol (MeCN)
$\Delta G^{\ddagger}_{298,s}$(rel) = +3.6 kcal/mol (DMSO)

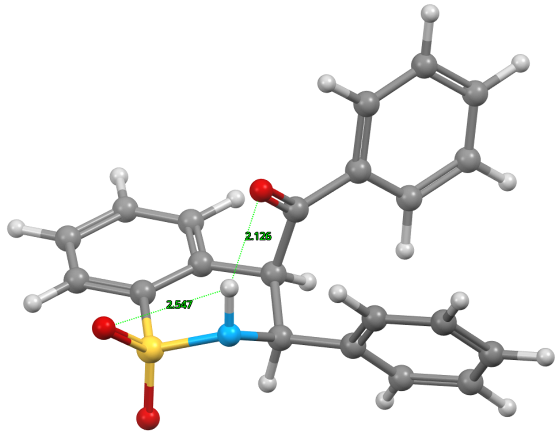

cis-**2a**

$\Delta G^{\ddagger}_{298,s}$(rel) = -0.3 kcal/mol (MeCN)
$\Delta G^{\ddagger}_{298,s}$(rel) = +0.4 kcal/mol (DMSO)

Figure S16. *Reaction Product* ***2a*** *(R, Ar = Ph)*

Cartesian Coordinates of DFT-optimized molecular structures:

II

```
E(PBE0-D3/def2-TZVP) = -1495.681493397 (conv) Lowest Freq. =     5.63 cm^-1
44
II (001c1/opt)
C    -0.5243442   -3.5529190   -2.2809591
C     0.3183387   -3.5947958   -3.3851585
C    -1.2217190   -2.3942766   -1.9860589
C     0.4617345   -2.4775568   -4.1929103
C    -1.0776752   -1.2780403   -2.8015962
C    -0.2356239   -1.3130814   -3.9021764
S    -1.9251947    0.2317743   -2.3959202
N    -1.4091268    0.6816143   -0.9959327
Li   -3.0073300    0.0804789   -0.1911107
C    -0.0588725    0.5874634   -0.5711031
C     0.0334828    0.2659383    0.8802522
C    -0.9973384   -0.1689152    1.7763066
C    -0.6129757   -0.4239025    3.1914614
O    -2.1784594   -0.3875129    1.4430012
C    -1.4140373   -1.2823015    3.9442439
C     0.4886809    0.1795528    3.7976658
C    -1.1083400   -1.5494622    5.2658478
C     0.7839488   -0.0754512    5.1265493
C    -0.0078543   -0.9460432    5.8605442
H    -0.6393570   -4.4272829   -1.6510635
H     0.8628415   -4.5033897   -3.6147683
H    -1.8812402   -2.3574050   -1.1267999
H     1.1176447   -2.5101649   -5.0549597
H    -0.1447394   -0.4275433   -4.5183702
H     0.4677313   -0.2509512   -1.0750416
H     1.0308643    0.3297295    1.2955308
H    -2.2755592   -1.7344844    3.4693022
H     1.1044393    0.8796842    3.2461860
H    -1.7303133   -2.2280265    5.8376339
H     1.6336176    0.4103548    5.5912310
H     0.2294349   -1.1509221    6.8979886
O    -1.7082815    1.1193503   -3.5051251
O    -3.3281162   -0.1193335   -2.1103986
C     0.8058667    1.8245518   -0.8388198
C     0.2159010    3.0265045   -1.1965602
C     2.1893894    1.7506225   -0.7217489
C     2.9755419    2.8688709   -0.9477026
C     1.0027686    4.1469275   -1.4232985
C     2.3811000    4.0741794   -1.2952604
H     2.6615767    0.8051220   -0.4690724
H    -0.8587845    3.0630461   -1.3207004
H     4.0538904    2.7975356   -0.8626545
H     0.5332568    5.0806928   -1.7108339
H     2.9932321    4.9497683   -1.4776403
```

***trans*-TS(II-III)**$^-$

```
E(PBE0-D3/def2-TZVP) = -1488.131391859 (conv) Lowest Freq. = -561.42 cm^-1
43
trans-TS(II-III)- (006TSc1/opt)
C    -0.4233926   -1.5893314   -3.7048780
C    -1.4452665   -2.0343591   -2.8541714
C     0.8705122   -1.4344350   -3.2131679
C    -1.1837085   -2.3156920   -1.5380740
C     1.1580830   -1.7372033   -1.9009354
C     0.1243328   -2.1178725   -0.9934914
S     2.7605124   -1.3964182   -1.1711730
N     2.5604559   -1.6500321    0.3345973
C     1.5073946   -0.9769368    1.0555543
C     0.3020913   -0.5502881    0.2326344
C    -1.0011748   -0.4848836    0.8740493
C    -2.0488834    0.3859123    0.2554711
O    -1.2846630   -1.1480994    1.8686465
C    -1.9387264    0.9176327   -1.0275926
C    -3.2004204    0.6474526    0.9958176
C    -2.9588541    1.6941675   -1.5551491
C    -4.2124750    1.4345588    0.4757562
C    -4.0947835    1.9600200   -0.8049444
H    -0.6406320   -1.3704690   -4.7444811
H    -2.4497191   -2.1650756   -3.2426934
H     1.6684300   -1.0693419   -3.8496910
H    -1.9746474   -2.6678280   -0.8851218
H     0.4183212   -2.7397829   -0.1506961
H     1.1227192   -1.6893221    1.7953974
H     0.5193790    0.2408483   -0.4765491
H    -1.0661385    0.7091783   -1.6330052
H    -3.2724466    0.2132261    1.9857795
H    -2.8637488    2.0920031   -2.5592928
H    -5.0983370    1.6391037    1.0673465
H    -4.8880165    2.5742147   -1.2173829
O     3.6971023   -2.3455264   -1.7288383
O     2.9757804   -0.0088977   -1.5859640
C     1.9519162    0.2443102    1.8522482
C     1.3291302    0.5416391    3.0625887
C     2.9657847    1.0804613    1.3961992
C     3.3409422    2.1922127    2.1359301
C     1.7069011    1.6518950    3.8029984
C     2.7161760    2.4846299    3.3408929
H     3.4473827    0.8455312    0.4542870
H     0.5378417   -0.1095650    3.4190824
H     4.1333964    2.8355501    1.7671042
H     1.2142667    1.8642568    4.7464944
H     3.0171821    3.3525559    3.9184171
```

***cis*-TS(II-III)$^-$**

```
E(PBE0-D3/def2-TZVP) = -1488.124711481 (conv) Lowest Freq. = -534.03 cm^-1
43
cis-TS(II-III)-  (007TSc1/opt)
C     1.3158975    0.0755844   -4.1390239
C     0.0531543   -0.4662331   -3.8669731
C     2.3651496   -0.1214171   -3.2440632
C    -0.1705570   -1.1624235   -2.7040443
C     2.1635026   -0.8415267   -2.0874435
C     0.8598359   -1.2875787   -1.7262678
S     3.4305815   -1.0880488   -0.8467349
N     2.5928160   -1.2104347    0.4487048
C     1.7524480   -0.0683005    0.6769976
C     0.4764642    0.0112641   -0.2087775
C    -0.7950056   -0.5190306    0.2659745
C    -2.0612201    0.1941648   -0.1147899
O    -0.8741029   -1.5360681    0.9415186
C    -2.1552470    1.1136196   -1.1576941
C    -3.2095328   -0.1085660    0.6159520
C    -3.3692162    1.7114981   -1.4630196
C    -4.4166832    0.5009322    0.3243643
C    -4.5016074    1.4133005   -0.7201115
H     1.4799296    0.6320474   -5.0554759
H    -0.7513411   -0.3434856   -4.5848055
H     3.3518709    0.2844728   -3.4352137
H    -1.1488592   -1.5821633   -2.4977360
H     0.8069312   -2.0809764   -0.9843035
H     2.2890701    0.8680380    0.4482857
H     0.4038898    0.9365026   -0.7703968
H    -1.2847733    1.3482339   -1.7566197
H    -3.1183894   -0.8347512    1.4147822
H    -3.4286737    2.4148532   -2.2863990
H    -5.2979645    0.2637254    0.9106134
H    -5.4479323    1.8886718   -0.9544565
O     4.0886126   -2.3391336   -1.1466545
O     4.2554966    0.1060479   -0.9751606
C     1.3616728    0.0343174    2.1377782
C     1.6470523   -0.9892531    3.0293420
C     0.7136426    1.1723753    2.6089074
C     0.3489391    1.2852209    3.9415581
C     1.2846475   -0.8799037    4.3638697
C     0.6329430    0.2545054    4.8270383
H     0.4909207    1.9823135    1.9199081
H     2.1587142   -1.8604677    2.6395122
H    -0.1544591    2.1809647    4.2905406
H     1.5107325   -1.6905557    5.0491596
H     0.3506496    0.3376641    5.8713578
```

***trans*-TS(II-III)-$LiClO_4$**

```
E(PBE0-D3/def2-TZVP) = -1495.660446185 (conv) Lowest Freq. = -525.52 cm^-1
44
trans-TS(II-III) (002TSc1/opt)
C     1.7917973    0.3910311   -2.7215375
C     0.4185566    0.6104149   -2.8729594
C     2.2515886   -0.6184000   -1.8782502
C    -0.4924900   -0.1551119   -2.1884811
C     1.3508351   -1.3916919   -1.1866365
C    -0.0506873   -1.1272388   -1.2405479
S     1.8887545   -2.5267173    0.0487045
N     1.4325711   -1.8643938    1.3902650
Li    0.0410376   -3.2387609    1.4811853
C     0.9292745   -0.5150565    1.4250381
C    -0.3404998   -0.2990364    0.5992176
C    -1.5436654   -1.0182353    0.9713904
C    -2.8718733   -0.4548544    0.6228556
O    -1.4970438   -2.1449214    1.4967579
C    -3.0586373    0.8480572    0.1570601
C    -3.9870356   -1.2838261    0.7639549
C    -4.3260190    1.3053220   -0.1632115
C    -5.2512562   -0.8270705    0.4429786
C    -5.4241561    0.4697650   -0.0230768
H     2.5013135    0.9968630   -3.2713017
H     0.0714493    1.3725356   -3.5610886
H     3.3095170   -0.8109483   -1.7526755
H    -1.5564819   -0.0139485   -2.3380437
H    -0.7025285   -1.9714330   -1.0418836
H     0.5812157   -0.3791262    2.4619878
H    -0.4945532    0.7211335    0.2809447
H    -2.2197894    1.5228485    0.0484614
H    -3.8324781   -2.2914915    1.1278502
H    -4.4569042    2.3201873   -0.5194302
H    -6.1067847   -1.4828590    0.5534945
H    -6.4150219    0.8296602   -0.2746295
O     1.0723441   -3.7517287   -0.0669057
O     3.3000495   -2.7290671   -0.1324001
C     1.9559470    0.5893520    1.2104145
C     1.5699123    1.9257123    1.1299536
C     3.3120438    0.2891345    1.1591819
C     4.2527200    1.2944706    0.9888271
C     2.5078564    2.9313221    0.9600747
C     3.8567447    2.6180255    0.8792007
H     3.6279182   -0.7427614    1.2386151
H     0.5241361    2.2014490    1.2157544
H     5.3044094    1.0357183    0.9407879
H     2.1832810    3.9637267    0.8953580
H     4.5926325    3.4019496    0.7427450
```

***cis*-TS(II-III)- $LiClO_4$**

```
E(PBE0-D3/def2-TZVP) = -1495.662082784 (conv) Lowest Freq. = -516.75 cm^-1
44
cis-TS(II-III) (003TSc1/opt)
C      2.3016555    0.6472021   -3.7279079
C      0.9137012    0.7362821   -3.8630093
C      2.8631219   -0.2755143   -2.8436829
C      0.0875811   -0.0776091   -3.1249124
C      2.0480677   -1.0979980   -2.1046231
C      0.6285368   -0.9658047   -2.1518432
S      2.7181187   -2.1276424   -0.8288919
N      2.0678222   -1.5522527    0.4710379
Li     0.8358705   -3.0586768    0.4212423
C      1.5805497   -0.2034332    0.4209578
C      0.2445199   -0.0945739   -0.3210578
C     -0.8950508   -0.8962259    0.1006174
C     -2.2458428   -0.2895374    0.0705630
O     -0.7900902   -2.0973629    0.4016879
C     -2.4529951    1.0872611    0.1566931
C     -3.3504945   -1.1383066   -0.0188937
C     -3.7379622    1.6033084    0.1443244
C     -4.6317336   -0.6205365   -0.0471155
C     -4.8278594    0.7523866    0.0338759
H      2.9460476    1.2852256   -4.3204097
H      0.4871691    1.4345482   -4.5737545
H      3.9363409   -0.3667593   -2.7273620
H     -0.9878967   -0.0311622   -3.2528604
H      0.0490264   -1.8428616   -1.8839895
H      2.2757253    0.4747283   -0.0942793
H     -0.0022447    0.9146599   -0.6287986
H     -1.6116797    1.7585102    0.2743573
H     -3.1773603   -2.2060023   -0.0693934
H     -3.8895428    2.6729576    0.2269898
H     -5.4827272   -1.2862903   -0.1297930
H     -5.8325752    1.1586339    0.0171049
O      2.1261900   -3.4725559   -0.9613431
O      4.1463525   -2.0634887   -0.9547774
C      1.3521656    0.3765076    1.8027738
C      1.3708795   -0.4209085    2.9383320
C      1.0824365    1.7368109    1.9398877
C      0.8208291    2.2856236    3.1838509
C      1.1097596    0.1274485    4.1860268
C      0.8272431    1.4784287    4.3133723
H      1.0933066    2.3769735    1.0625970
H      1.6224693   -1.4680945    2.8332299
H      0.6206405    3.3471338    3.2746062
H      1.1344838   -0.5058565    5.0655727
H      0.6254445    1.9048235    5.2889976
```

***trans*-2a**

```
E(PBE0-D3/def2-TZVP) = -1488.157409415 (conv) Lowest Freq. =    16.14 cm^-1
43
trans-2 (004conf/c000/opt)
C     4.3912937   -1.8573770   -0.5021961
C     3.2936594   -2.6491918   -0.2016574
C     4.2496508   -0.4796981   -0.5597521
C     2.0506146   -2.0793157    0.0386433
C     3.0009585    0.0723623   -0.3388698
C     1.8797151   -0.7032987   -0.0369006
S     2.7526814    1.8088096   -0.3339459
N     1.8397741    1.9888912    1.0081684
C     0.6612239    1.1434520    1.2277646
C     0.5634017    0.0042862    0.1820762
C    -0.5232502   -0.9730890    0.6087684
C    -1.3918314   -1.5716975   -0.4339084
O    -0.6495870   -1.2676129    1.7762686
C    -2.4939118   -2.3182949   -0.0164708
C    -1.1345550   -1.4350534   -1.7969156
C    -3.3297121   -2.9080966   -0.9450174
C    -1.9656994   -2.0387208   -2.7263732
C    -3.0651412   -2.7698414   -2.3023389
H     5.3590336   -2.3099853   -0.6810781
H     3.4038787   -3.7256106   -0.1436346
H     5.0914770    0.1709046   -0.7623816
H     1.2132793   -2.7185748    0.2911436
H     2.4259838    2.1240620    1.8188720
H     0.7791969    0.6618801    2.2006770
H     0.2767657    0.4754386   -0.7606429
H    -2.6745372   -2.4163583    1.0468565
H    -0.2773350   -0.8713603   -2.1435096
H    -4.1904464   -3.4776710   -0.6150658
H    -1.7562515   -1.9343337   -3.7841360
H    -3.7193760   -3.2338051   -3.0314770
O     3.9905923    2.4816377   -0.0936239
O     1.9441400    2.1397341   -1.4697443
C    -0.6133416    1.9535941    1.2357114
C    -0.8690942    2.8770718    0.2270246
C    -1.5587009    1.7564644    2.2342123
C    -2.7487423    2.4696967    2.2257666
C    -2.0578617    3.5880014    0.2188724
C    -3.0013531    3.3856971    1.2169717
H    -1.3655386    1.0295521    3.0151674
H    -0.1263105    3.0458177   -0.5448221
H    -3.4780871    2.3089780    3.0112753
H    -2.2470365    4.3074673   -0.5694407
H    -3.9296198    3.9451876    1.2096628
```

***trans*-2a'**

```
E(PBE0-D3/def2-TZVP) = -1488.152038236 (conv) Lowest Freq. =    19.22 cm^-1
43
trans-2' (004c2/opt)
C     0.6754647    0.2709408   -3.8068558
C    -0.0735597    1.2229691   -3.1292454
C     1.1445777   -0.8362251   -3.1264668
C    -0.3544811    1.0586917   -1.7849353
C     0.8555392   -0.9983239   -1.7770441
C     0.1027929   -0.0535797   -1.0799791
S     1.5526108   -2.4193671   -0.9883951
N     0.8497476   -2.4260809    0.4870300
C     0.6909670   -1.1329576    1.1628317
C    -0.2500734   -0.2086790    0.3856276
C    -1.6898539   -0.7196218    0.4645931
C    -2.8081306    0.2577548    0.3922000
O    -1.9234312   -1.9058125    0.5478720
C    -2.6879849    1.5884075    0.7878617
C    -4.0410918   -0.2086424   -0.0633767
C    -3.7814645    2.4373574    0.7279645
C    -5.1255782    0.6443803   -0.1433438
C    -4.9976691    1.9692311    0.2542426
H     0.8956614    0.3932190   -4.8603434
H    -0.4410903    2.0991476   -3.6501552
H     1.7364163   -1.5889037   -3.6337273
H    -0.9428703    1.8072098   -1.2675638
H    -0.0506143   -2.8935906    0.4193683
H     0.1896818   -1.3822842    2.1028761
H    -0.2094100    0.7806374    0.8421181
H    -1.7489965    1.9695020    1.1700503
H    -4.1227361   -1.2482011   -0.3557123
H    -3.6832977    3.4662238    1.0529249
H    -6.0755651    0.2778371   -0.5136380
H    -5.8494851    2.6369689    0.1980095
O     1.0934317   -3.5961916   -1.6609115
O     2.9524863   -2.1695218   -0.8546742
C     2.0240449   -0.5182577    1.5265295
C     2.4543889    0.7099836    1.0460295
C     2.8474626   -1.2241581    2.3987726
C     4.0716555   -0.7119109    2.7855821
C     3.6836089    1.2272043    1.4330433
C     4.4941620    0.5201883    2.3036458
H     2.5261176   -2.1955527    2.7579730
H     1.8506320    1.2703021    0.3425538
H     4.7022292   -1.2765838    3.4623142
H     4.0085855    2.1841806    1.0417265
H     5.4551191    0.9221093    2.6026272
```

***cis*-2a**

```
E(PBE0-D3/def2-TZVP) = -1488.157672265 (conv) Lowest Freq. =    15.76 cm^-1
43
cis-2 (005conf/c000/opt)
C     4.5700695   -2.1383935   -0.0263912
C     3.4099815   -2.8759074   -0.2215297
C     4.5017603   -0.7591192    0.0302844
C     2.1919614   -2.2330772   -0.3515413
C     3.2745978   -0.1214013   -0.1010355
C     2.0994425   -0.8447824   -0.2934207
S     3.2692885    1.6474738   -0.0802880
N     1.6881799    2.0071811    0.1571709
C     0.8111609    1.2991834   -0.7666035
C     0.7373966   -0.2025935   -0.4010239
C     0.0043875   -0.3613636    0.9312250
C    -1.3076490   -1.0496425    0.9864152
O     0.5108663    0.0860559    1.9399373
C    -2.1004096   -1.2715889   -0.1375751
C    -1.7709226   -1.4533728    2.2396846
C    -3.3373464   -1.8819537   -0.0091945
C    -2.9957586   -2.0800664    2.3633595
C    -3.7824733   -2.2932577    1.2377882
H     5.5250938   -2.6377995    0.0823433
H     3.4547763   -3.9574538   -0.2703196
H     5.3939901   -0.1646782    0.1867984
H     1.2876784   -2.8147682   -0.4989052
H     1.4612658    1.8064295    1.1296986
H     1.2774336    1.3735852   -1.7524315
H     0.1888880   -0.7149514   -1.1908332
H    -1.7794696   -0.9355096   -1.1147001
H    -1.1468234   -1.2632668    3.1040729
H    -3.9558444   -2.0342113   -0.8855198
H    -3.3437492   -2.4013271    3.3379309
H    -4.7466230   -2.7789211    1.3348683
O     3.9898666    2.1066189    1.0642694
O     3.6301674    2.0853572   -1.3939723
C    -0.5659303    1.9004846   -0.8533572
C    -1.1660883    2.5408921    0.2230802
C    -1.2901252    1.7384761   -2.0301999
C    -2.5962060    2.1901135   -2.1265786
C    -2.4734088    2.9946272    0.1284267
C    -3.1939086    2.8155726   -1.0420437
H    -0.8204559    1.2591195   -2.8840198
H    -0.6159769    2.6920257    1.1436682
H    -3.1446937    2.0623877   -3.0525838
H    -2.9296841    3.4918966    0.9762493
H    -4.2147058    3.1719267   -1.1132029
```

8. References

[50] Y. Matano, M. Yoshimune, H. Suzuki, *J. Org. Chem.* **1995**, *60*, 4663-4665.

[51] F. Zhang, Y. Zhang, Q. Tan, L. Lin, X. Liu, X. Feng, *Org. Lett.* **2019**, *21*, 5928-5932.

[52] L. Ma, P. Jiao, Q. Zhang, J. Xu, *Tetrahedron: Asymmetry* **2005**, *16*, 3718-3734.

[53] Y. Cai, X. Liu, J. Li, W. Chen, W. Wang, L. Lin, X. Feng, *Chem. Eur. J.* **2011**, *17*, 14916-14921.

[54] T. Hashimoto, K. Takino, K. Hato, K. Maruoka, *Angew. Chem. Int. Ed.* **2016**, *55*, 8081-8085.

[55] A. R. White, L. Wang, D. A. Nicewicz, *Synlett*. **2019**, *30*, 7,827-832.

[56] T. Jin, N. Sinha, D. S. Wagner, A. Prescimone, D. Häussinger, O. S. Wenger, *J. Am. Chem. Soc.* **2025**,*147*, 4587-4594.

[57] J. P. Perdew, M. Ernzerhof, K. Burke, *J. Chem. Phys.* **1996**, *105*, 9982-9985.

[58] C. Adamo, V. Barone, *J. Chem. Phy.* **1999**, *110*, 6158-6170.

[59] S. Grimme, J. Antony, S. Ehrlich, H. Krieg, *J. Chem. Phys.* **2010**, *132*, 154104.

[60] S. Grimme, S. Ehrlich, L. Goerigk, *J. Comput. Chem.* **2011**, *32*, 1456-1465.

[61] F. Weigend, R. Ahlrichs, *Phys. Chem. Chem. Phys.* **2005**, *7*, 3297-3305.

[62] S. Grimme, *Chem. Eur. J.* **2012**, *18*, 9955-9964.

[63] Y. Zhao, D. G. Truhlar, *J. Phys. Chem. A* **2005**, *109*, 5656-5667.

[64] A. Klamt, *J. Phys. Chem.* **1999**, *99*, 2224-2235.

[65] A. Klamt, V. Jonas, T. Bürger, J. C. Lohrenz, *J. Phys. Chem. A* **1998**, *102*, 5074-5085.

[66] TURBOMOLE V7.9 (2025), a development of University of Karlsruhe and Forschungszentrum Karlsruhe GmbH, 1989-2007, TURBOMOLE GmbH, since 2007; available from http://www.turbomole.com.

[67] S. Grimme, *J. Chem. Theory Comput.* **2019**, *115*, 2847-2862.

[68] P. Pracht, F. Bohle, S. Grimme, *Phys. Chem. Chem. Phys.* **2020**, *22*, 7169-7192.

8. NMR spectra

Phenyl(3-phenyl-1-tosylaziridin-2-yl)methanone (1a)

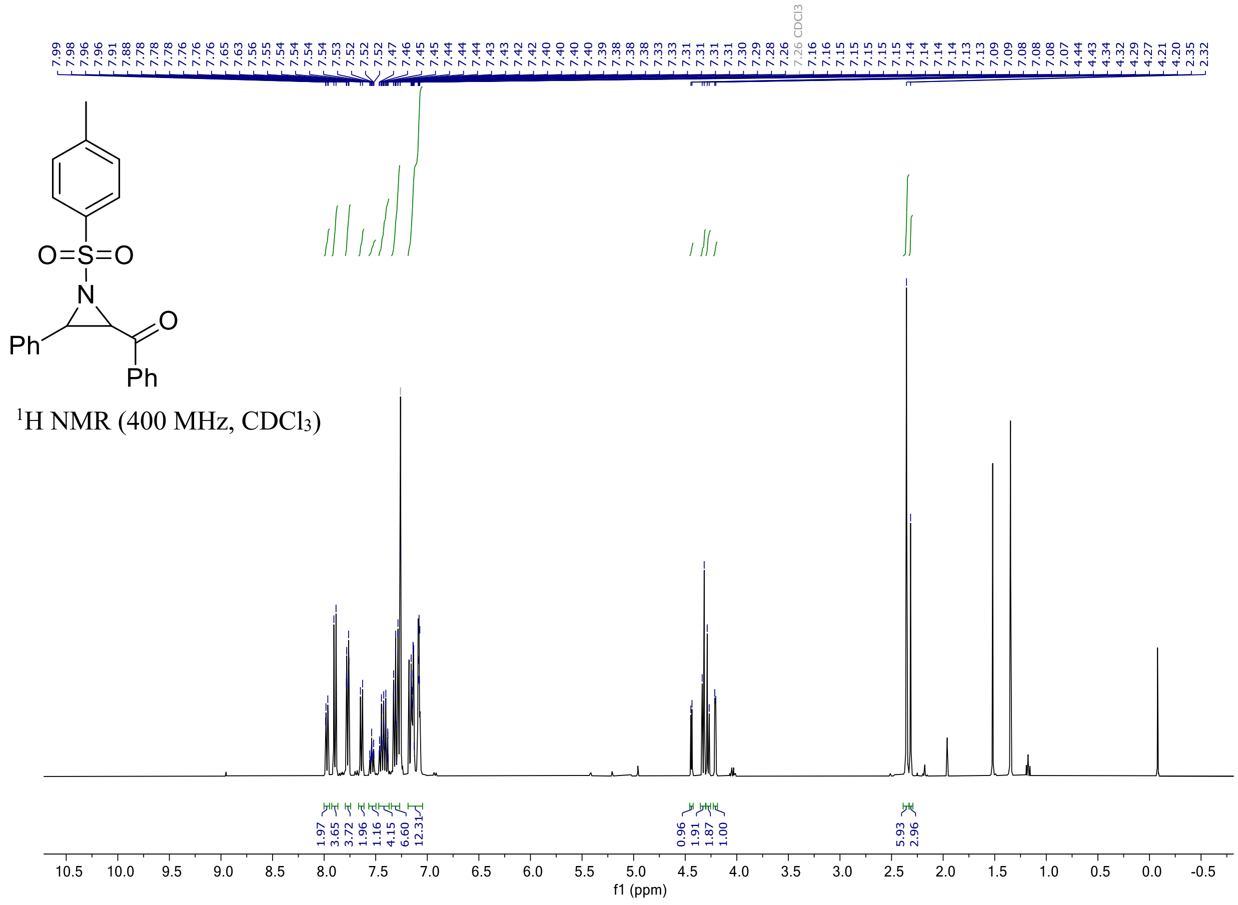

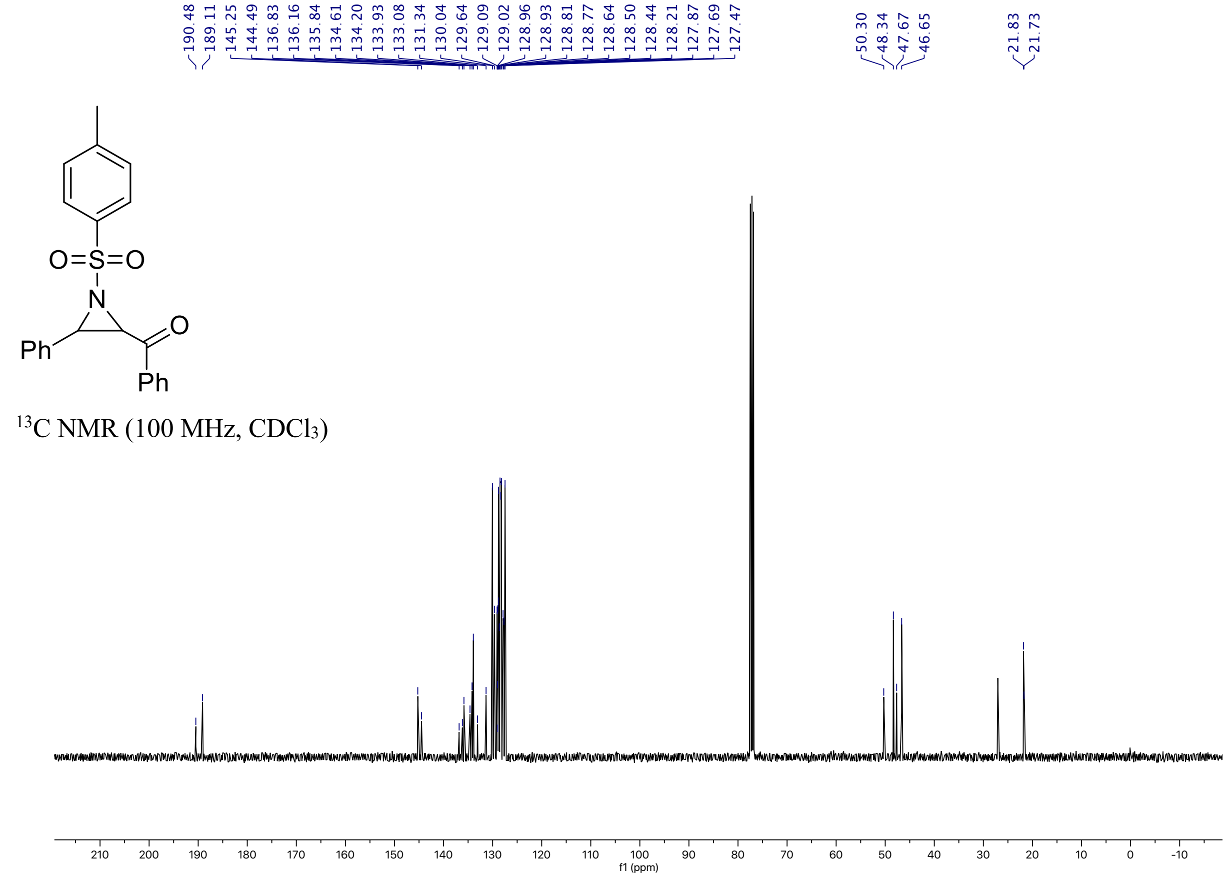

Phenyl(3-phenyl-1-(phenylsulfonyl)aziridin-2-yl)methanone (1b)

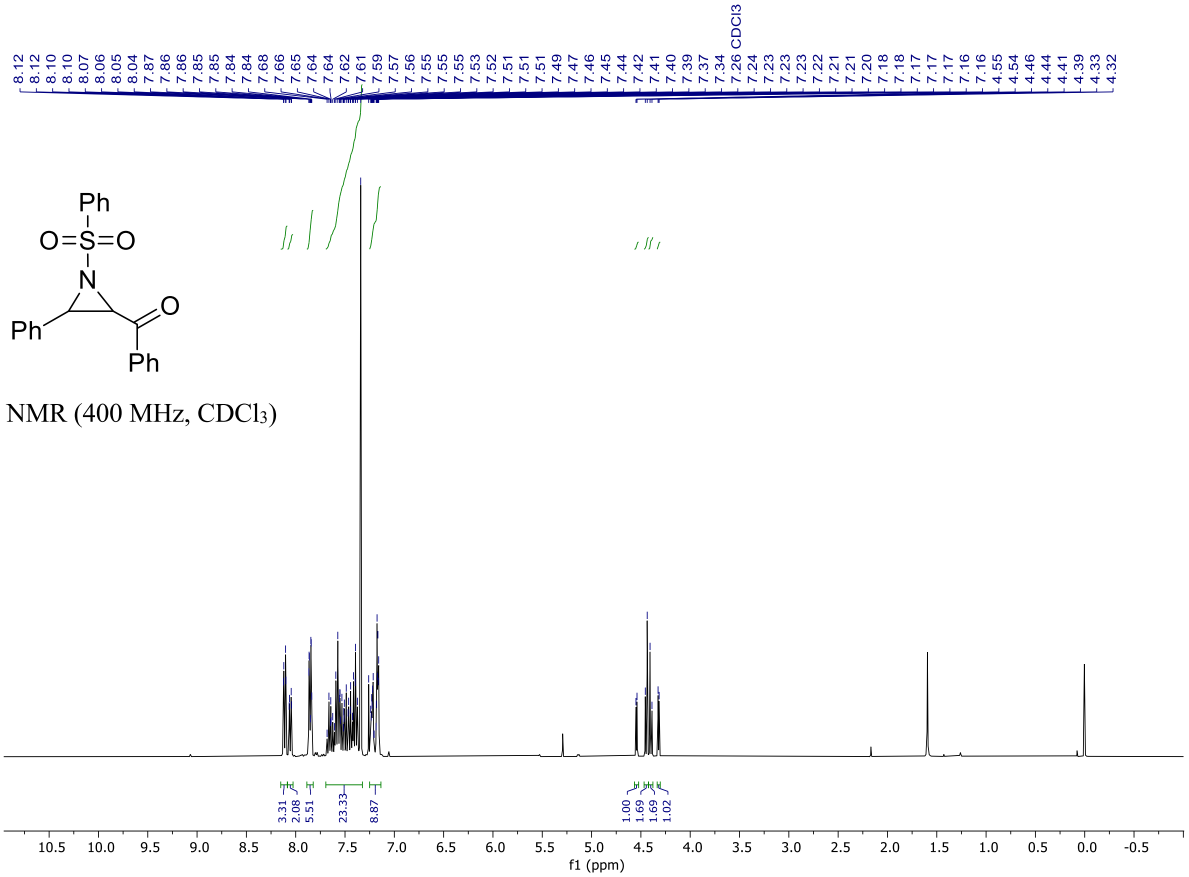

^{1}H NMR (400 MHz, $CDCl_3$)

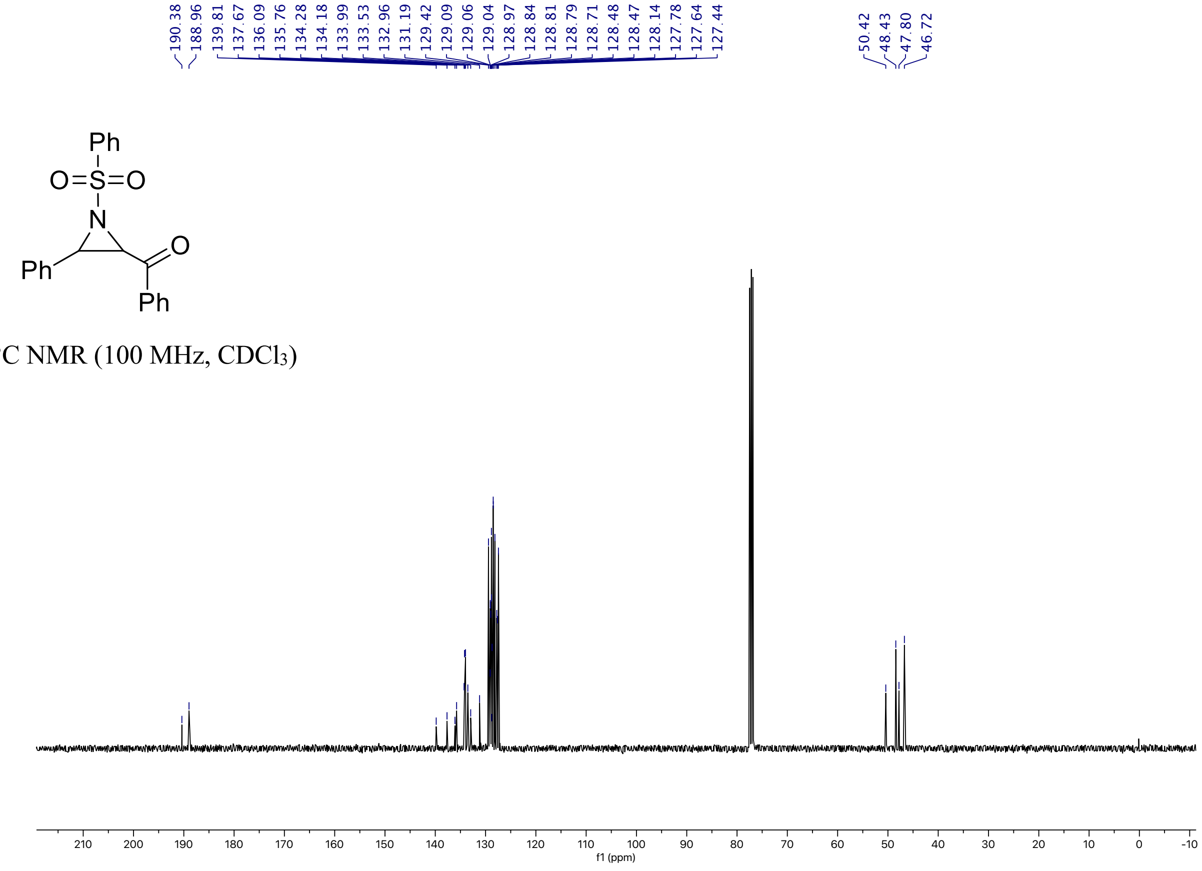

^{13}C NMR (100 MHz, $CDCl_3$)

Phenyl(3-phenyl-1-(*o*-tolylsulfonyl)aziridin-2-yl)methanone (1c)

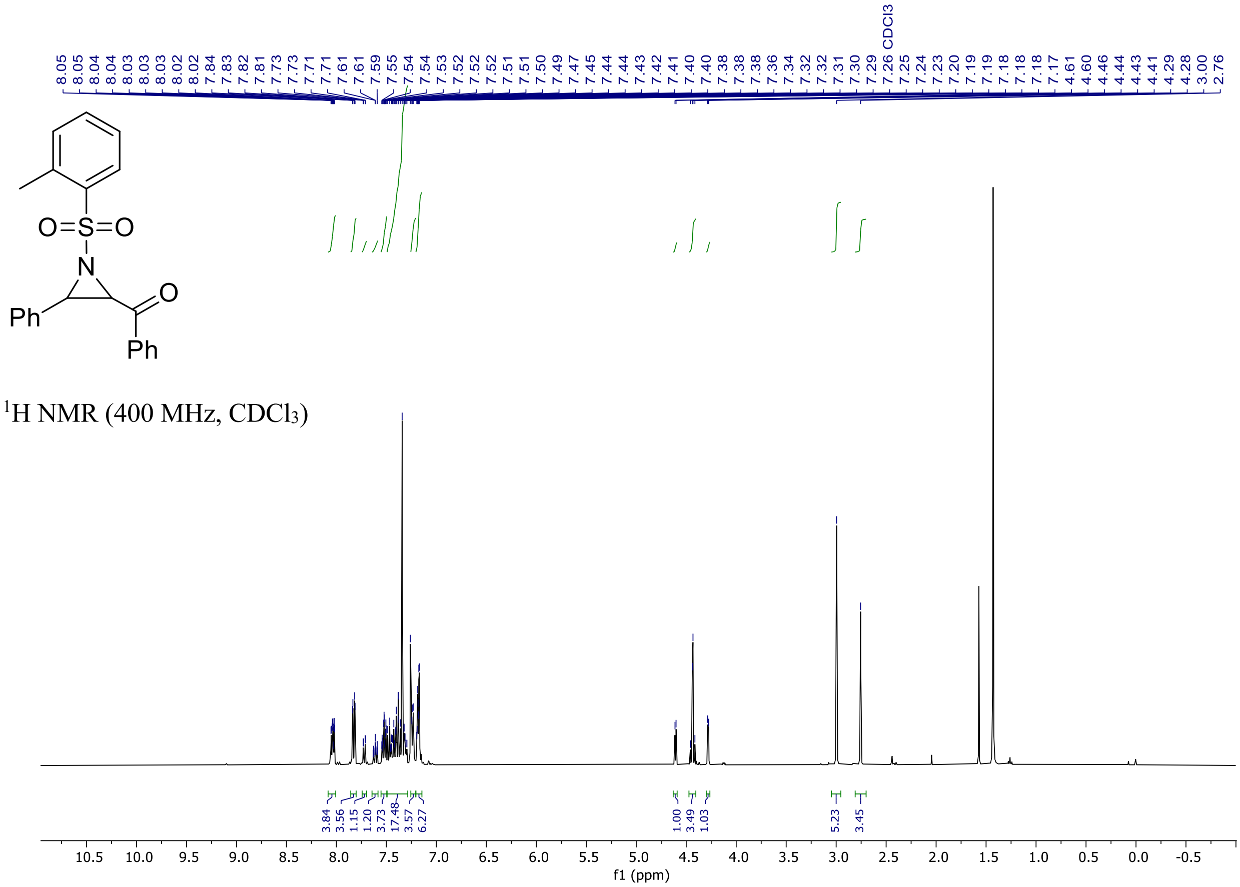

^{1}H NMR (400 MHz, $CDCl_3$)

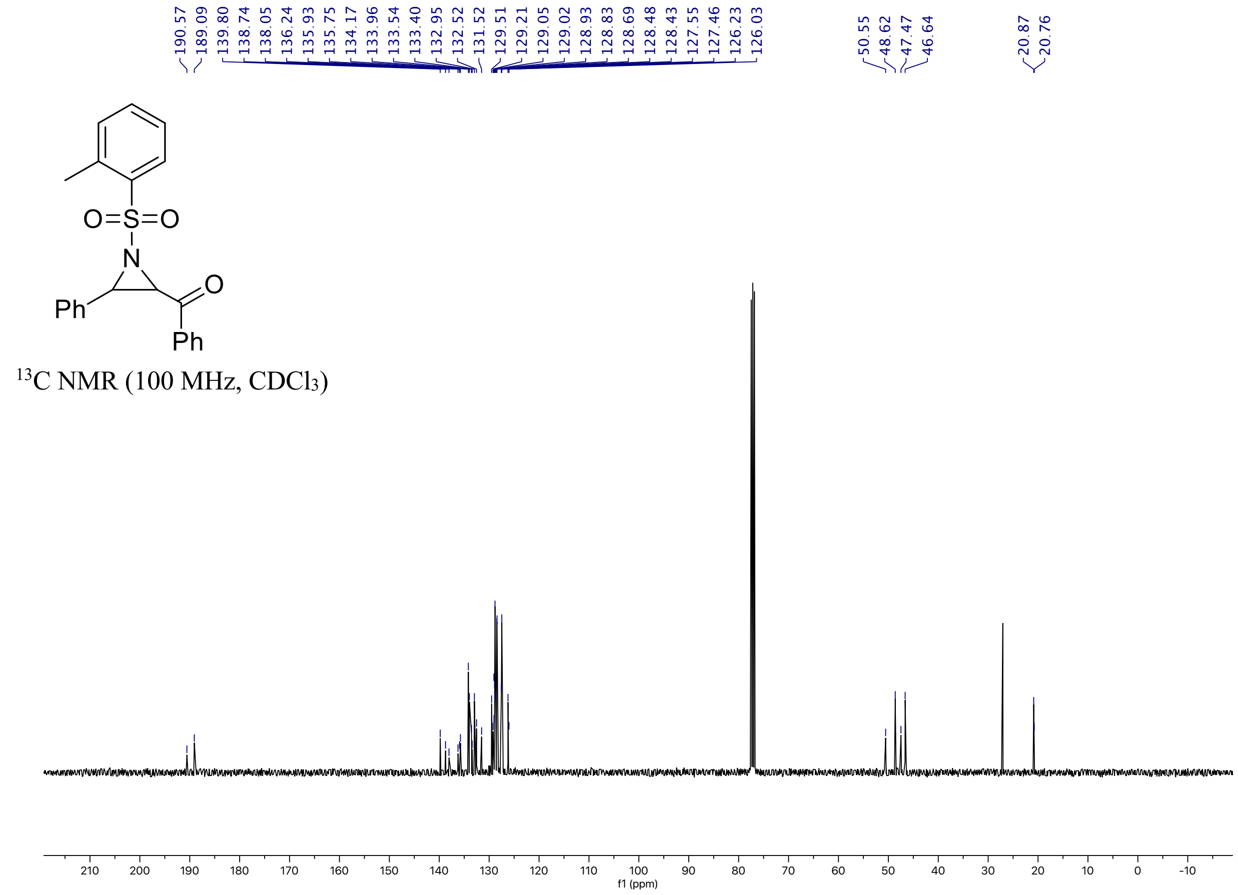

^{13}C NMR (100 MHz, $CDCl_3$)

(1-((4-Methoxyphenyl)sulfonyl)-3-phenylaziridin-2-yl)(phenyl)methanone (1d)

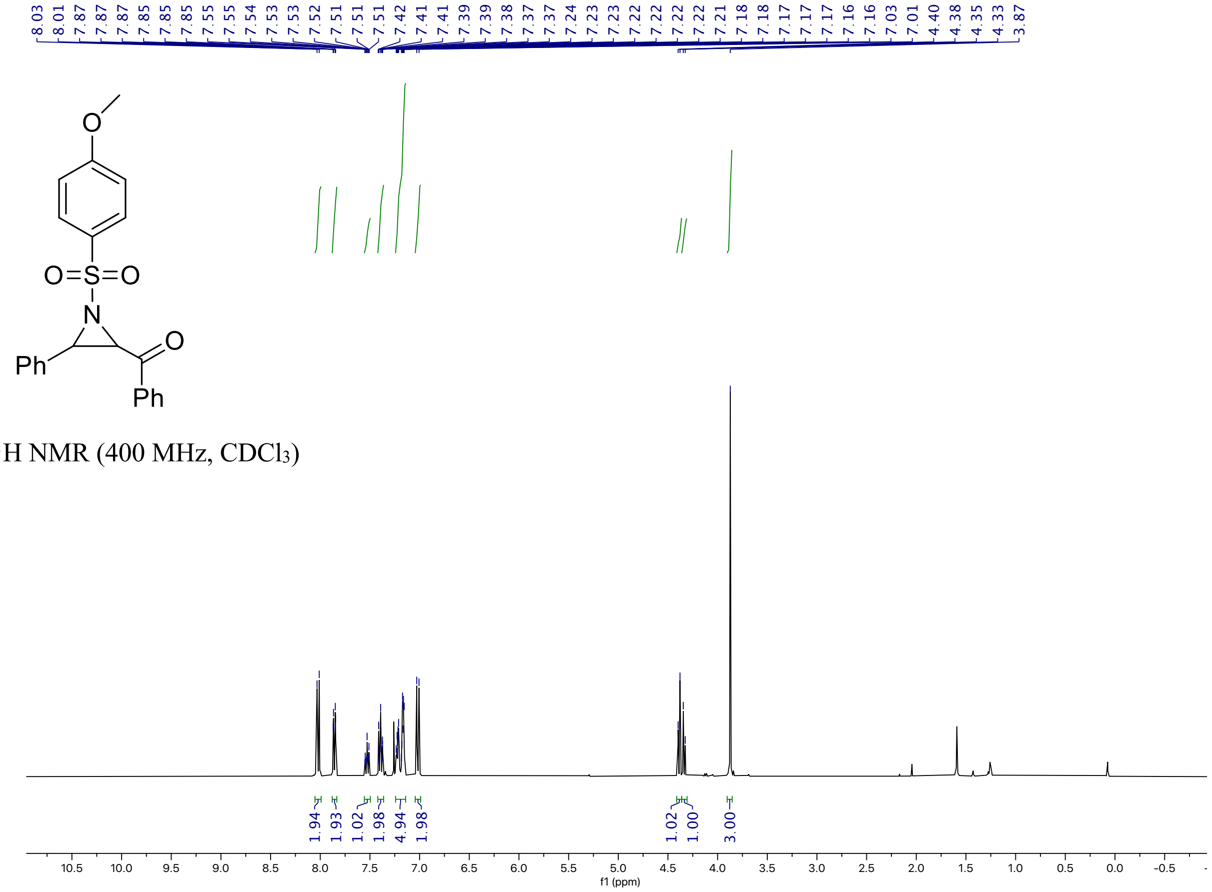

^{1}H NMR (400 MHz, $CDCl_3$)

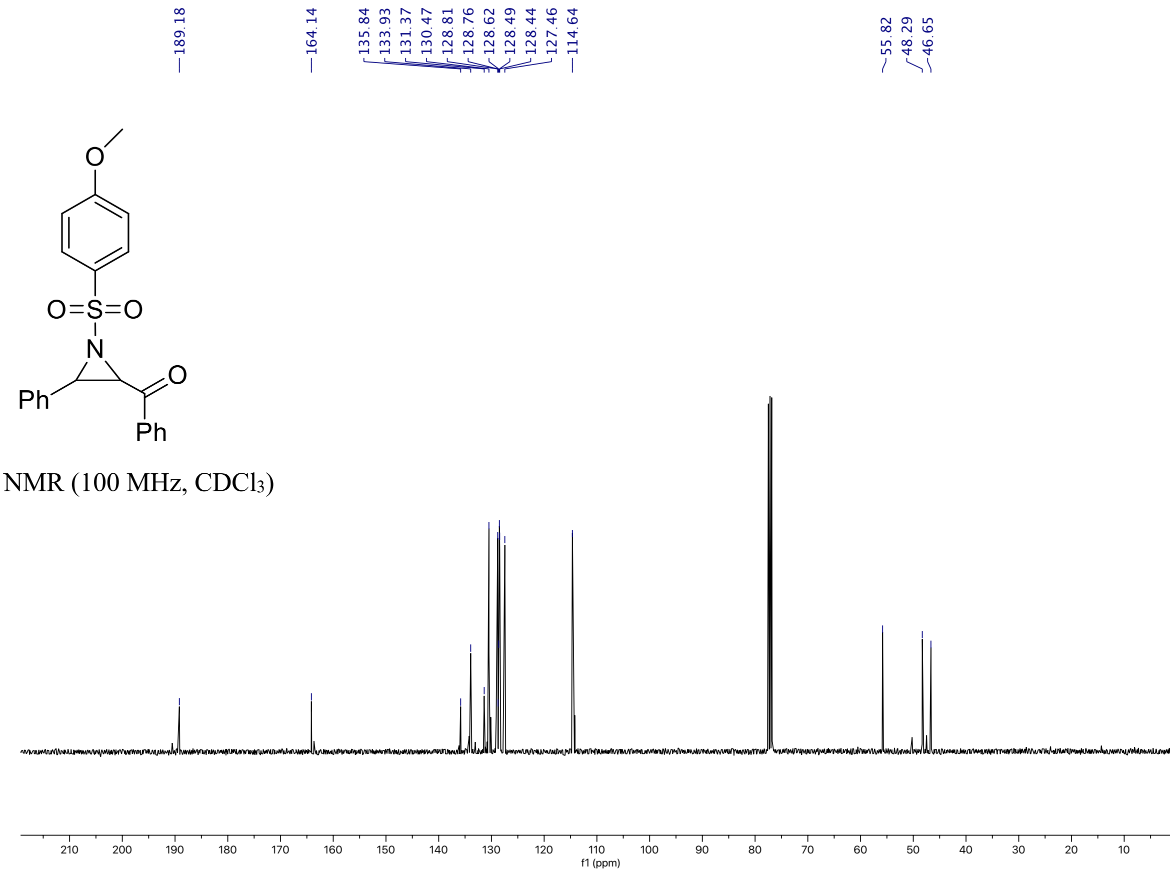

^{13}C NMR (100 MHz, $CDCl_3$)

(1-((4-Chlorophenyl)sulfonyl)-3-phenylaziridin-2-yl)(phenyl)methanone (1e)

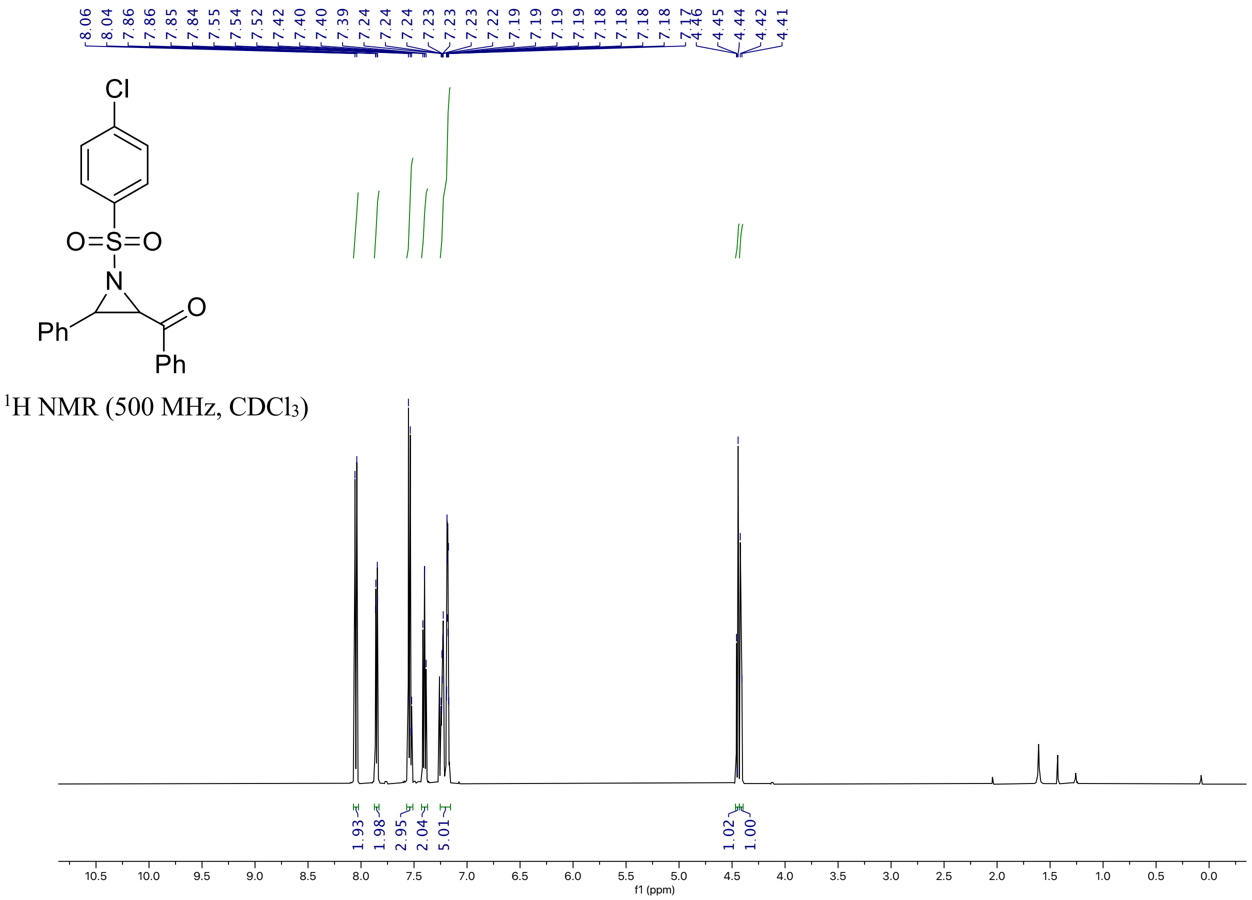

^{1}H NMR (500 MHz, $CDCl_3$)

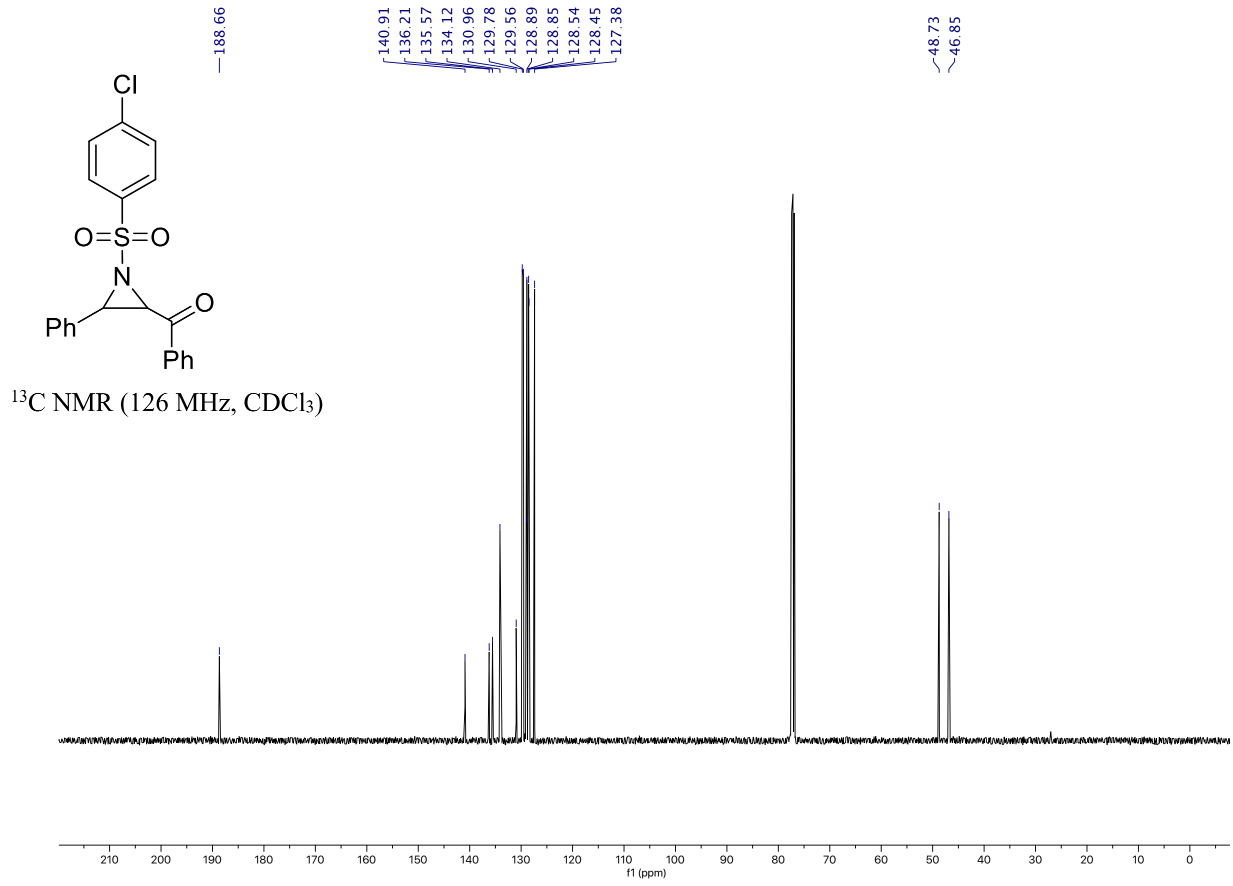

^{13}C NMR (126 MHz, $CDCl_3$)

(1-((4-Bromophenyl)sulfonyl)-3-phenylaziridin-2-yl)(phenyl)methanone (1f)

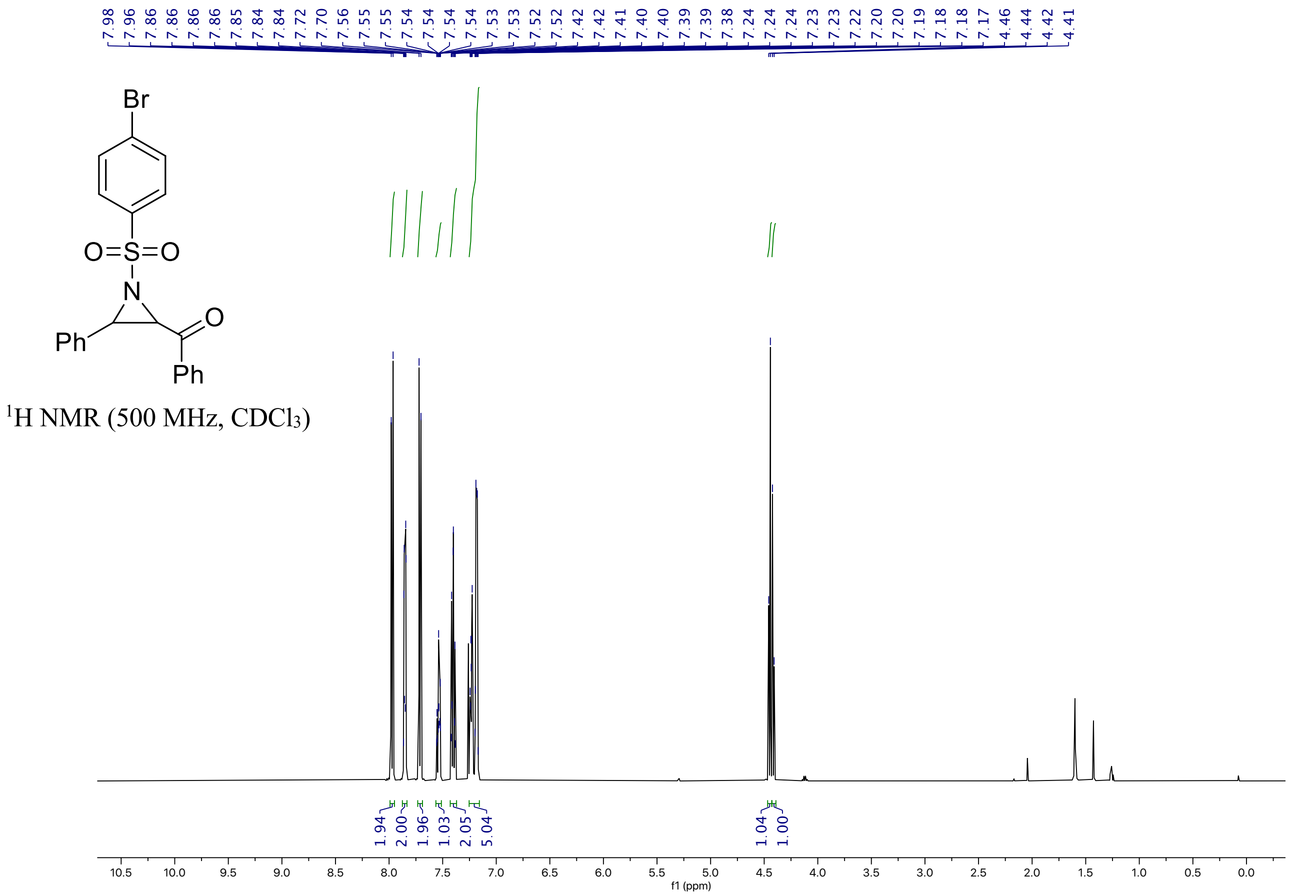

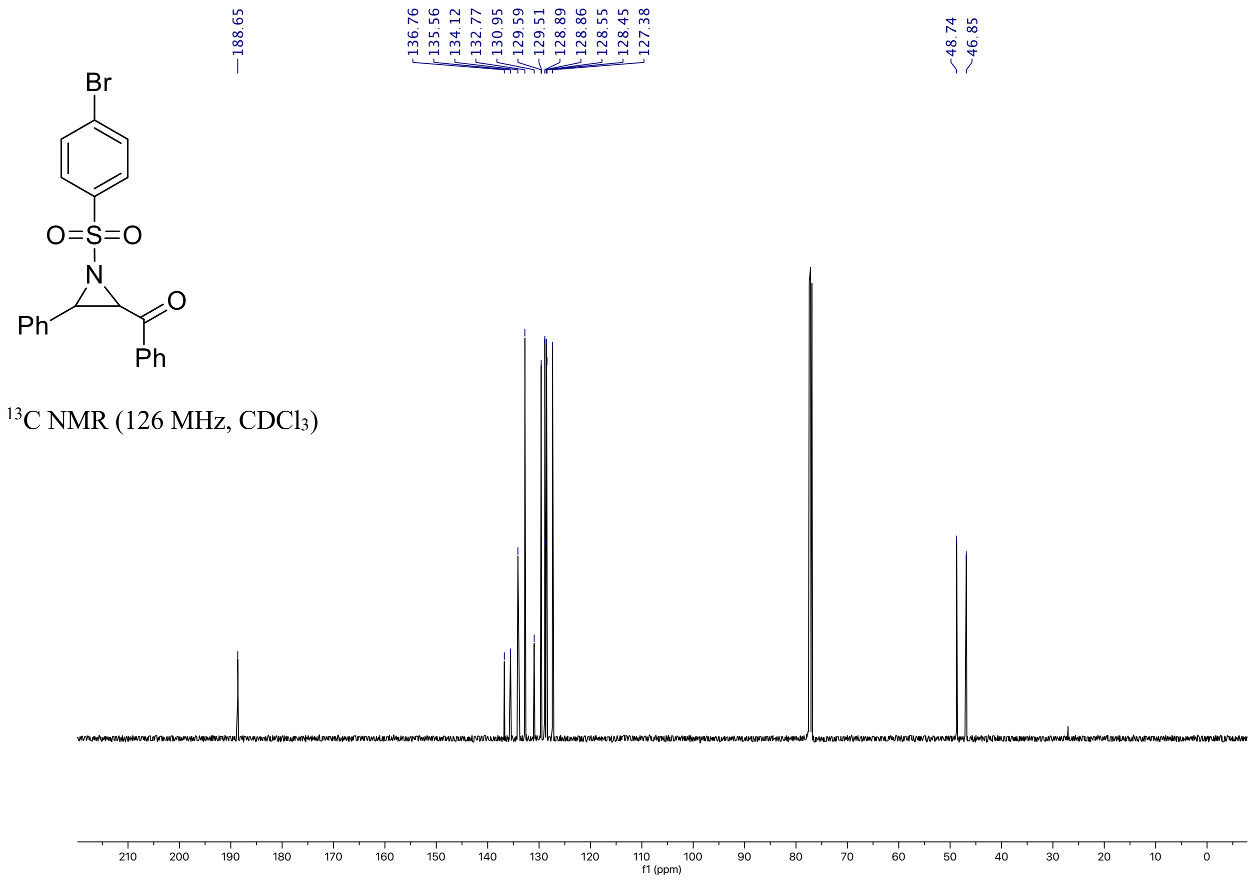

(1-((4-Nitrophenyl)sulfonyl)-3-phenylaziridin-2-yl)(phenyl)methanone (1g)

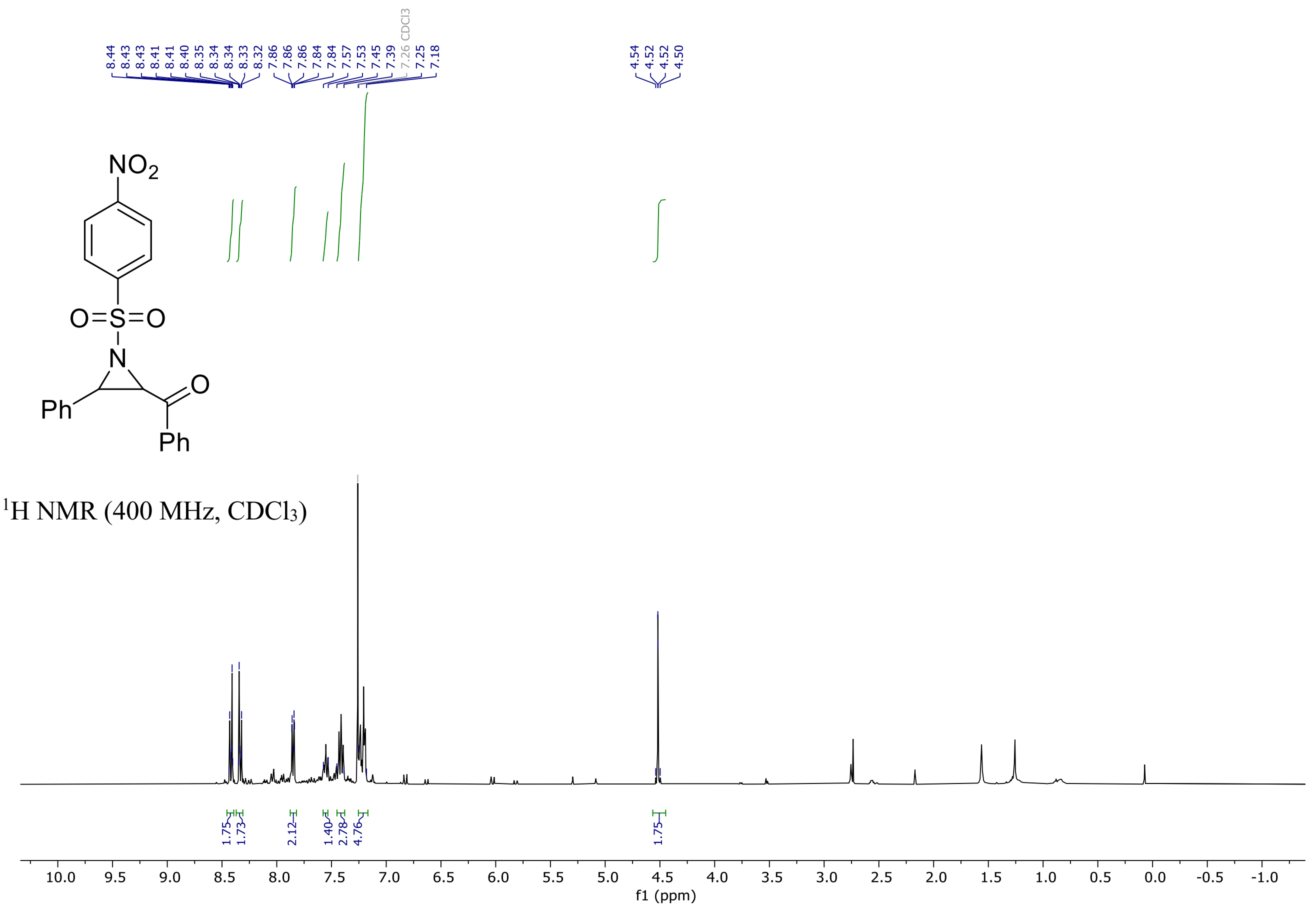

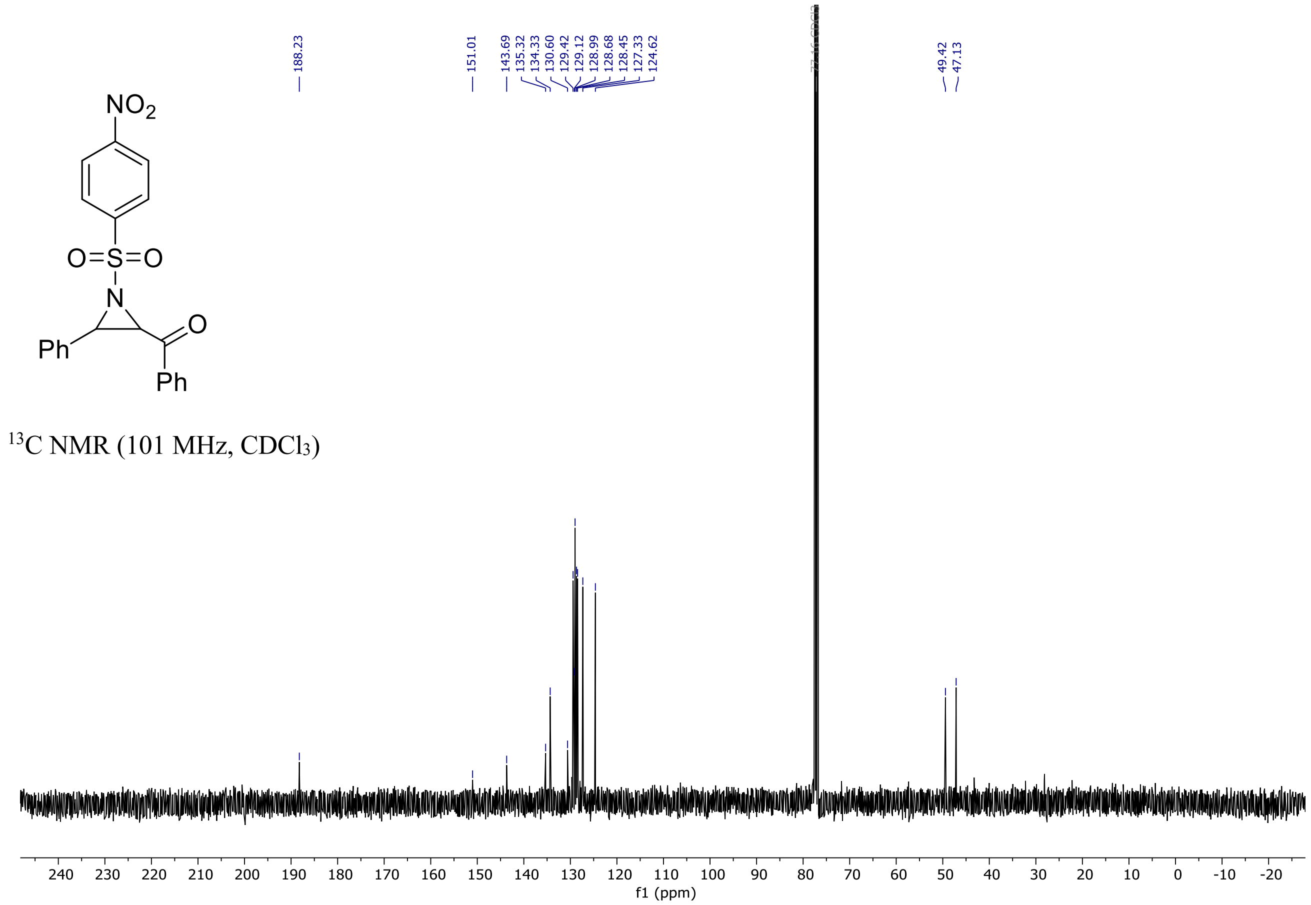

(1-([1,1'-Biphenyl]-4-ylsulfonyl)-3-phenylaziridin-2-yl)(phenyl)methanone (1h)

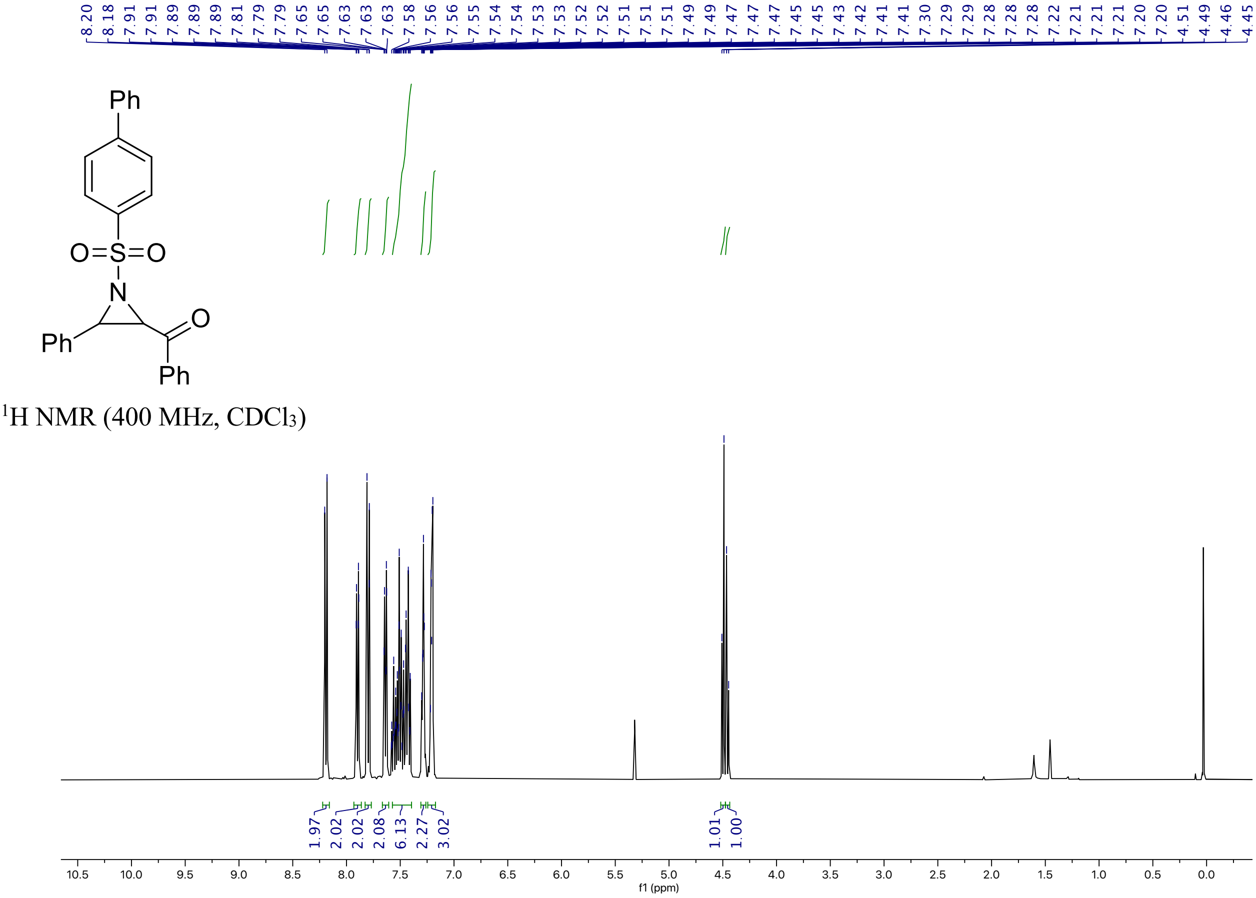

^{1}H NMR (400 MHz, $CDCl_3$)

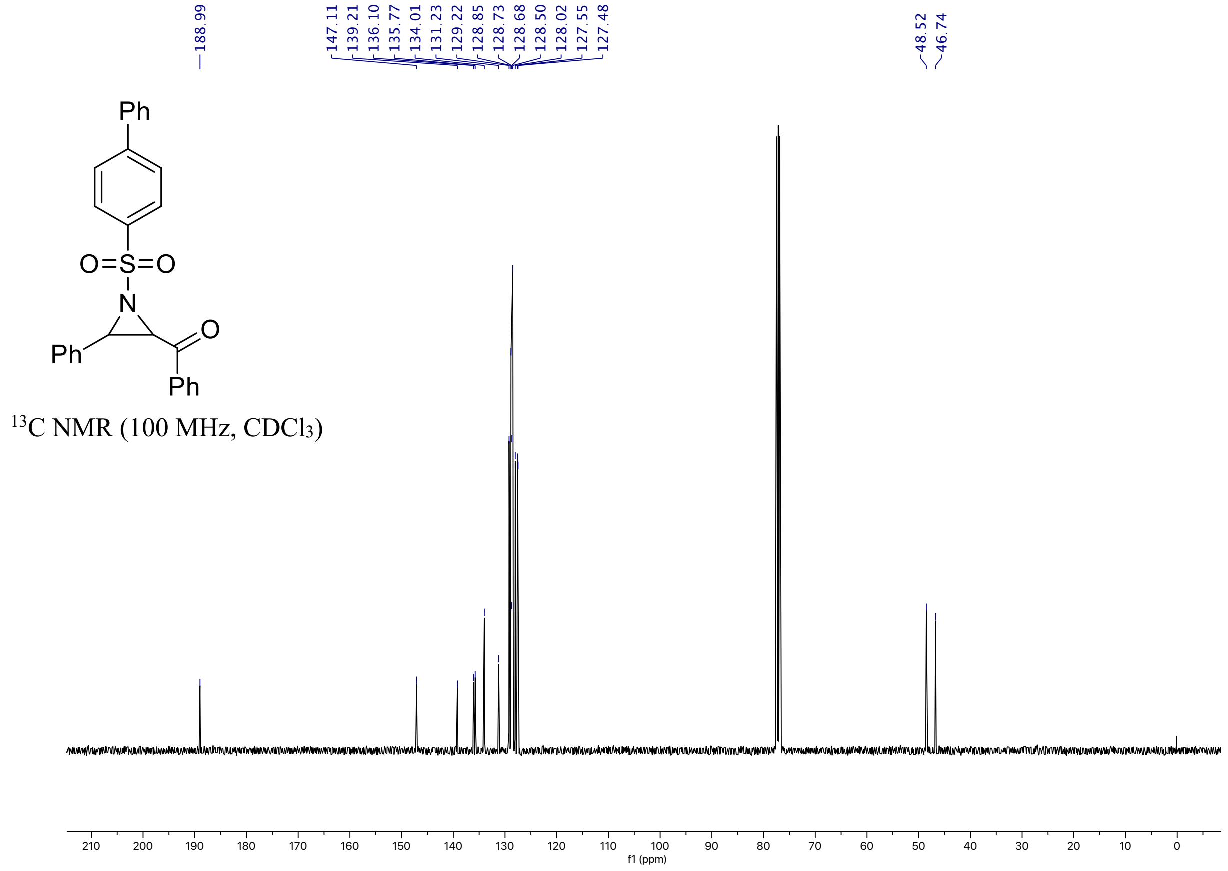

^{13}C NMR (100 MHz, $CDCl_3$)

(3-Phenyl-1-tosylaziridin-2-yl)(*p*-tolyl)methanone (1i)

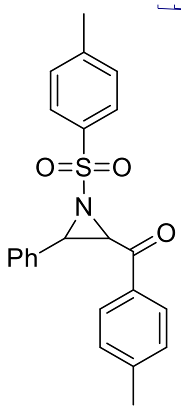

^{1}H NMR (500 MHz, $CDCl_3$)

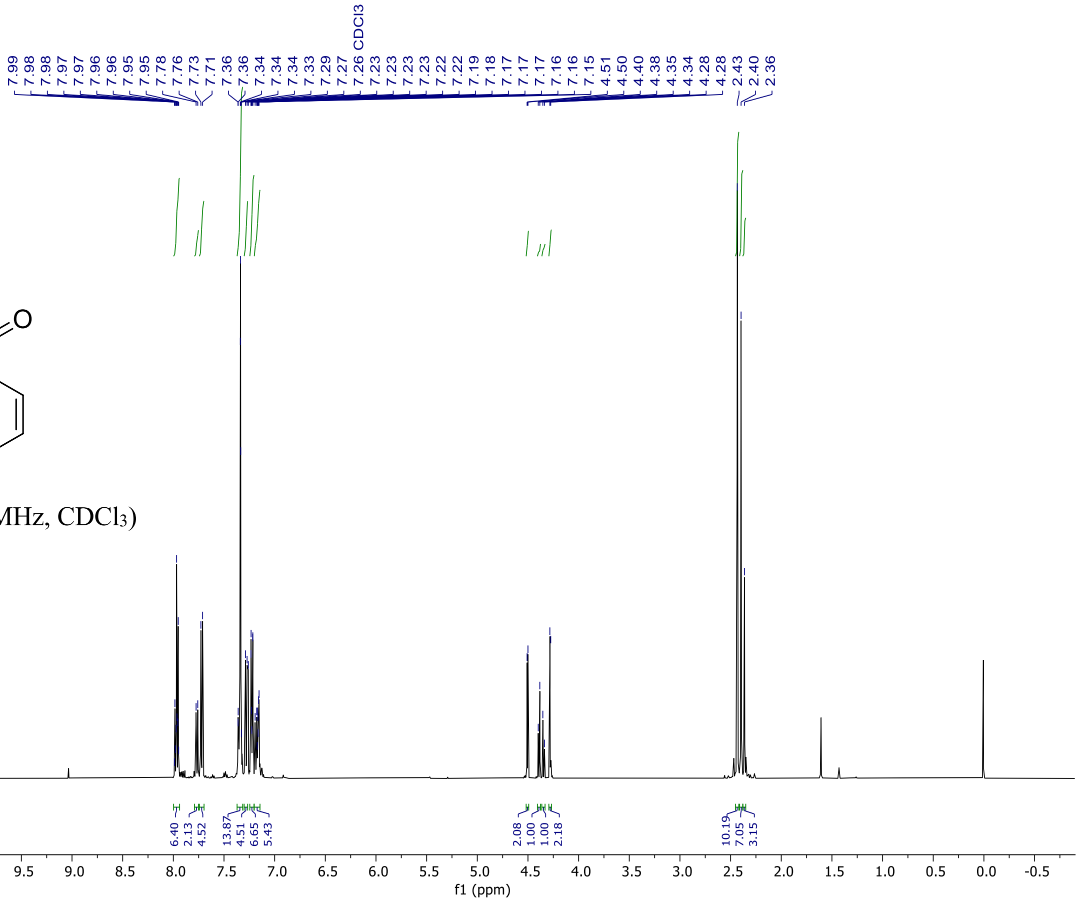

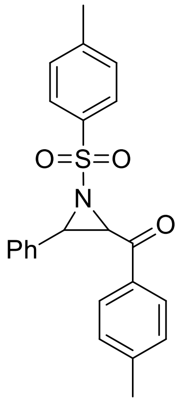

^{13}C NMR (126 MHz, $CDCl_3$)

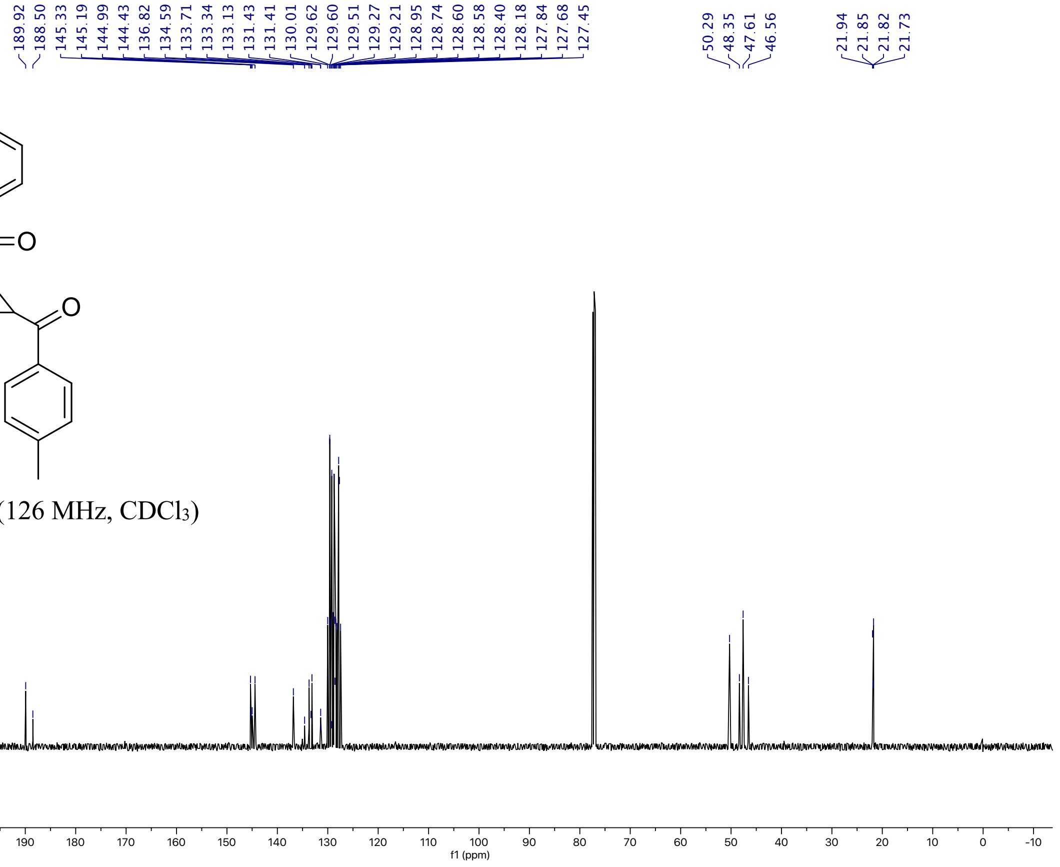

(4-Methoxyphenyl)(3-phenyl-1-tosylaziridin-2-yl)methanone (1j)

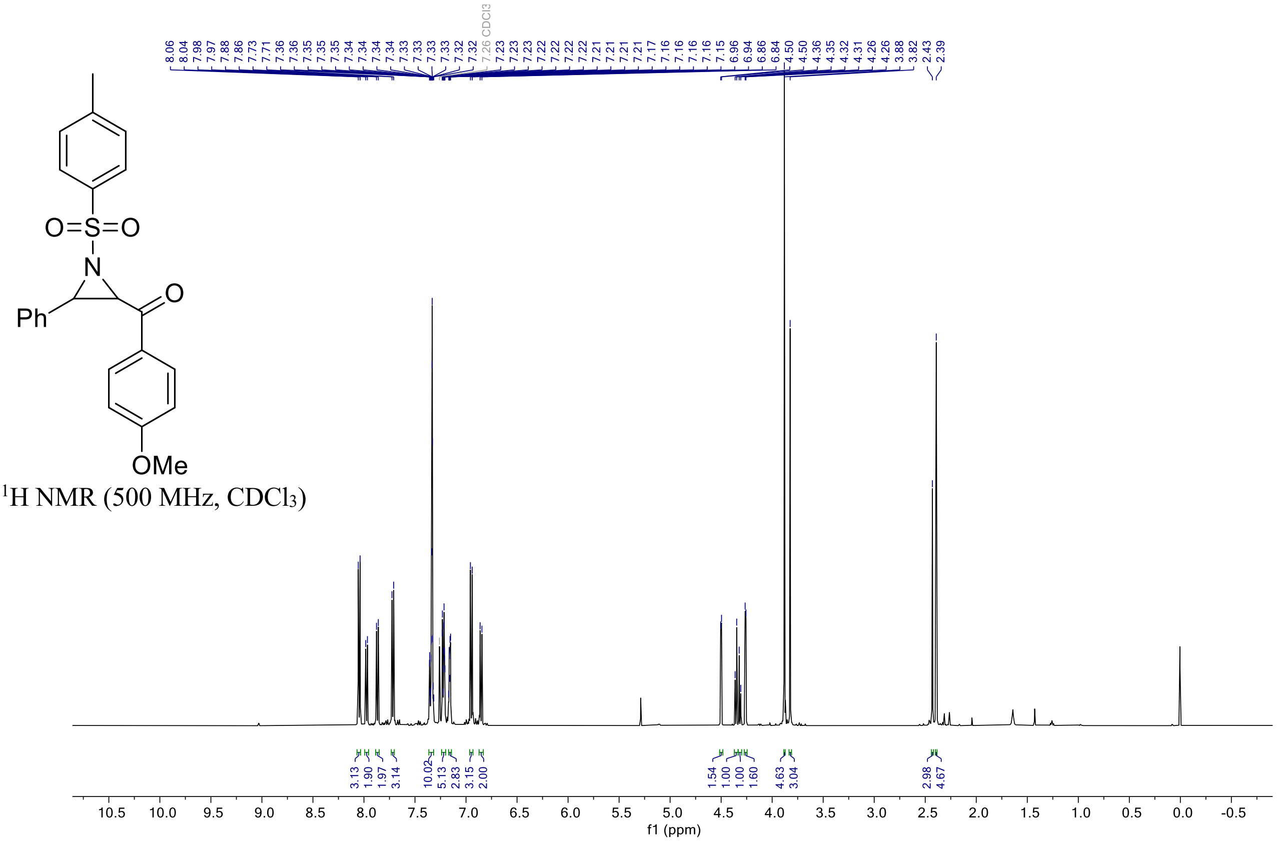

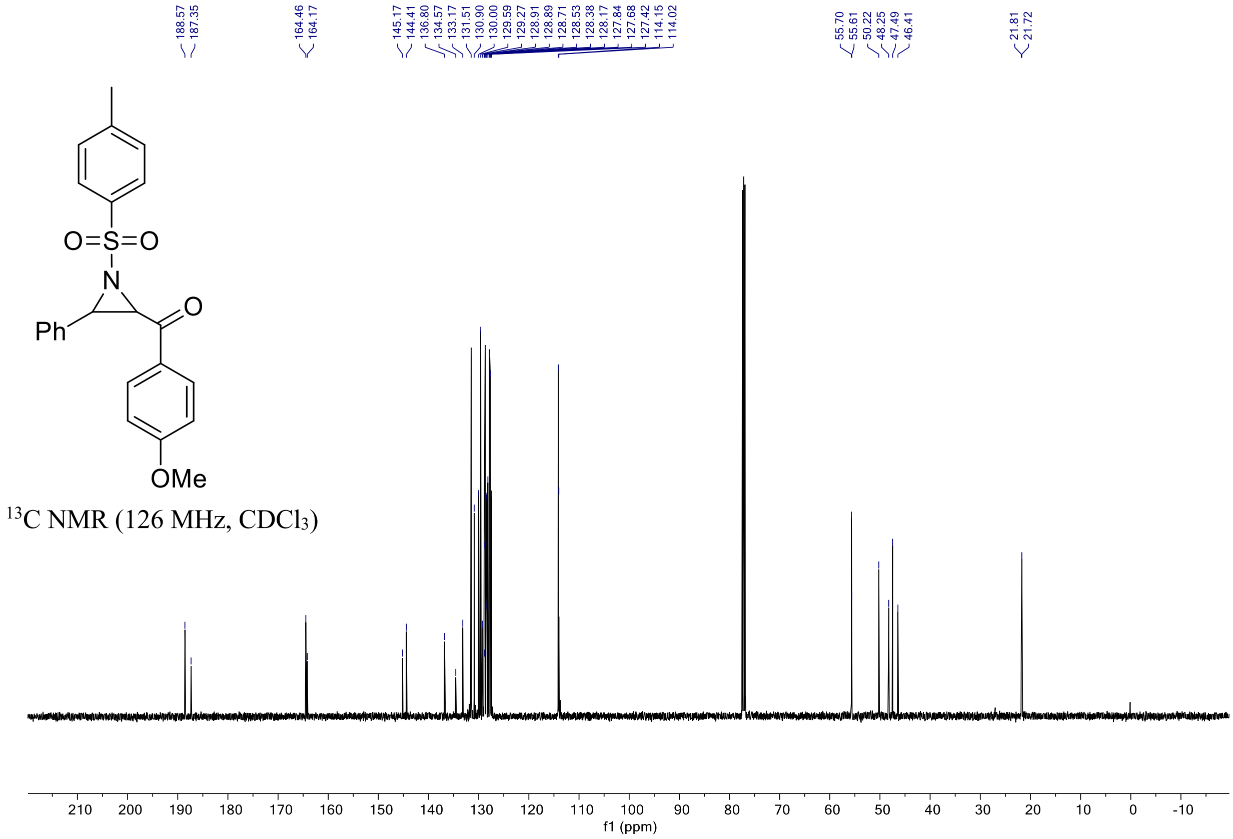

(3-Methoxyphenyl)(3-phenyl-1-tosylaziridin-2-yl)methanone (1k)

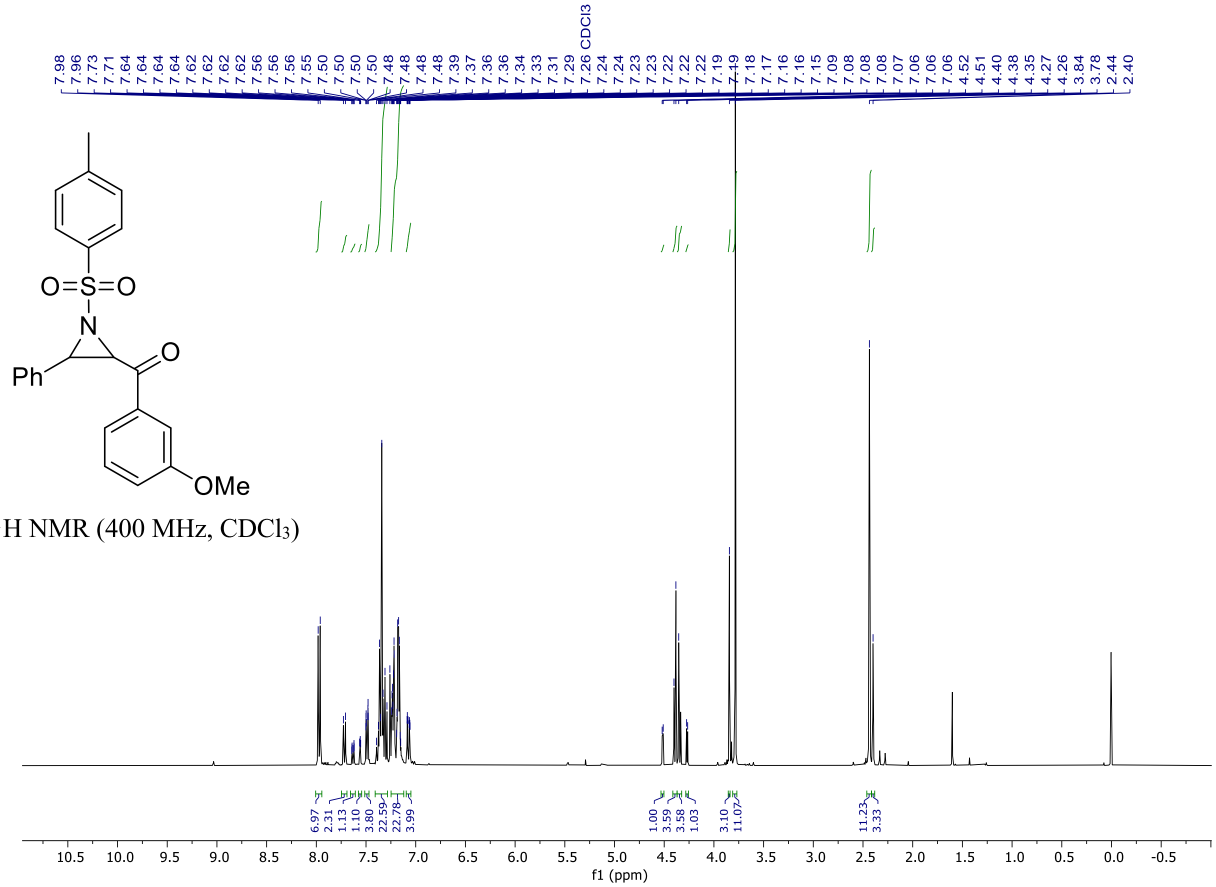

^{1}H NMR (400 MHz, $CDCl_3$)

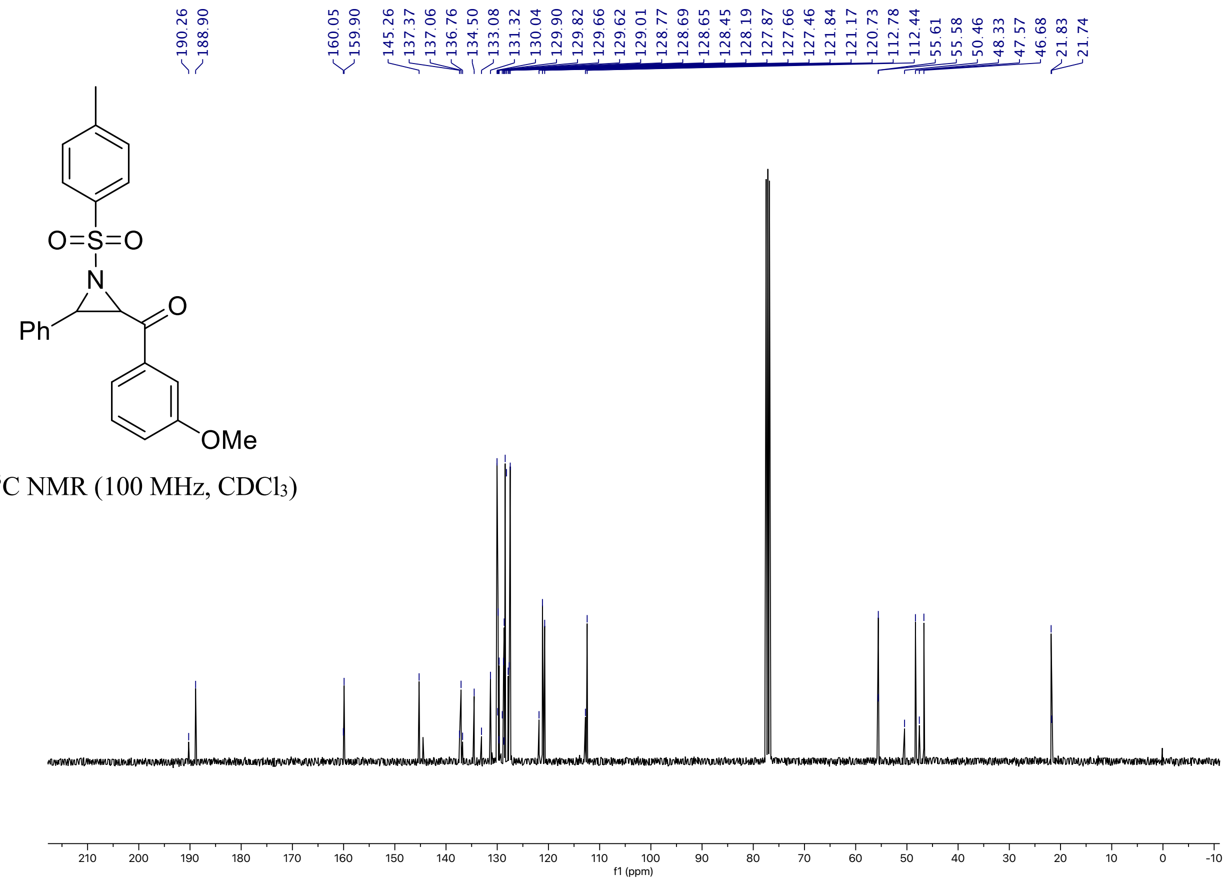

^{13}C NMR (100 MHz, $CDCl_3$)

(2-Methoxyphenyl)(3-phenyl-1-tosylaziridin-2-yl)methanone (1l)

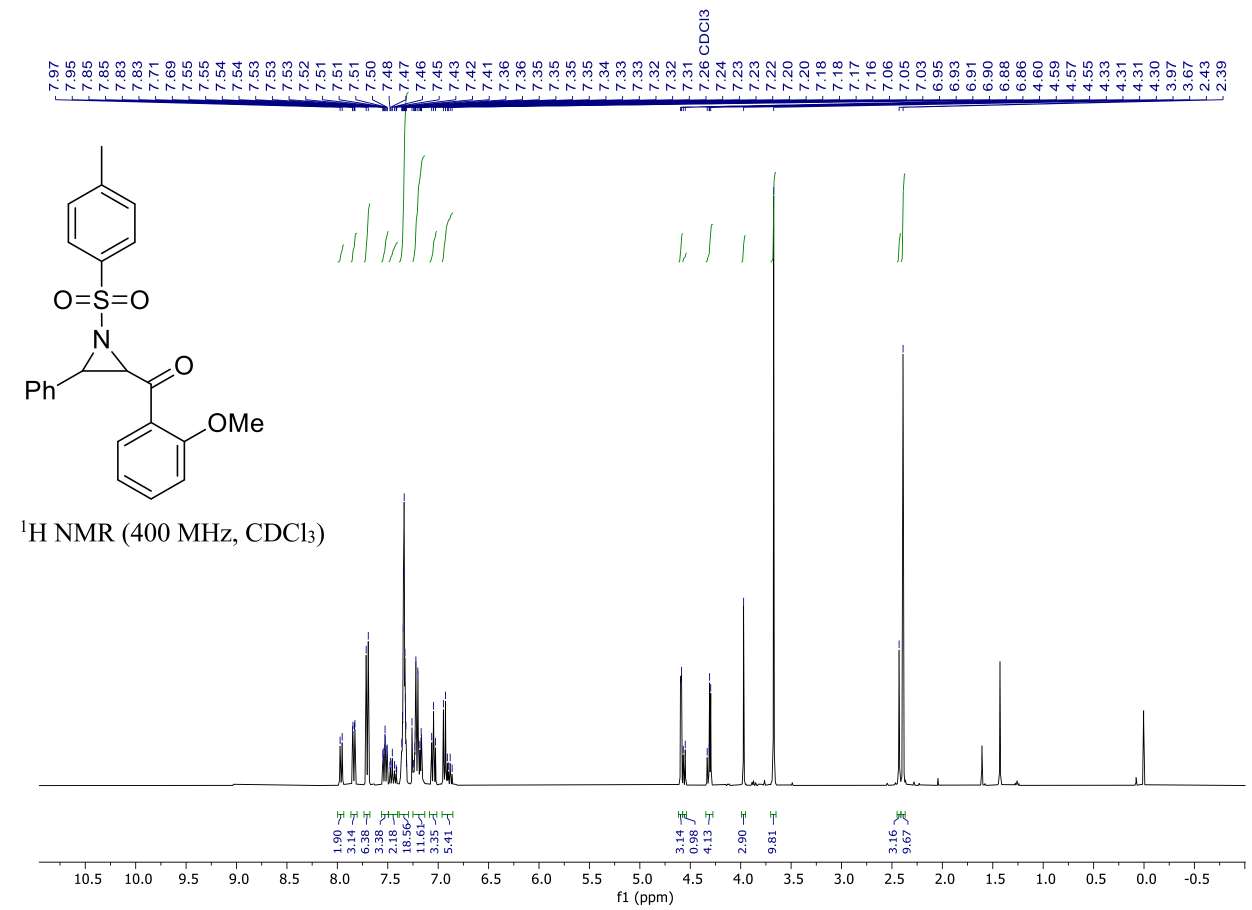

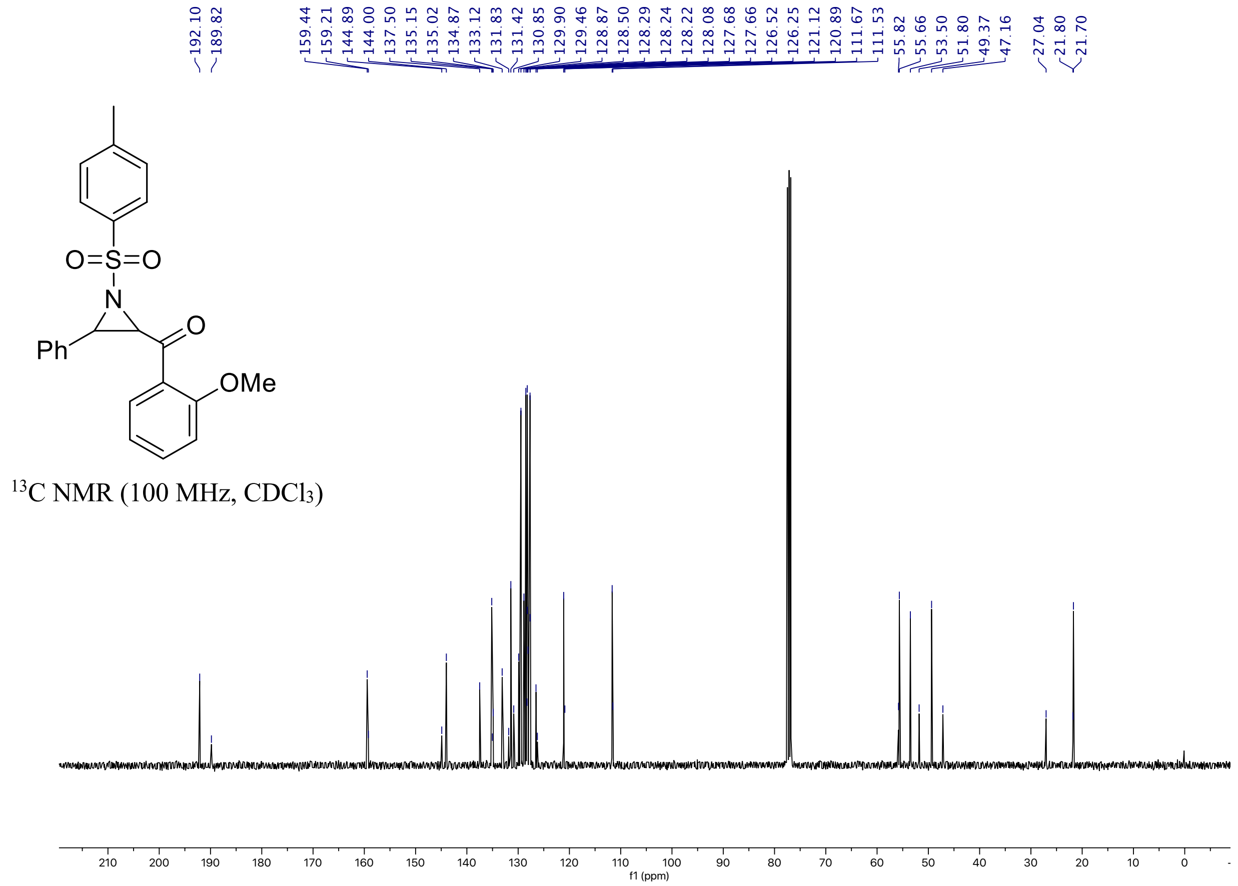

Naphthalen-2-yl(3-phenyl-1-tosylaziridin-2-yl)methanone (1m)

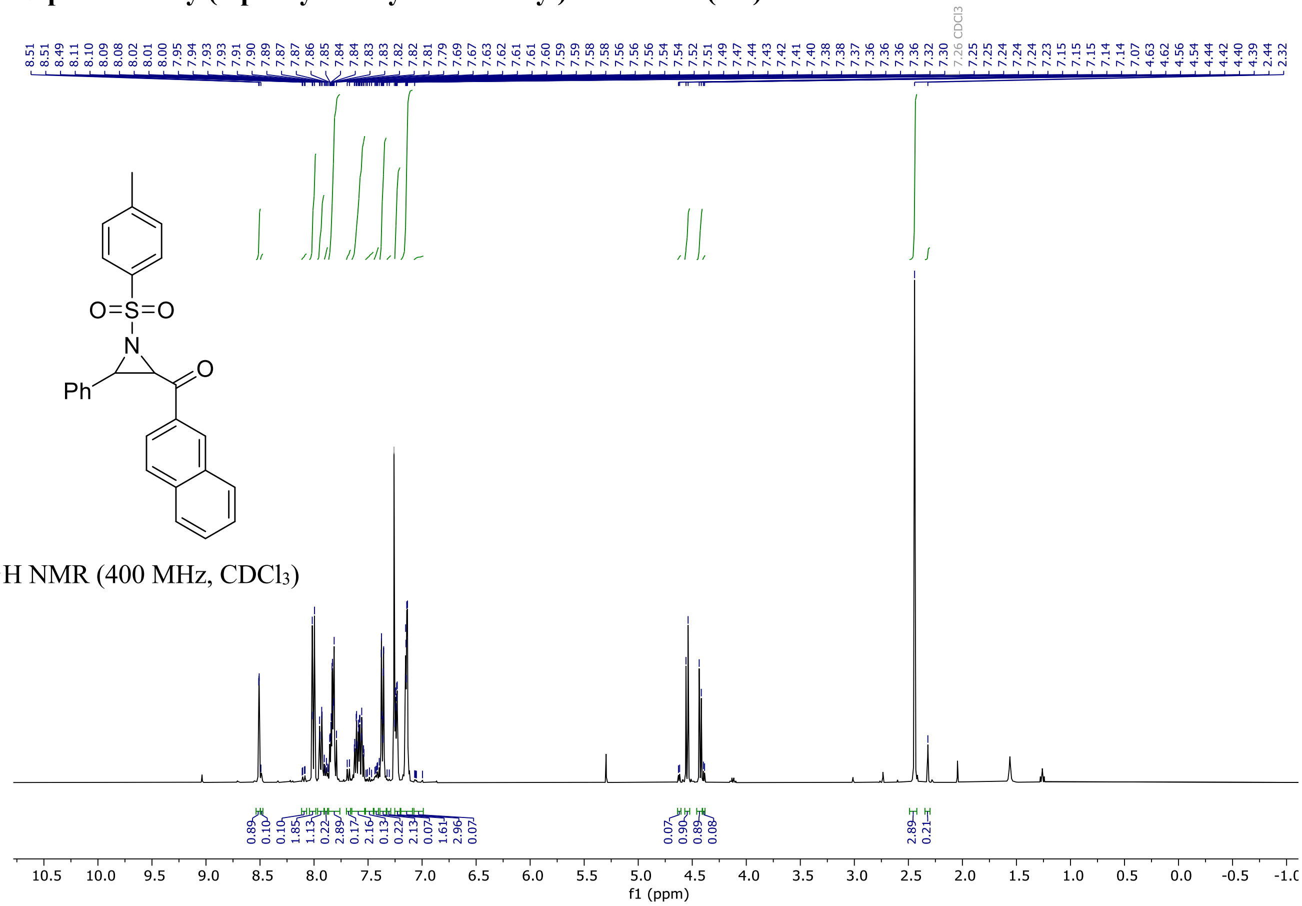

^{1}H NMR (400 MHz, $CDCl_3$)

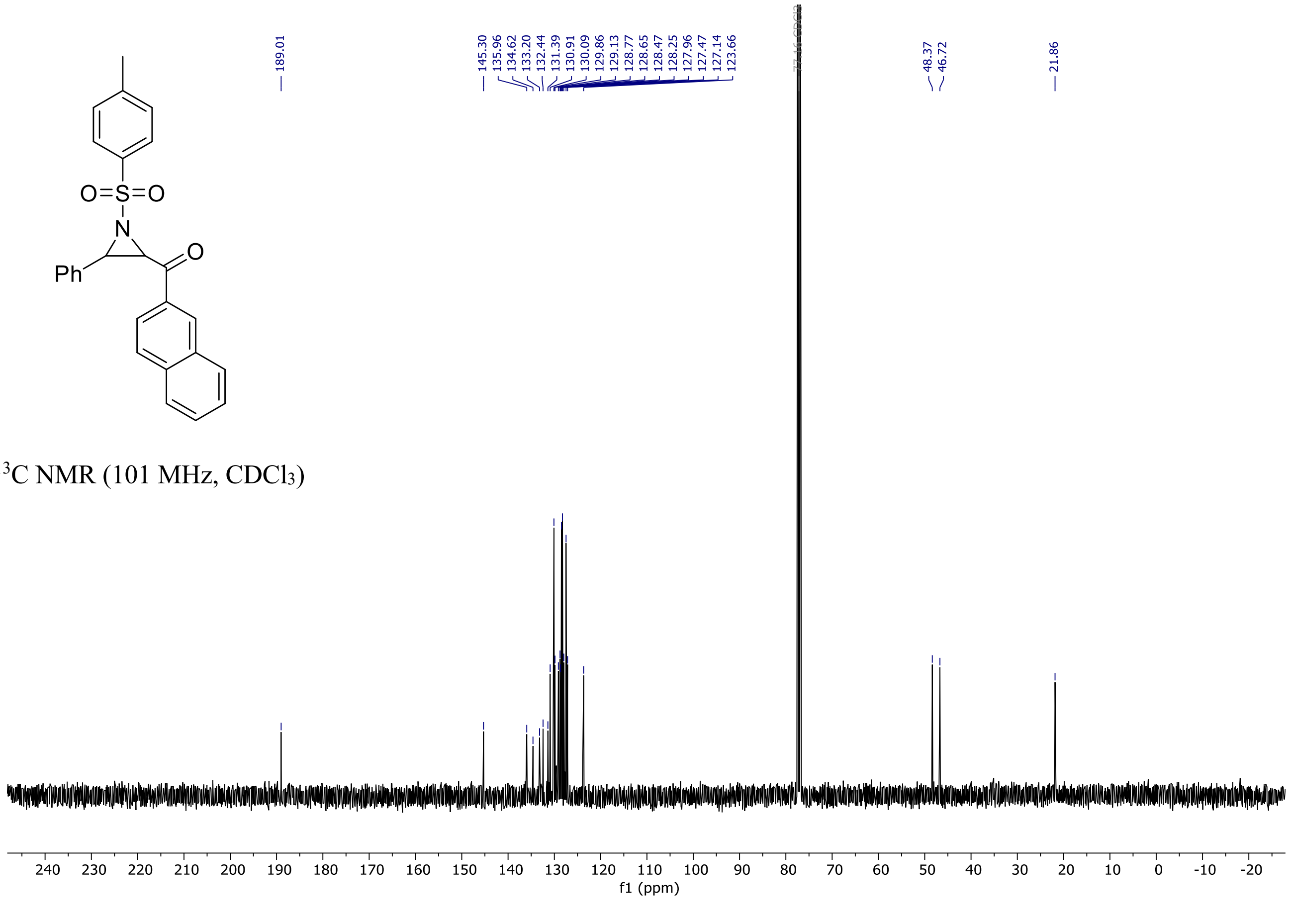

^{13}C NMR (101 MHz, $CDCl_3$)

(4-Chlorophenyl)(3-phenyl-1-tosylaziridin-2-yl)methanone (1n)

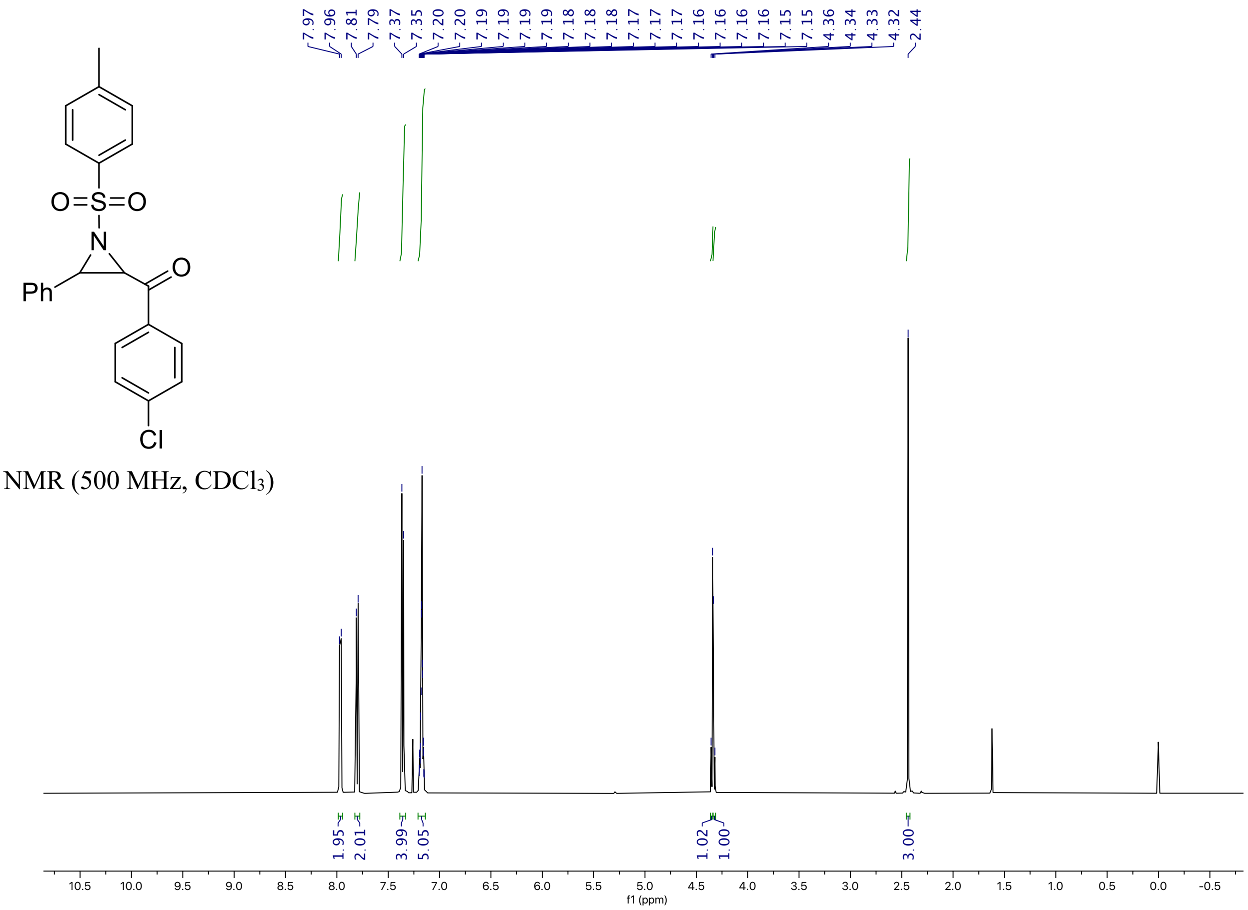

^{1}H NMR (500 MHz, $CDCl_3$)

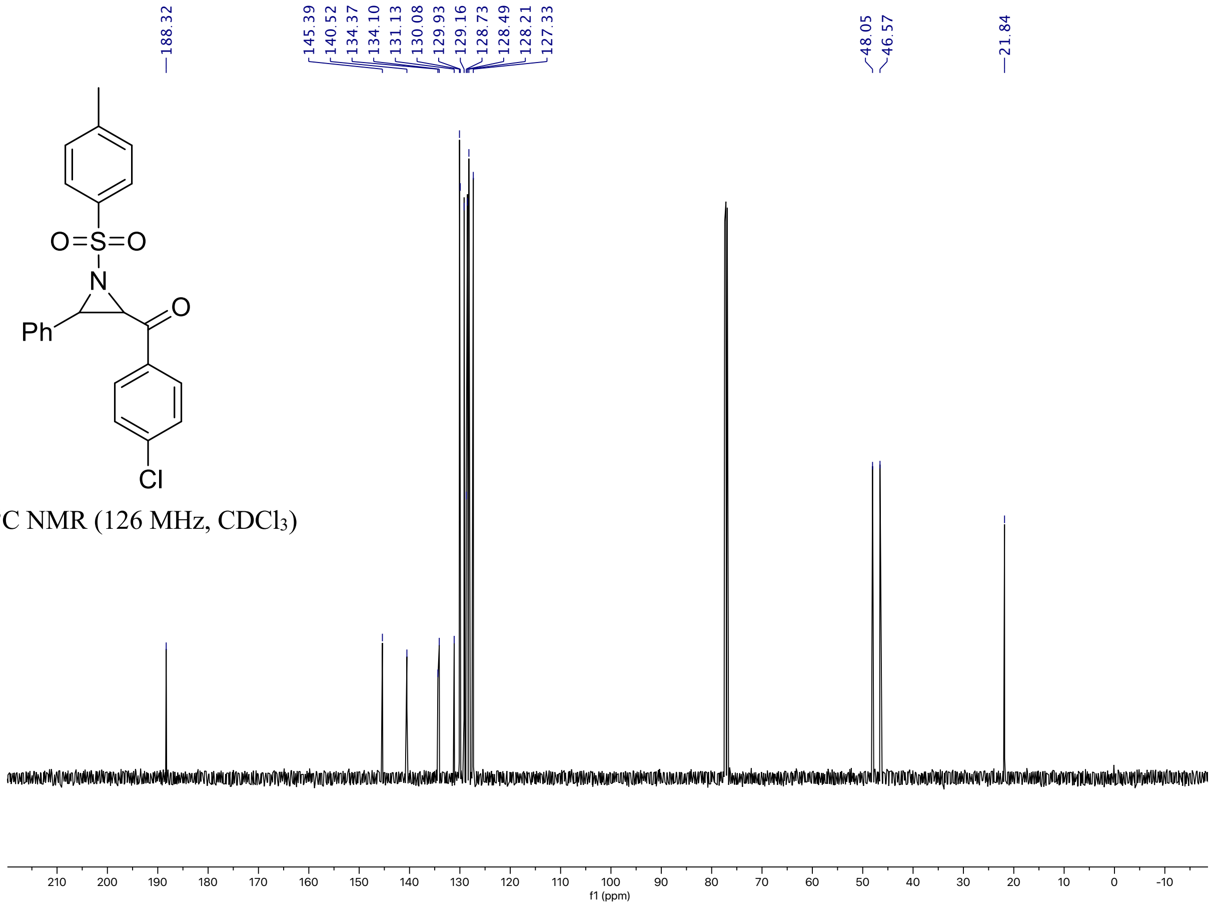

^{13}C NMR (126 MHz, $CDCl_3$)

(4-Bromophenyl)(3-phenyl-1-tosylaziridin-2-yl)methanone (1o)

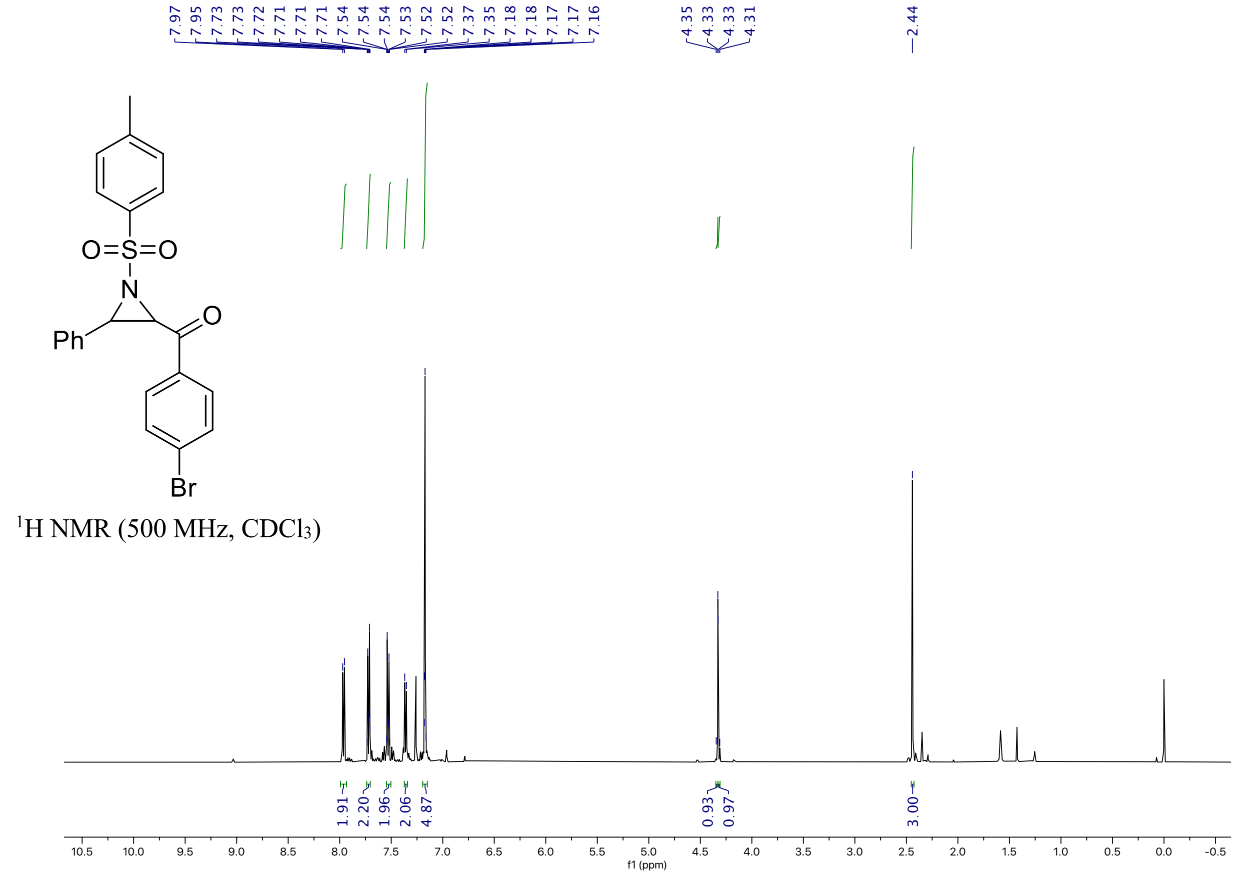

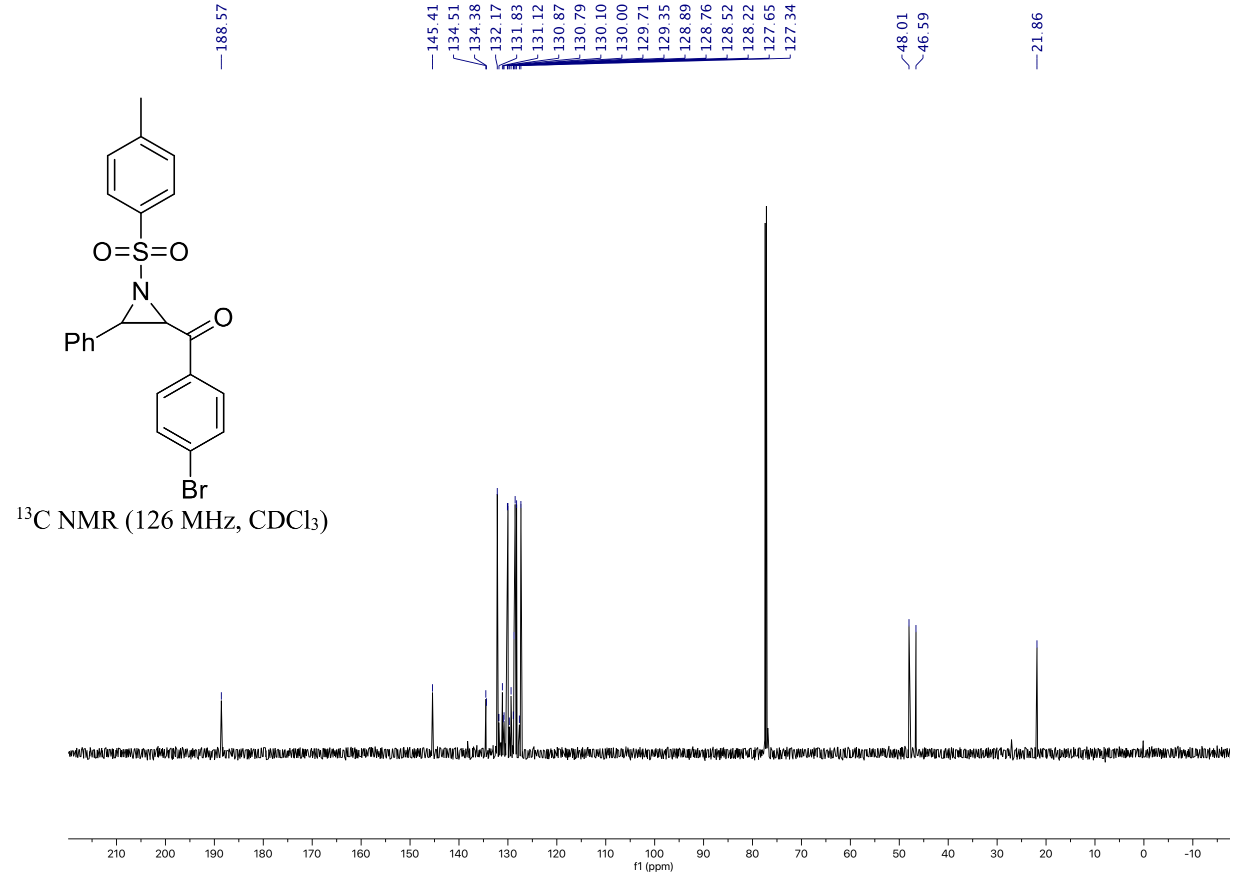

(4-Fluoroophenyl)(3-phenyl-1-tosylaziridin-2-yl)methanone (1p)

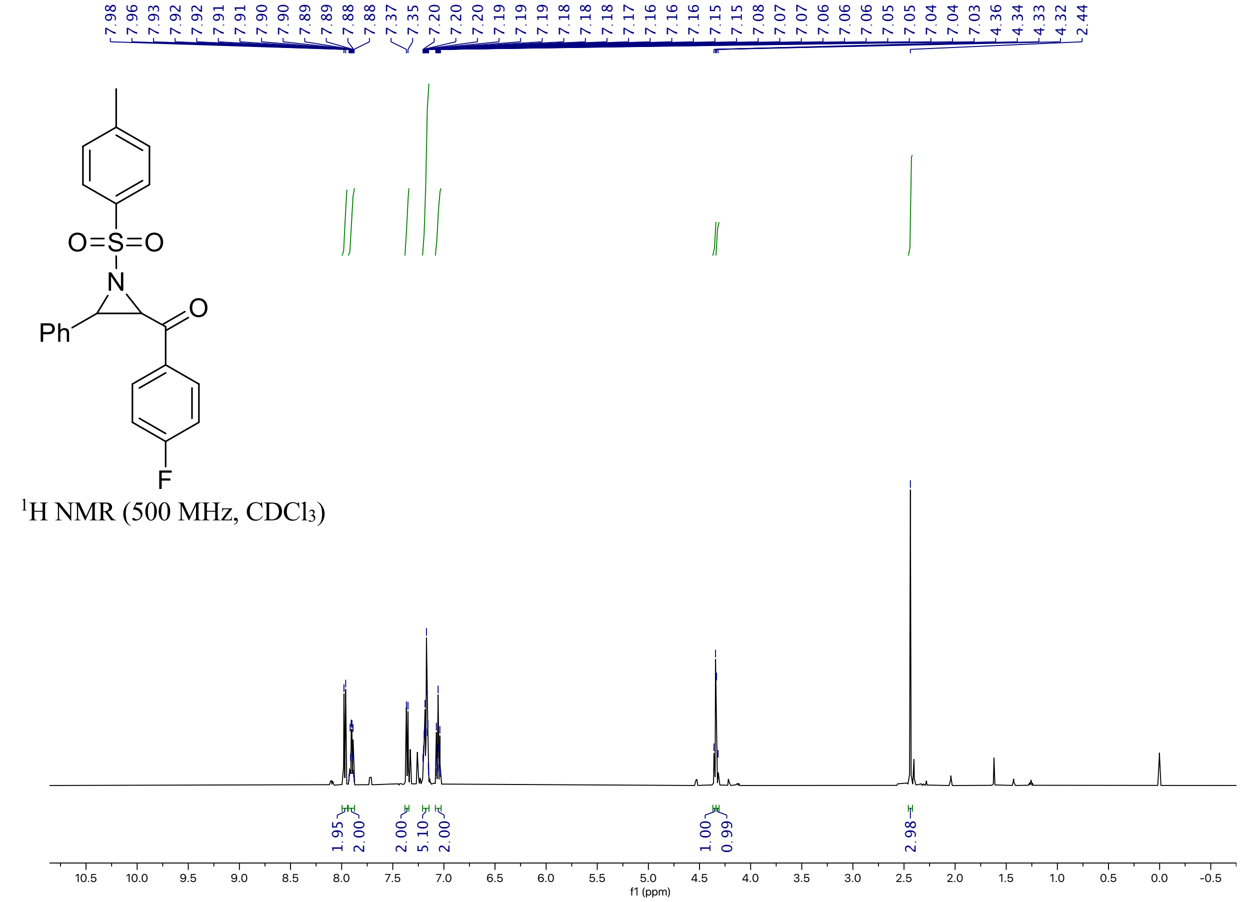

^{1}H NMR (500 MHz, $CDCl_3$)

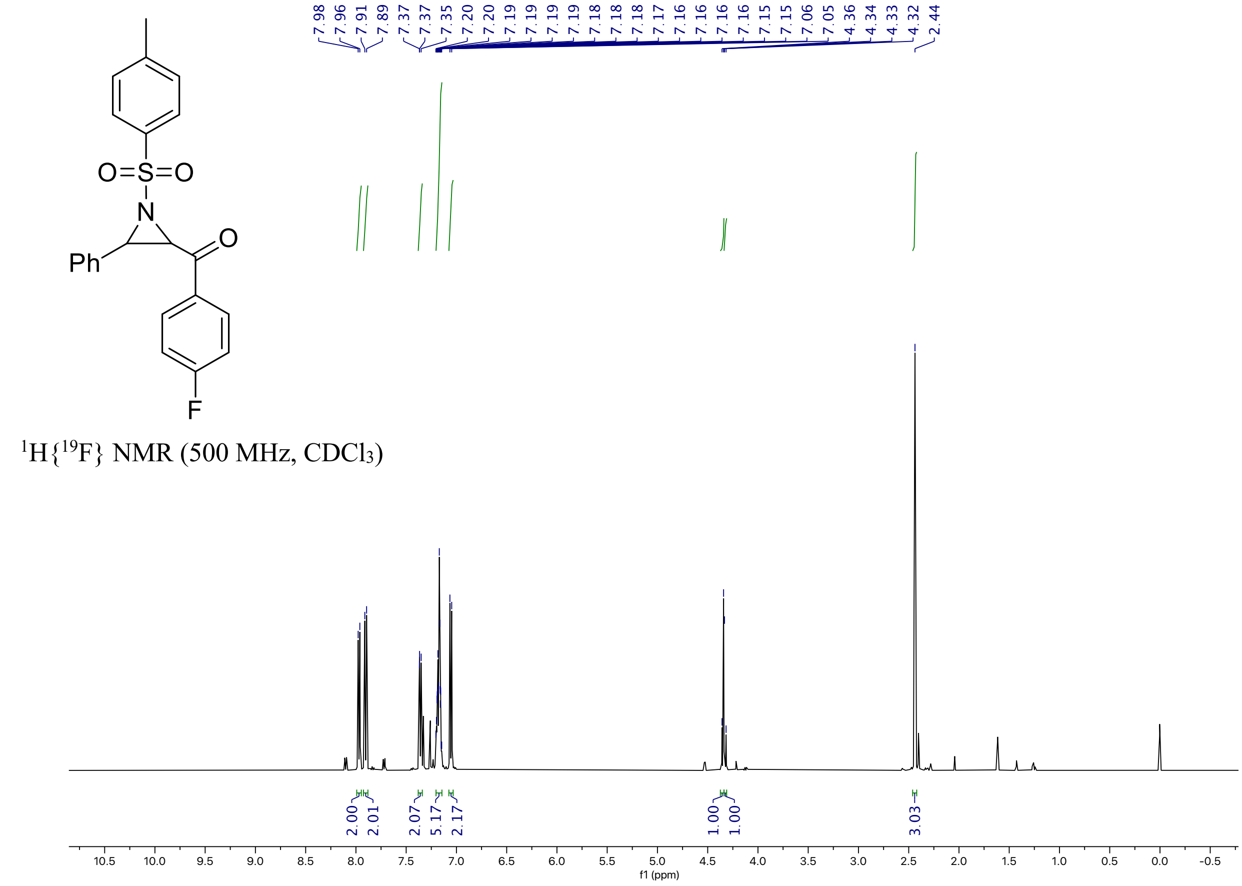

^{1}H{^{19}F} NMR (500 MHz, $CDCl_3$)

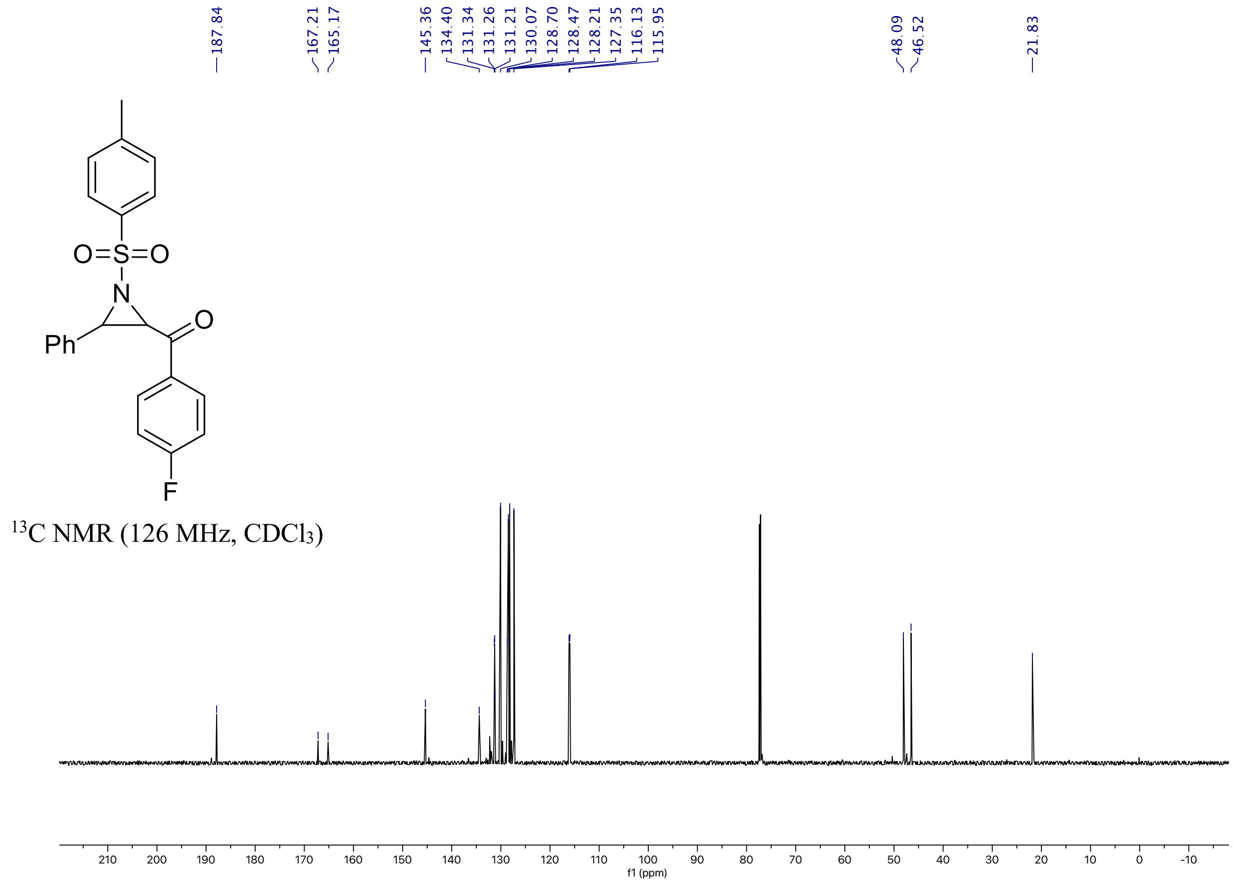

^{13}C NMR (126 MHz, $CDCl_3$)

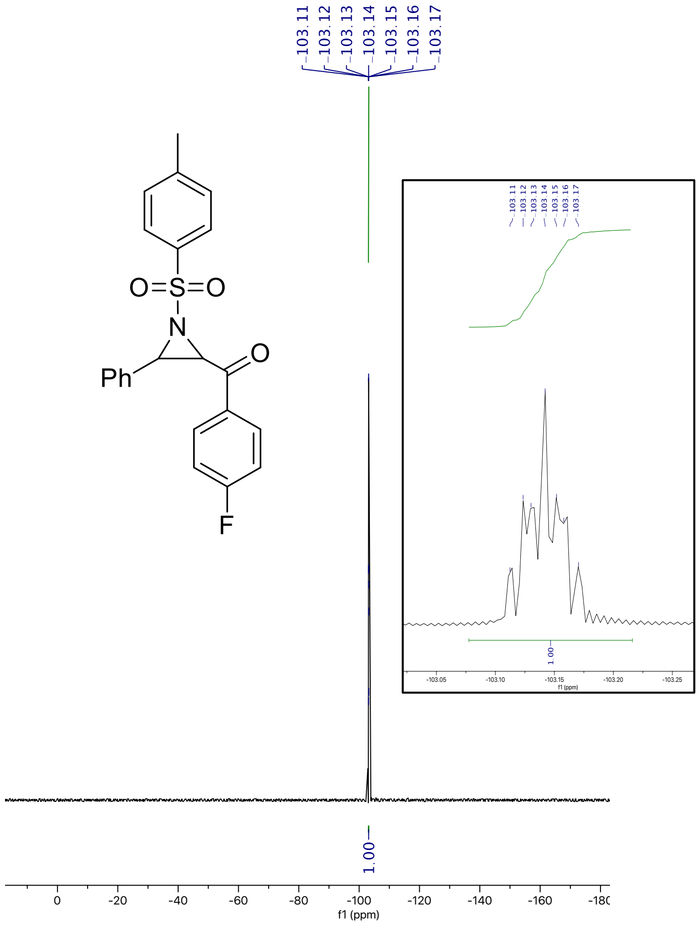

^{19}F NMR (471 MHz, $CDCl_3$)

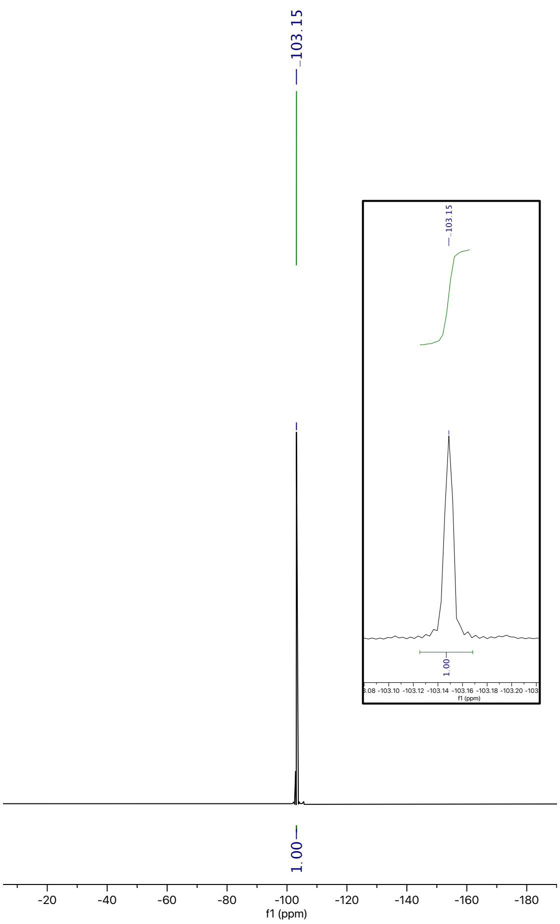

^{19}F {^{1}H} NMR (471 MHz, $CDCl_3$)

(3-Fluoroophenyl)(3-phenyl-1-tosylaziridin-2-yl)methanone (1q)

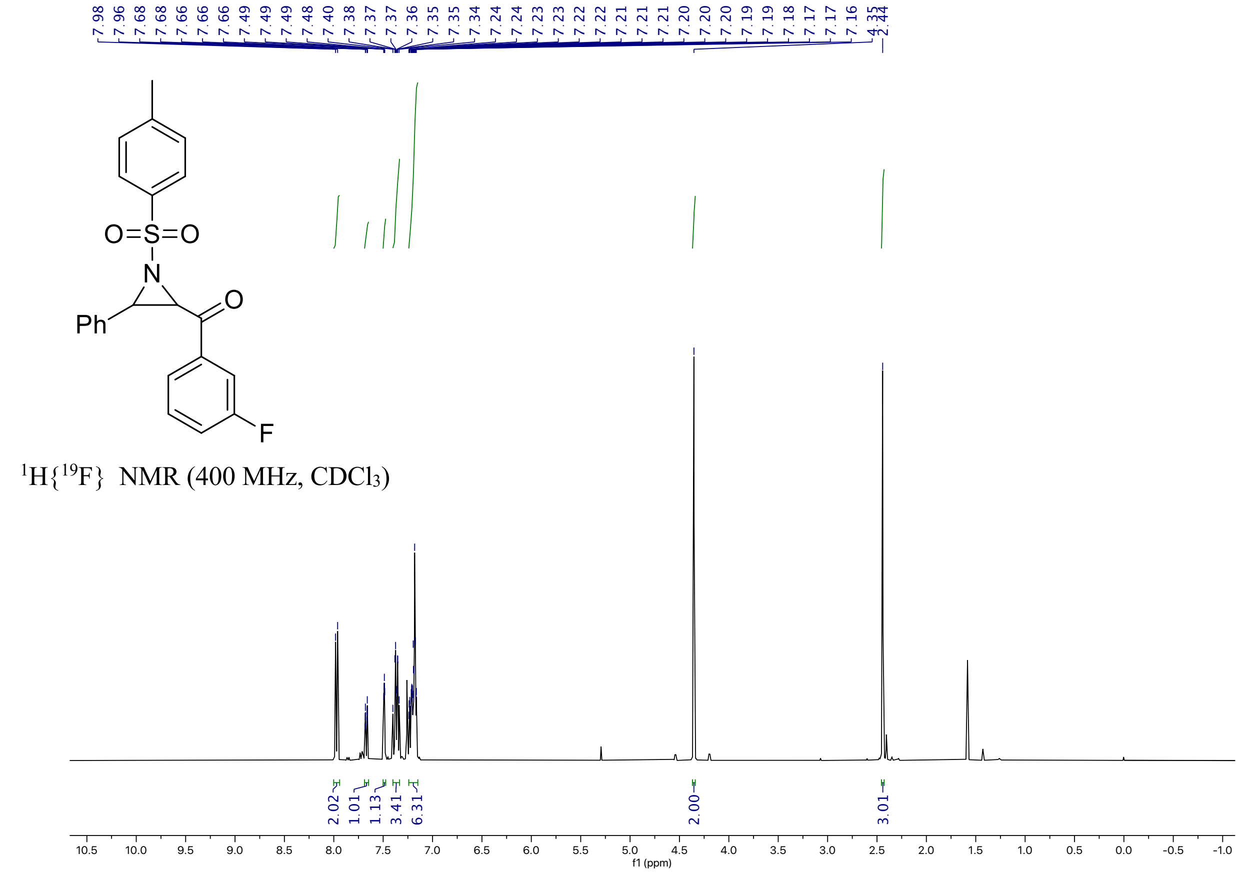

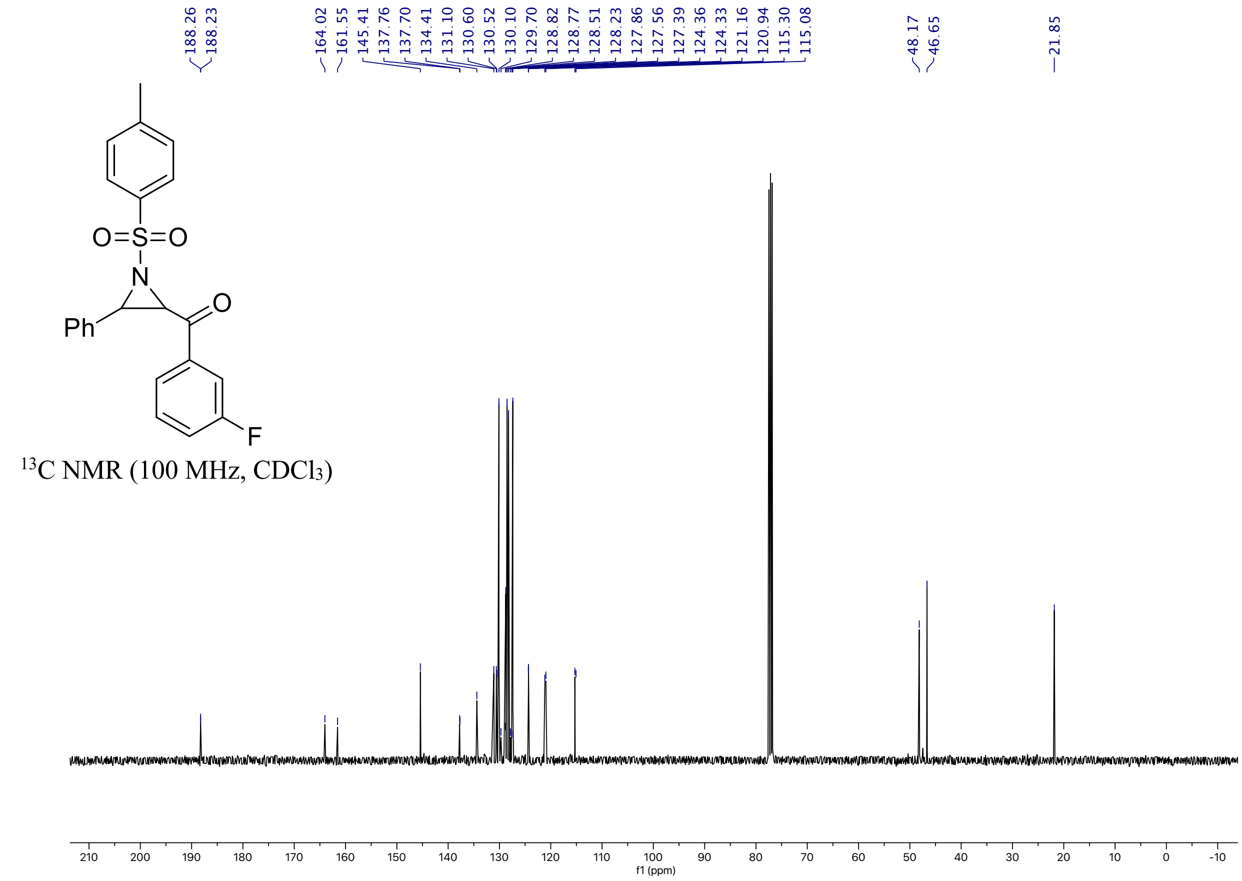

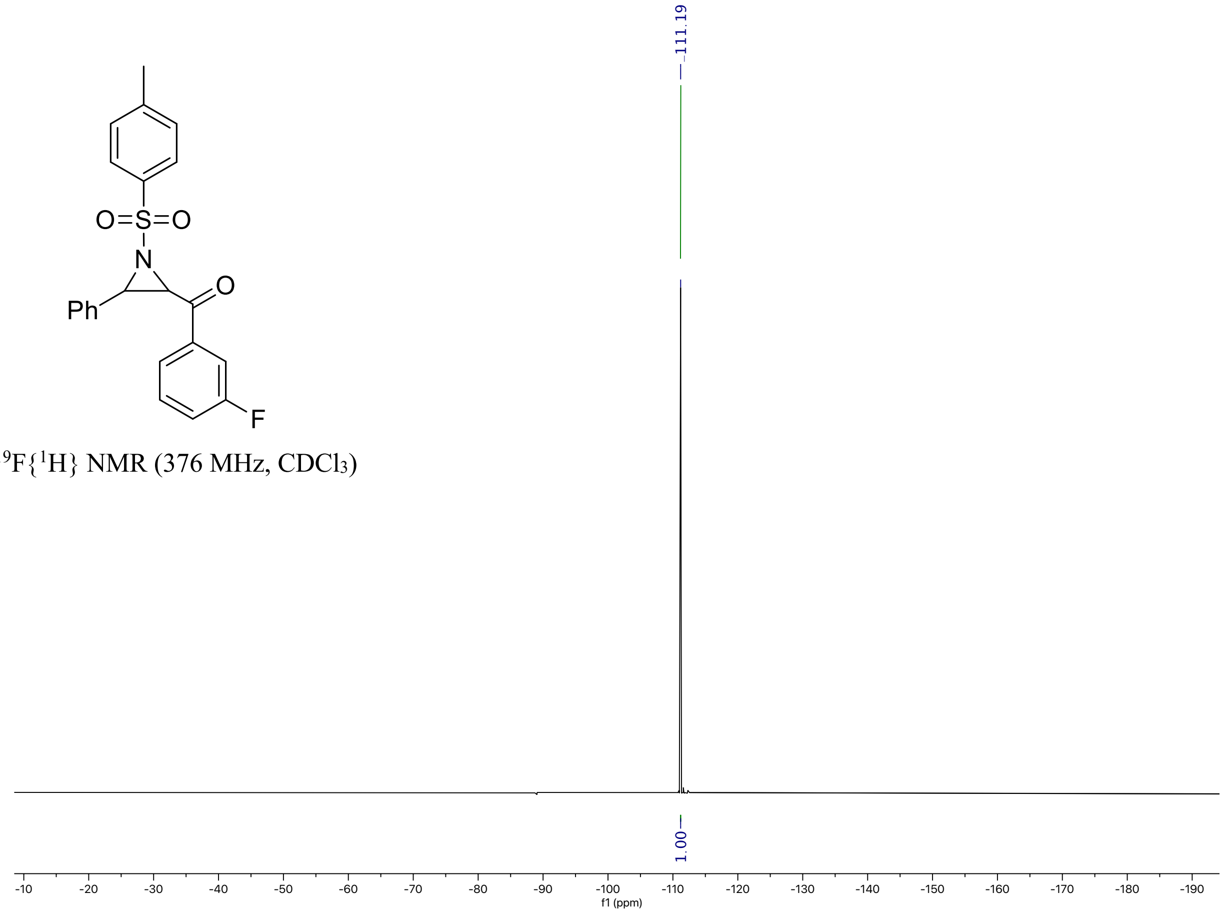

$^{19}F\{^{1}H\}$ NMR (376 MHz, $CDCl_3$)

(2-Fluorophenyl)(3-phenyl-1-tosylaziridin-2-yl)methanone (1r)

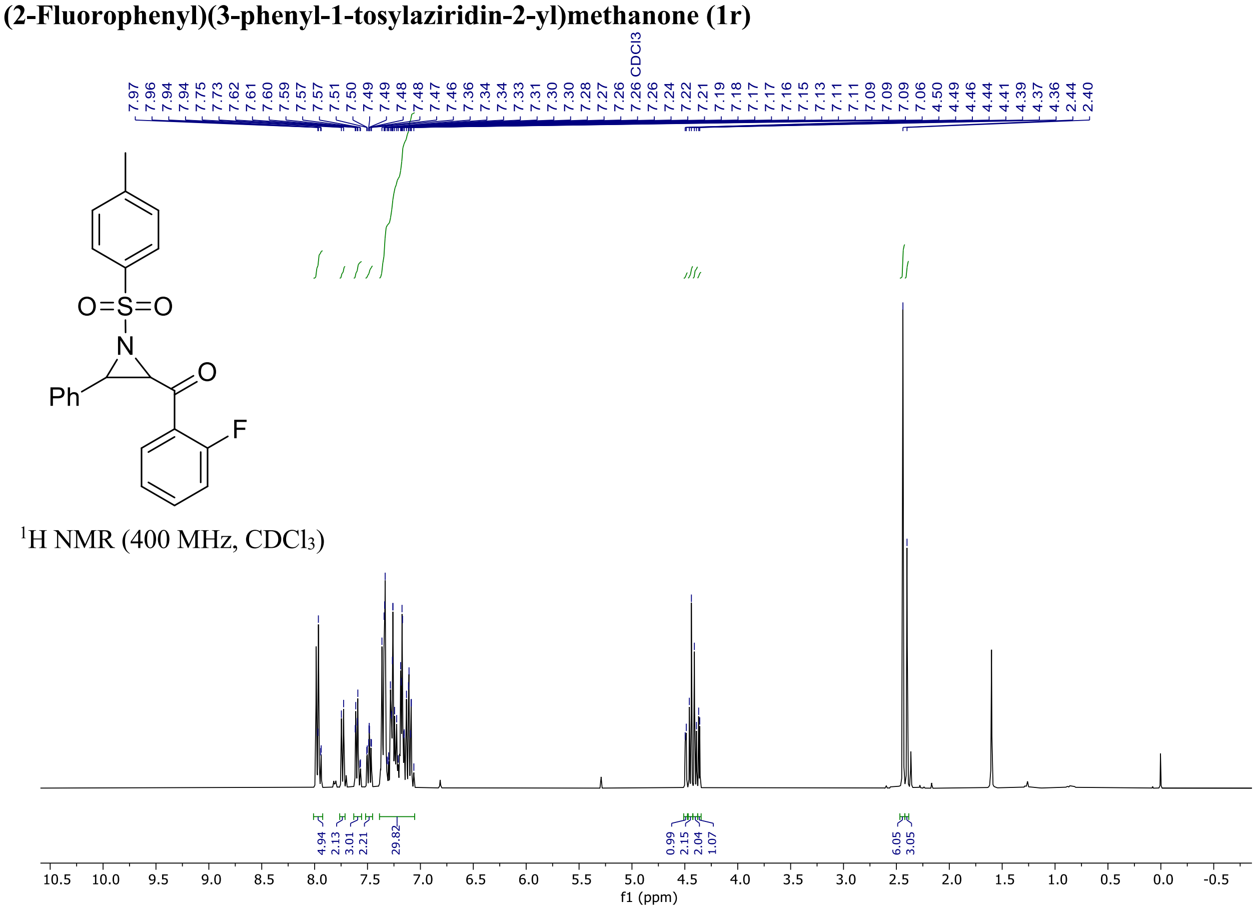

^{1}H NMR (400 MHz, $CDCl_3$)

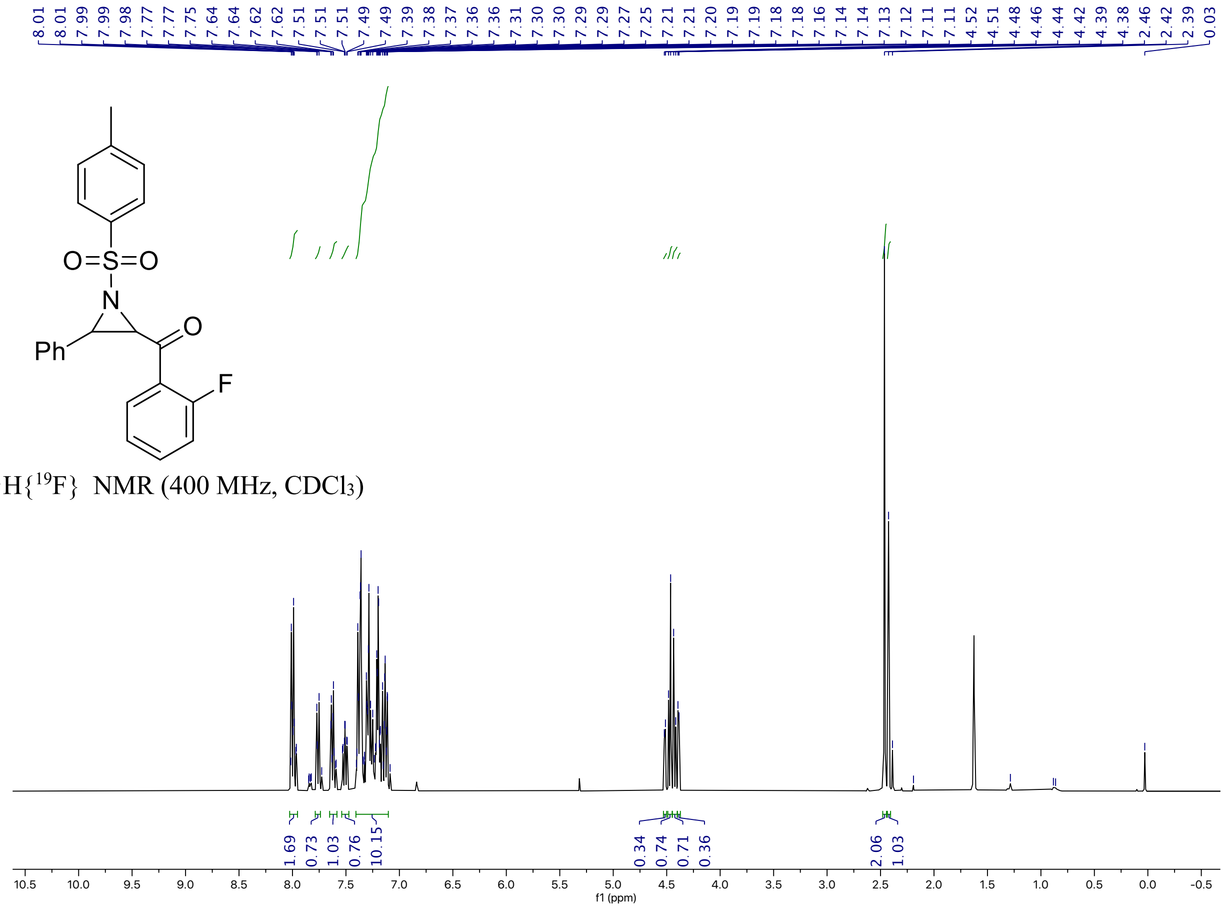

^{1}H{^{19}F} NMR (400 MHz, $CDCl_3$)

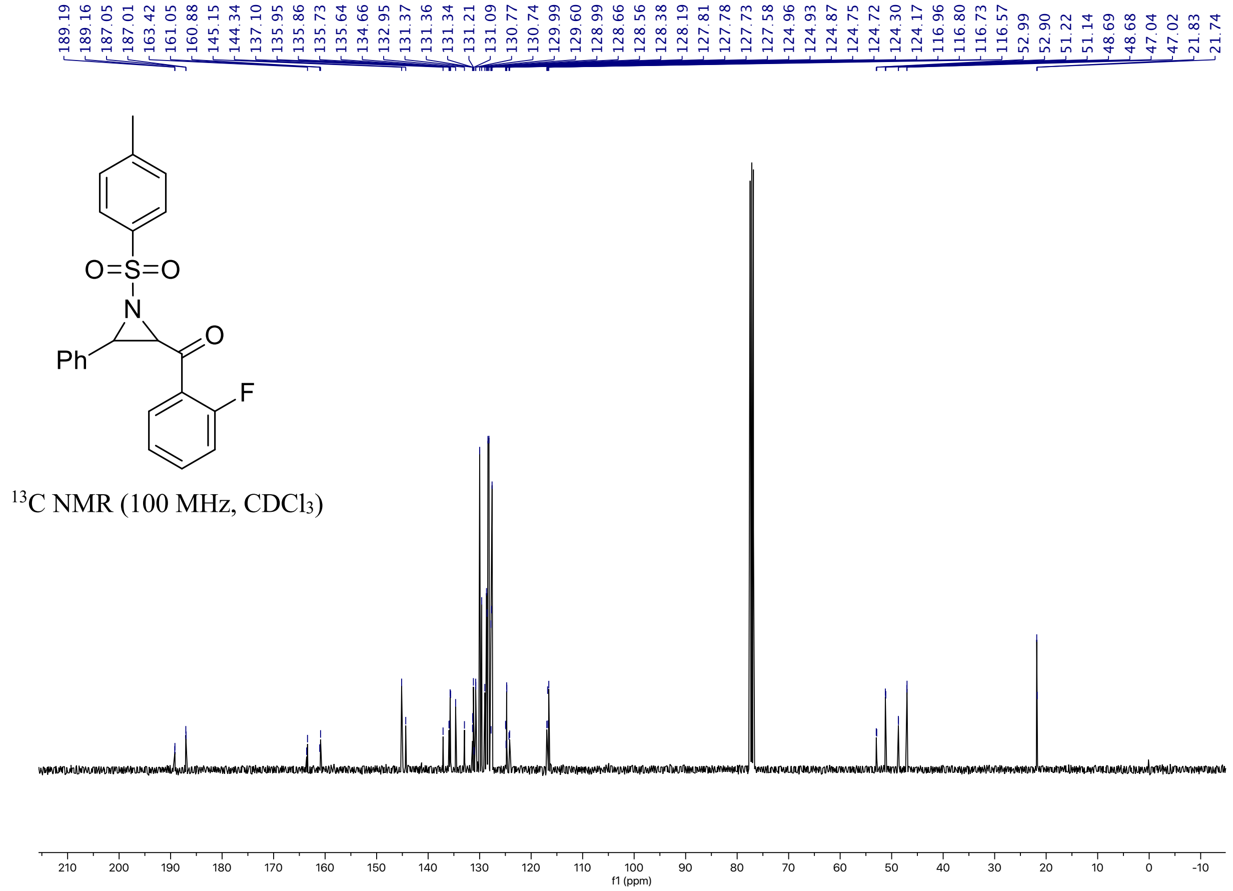

^{13}C NMR (100 MHz, CDCl$_3$)

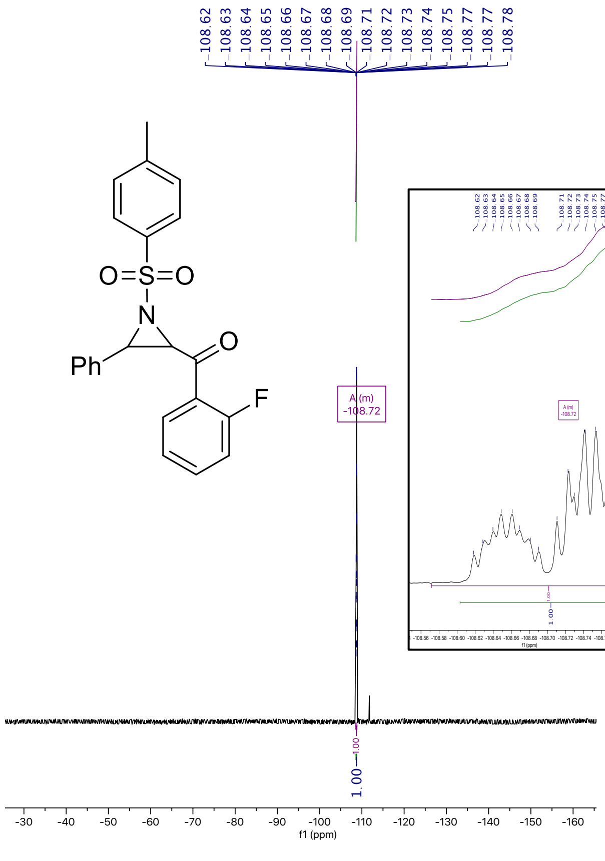

^{19}F NMR (376 MHz, CDCl$_3$)

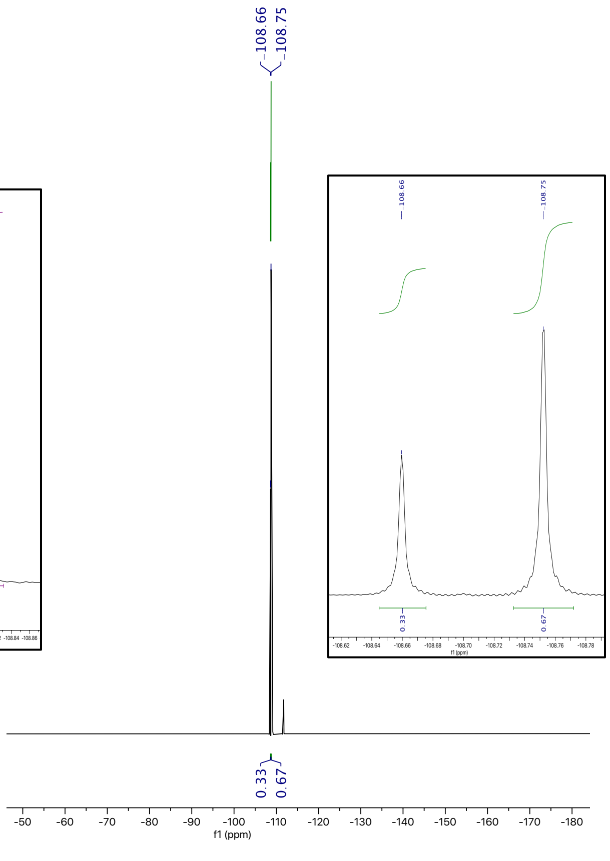

^{19}F{^{1}H} NMR (376 MHz, CDCl$_3$)

(3-Phenyl-1-tosylaziridin-2-yl)(pyridin-2-yl)methanone (1s)

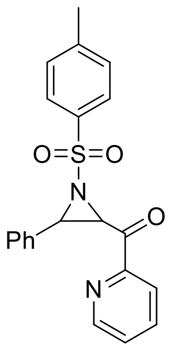

^{1}H NMR (400 MHz, $CDCl_3$)

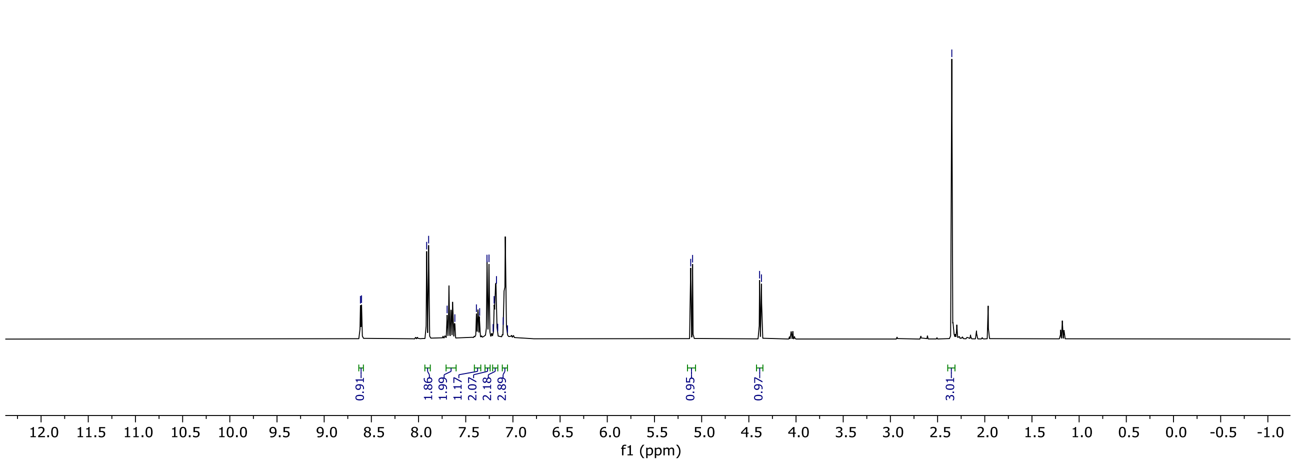

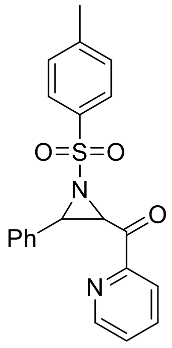

^{13}C NMR (101 MHz, $CDCl_3$)

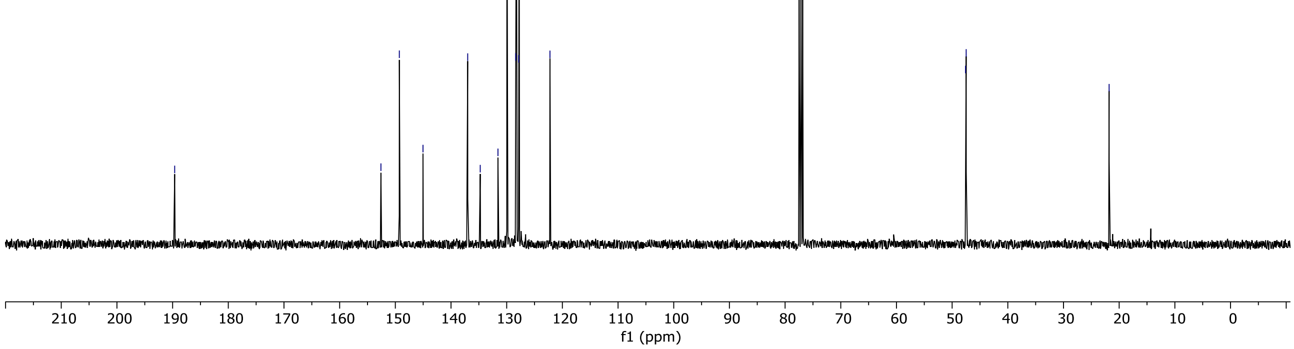

(3-Phenyl-1-tosylaziridin-2-yl)(thiophen-2-yl)methanone (1t)

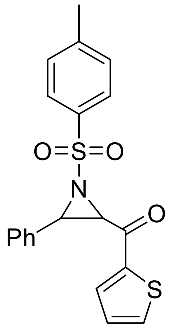

^{1}H NMR (400 MHz, $CDCl_3$)

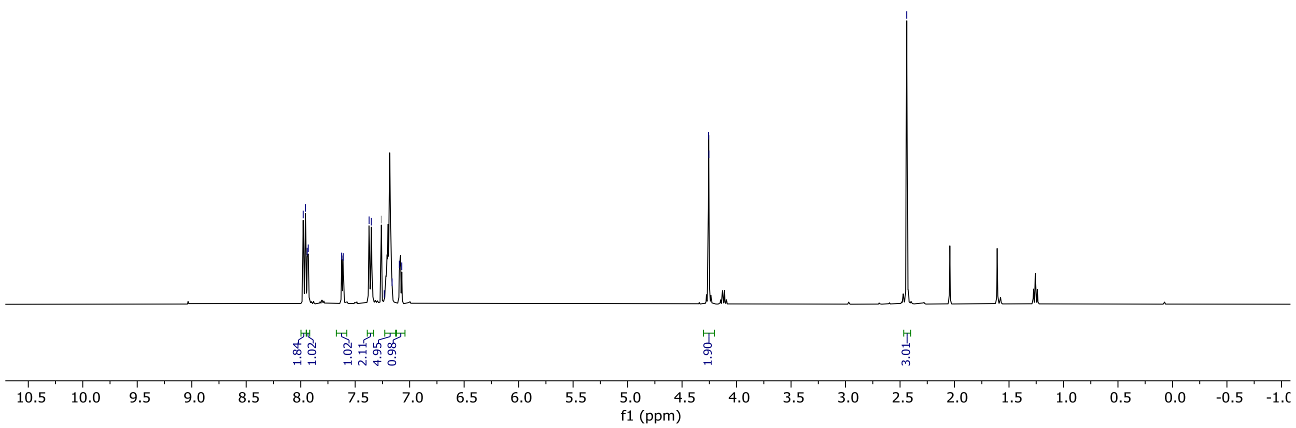

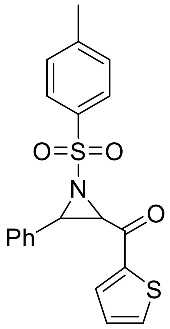

^{13}C NMR (101 MHz, $CDCl_3$)

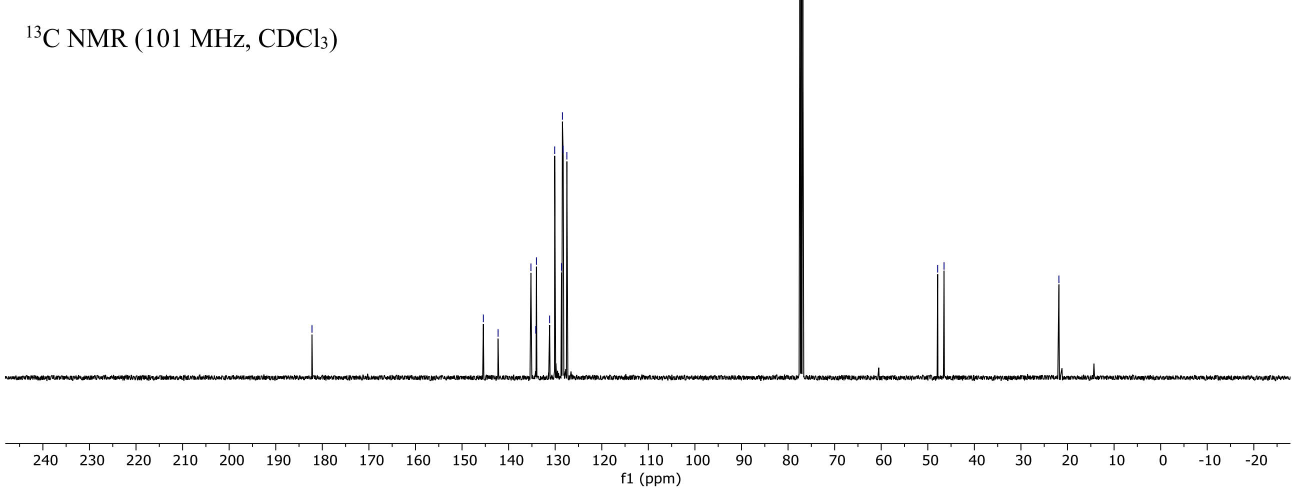

1-(3-Phenyl-1-tosylaziridin-2-yl)ethan-1-one (1u)

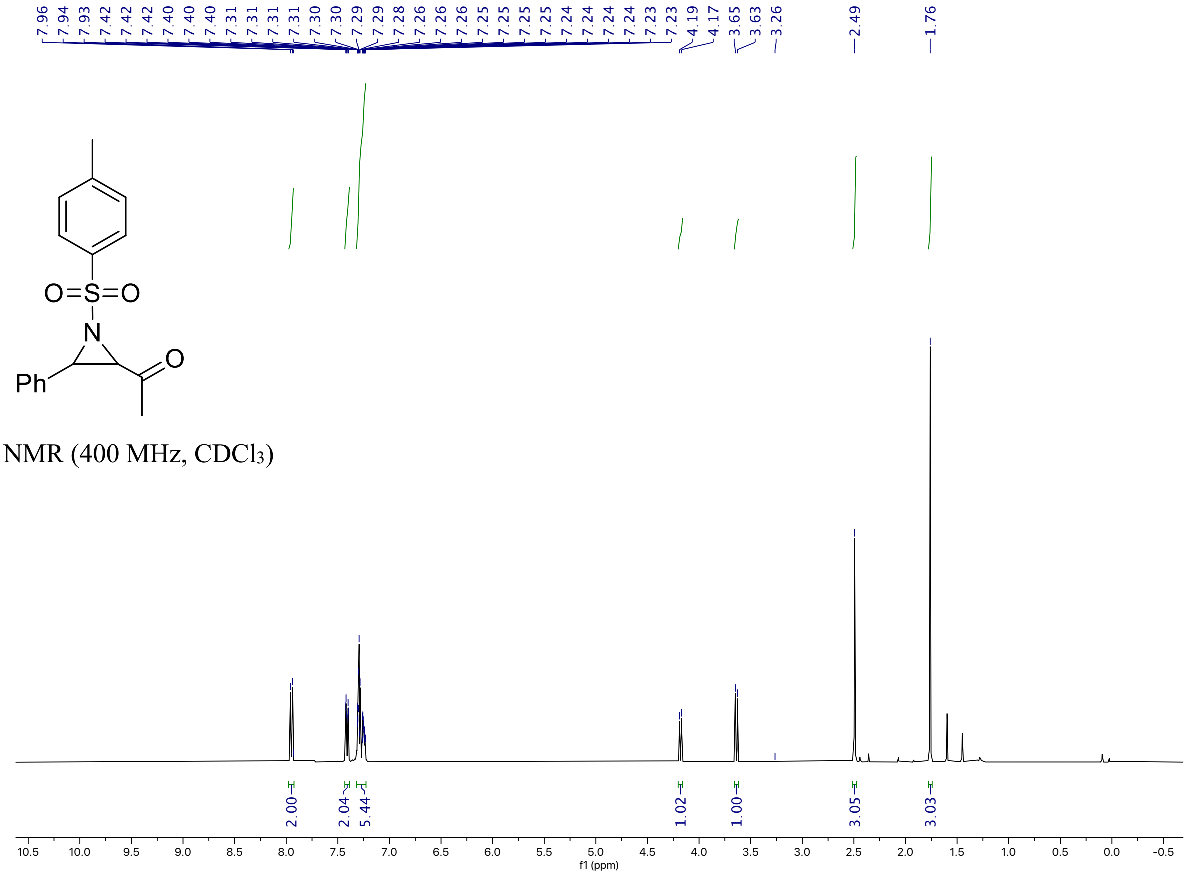

^{1}H NMR (400 MHz, $CDCl_3$)

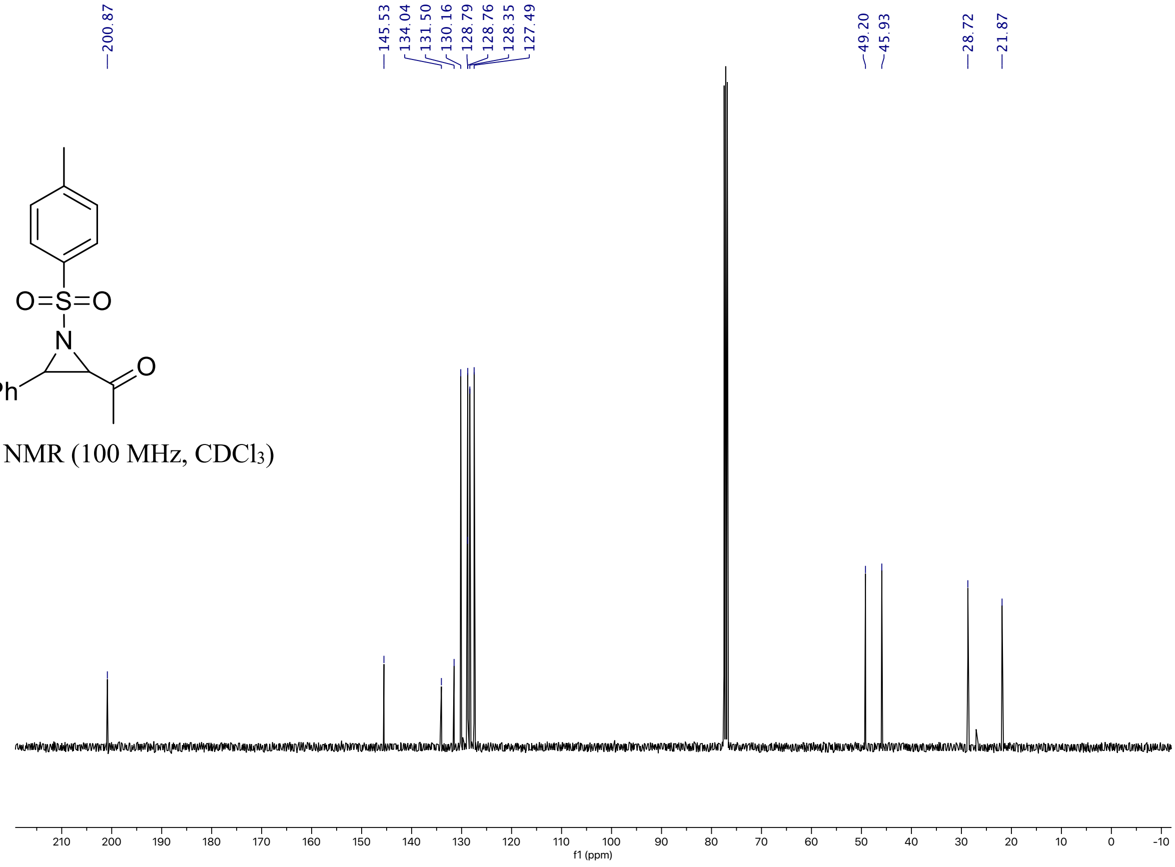

^{13}C NMR (100 MHz, $CDCl_3$)

2,2-Dimethyl-1-(3-phenyl-1-tosylaziridin-2-yl)propan-1-one (1v)

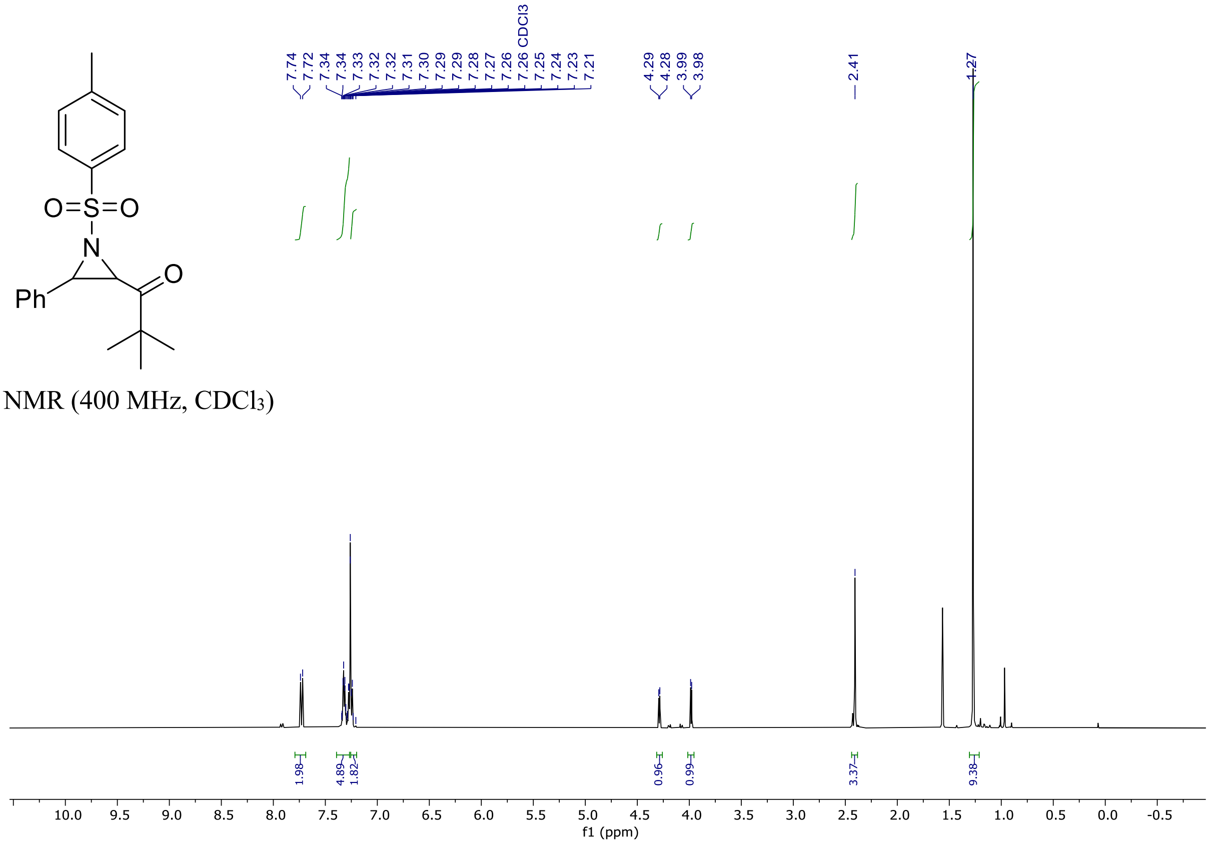

^{1}H NMR (400 MHz, $CDCl_3$)

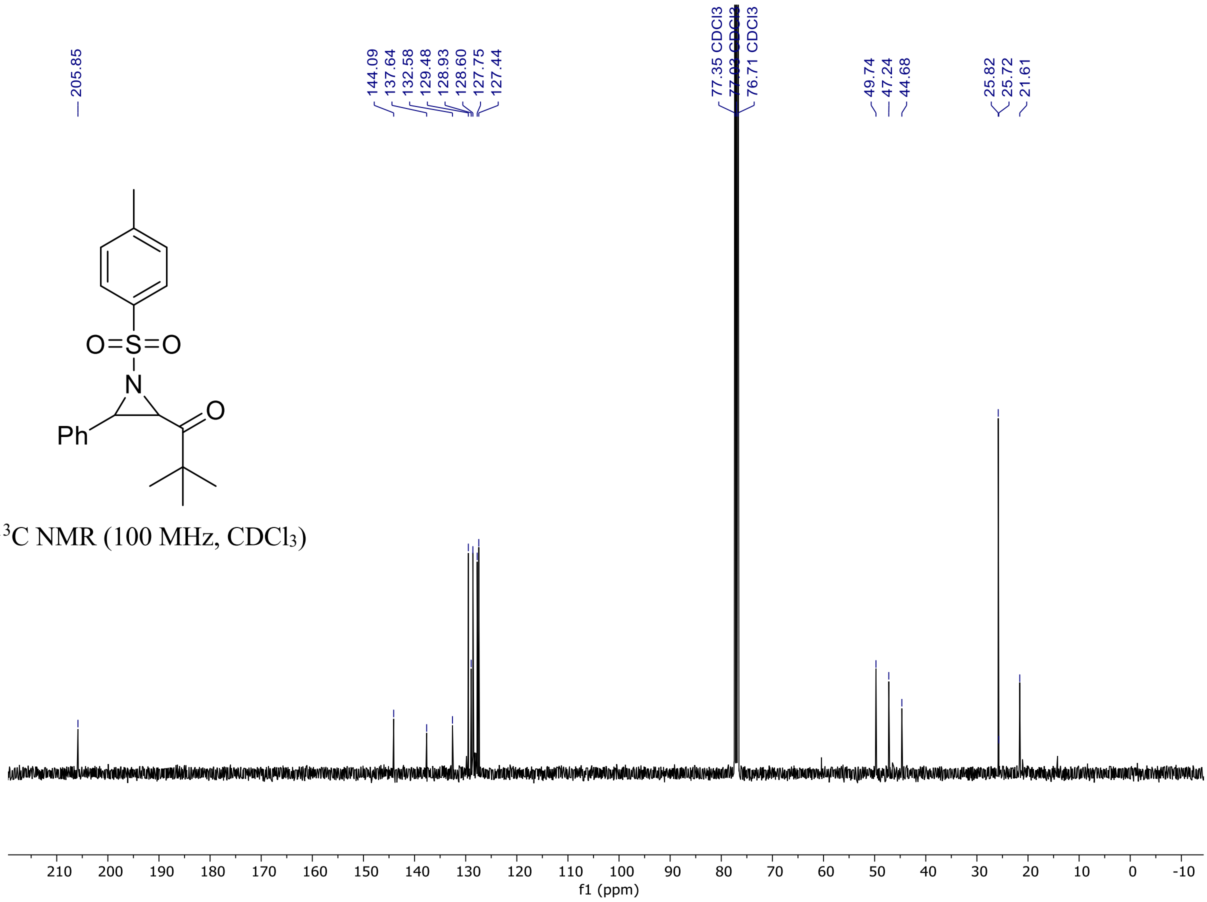

^{13}C NMR (100 MHz, $CDCl_3$)

Phenyl(3-(*p*-tolyl)-1-tosylaziridin-2-yl)methanone (1w)

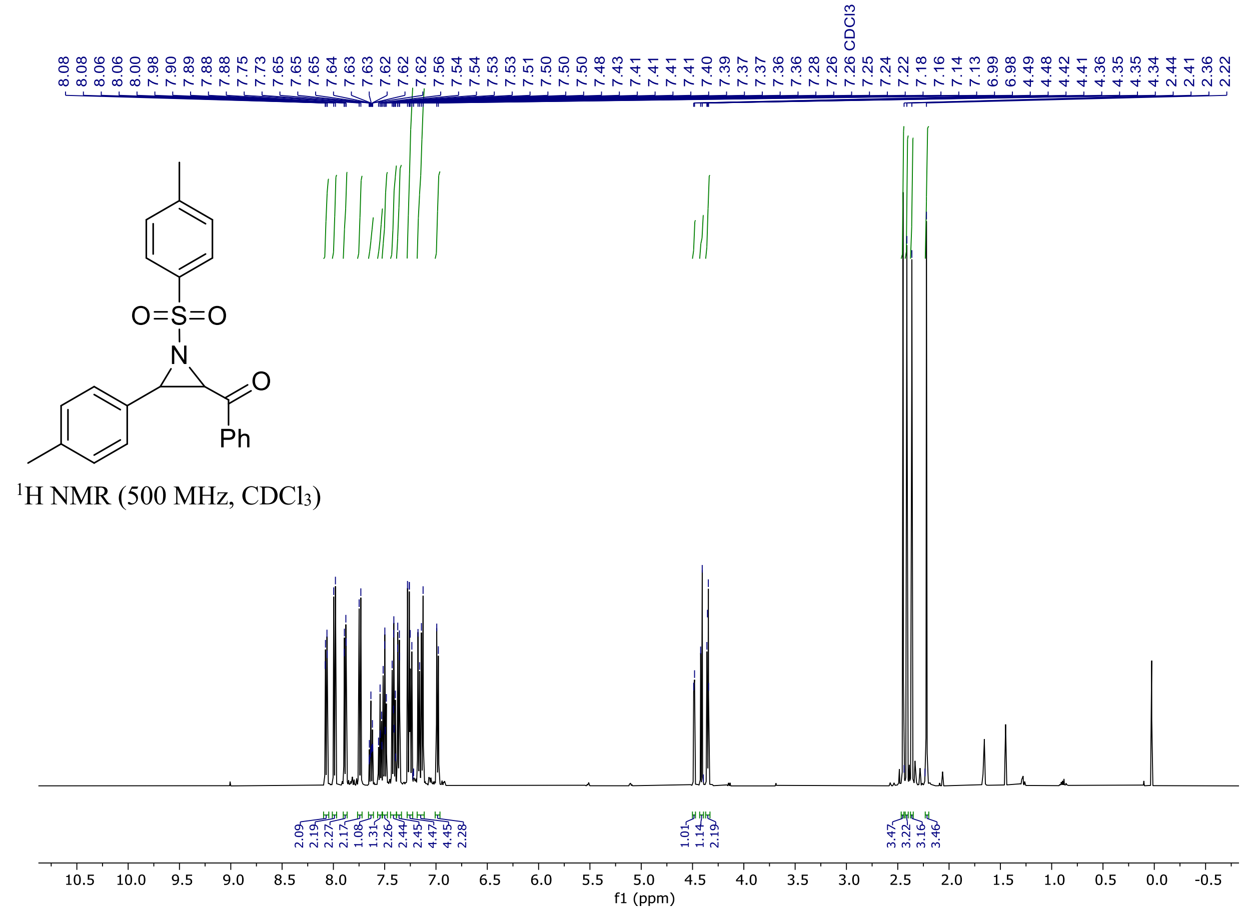

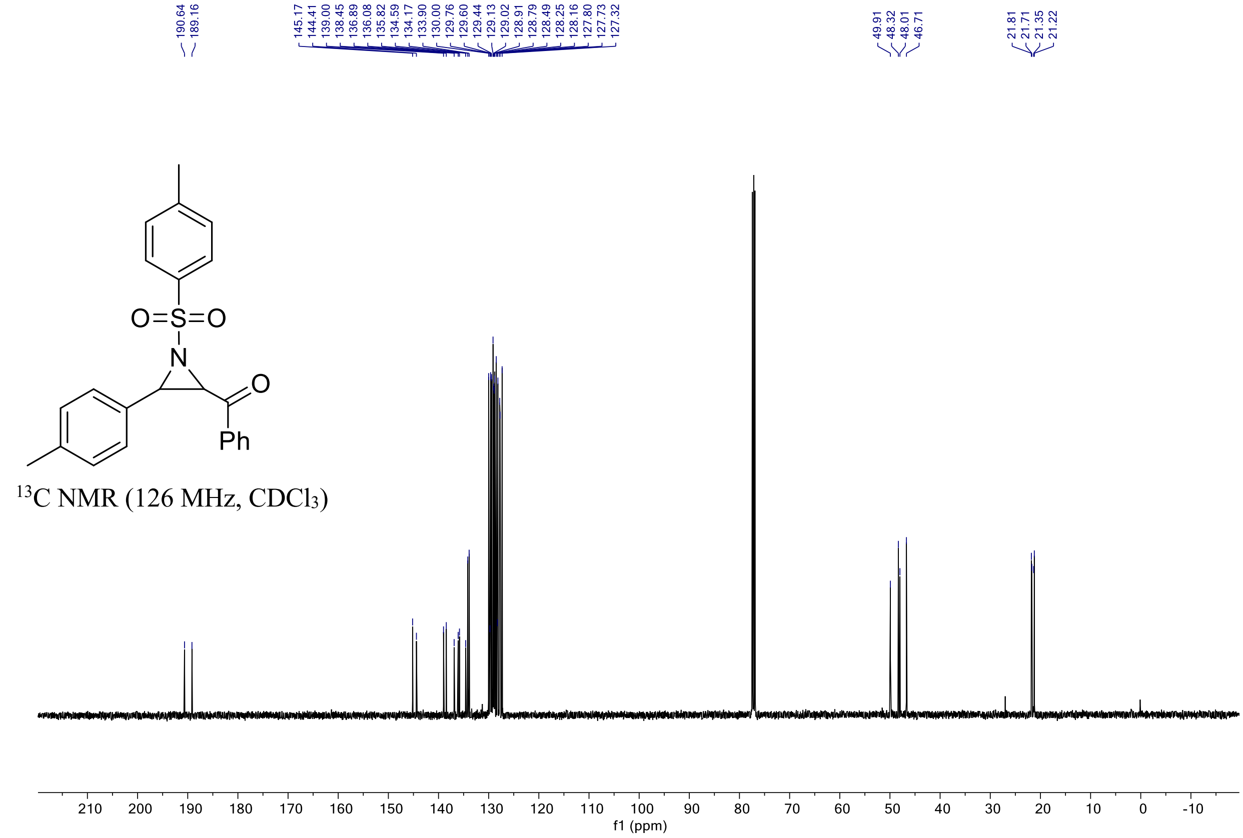

Phenyl(3-(*o*-tolyl)-1-tosylaziridin-2-yl)methanone (1x)

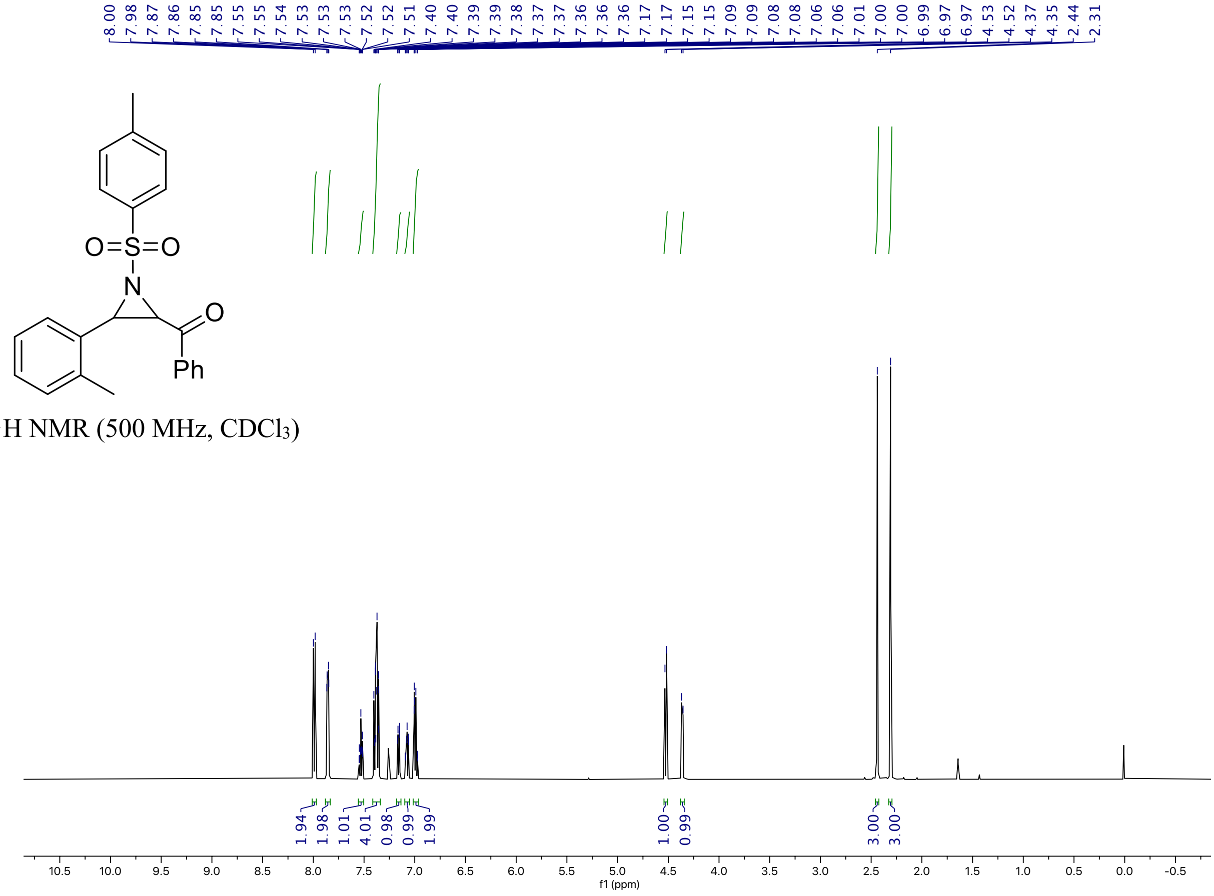

^{1}H NMR (500 MHz, $CDCl_3$)

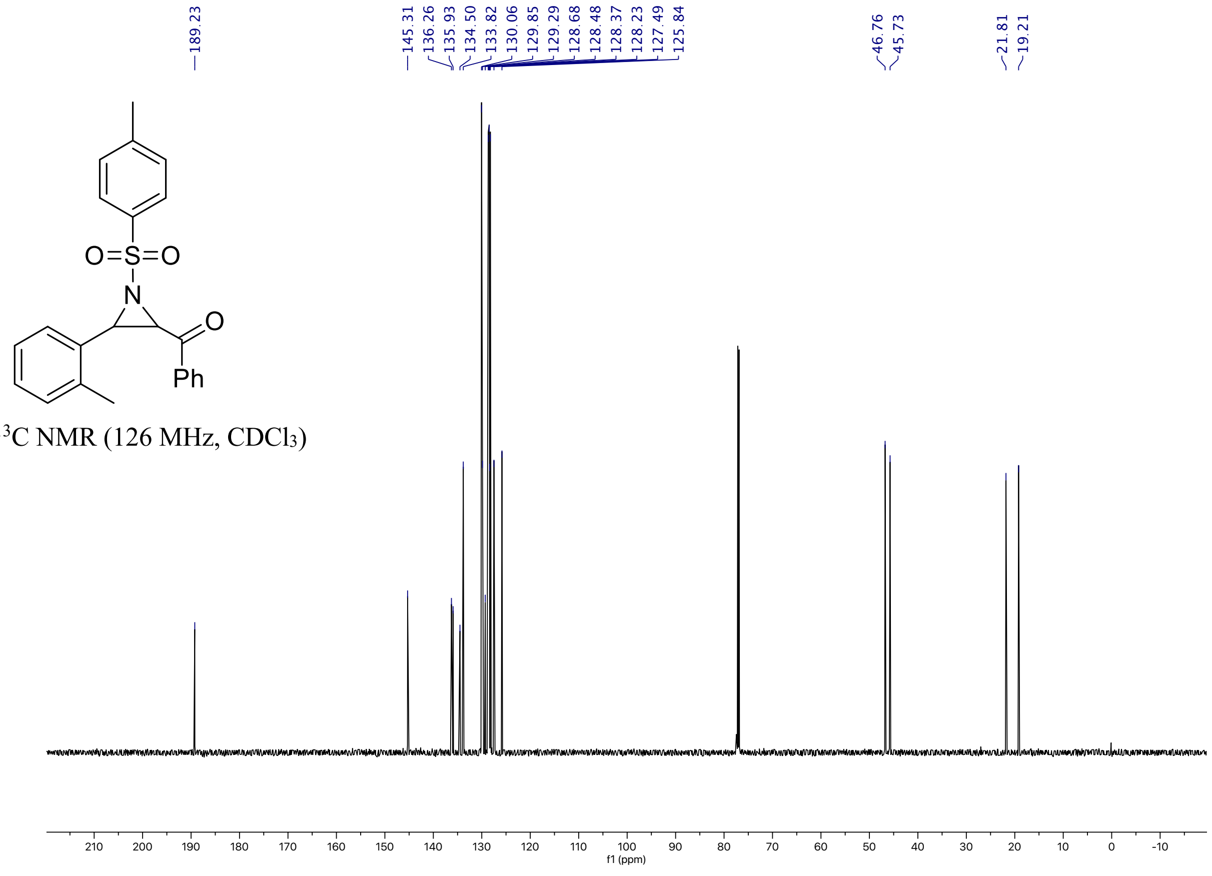

^{13}C NMR (126 MHz, $CDCl_3$)

(3-(3-Methoxyphenyl)-1-tosylaziridin-2-yl)(phenyl)methanone (1y)

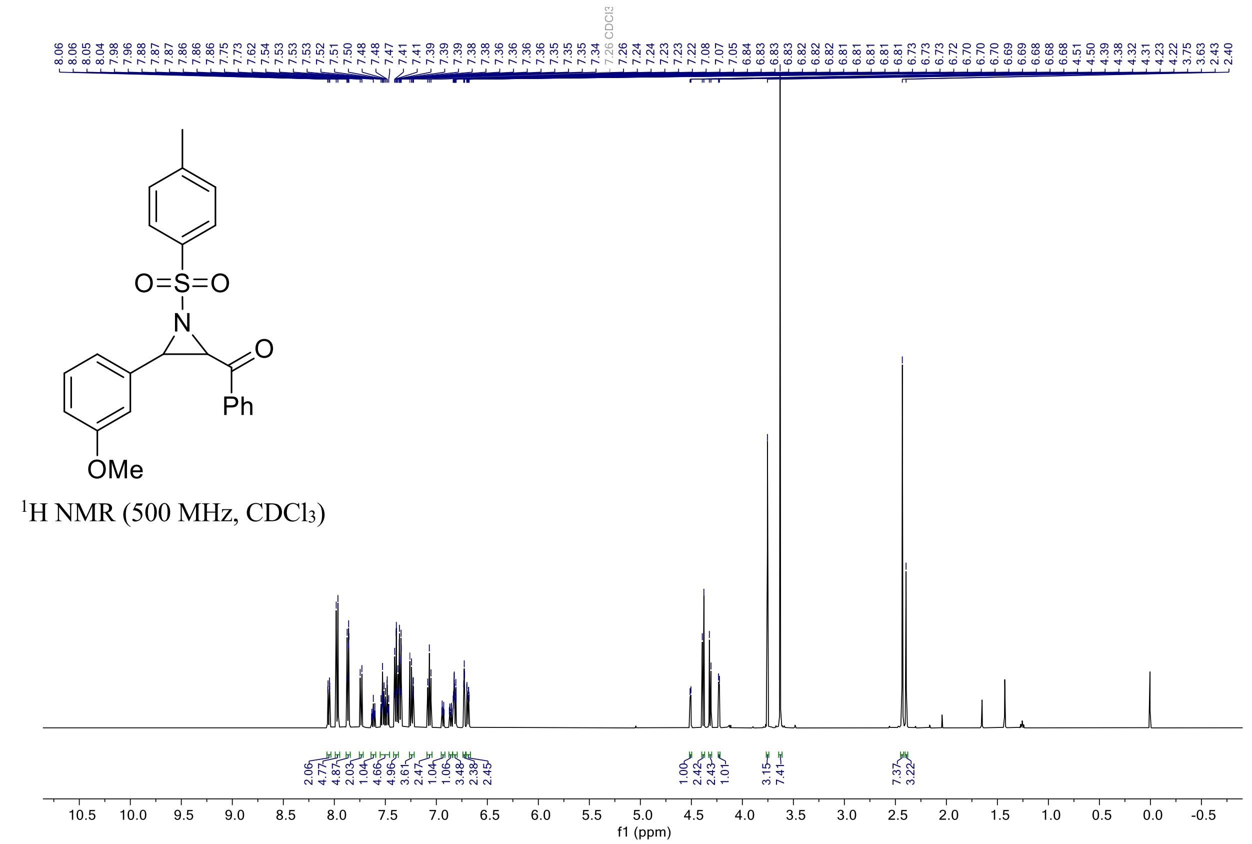

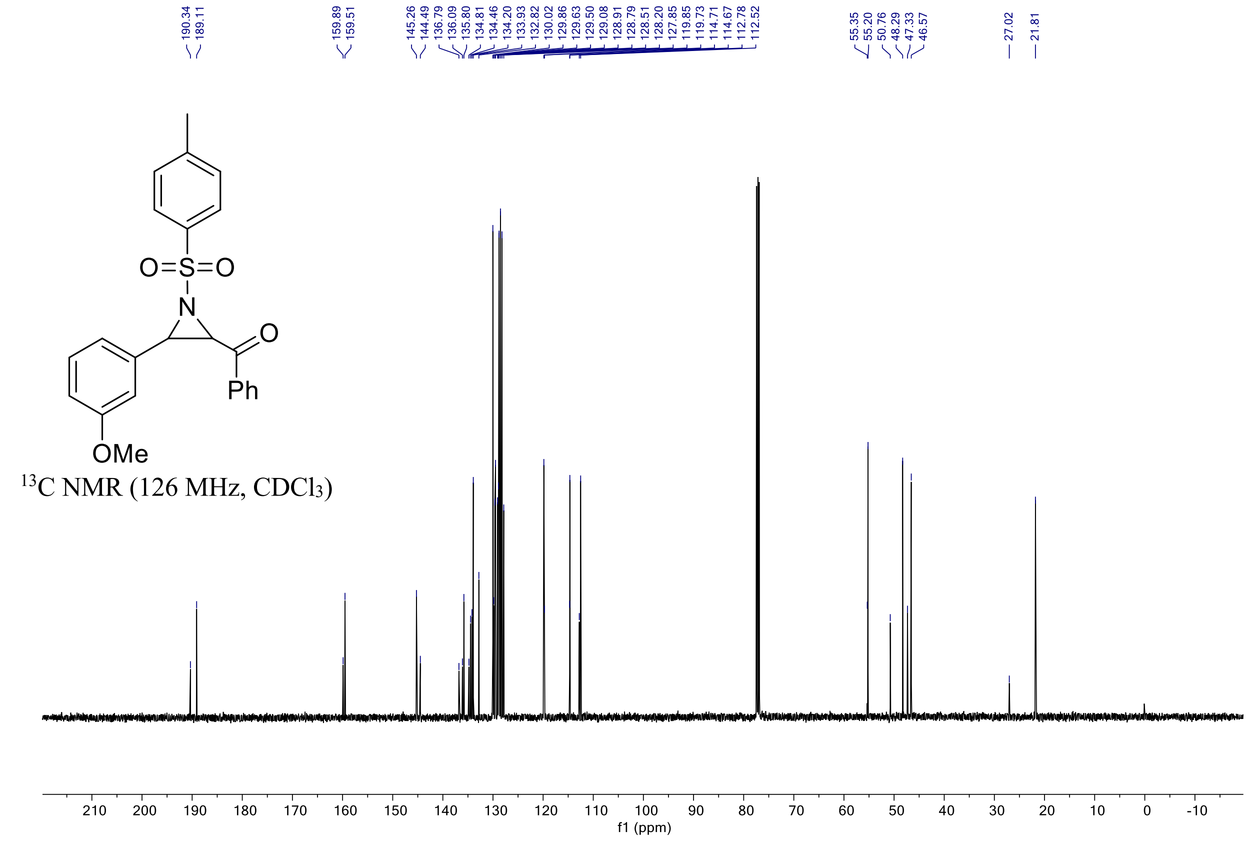

(3-(4-Fluorophenyl)-1-tosylaziridin-2-yl)(phenyl)methanone (1z)

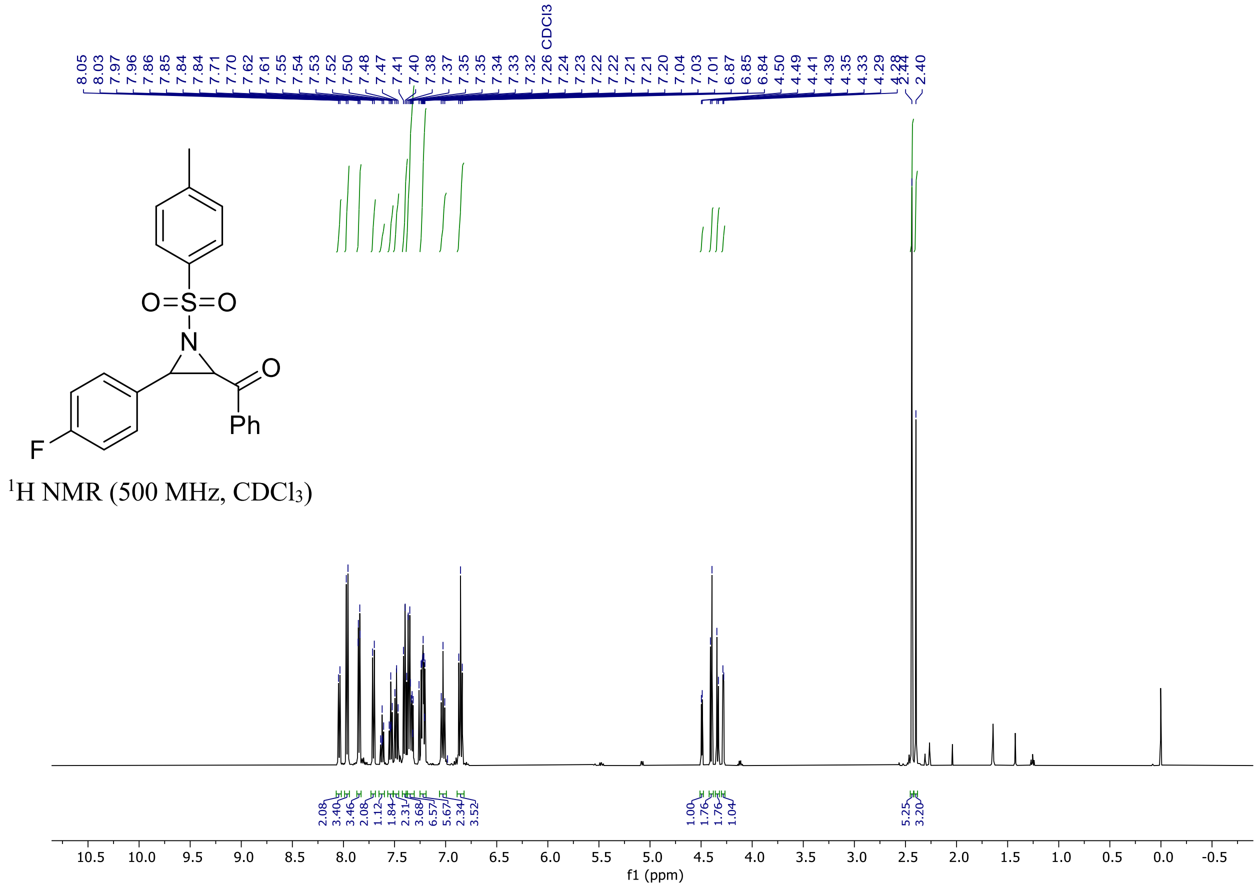

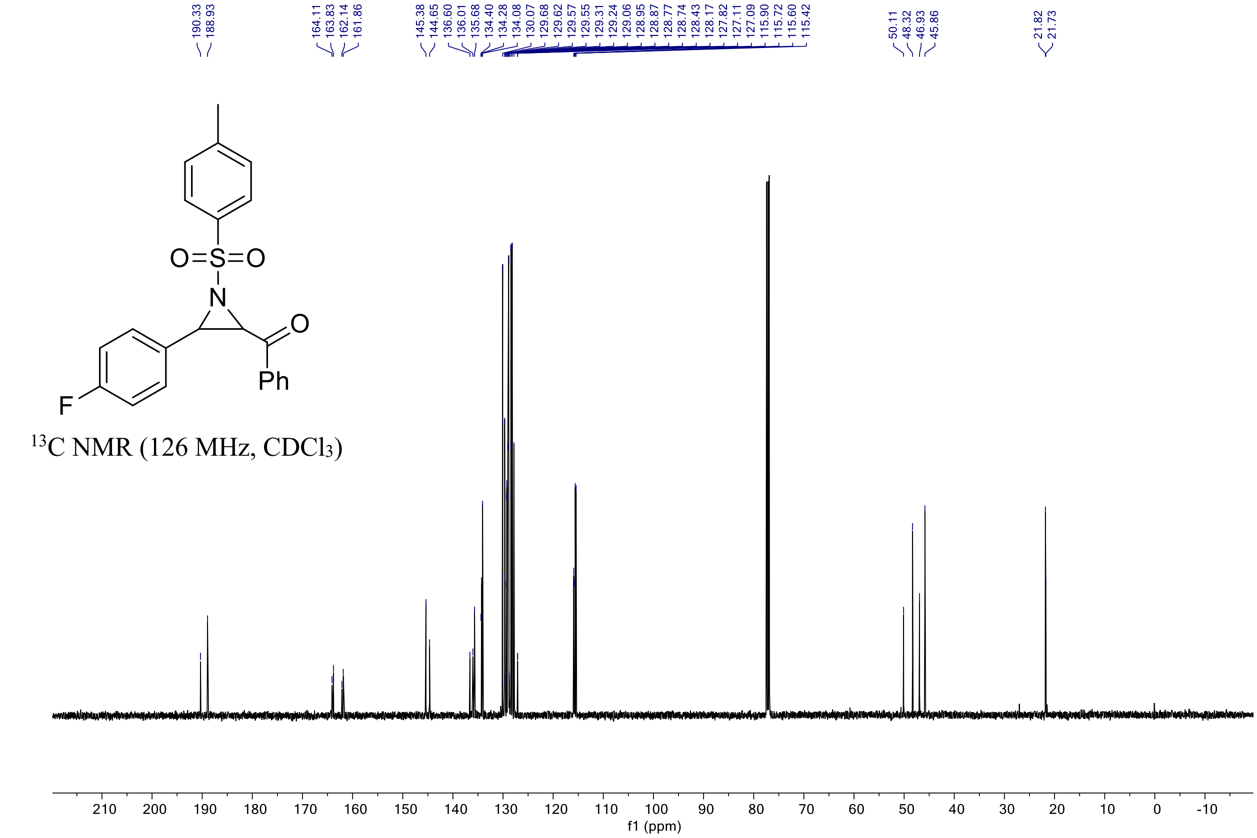

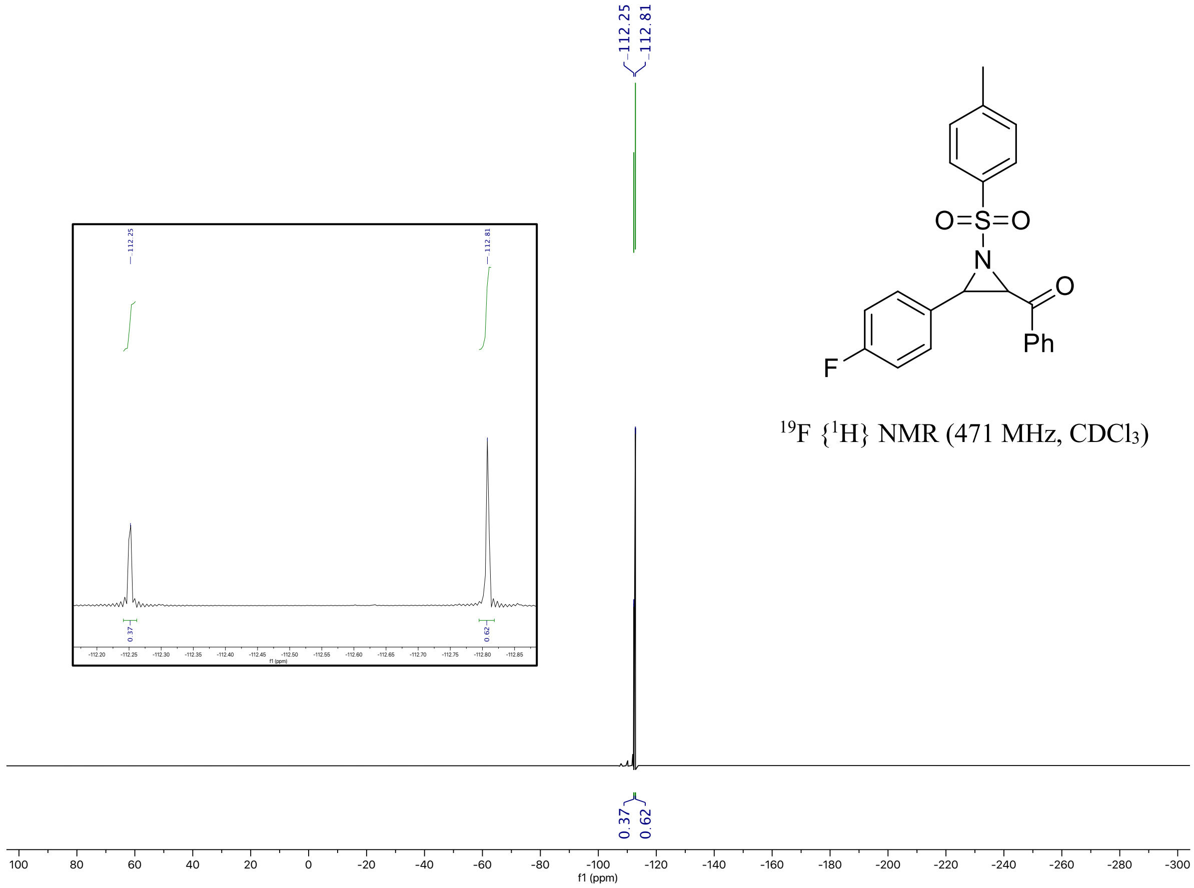

$^{19}F\{^{1}H\}$ NMR (471 MHz, $CDCl_3$)

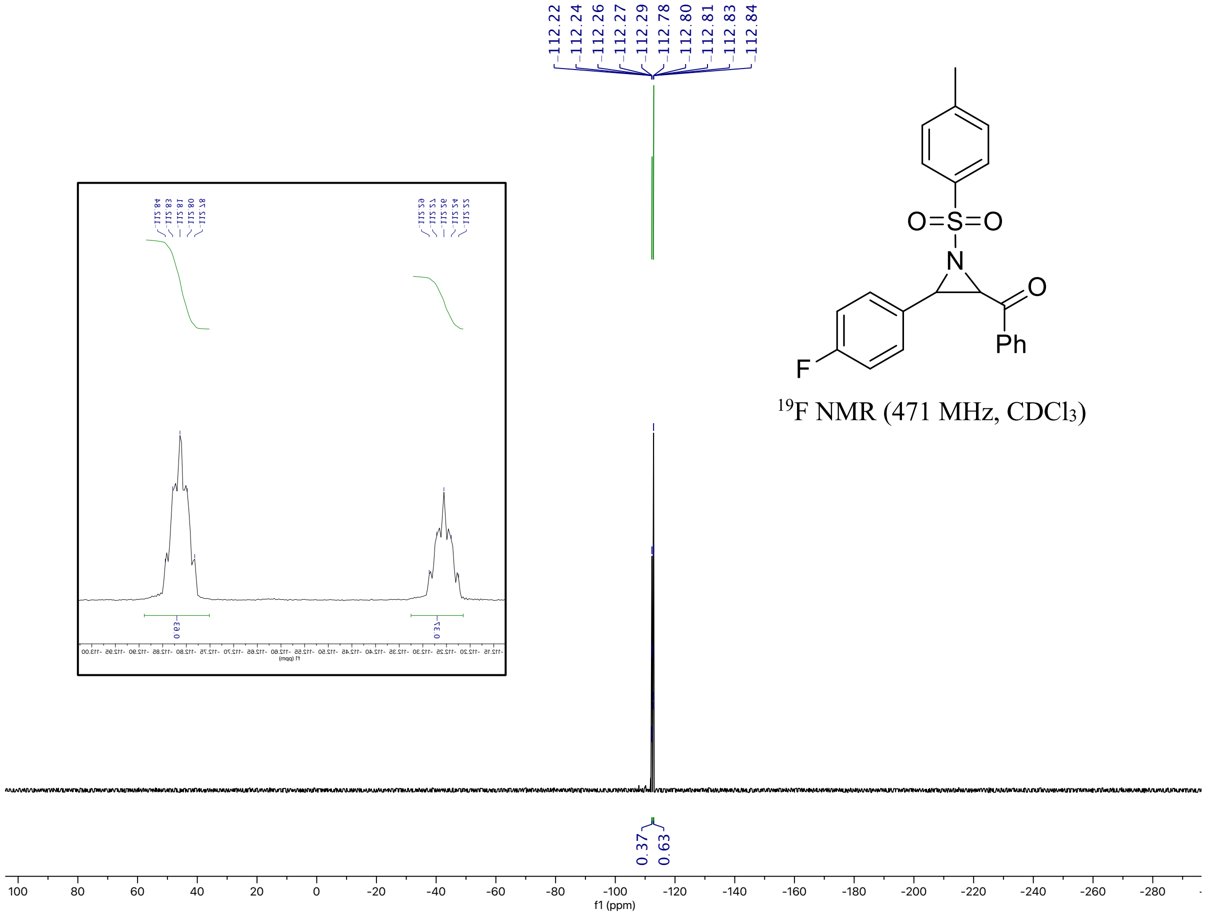

^{19}F NMR (471 MHz, $CDCl_3$)

(3-(4-Chlorophenyl)-1-tosylaziridin-2-yl)(phenyl)methanone (1za)

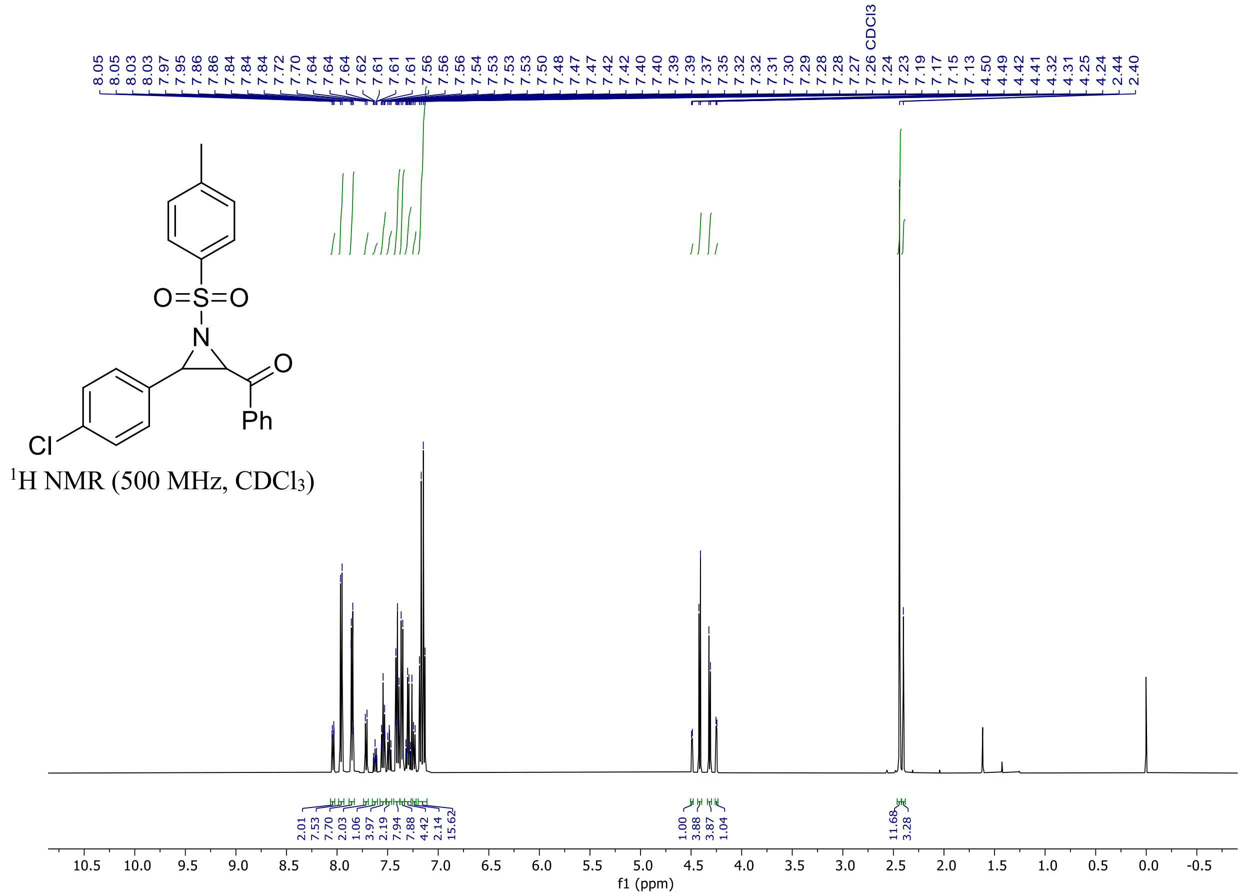

^{1}H NMR (500 MHz, $CDCl_3$)

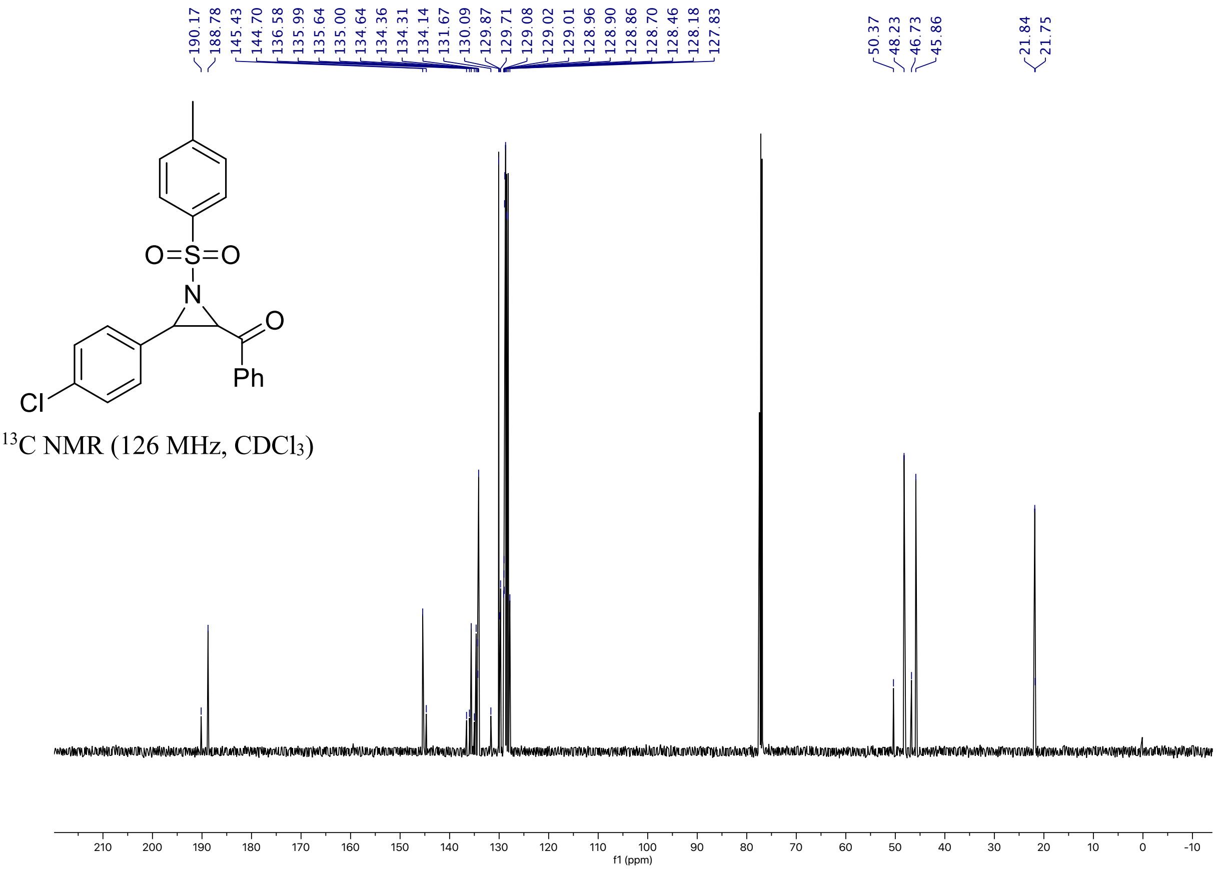

^{13}C NMR (126 MHz, $CDCl_3$)

(3-(2-Chlorophenyl)-1-tosylaziridin-2-yl)(phenyl)methanone (1zb)

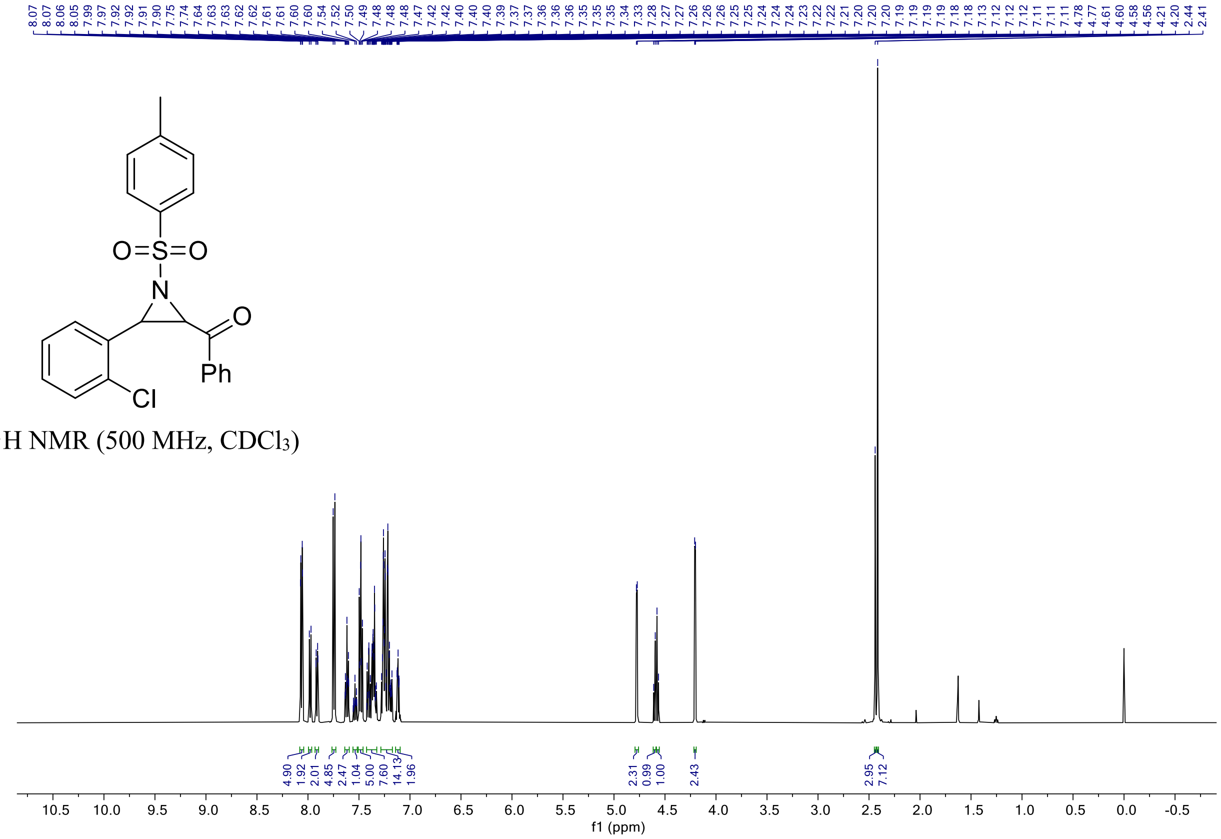

^{1}H NMR (500 MHz, $CDCl_3$)

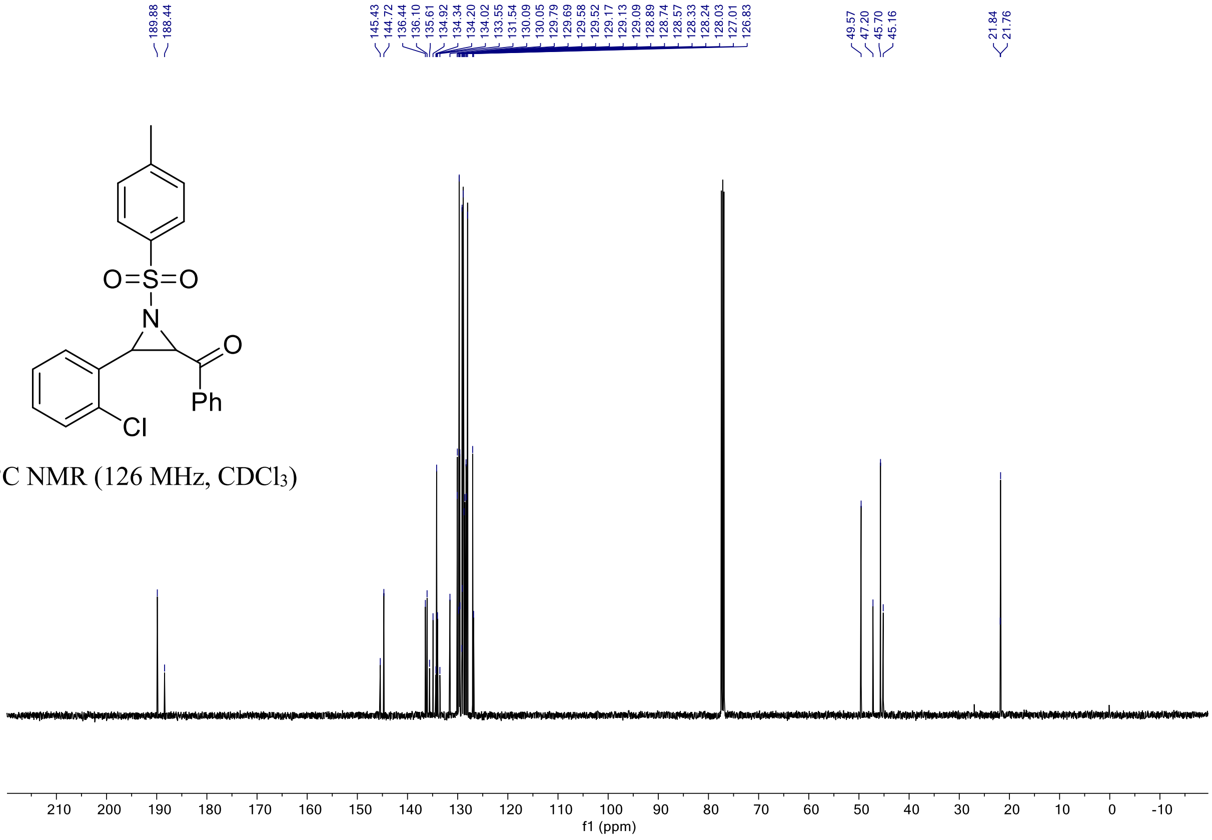

^{13}C NMR (126 MHz, $CDCl_3$)

Phenyl(1-tosylaziridin-2-yl)methanone (1zc)

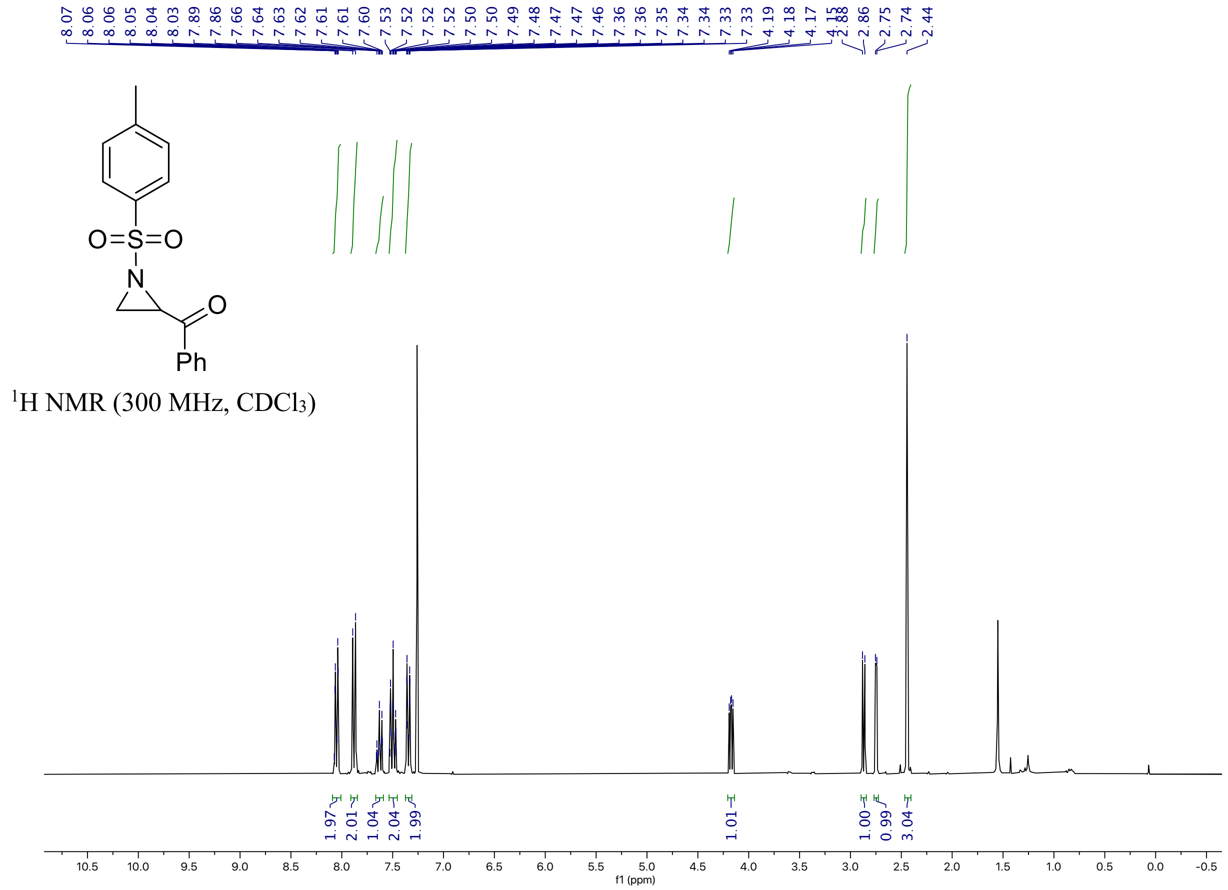

^{1}H NMR (300 MHz, CDCl$_3$)

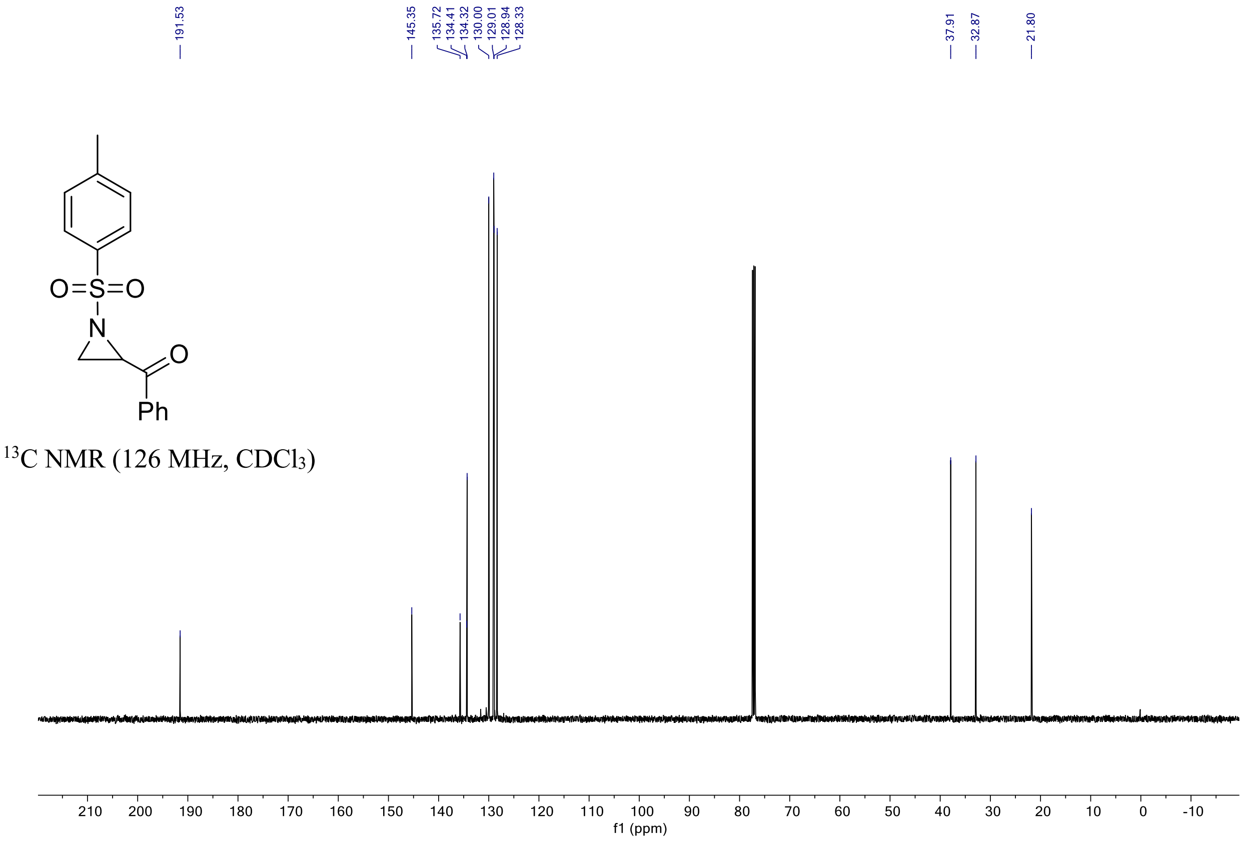

^{13}C NMR (126 MHz, CDCl$_3$)

(3-Methyl-1-tosylaziridin-2-yl)(phenyl)methanone (1zd)

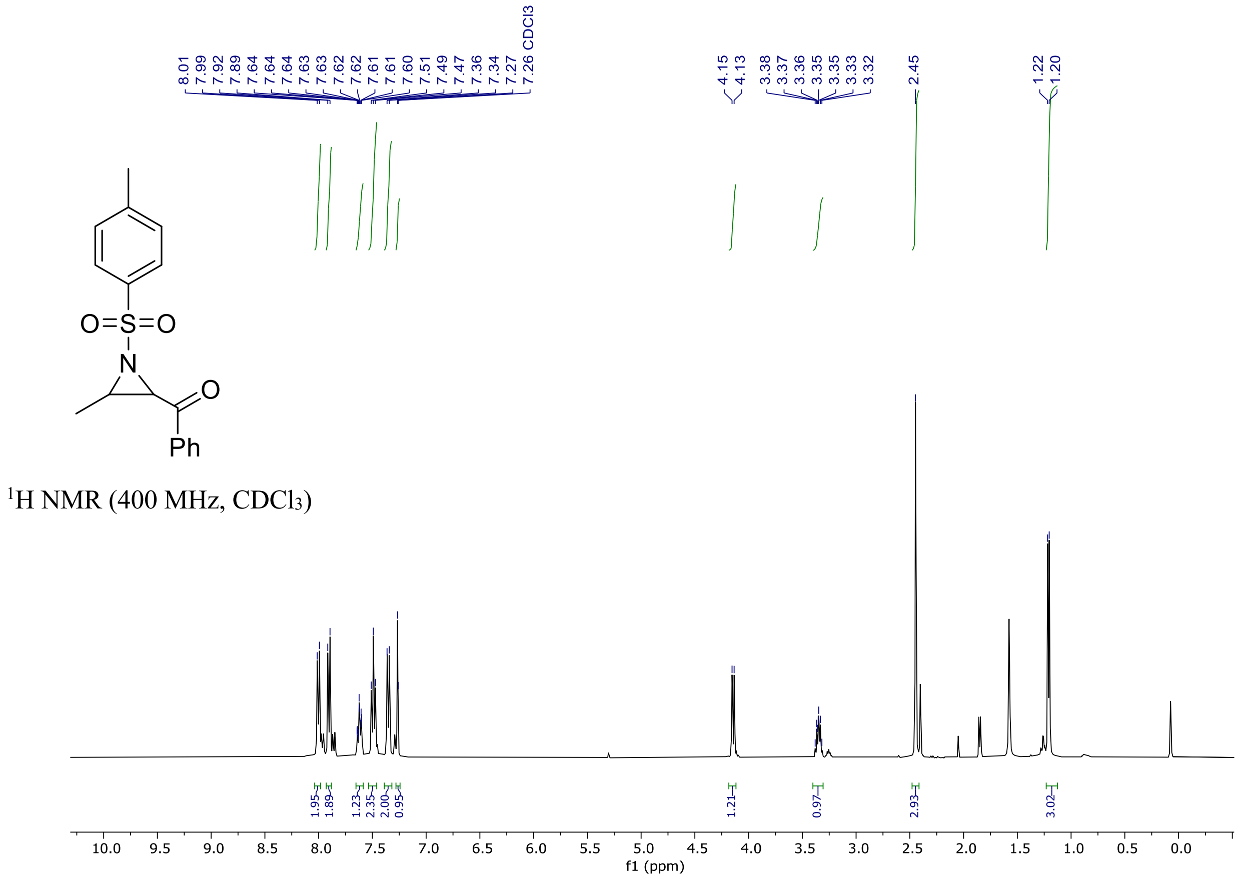

^{1}H NMR (400 MHz, $CDCl_3$)

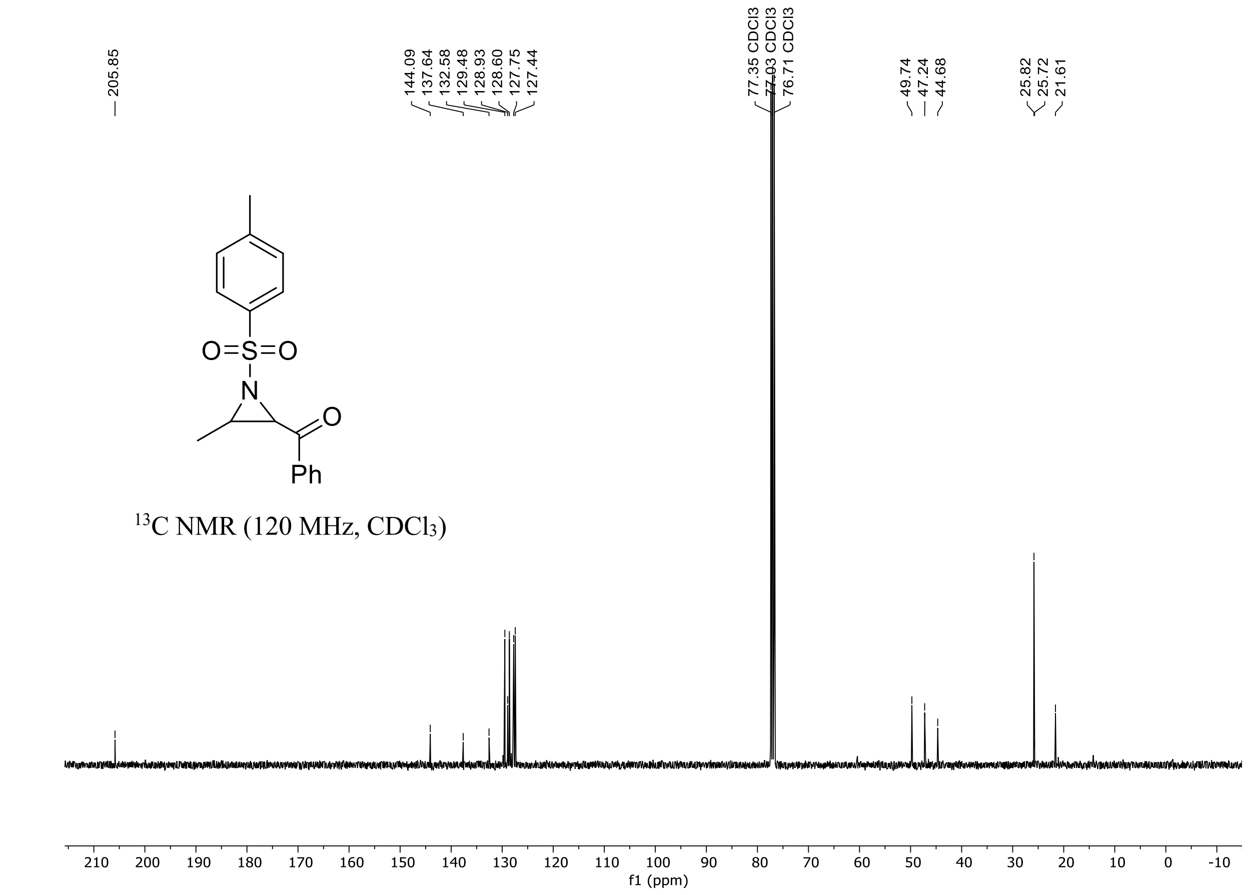

^{13}C NMR (120 MHz, $CDCl_3$)

2-Phenyl-1-tosyl-1a,7b-dihydro-1*H*-azirino[2,3-c]quinoline (1ze)

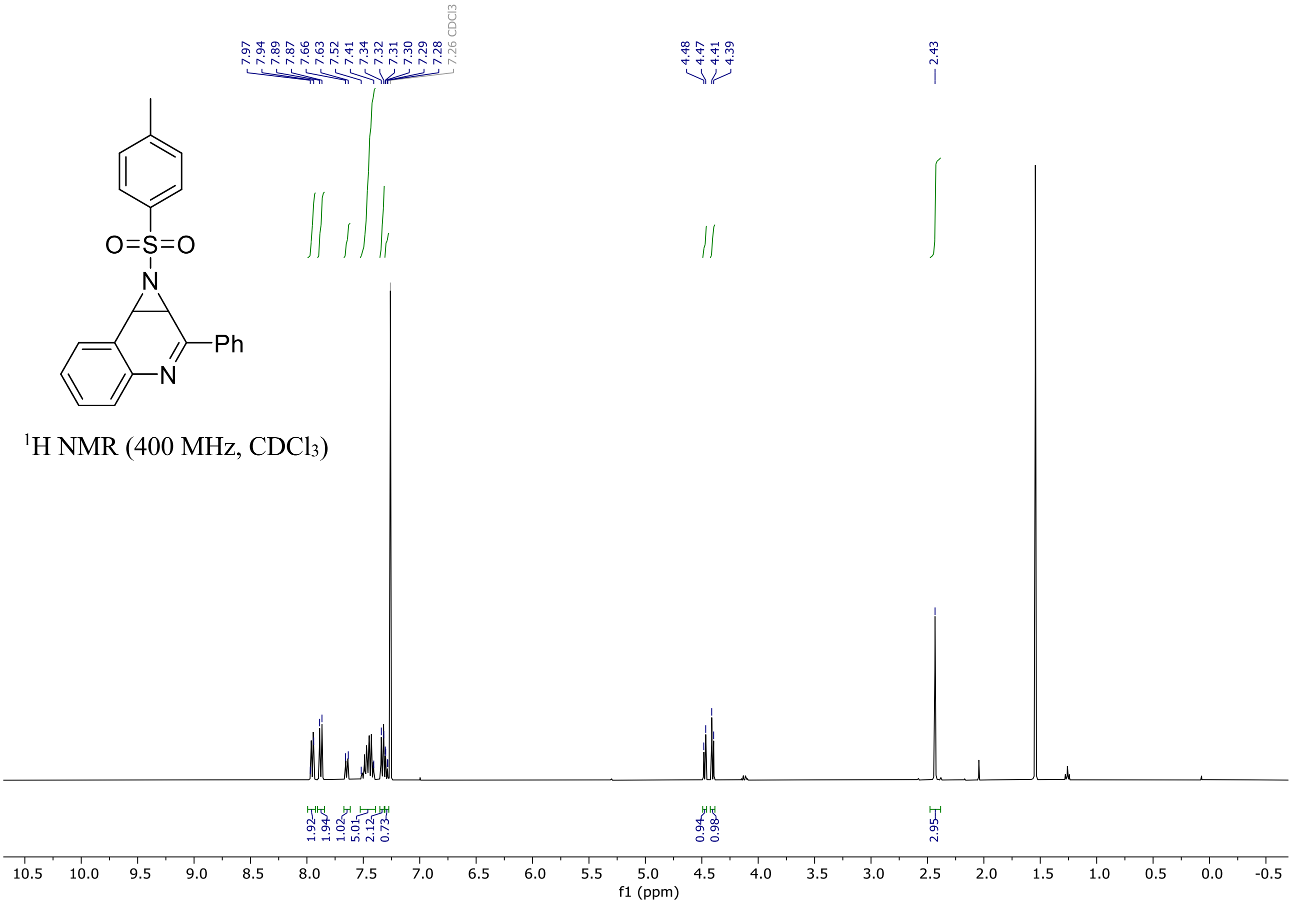

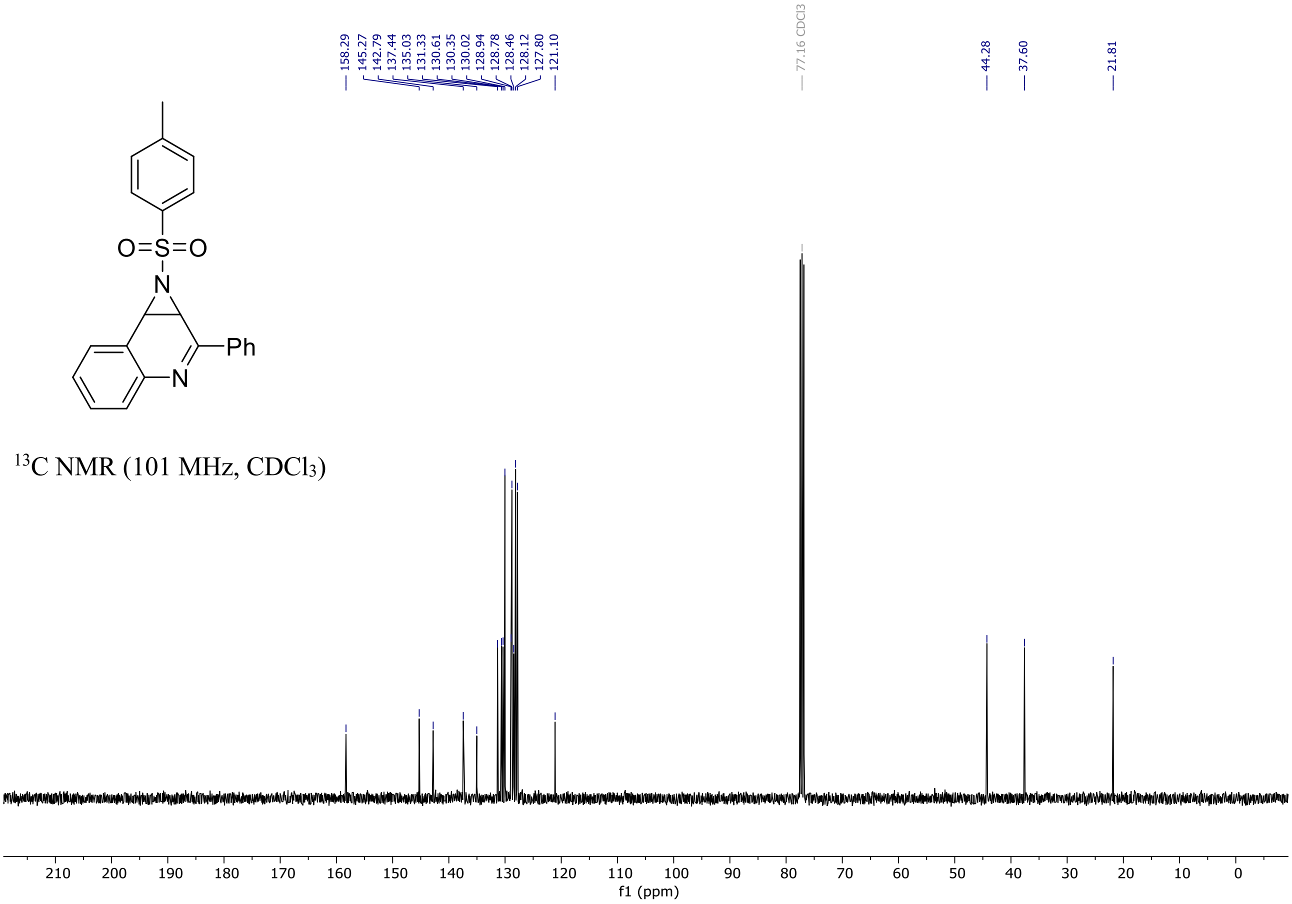

(6-Methyl-1,1-dioxido-3-phenyl-3,4-dihydro-2*H*-benzo[*e*][1,2]thiazin-4-yl)(phenyl)methanone (2a)

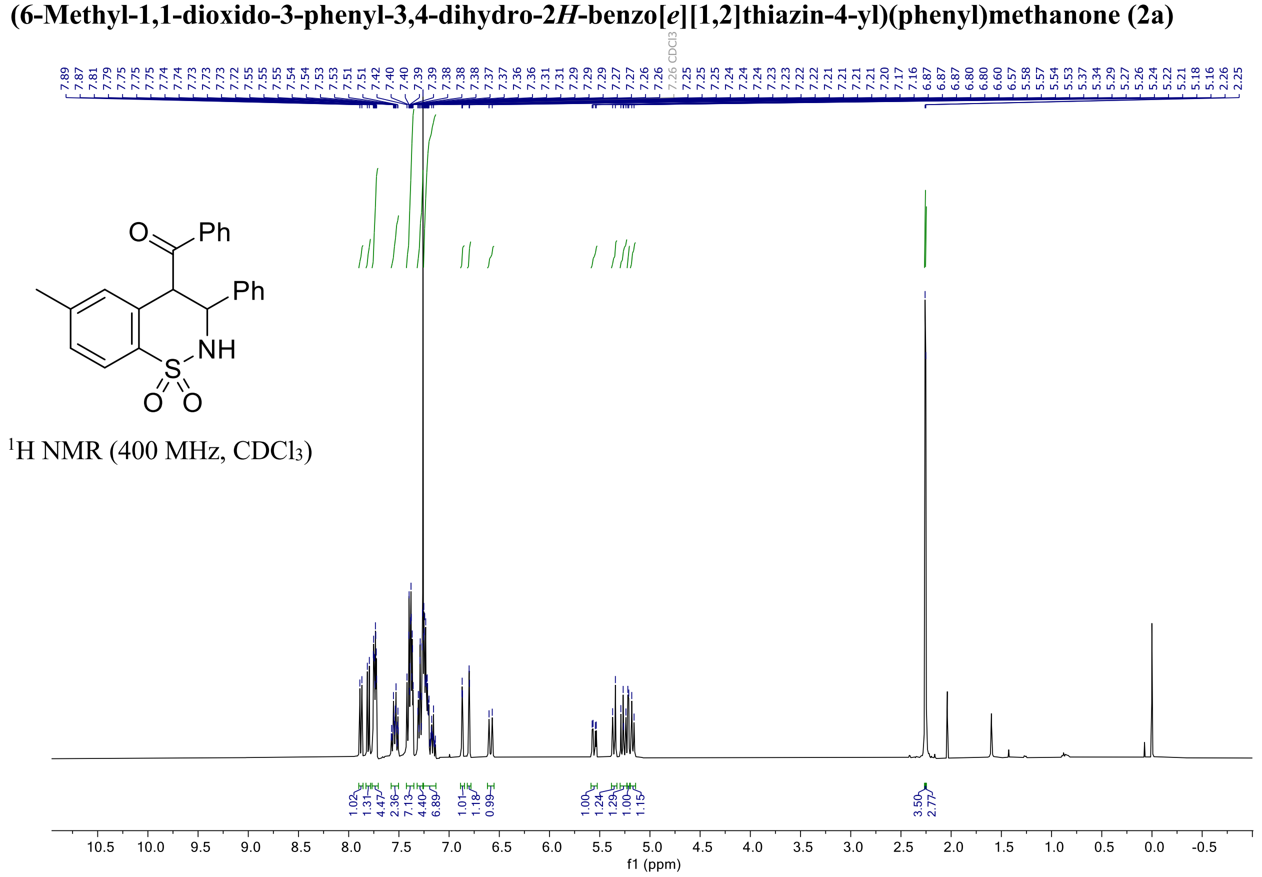

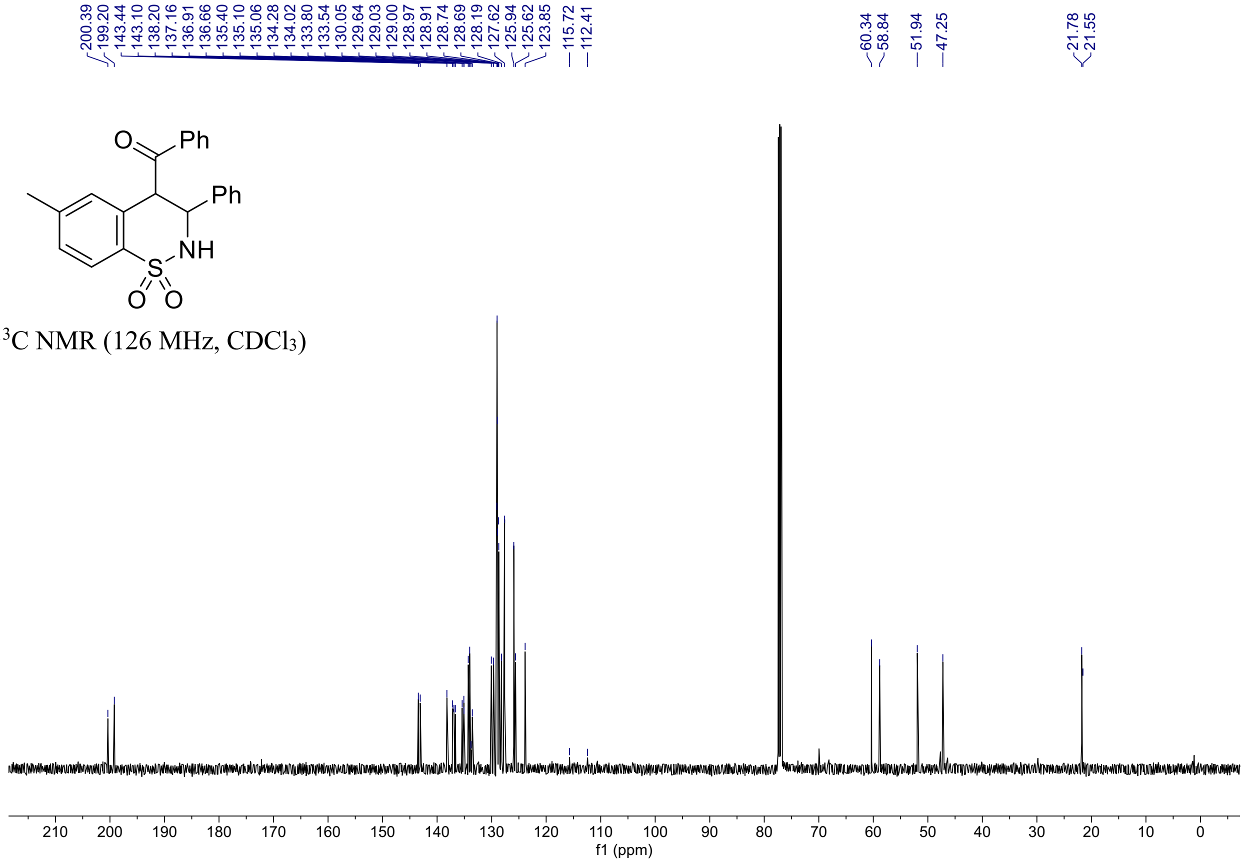

***trans*-(6-Methyl-1,1-dioxido-3-phenyl-3,4-dihydro-2*H*-benzo[*e*][1,2]thiazin-4-yl)(phenyl)methanone (*trans*-2a)**

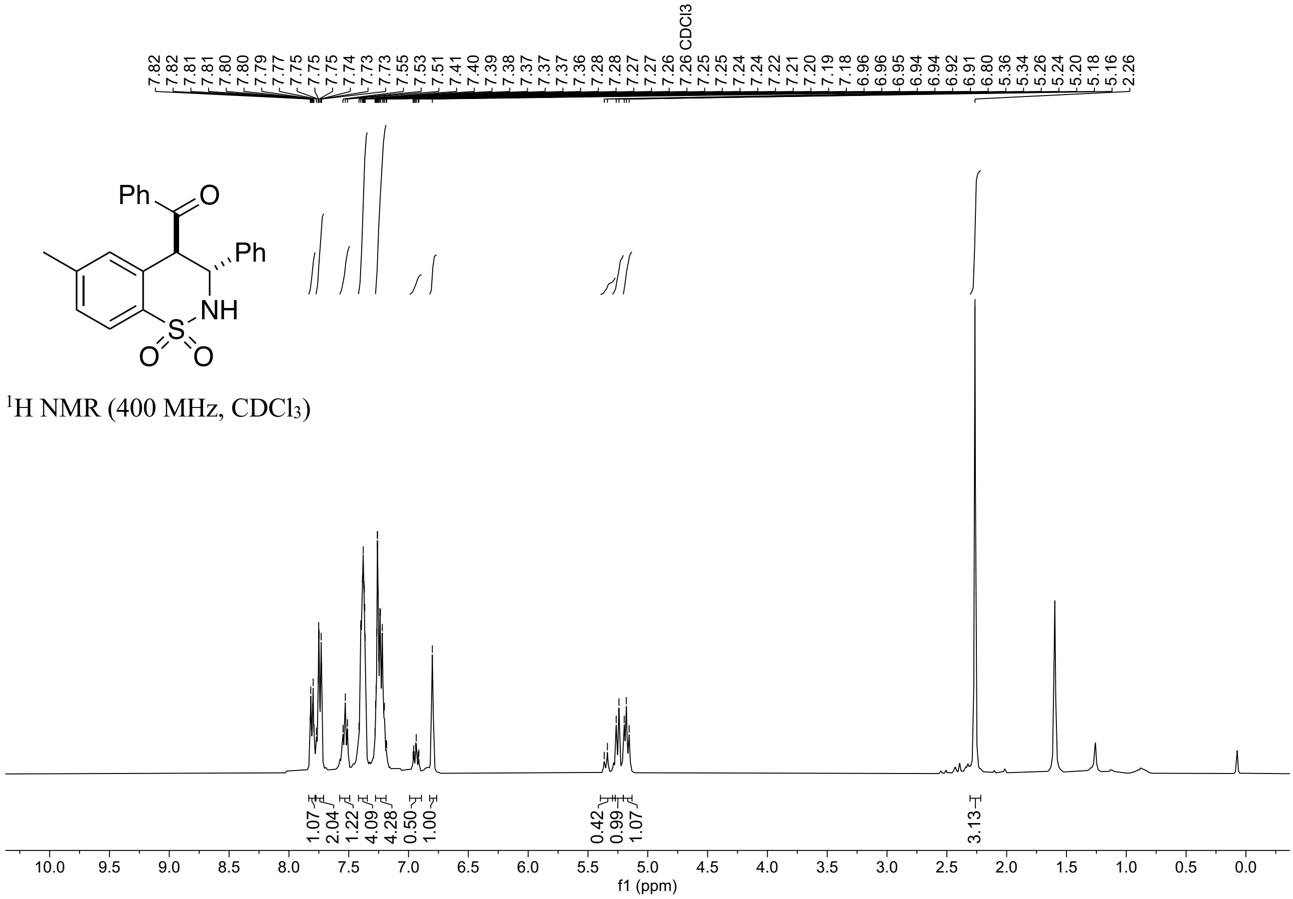

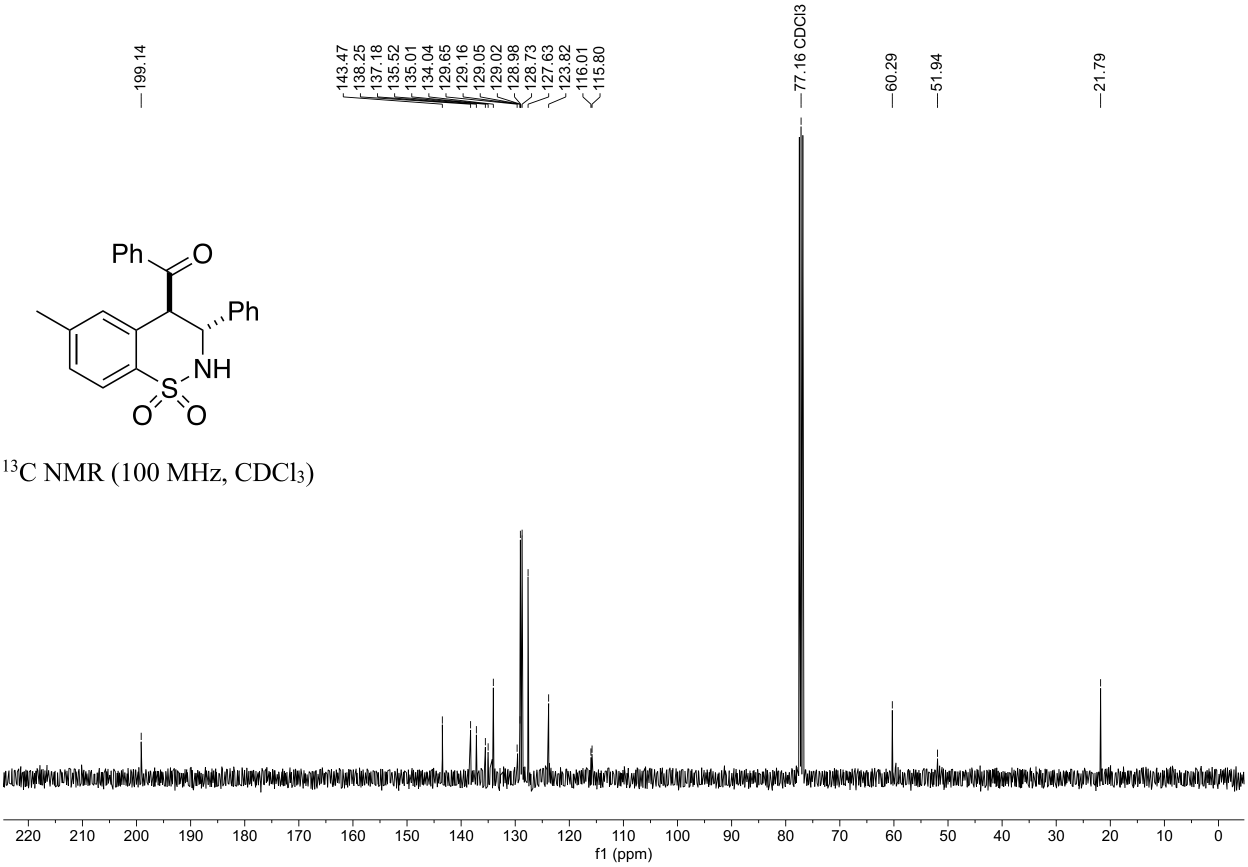

(1,1-Dioxido-3-phenyl-3,4-dihydro-2*H*-benzo[*e*][1,2]thiazin-4-yl)(phenyl)methanone (2b)

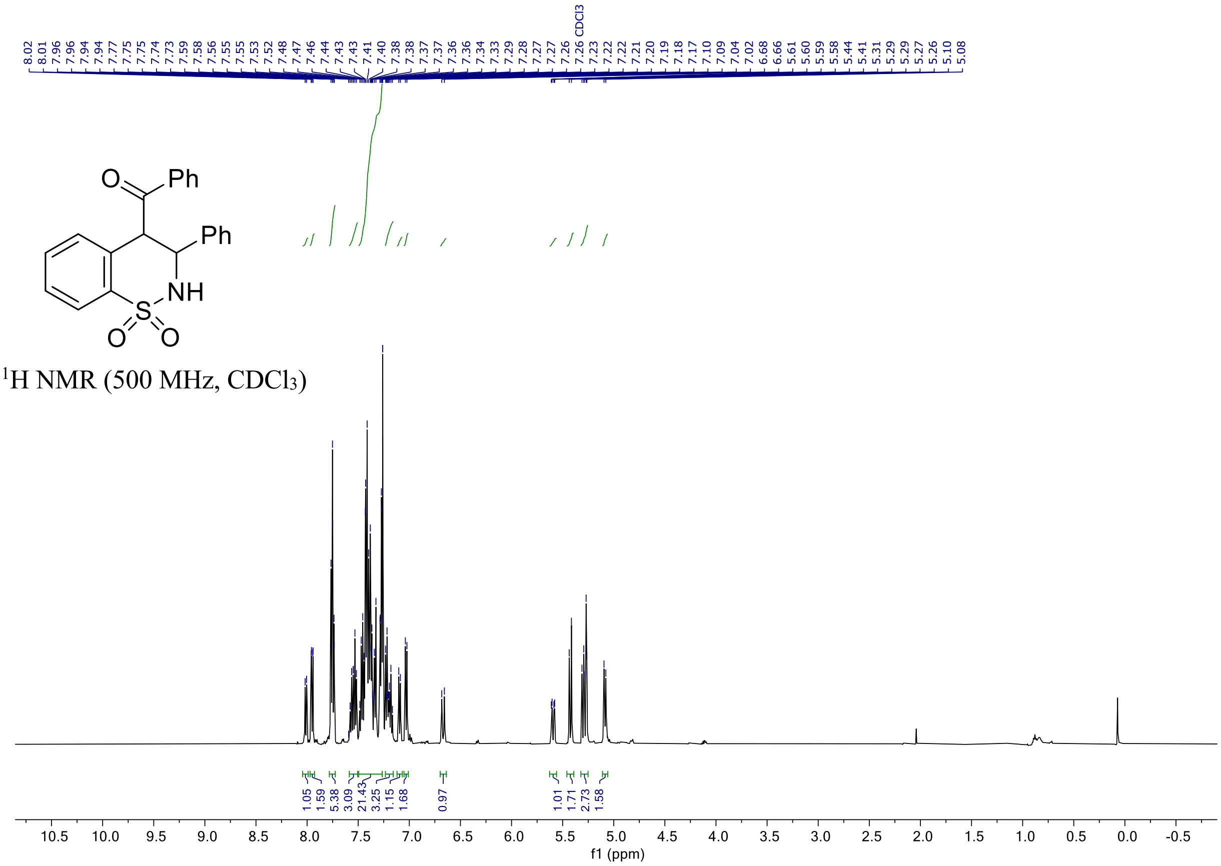

^{1}H NMR (500 MHz, $CDCl_3$)

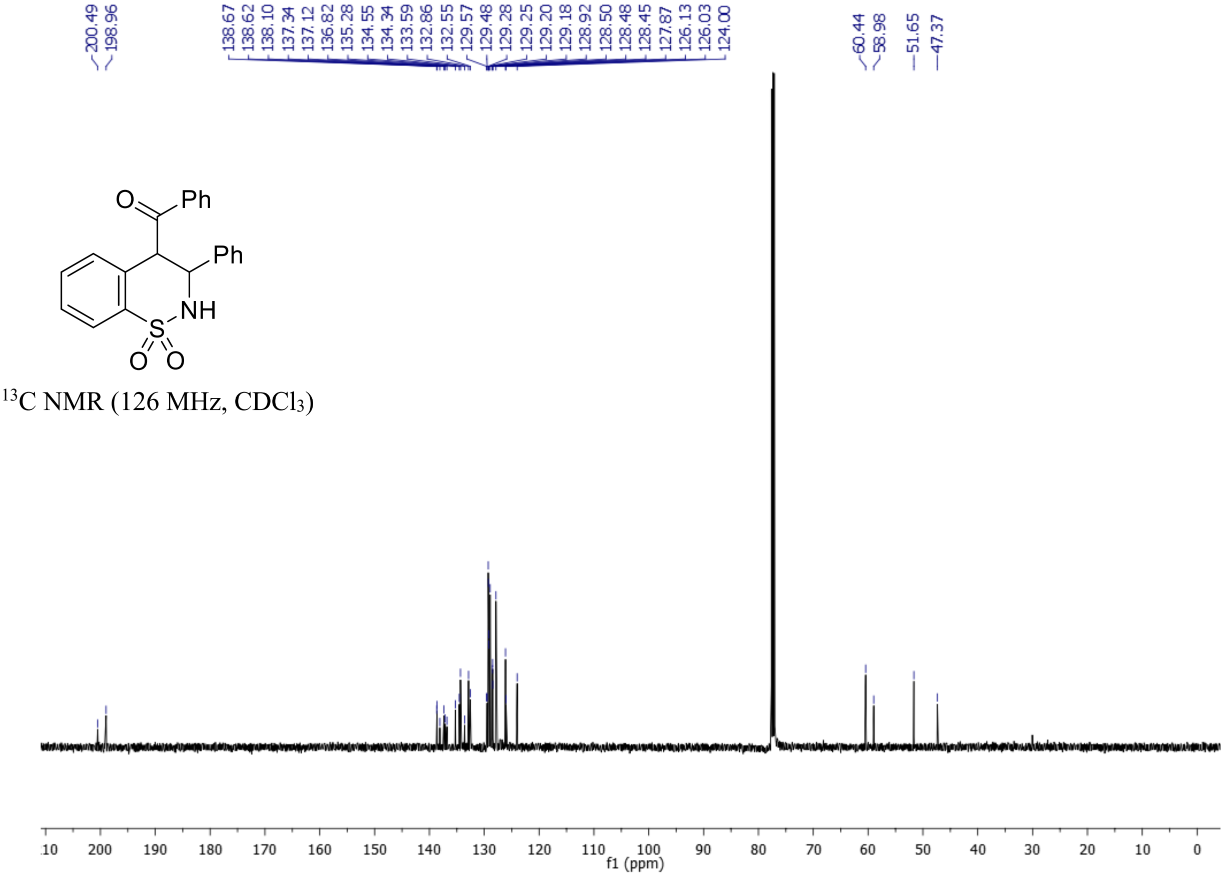

^{13}C NMR (126 MHz, $CDCl_3$)

(8-Methyl-1,1-dioxido-3-phenyl-3,4-dihydro-2*H*-benzo[*e*][1,2]thiazin-4-yl)(phenyl)methanone (2c)

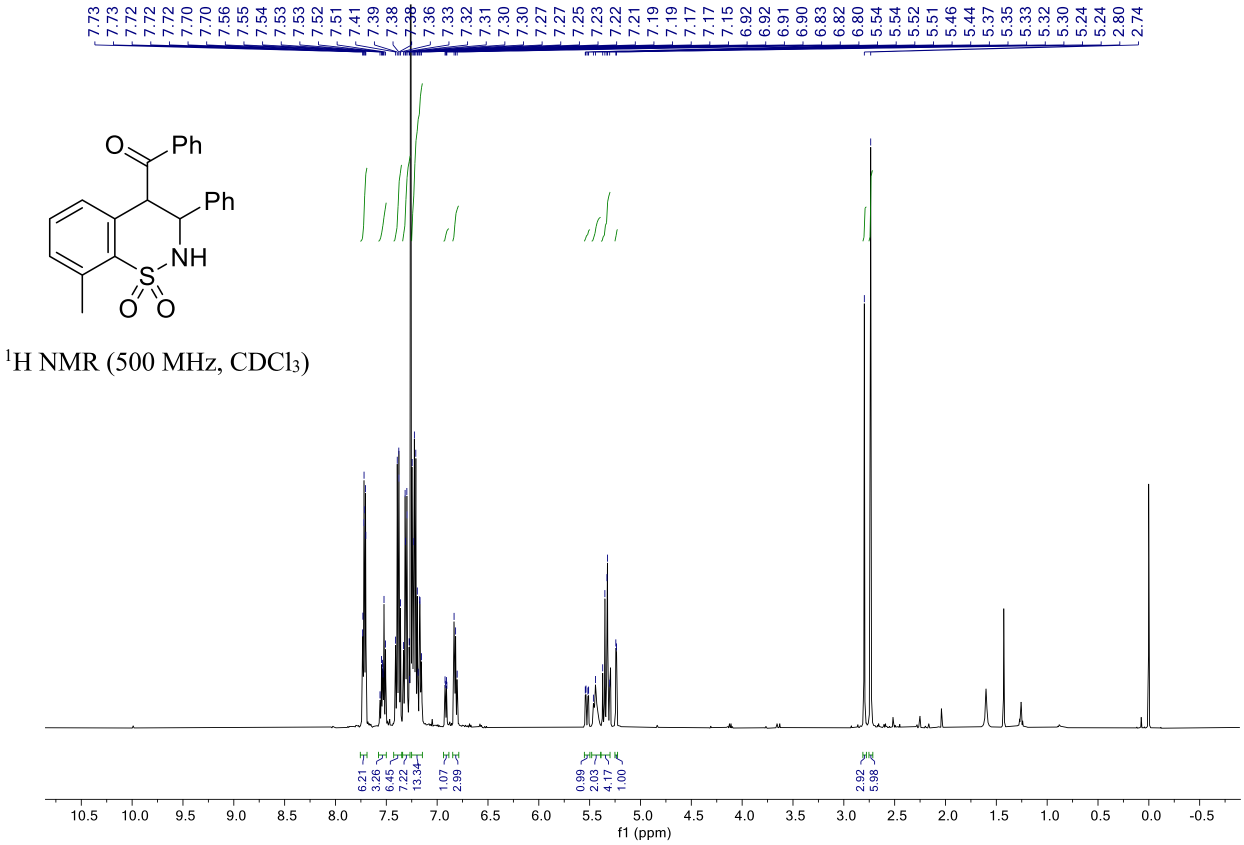

^{1}H NMR (500 MHz, $CDCl_3$)

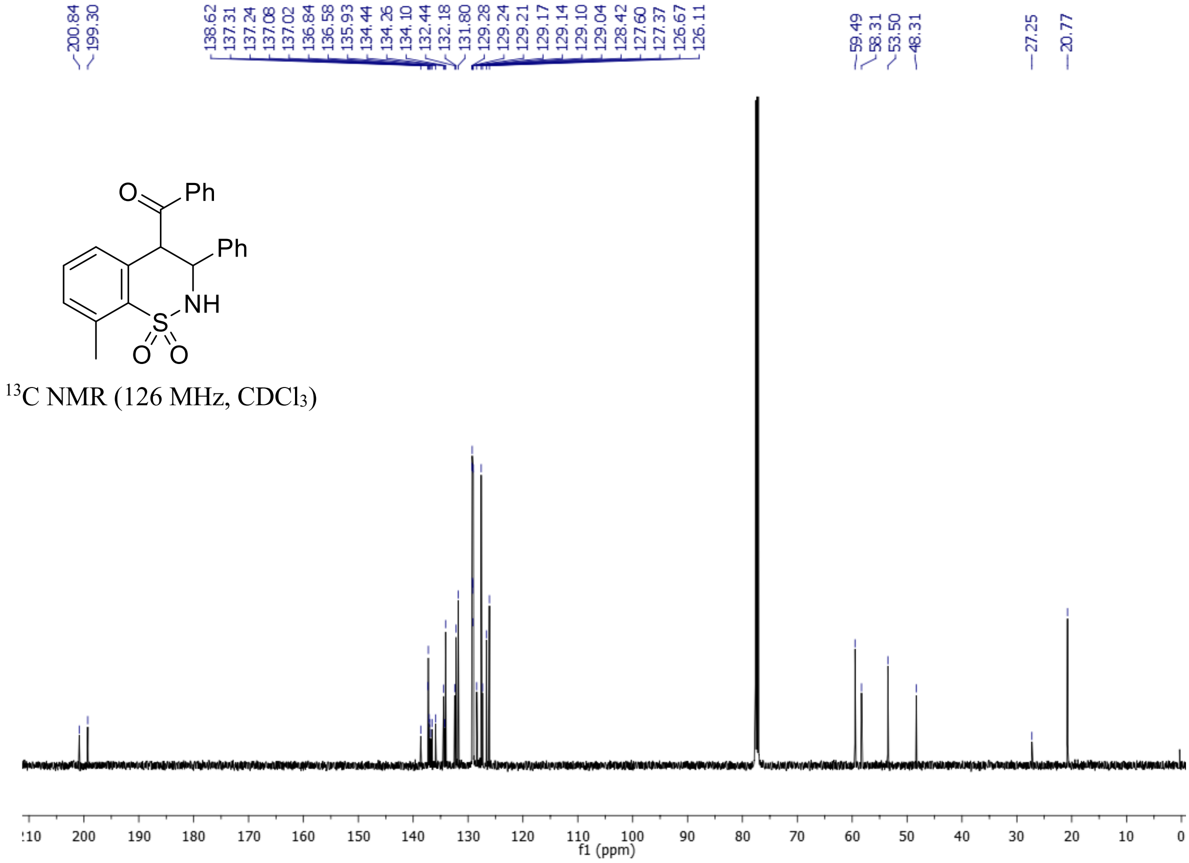

^{13}C NMR (126 MHz, $CDCl_3$)

(6-Methoxy-1,1-dioxido-3-phenyl-3,4-dihydro-2*H*-benzo[*e*][1,2]thiazin-4-yl)(phenyl)methanone (2d)

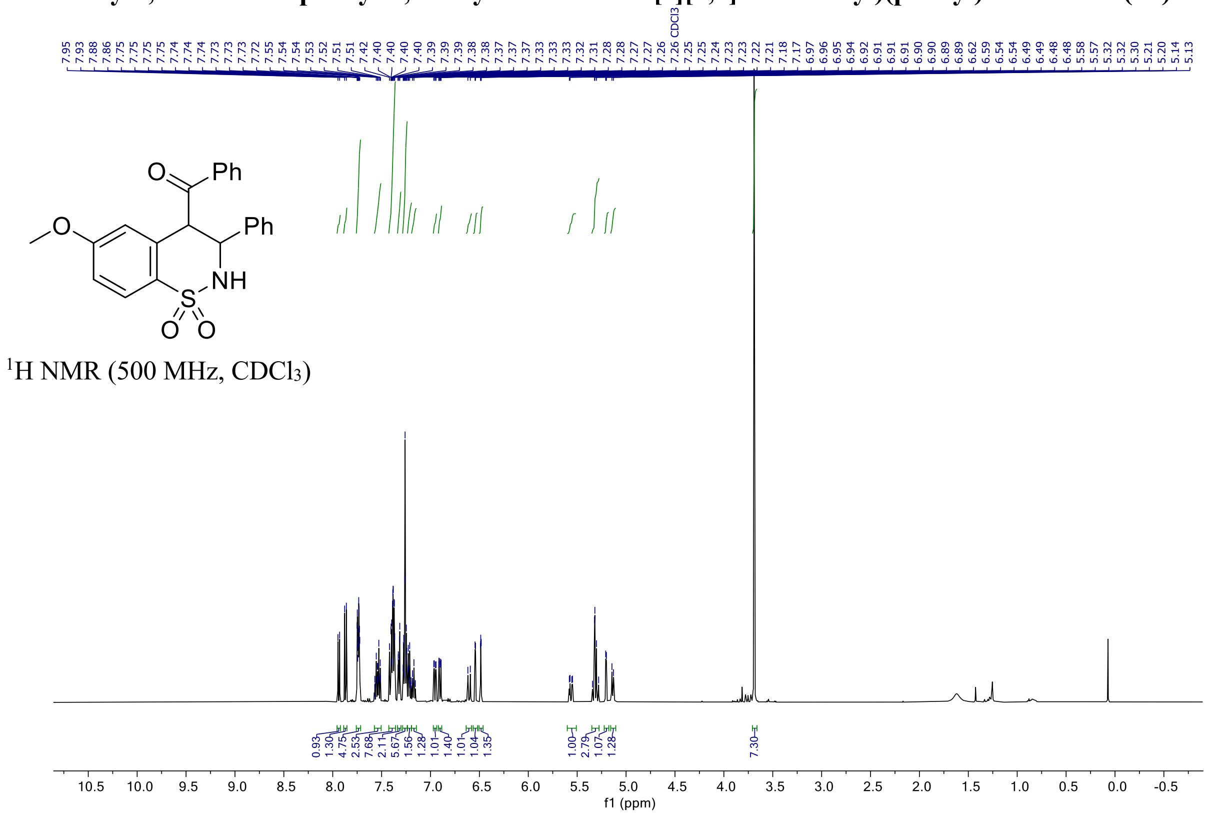

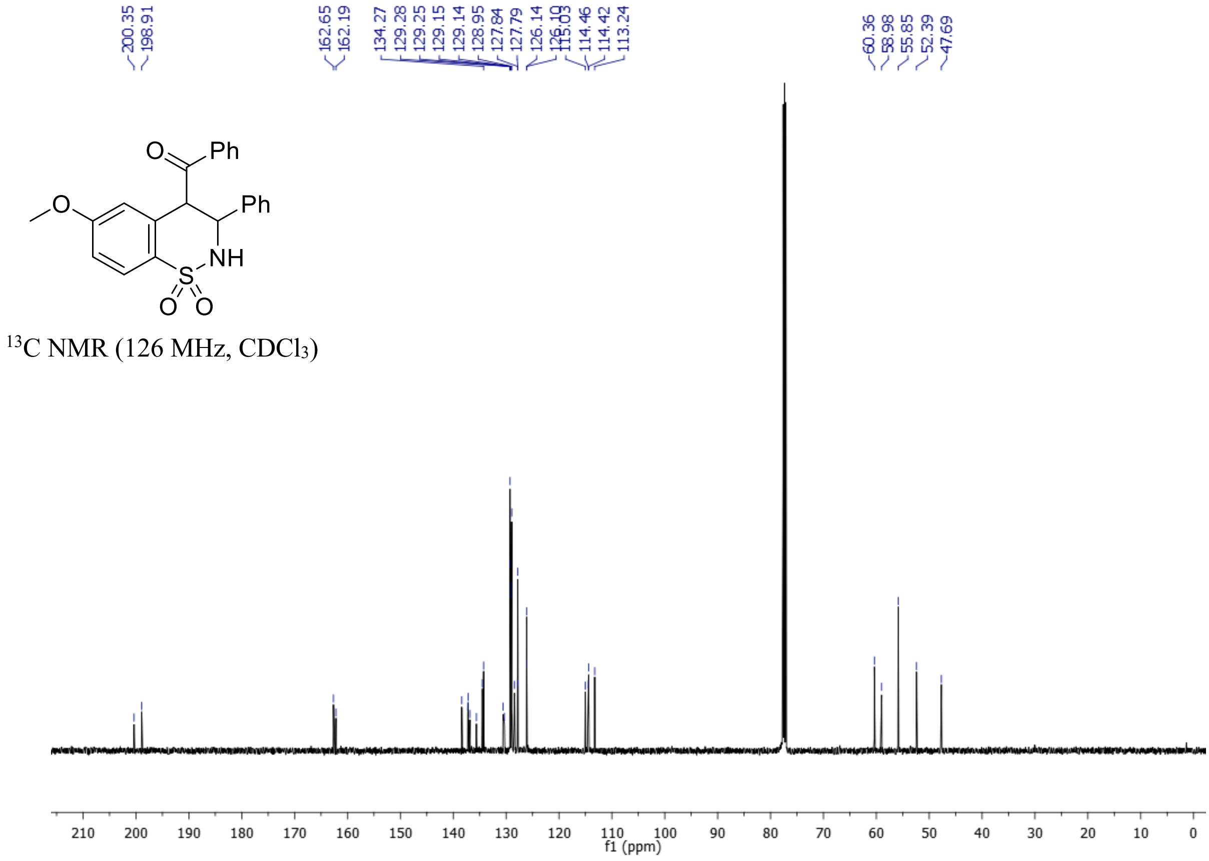

(6-Chloro-1,1-dioxido-3-phenyl-3,4-dihydro-2H-benzo[e][1,2]thiazin-4-yl)(phenyl)methanone (2e)

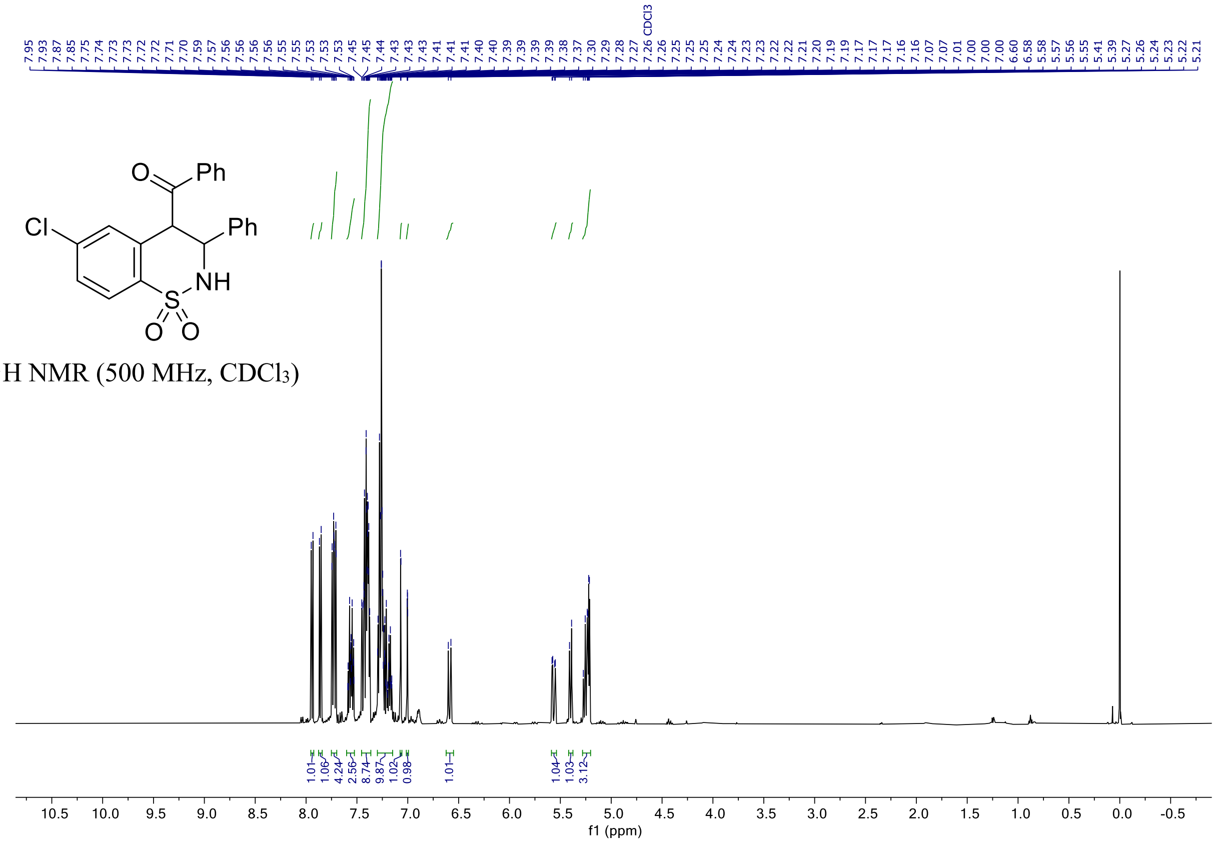

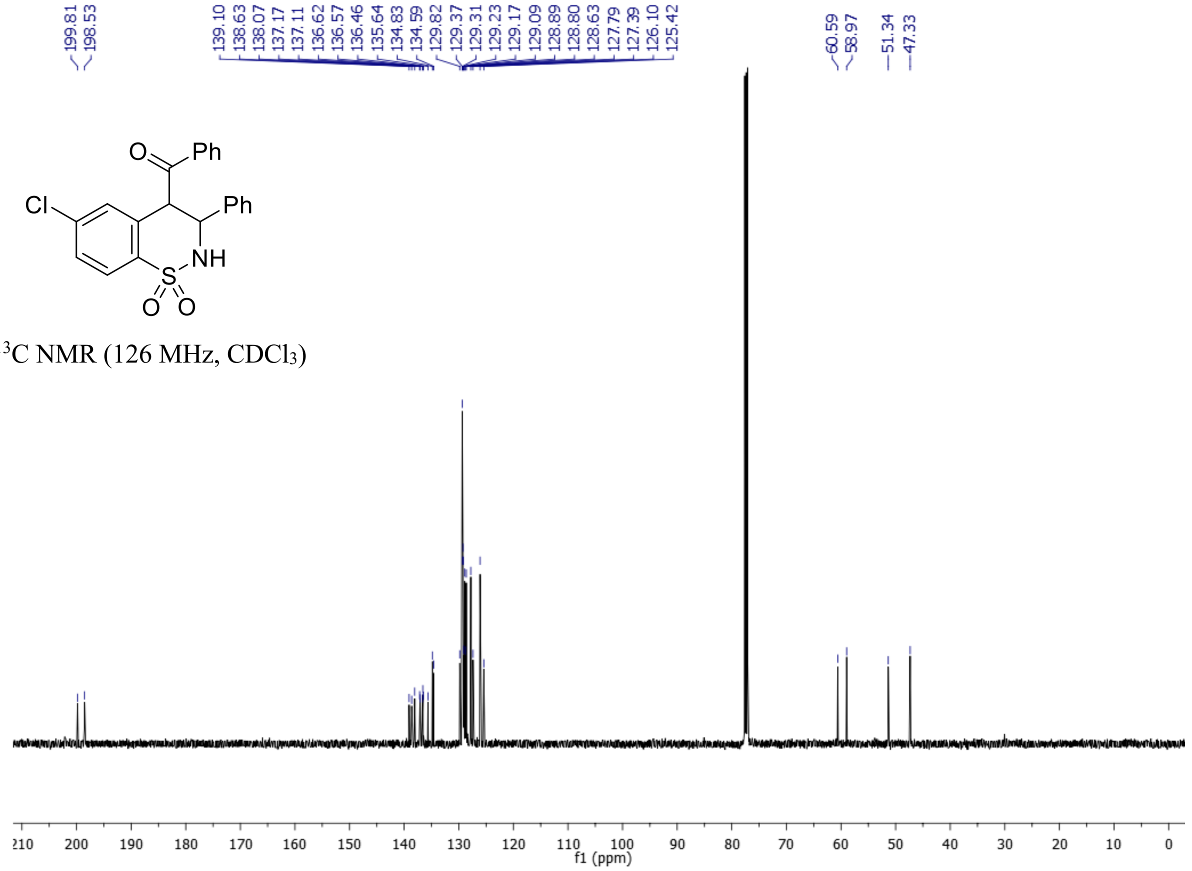

(6-Bromo-1,1-dioxido-3-phenyl-3,4-dihydro-2*H*-benzo[*e*][1,2]thiazin-4-yl)(phenyl)methanone (2f)

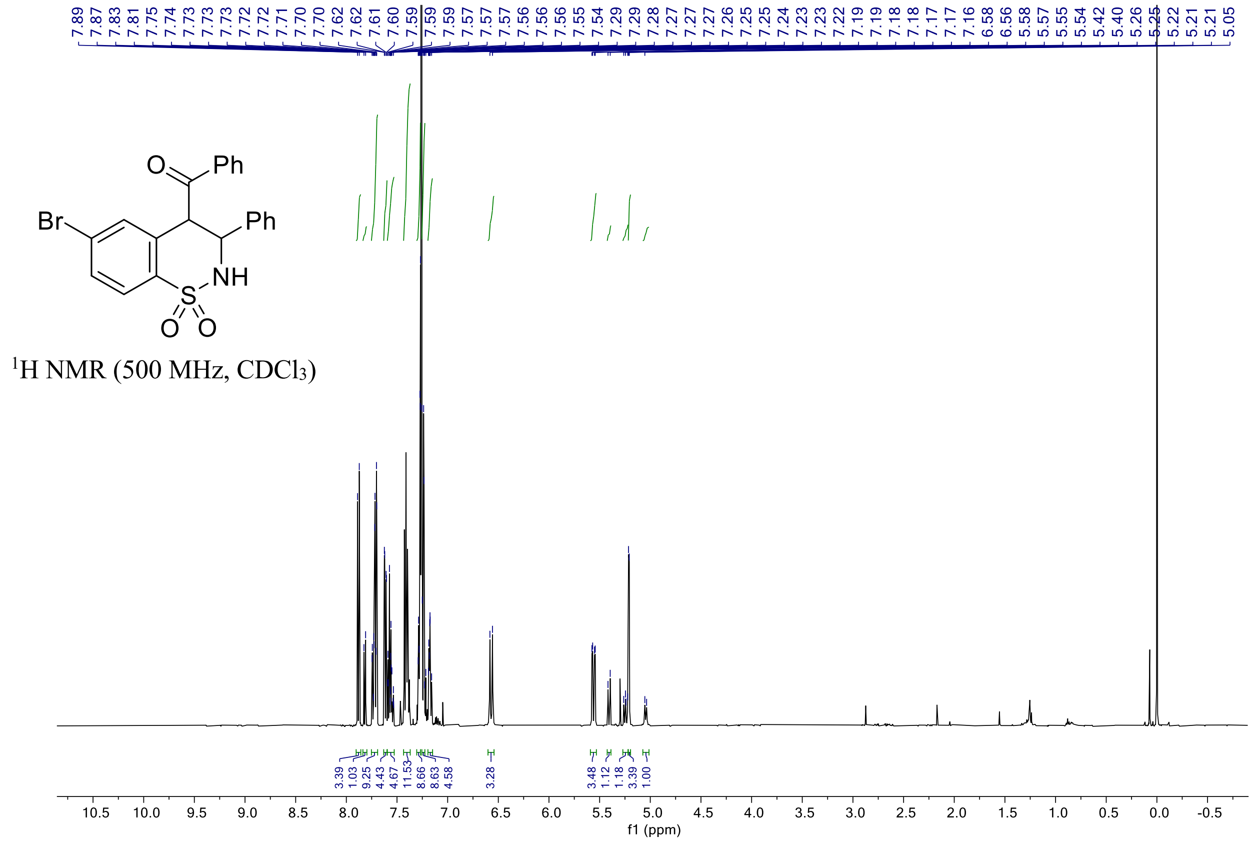

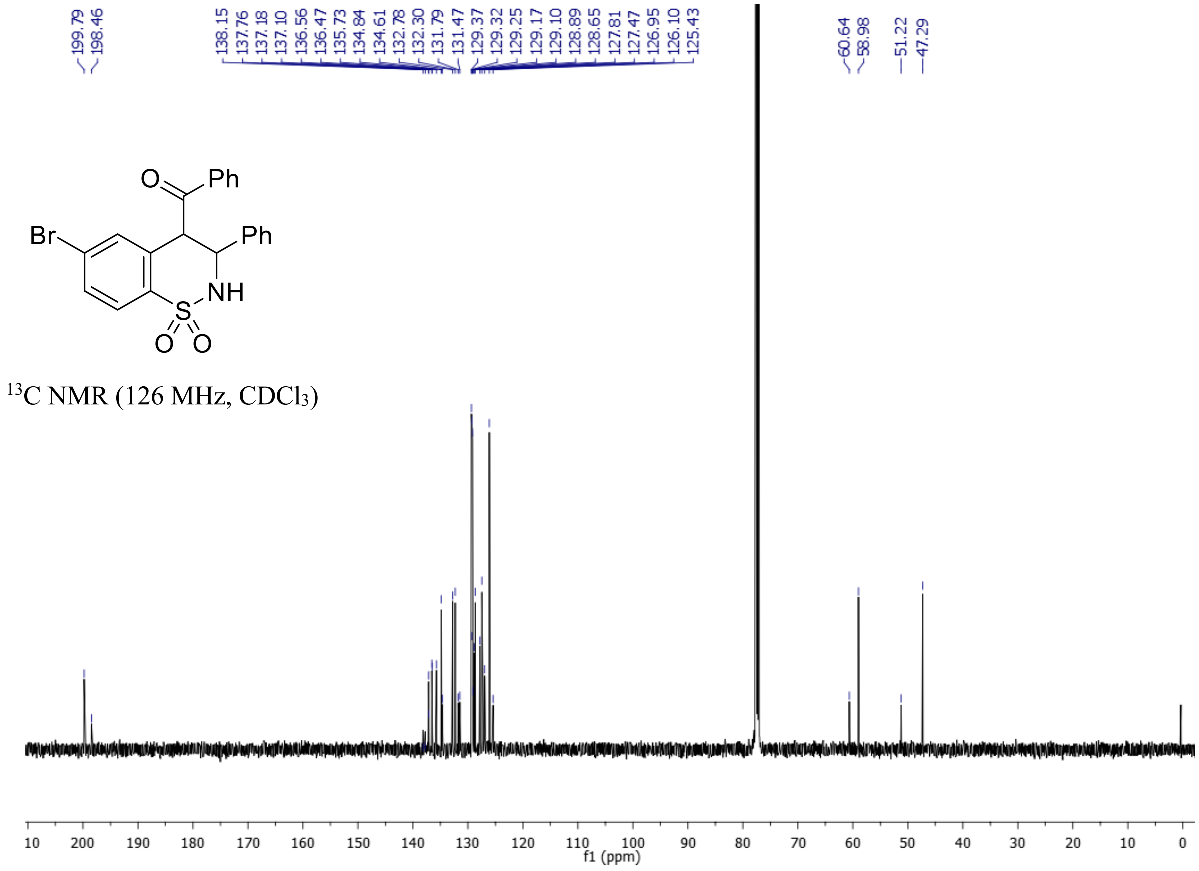

(6-Nitro-1,1-dioxido-3-phenyl-3,4-dihydro-2*H*-benzo[*e*][1,2]thiazin-4-yl)(phenyl)methanone (2g)

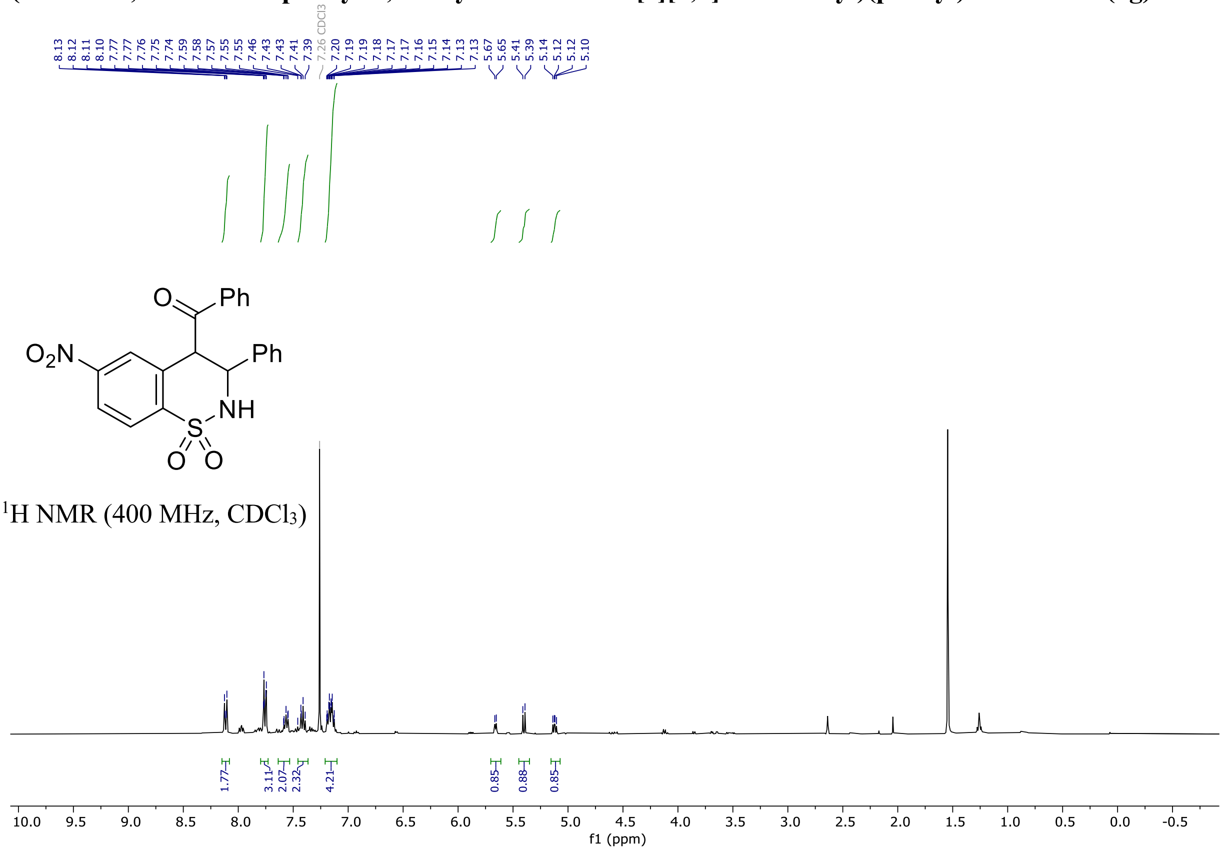

^{1}H NMR (400 MHz, $CDCl_3$)

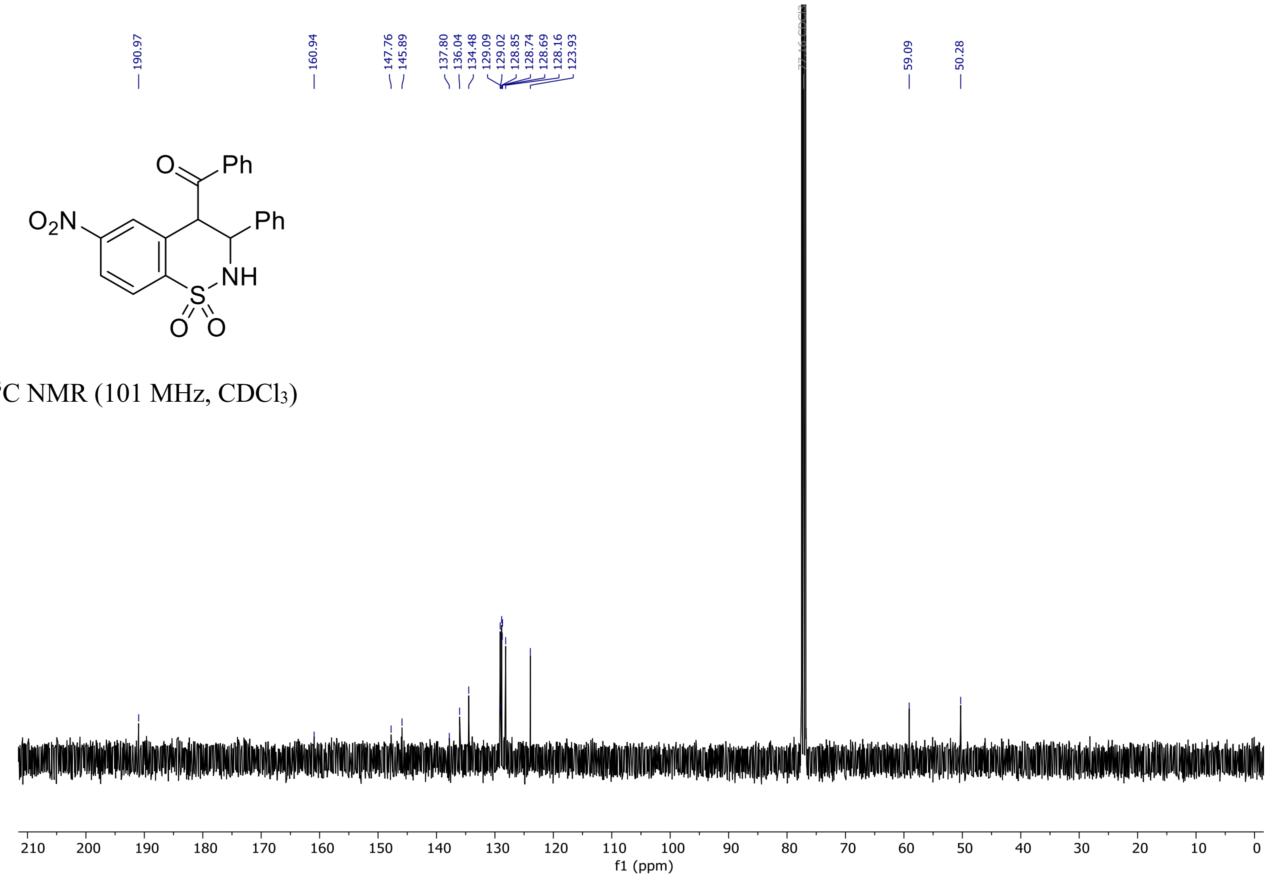

^{13}C NMR (101 MHz, $CDCl_3$)

(1,1-Dioxido-3,6-diphenyl-3,4-dihydro-2*H*-benzo[*e*][1,2]thiazin-4-yl)(phenyl)methanone (2h)

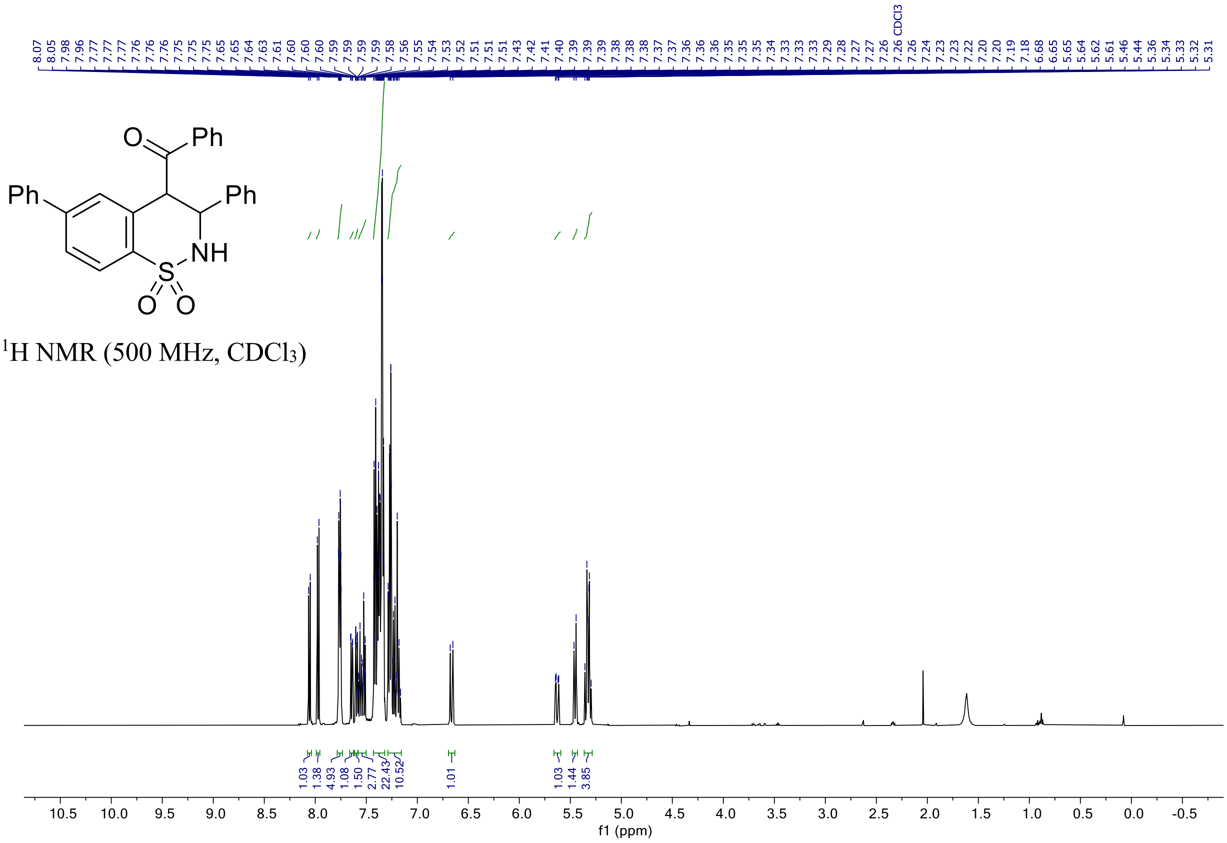

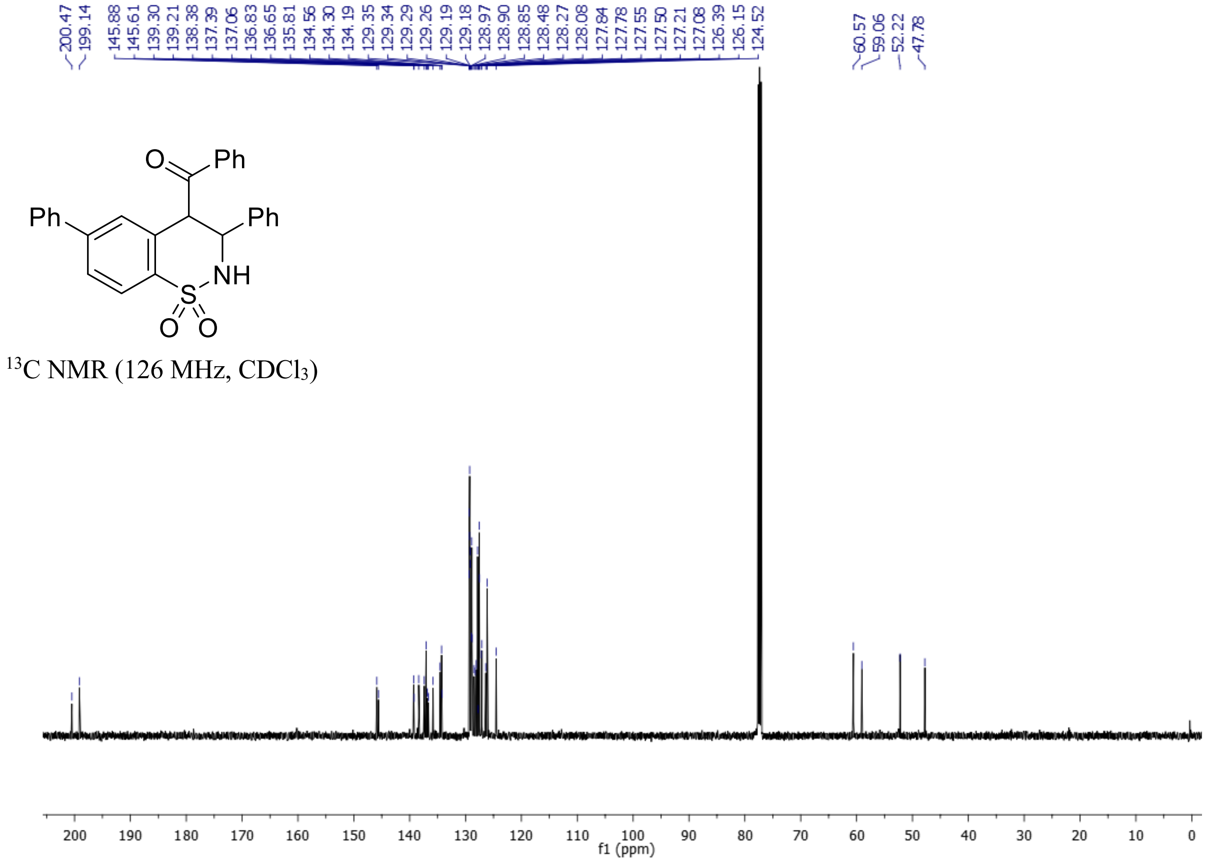

(6-Methyl-1,1-dioxido-3-phenyl-3,4-dihydro-2*H*-benzo[*e*][1,2]thiazin-4-yl)(*p*-tolyl)methanone (2i)

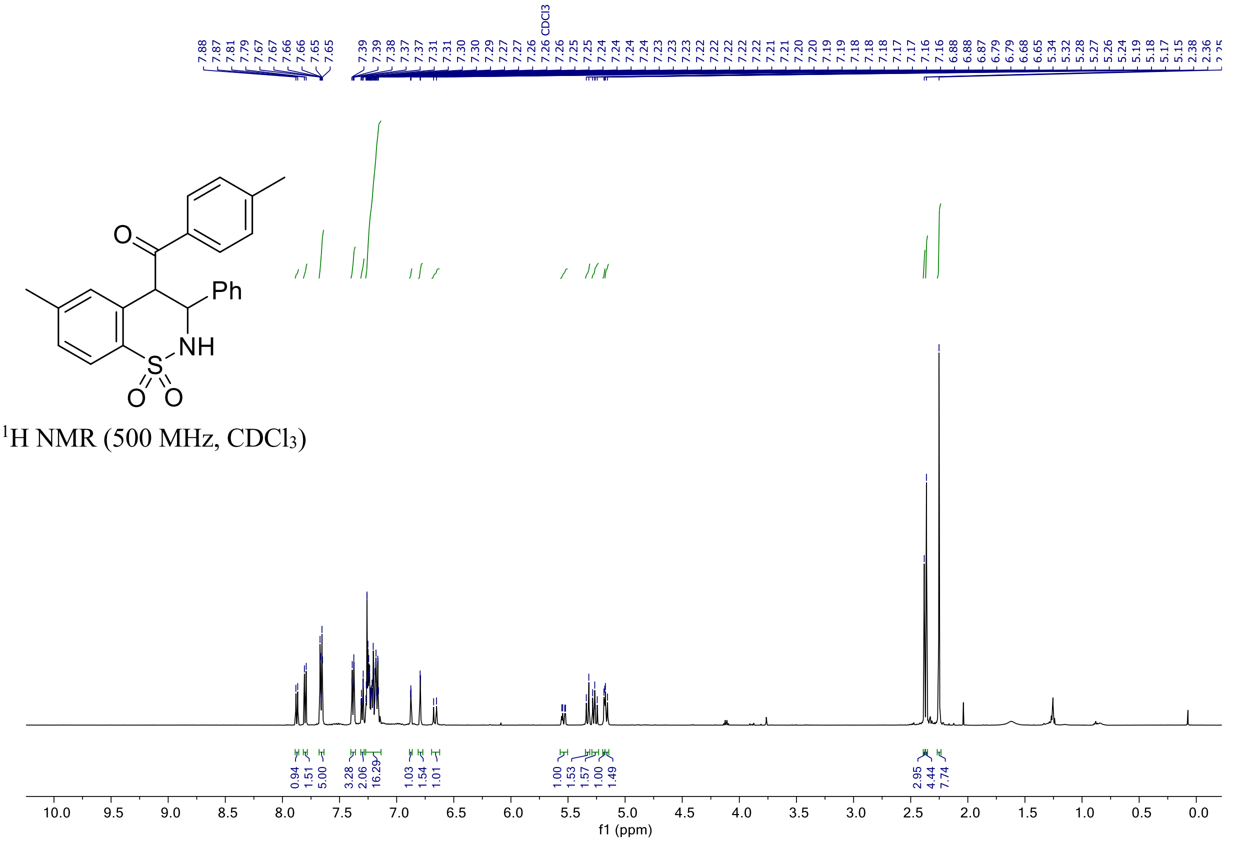

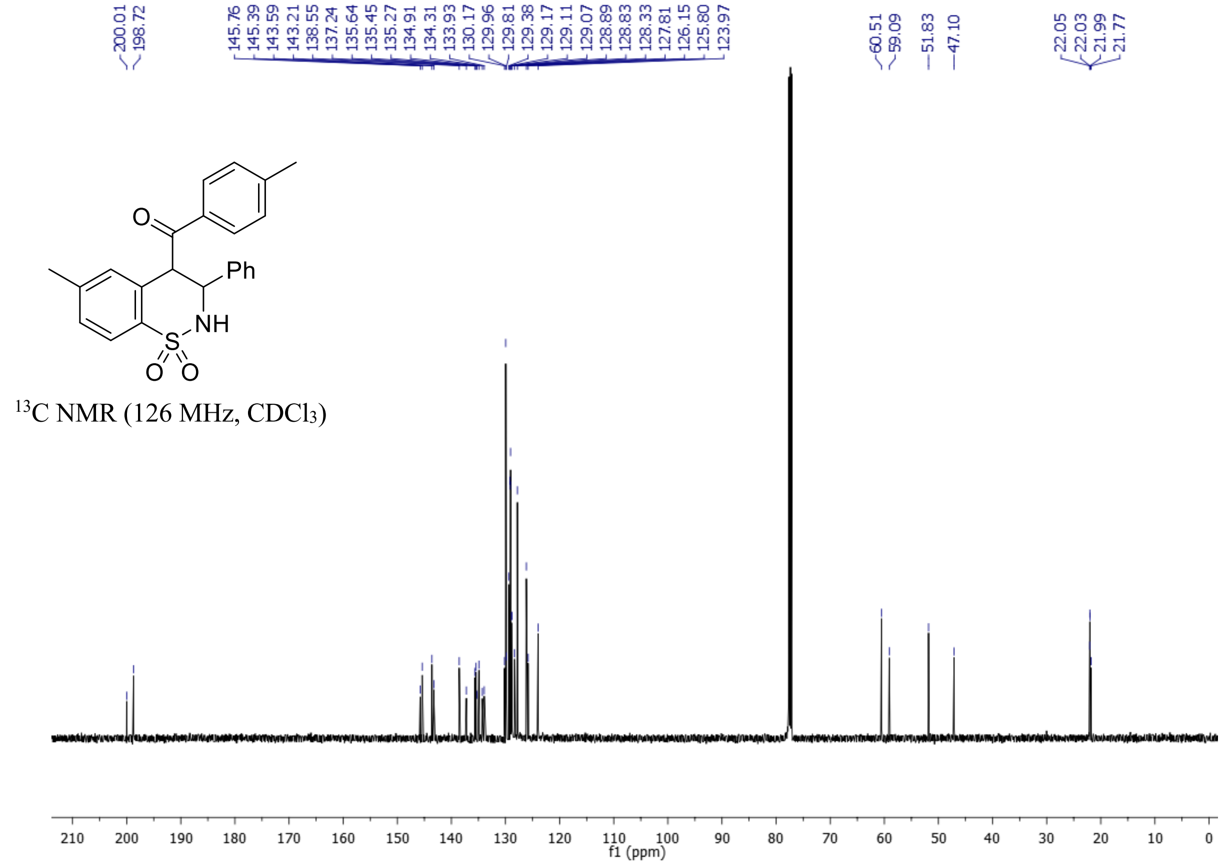

(4-Methoxyphenyl)(6-methyl-1,1-dioxido-3-phenyl-3,4-dihydro-2*H*-benzo[*e*][1,2]thiazin-4-yl)methanone (2j)

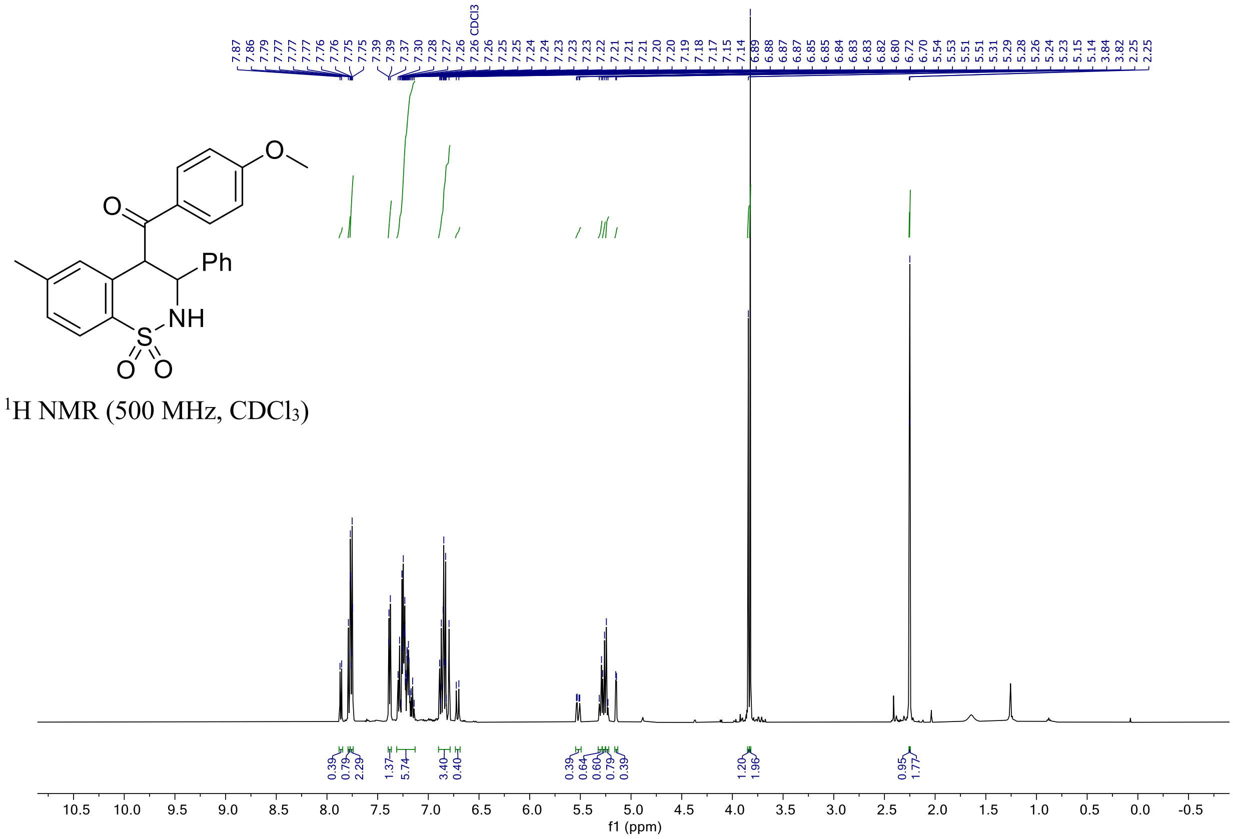

^{1}H NMR (500 MHz, $CDCl_3$)

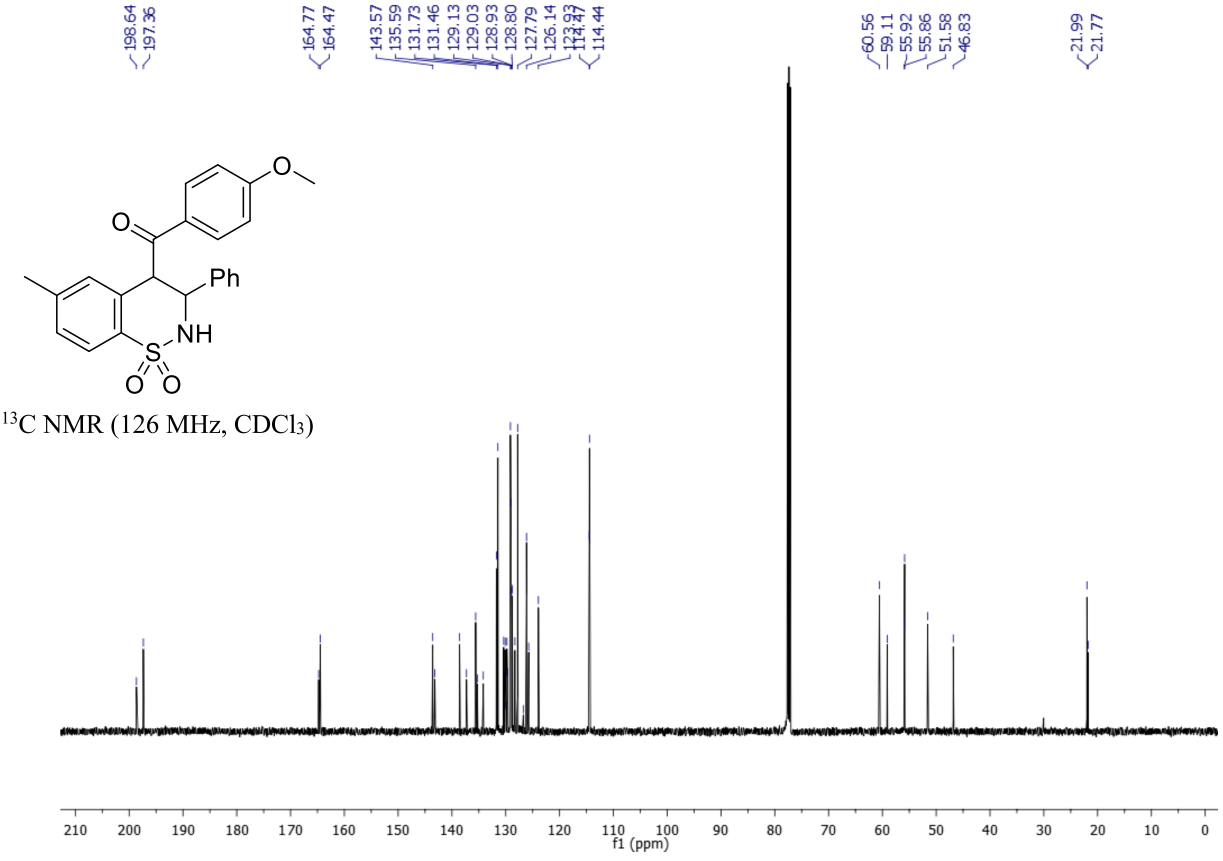

^{13}C NMR (126 MHz, $CDCl_3$)

(3-Methoxyphenyl)(6-methyl-1,1-dioxido-3-phenyl-3,4-dihydro-2*H*-benzo[*e*][1,2]thiazin-4-yl)methanone (2k)

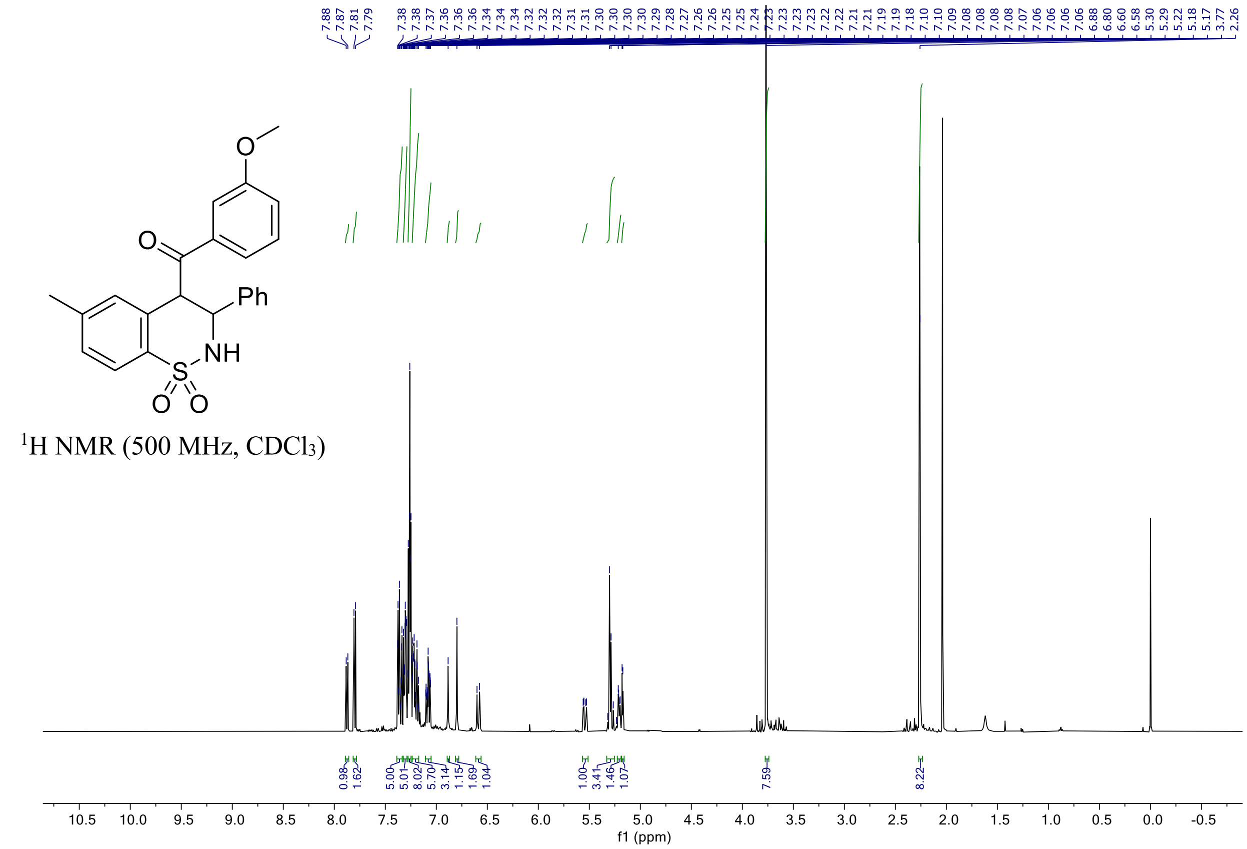

^{1}H NMR (500 MHz, $CDCl_3$)

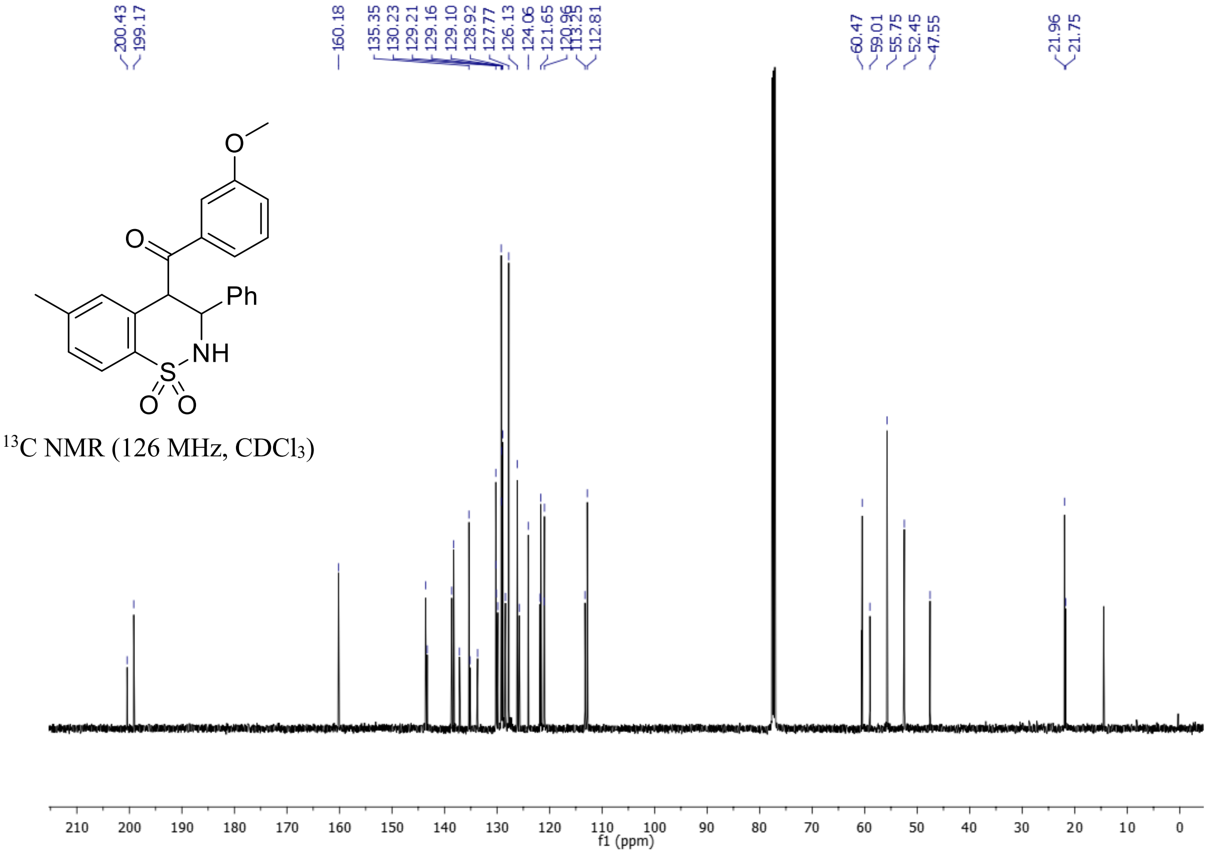

^{13}C NMR (126 MHz, $CDCl_3$)

(2-Methoxyphenyl)(6-methyl-1,1-dioxido-3-phenyl-3,4-dihydro-2*H*-benzo[*e*][1,2]thiazin-4-yl)methanone (2l)

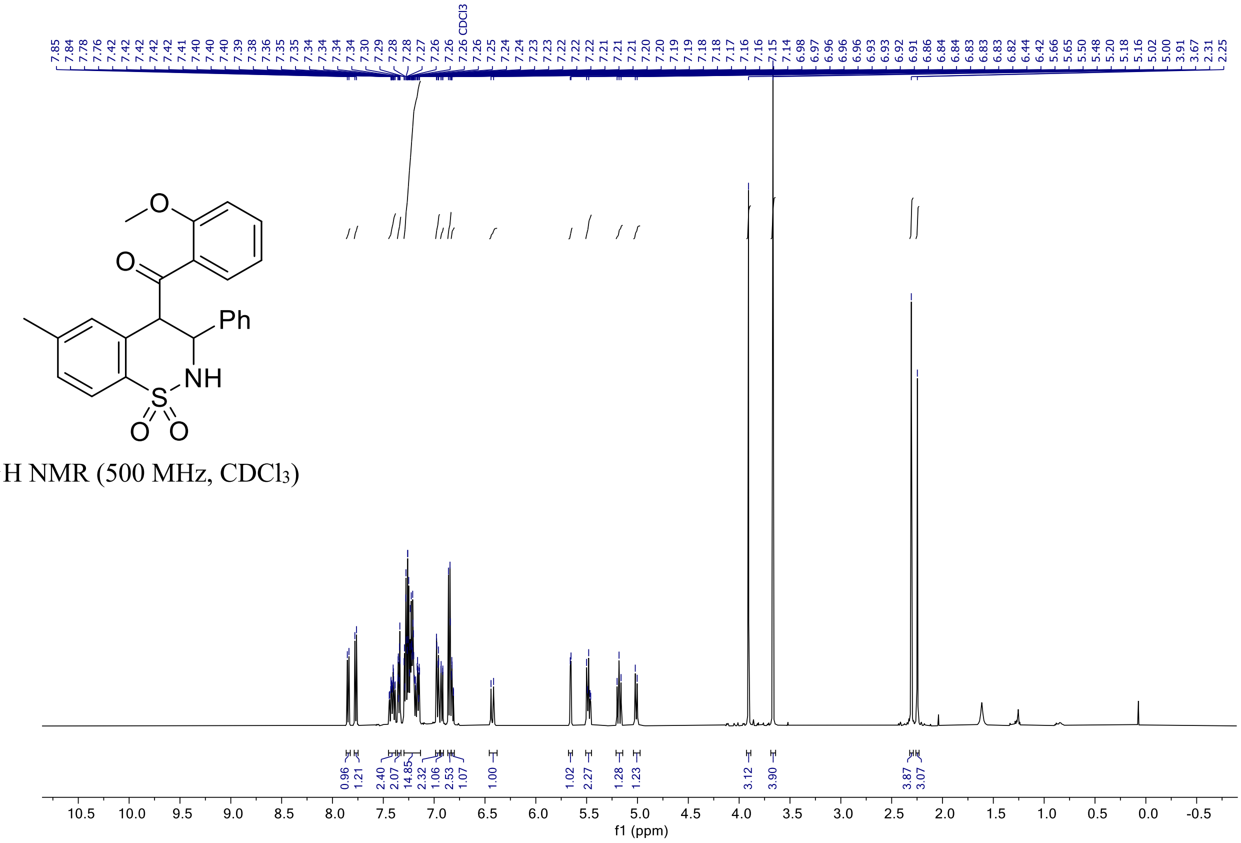

^{1}H NMR (500 MHz, $CDCl_3$)

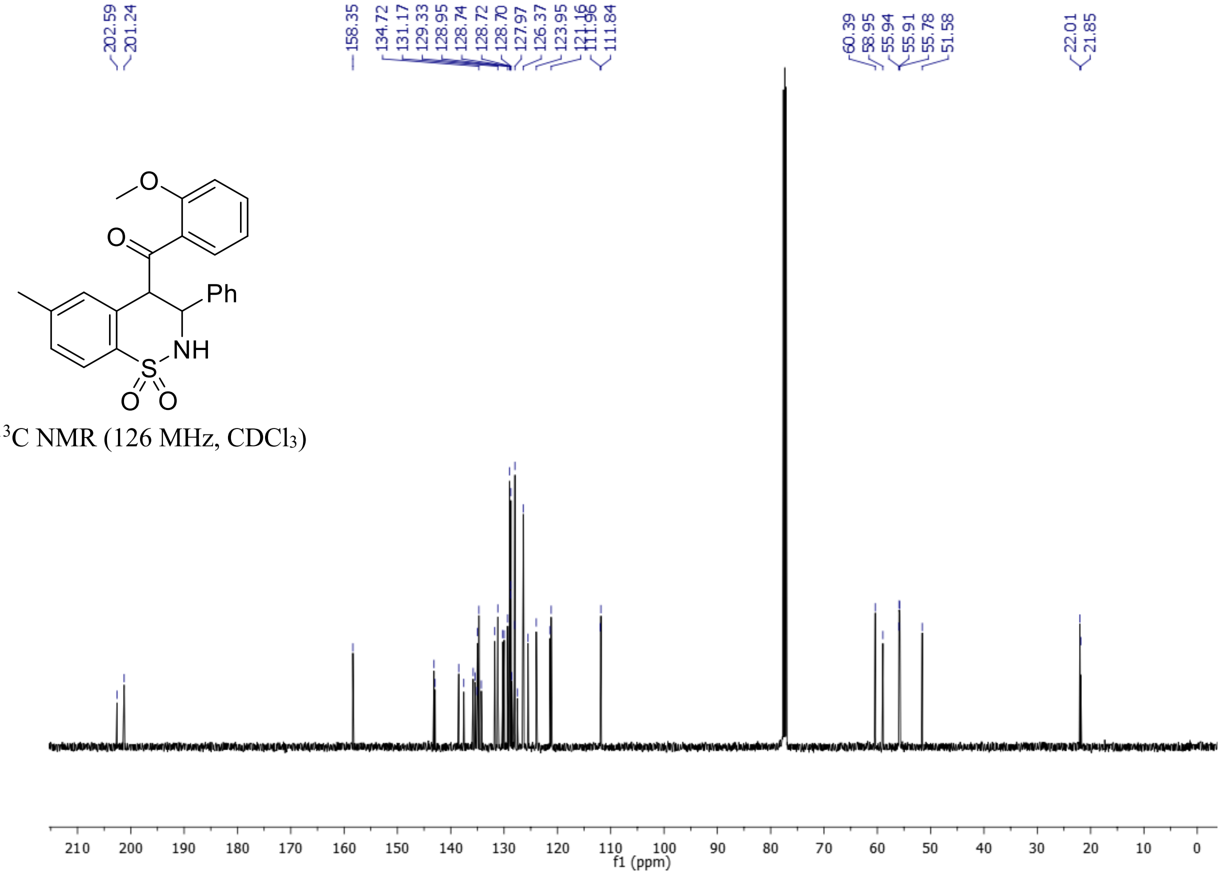

^{13}C NMR (126 MHz, $CDCl_3$)

(6-Methyl-1,1-dioxido-3-phenyl-3,4-dihydro-2*H*-benzo[*e*][1,2]thiazin-4-yl)(naphthalen-2-yl)methanone (2m)

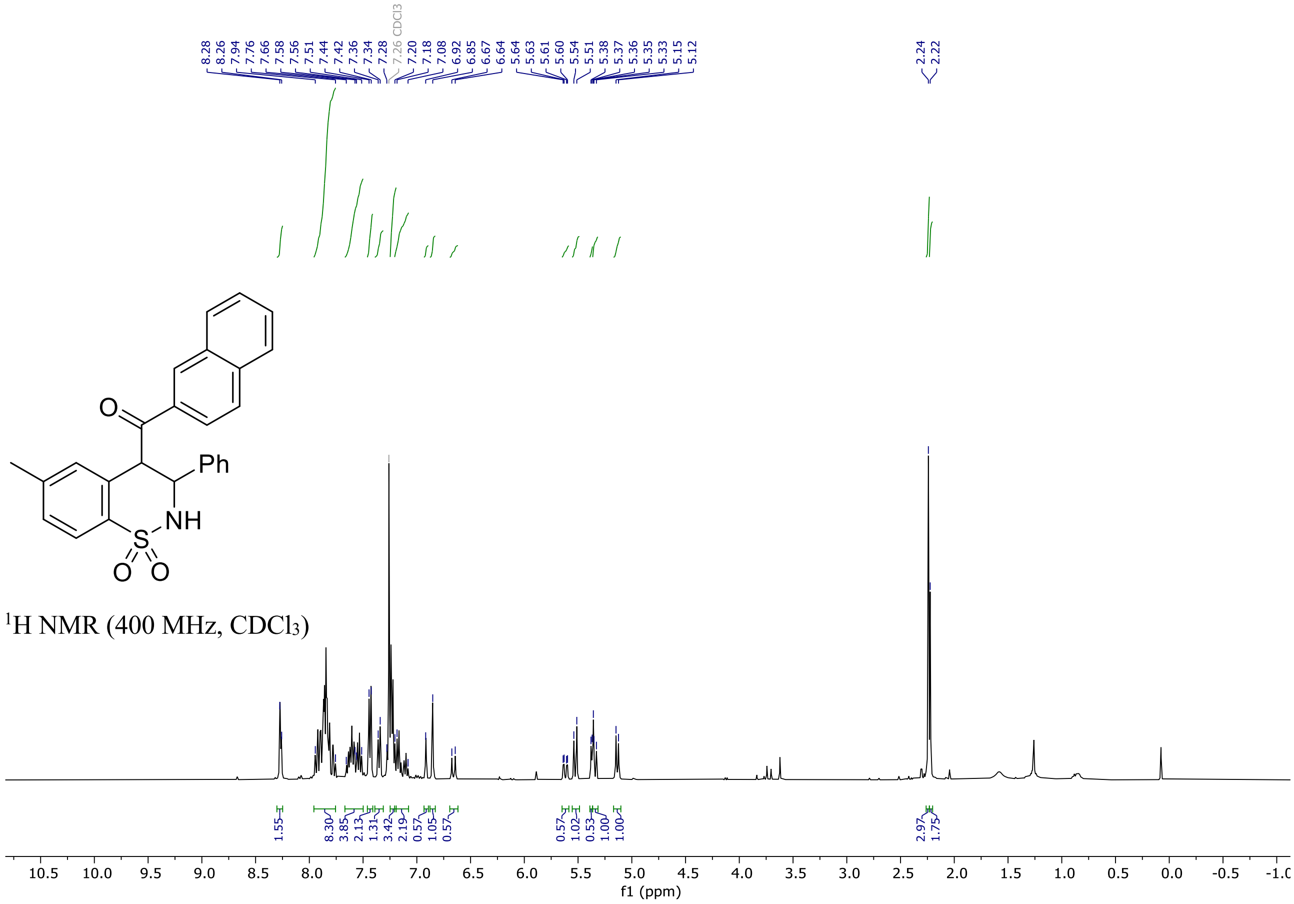

^{1}H NMR (400 MHz, $CDCl_3$)

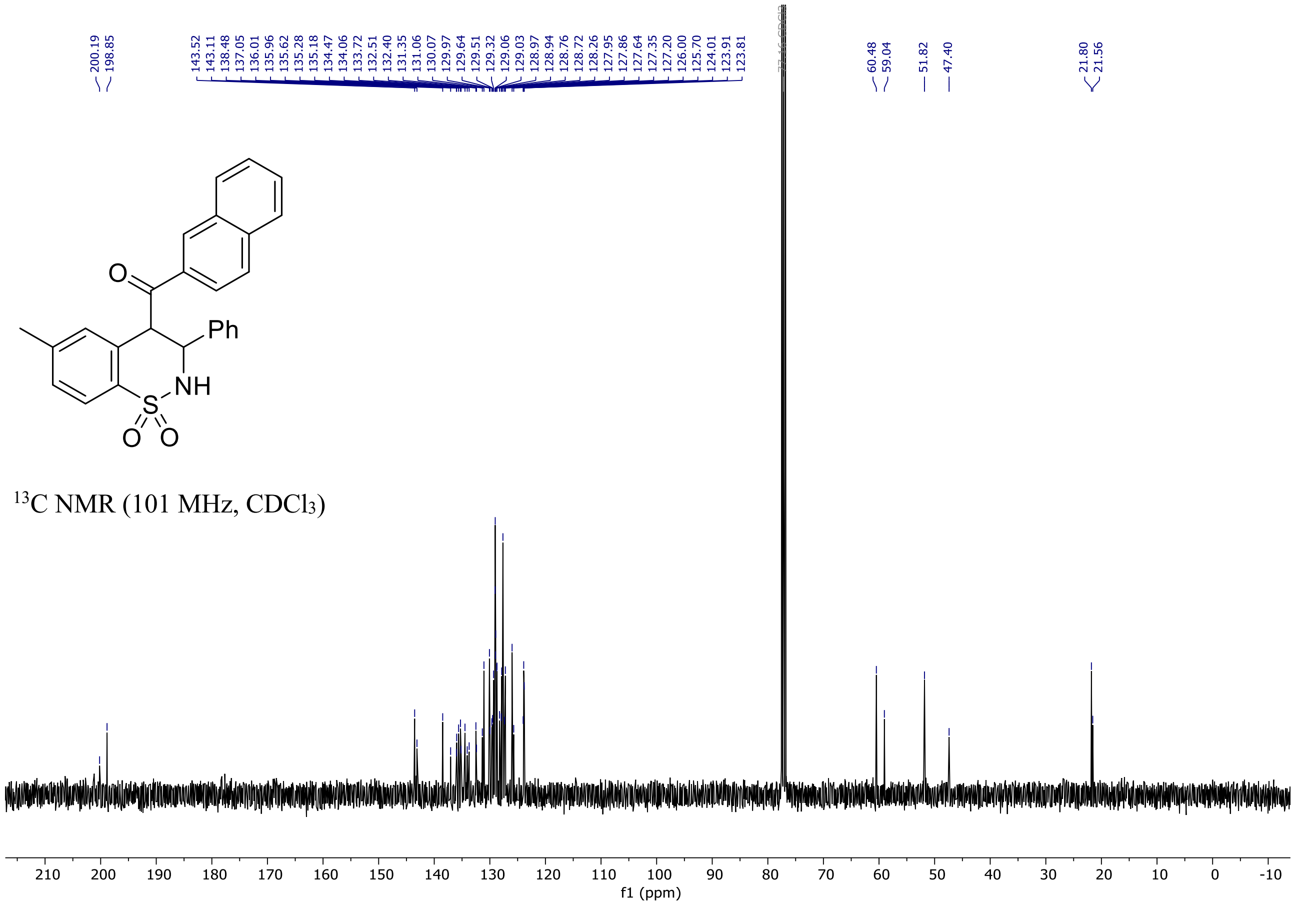

^{13}C NMR (101 MHz, $CDCl_3$)

(3-(4-Chlorophenyl)-6-methyl-1,1-dioxido-3,4-dihydro-2*H*-benzo[*e*][1,2]thiazin-4-yl)methanone (2n)

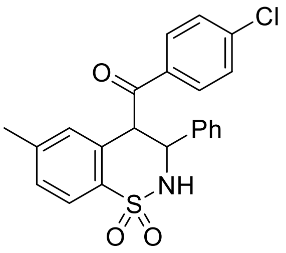

^{1}H NMR (500 MHz, $CDCl_3$)

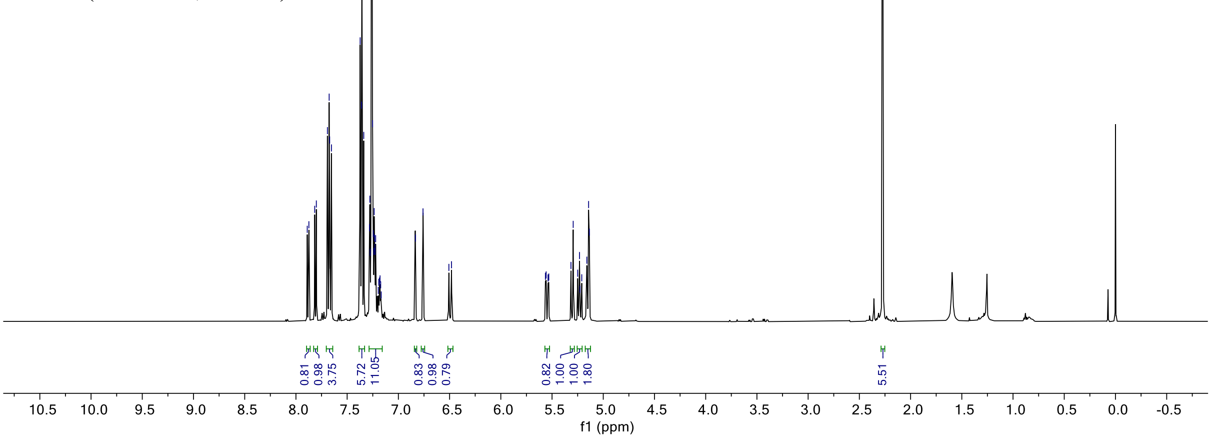

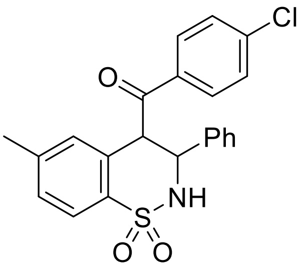

^{13}C NMR (126 MHz, $CDCl_3$)

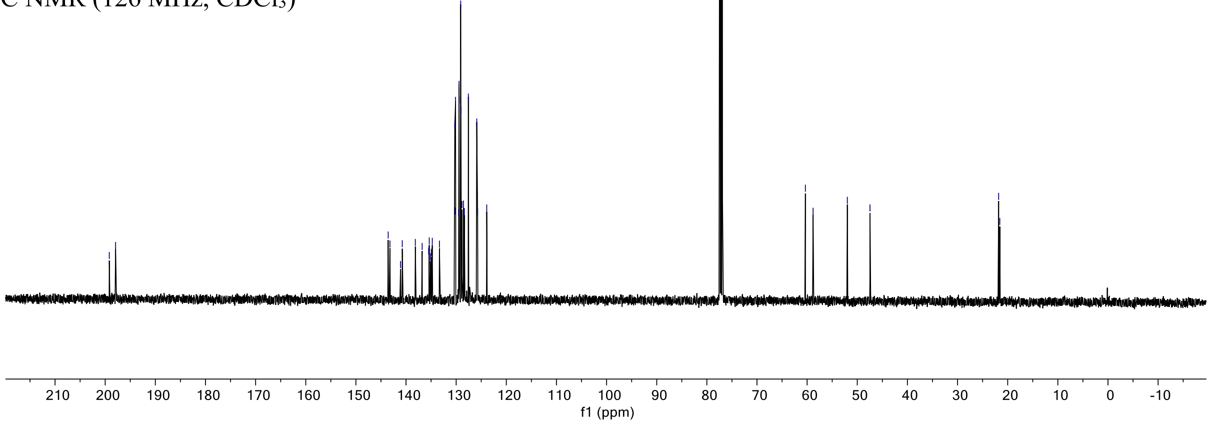

(4-Bromophenyl)(6-methyl-1,1-dioxido-3-phenyl-3,4-dihydro-2*H*-benzo[*e*][1,2]thiazin-4-yl)methanone (2o)

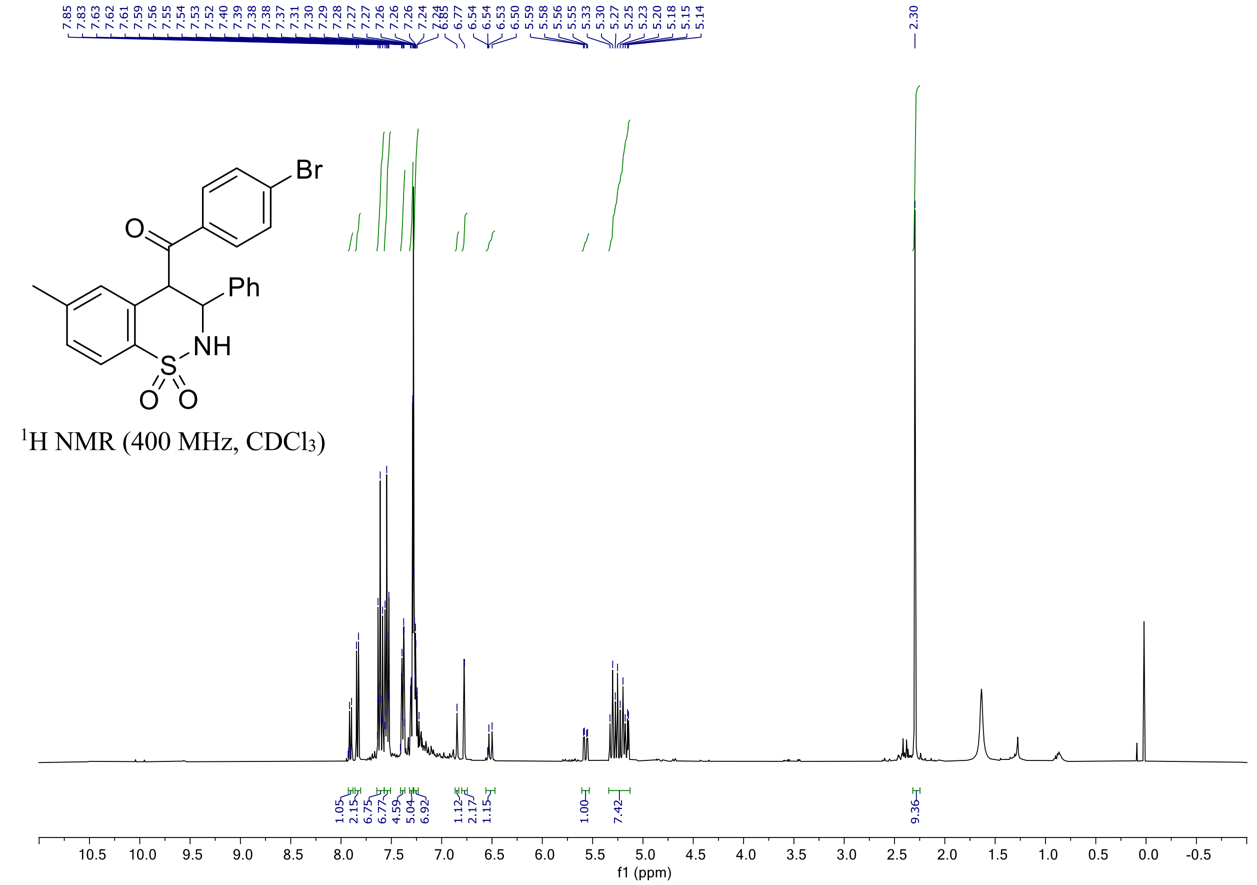

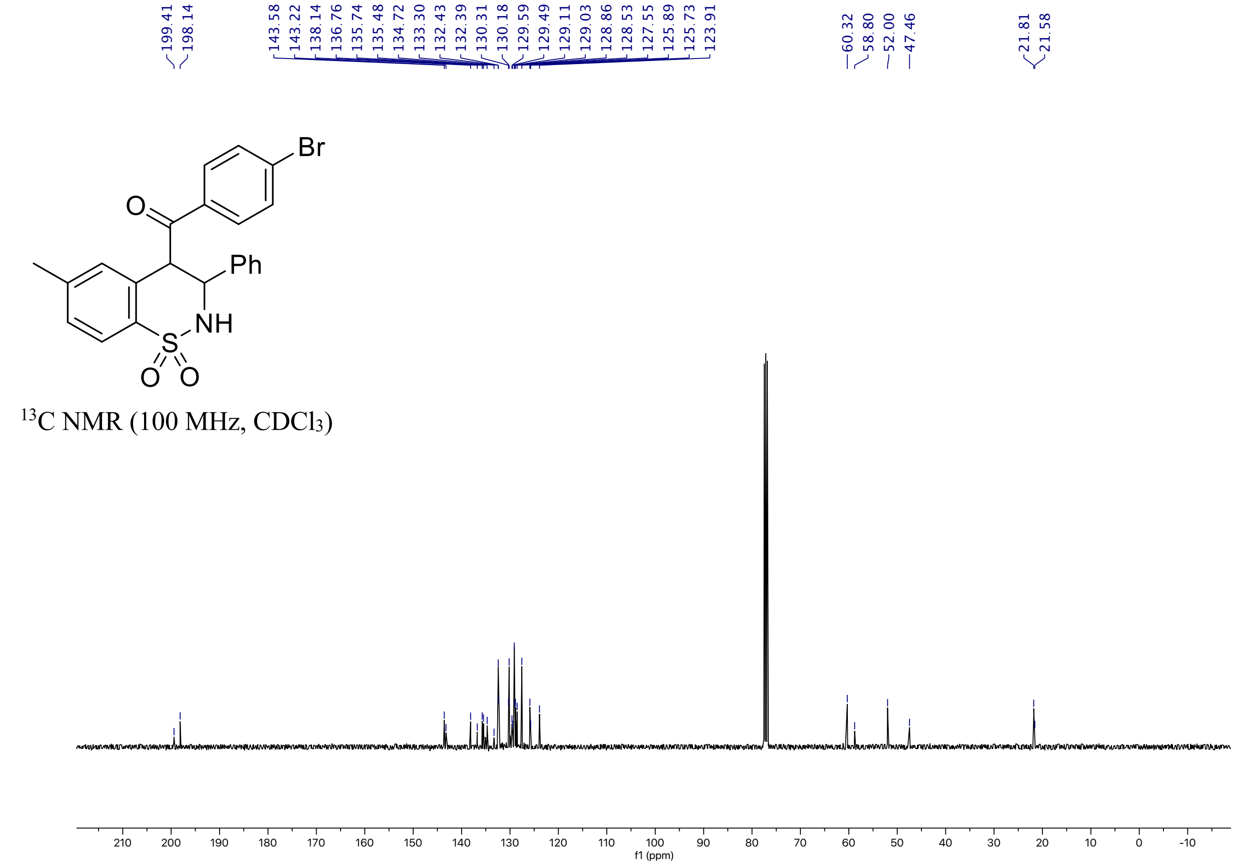

(4-Fluorophenyl)(6-methyl-1,1-dioxido-3-phenyl-3,4-dihydro-2*H*-benzo[*e*][1,2]thiazin-4-yl)methanone (2p)

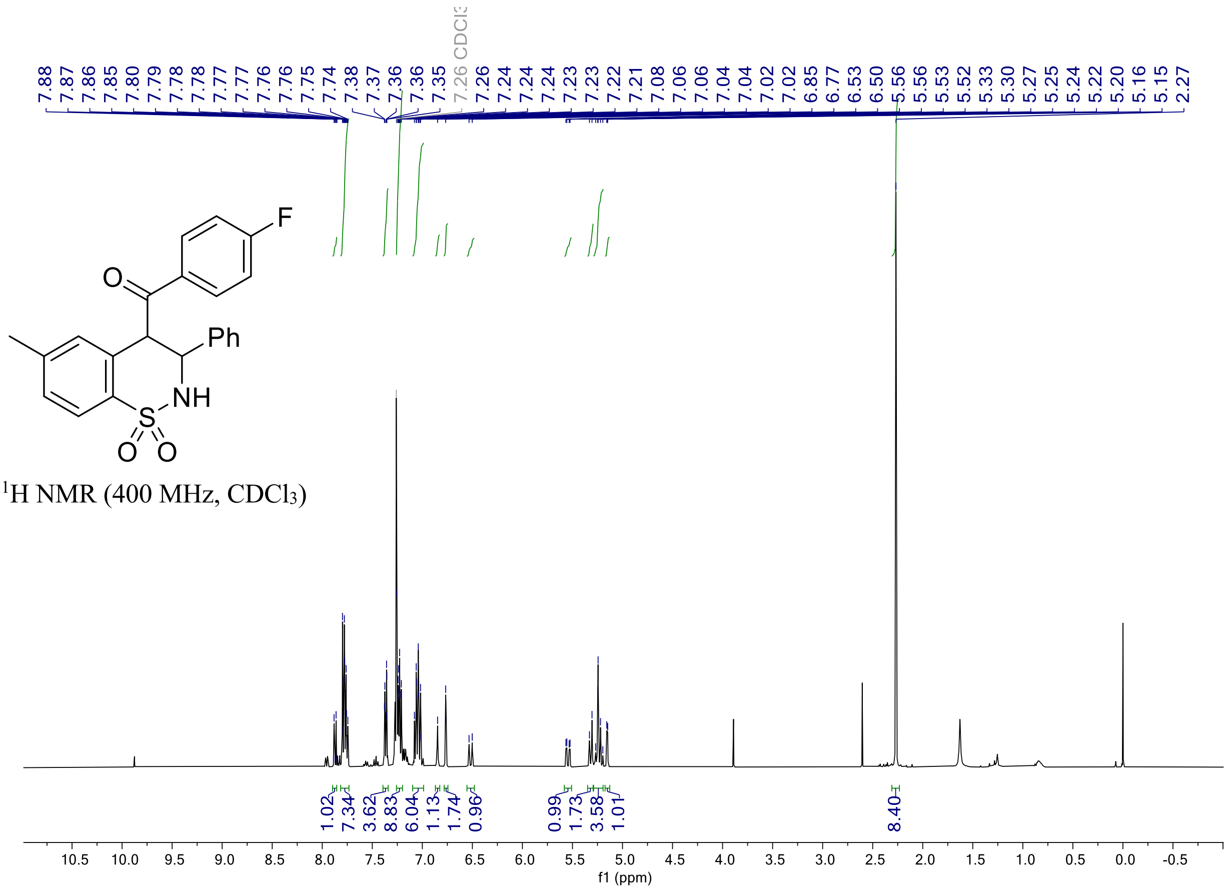

^{1}H NMR (400 MHz, $CDCl_3$)

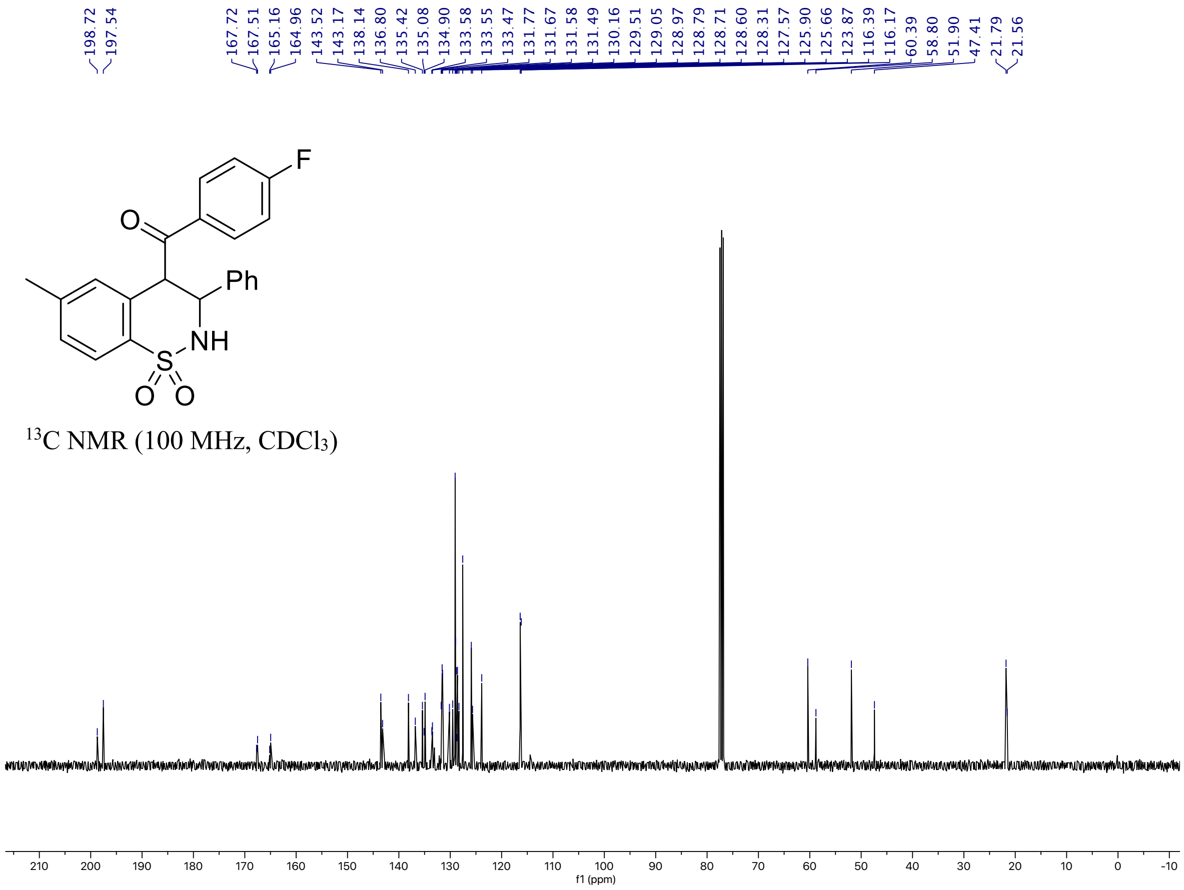

^{13}C NMR (100 MHz, $CDCl_3$)

(3-Fluorophenyl)(6-methyl-1,1-dioxido-3-phenyl-3,4-dihydro-2*H*-benzo[*e*][1,2]thiazin-4-yl)methanone (2q)

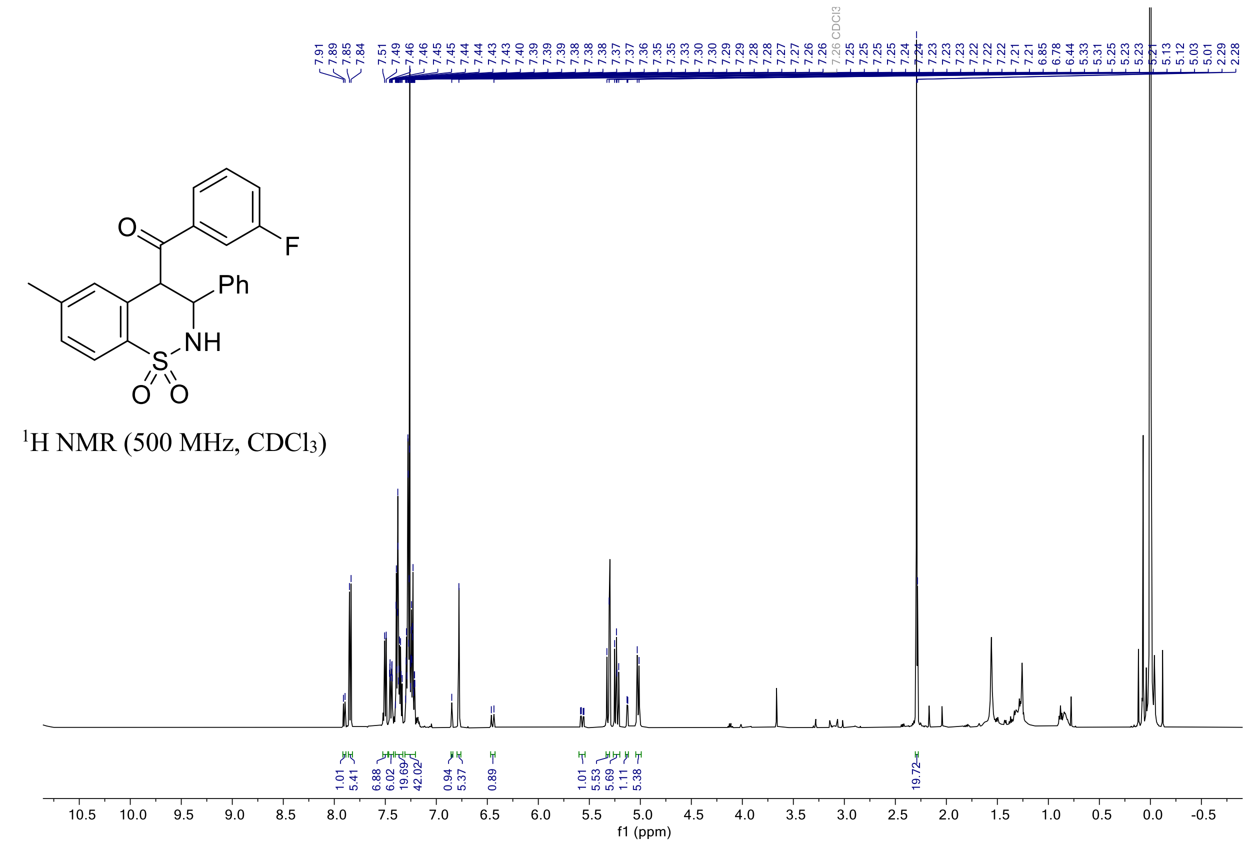

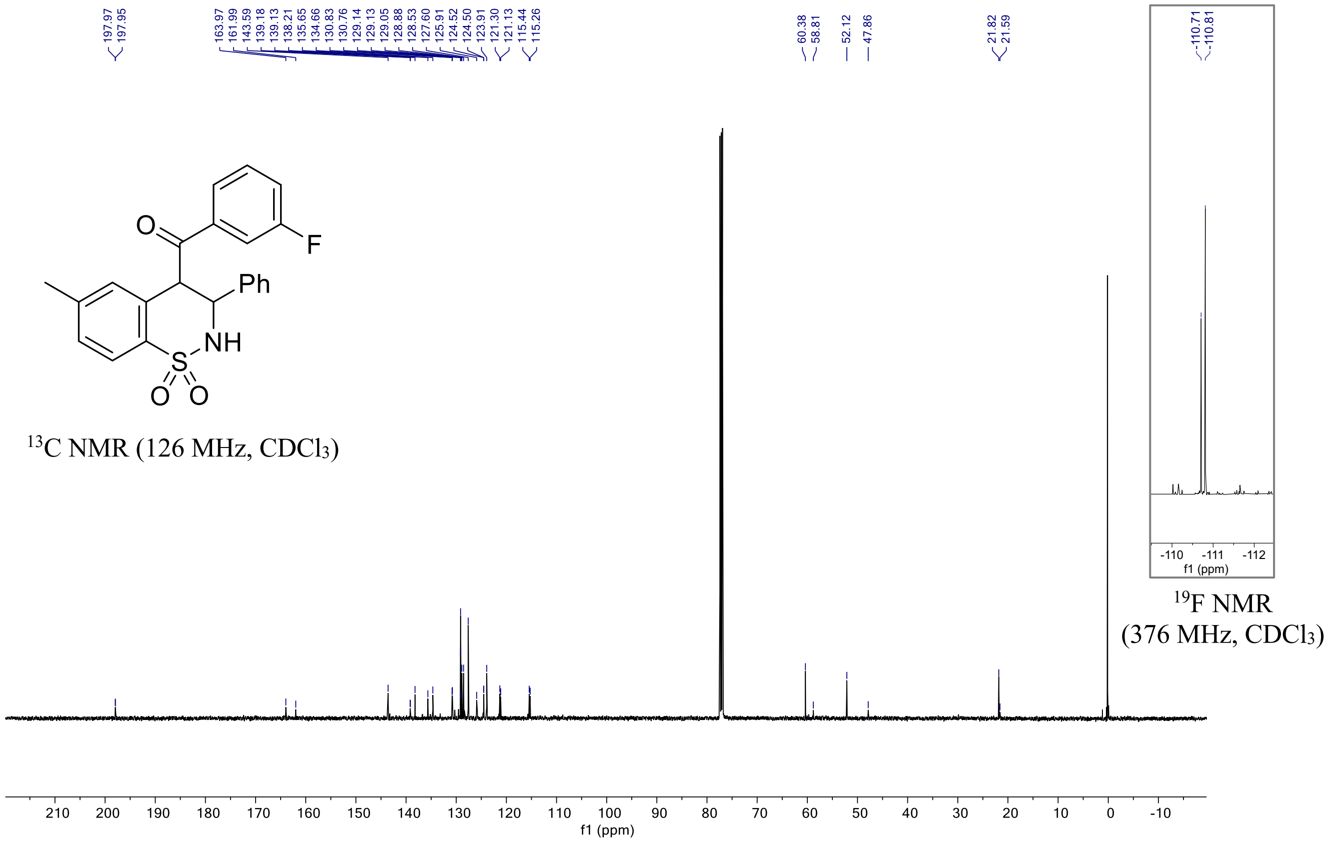

(2-Fluorophenyl)(6-methyl-1,1-dioxido-3-phenyl-3,4-dihydro-2*H*-benzo[*e*][1,2]thiazin-4-yl)methanone (2r)

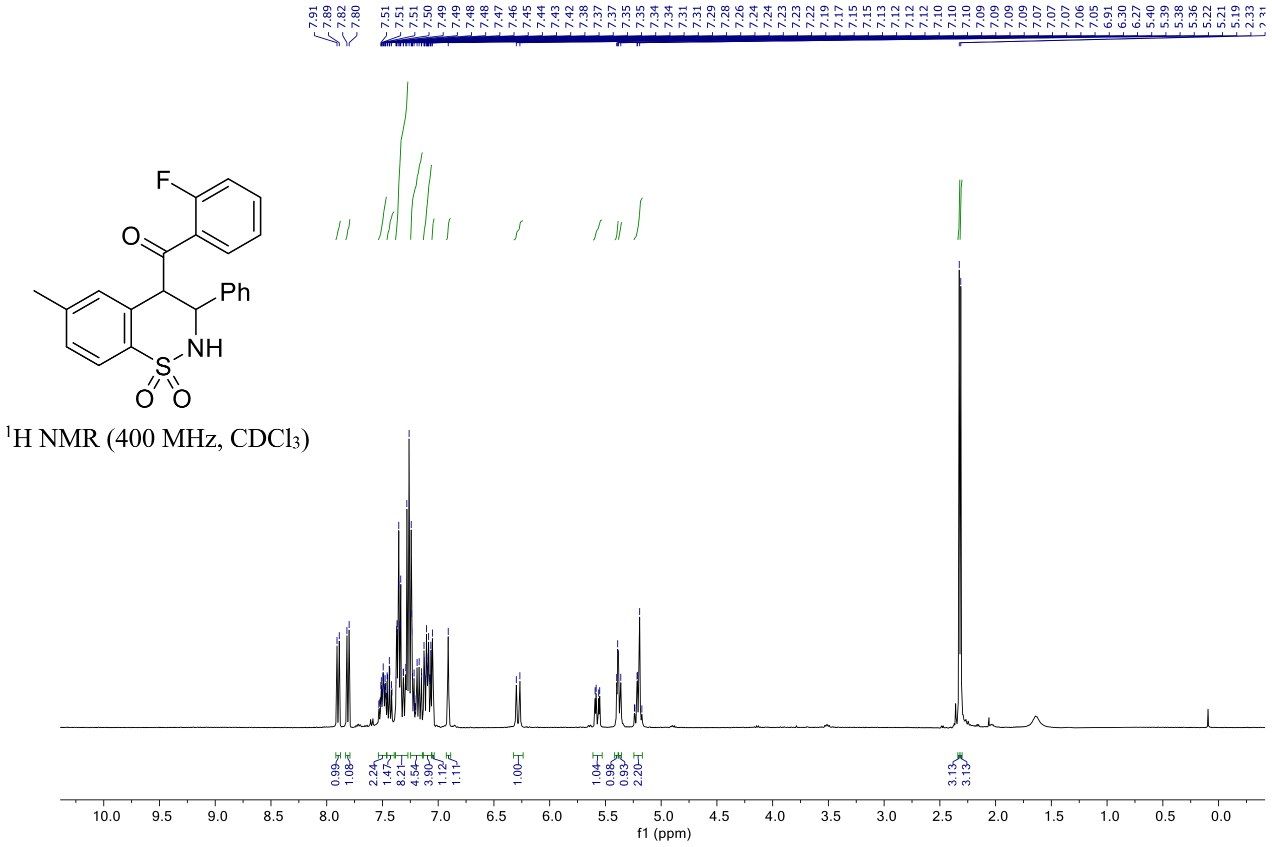

^{1}H NMR (400 MHz, CDCl$_3$)

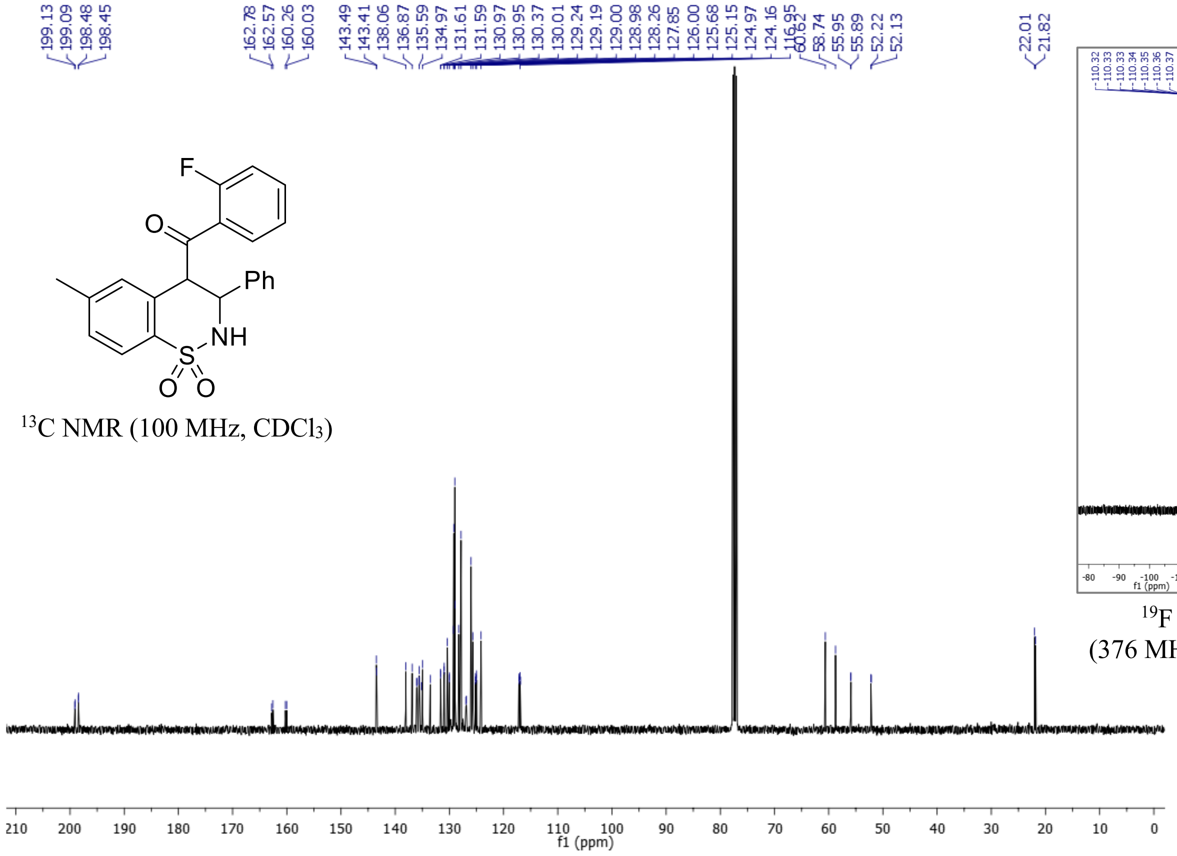

^{13}C NMR (100 MHz, CDCl$_3$)

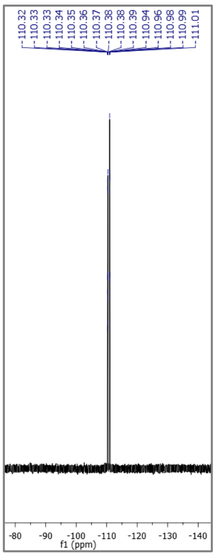

^{19}F NMR (376 MHz, CDCl$_3$)

4-Methyl-*N*-(3-oxo-1-phenyl-3-(pyridin-2-yl)propyl)benzenesulfonamide (2s')

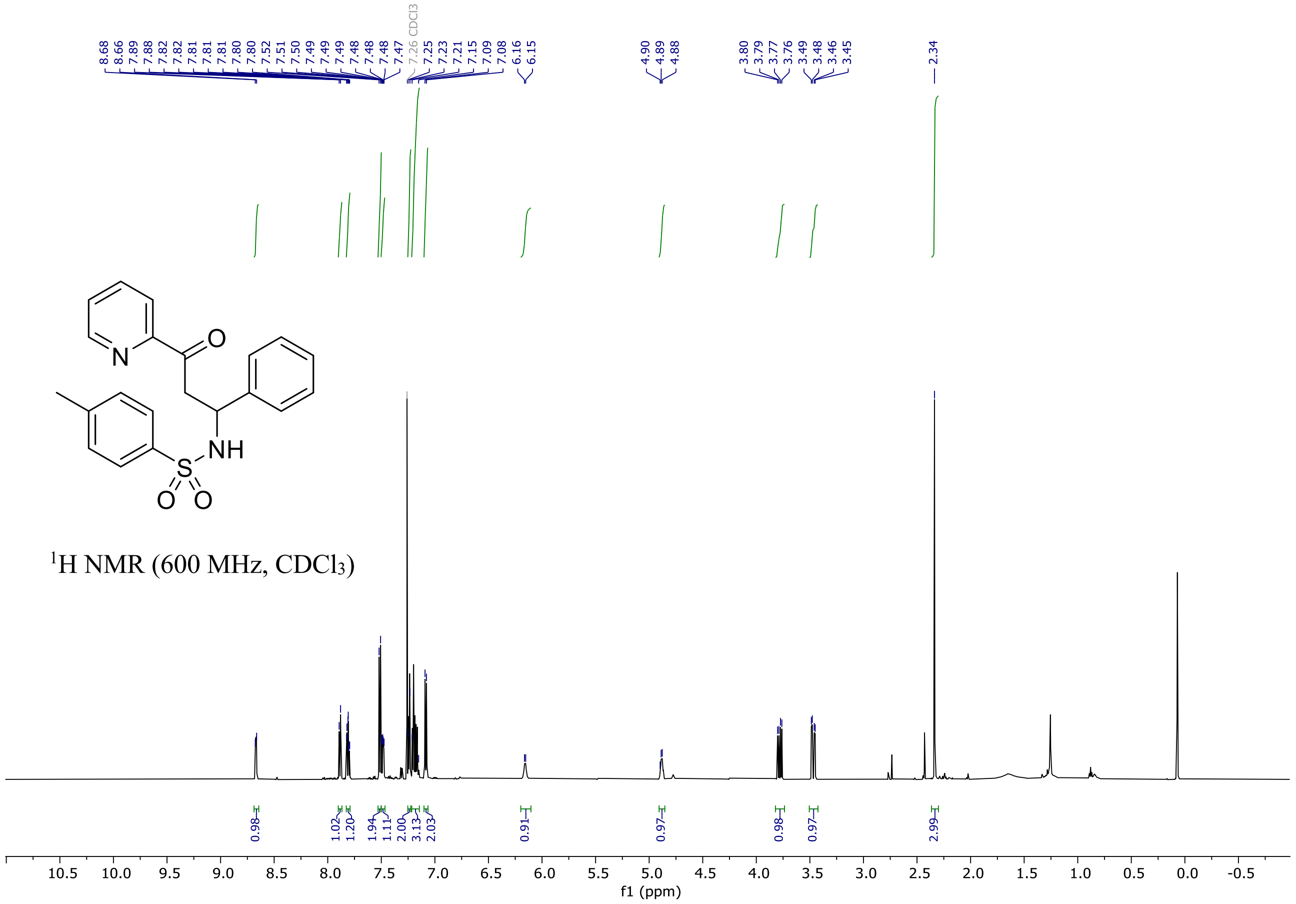

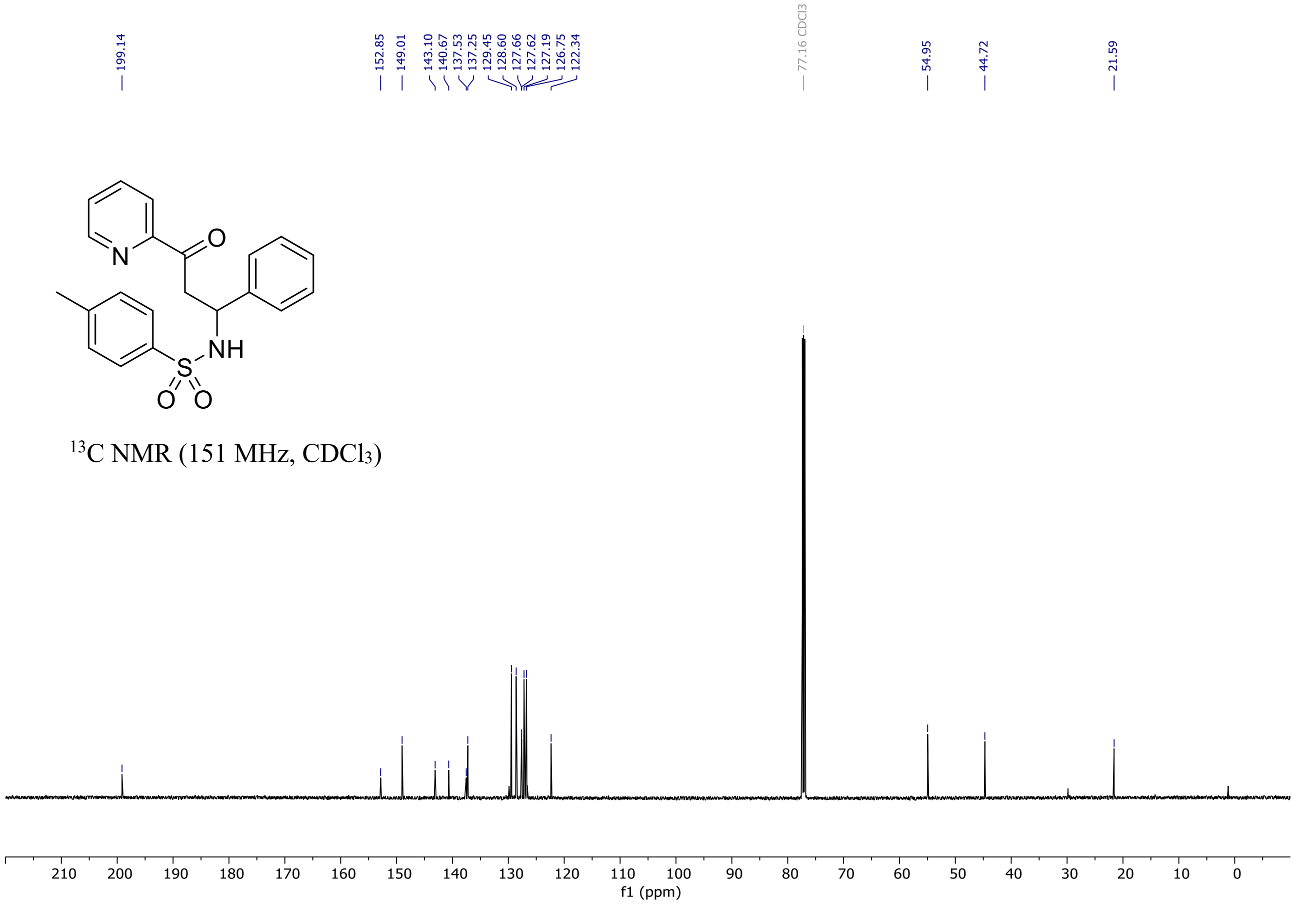

(6-Methyl-1,1-dioxido-3-phenyl-3,4-dihydro-2*H*-benzo[*e*][1,2]thiazin-4-yl)(thiophen-2-yl)methanone (2t)

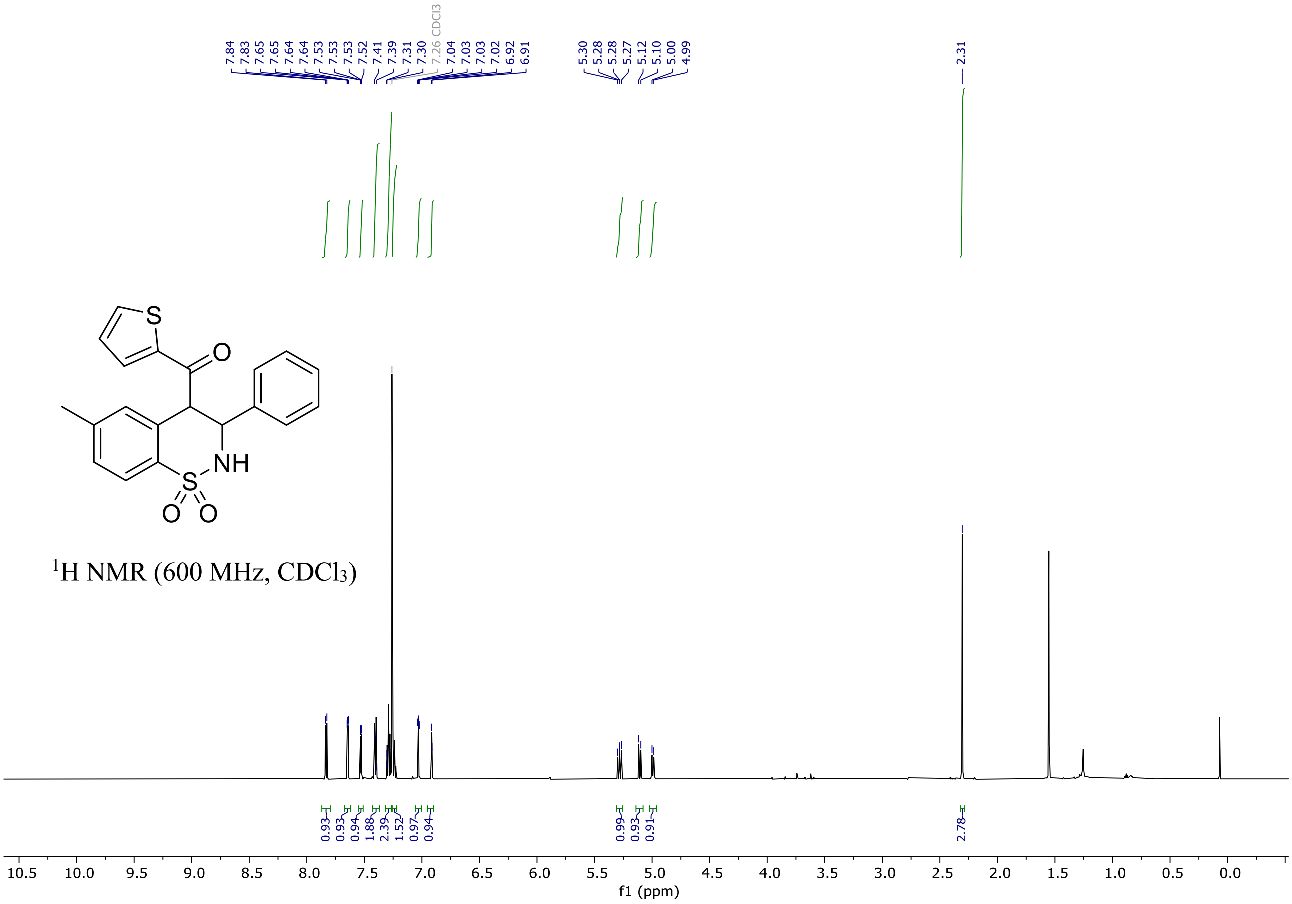

^{1}H NMR (600 MHz, $CDCl_3$)

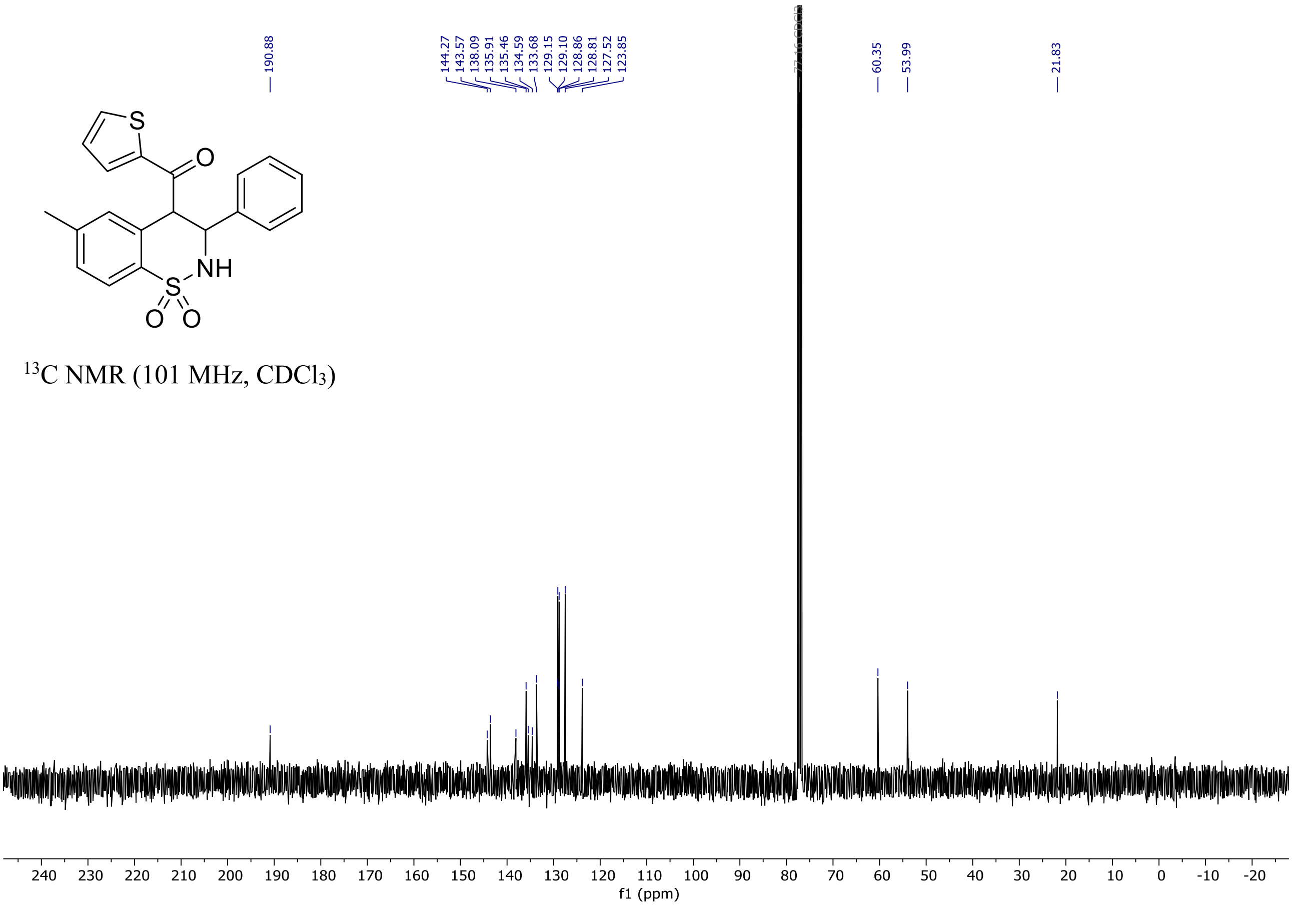

^{13}C NMR (101 MHz, $CDCl_3$)

(6-Methyl-1,1-dioxido-3-(*p*-tolyl)-3,4-dihydro-2*H*-benzo[*e*][1,2]thiazin-4-yl)(phenyl)methanone (2w)

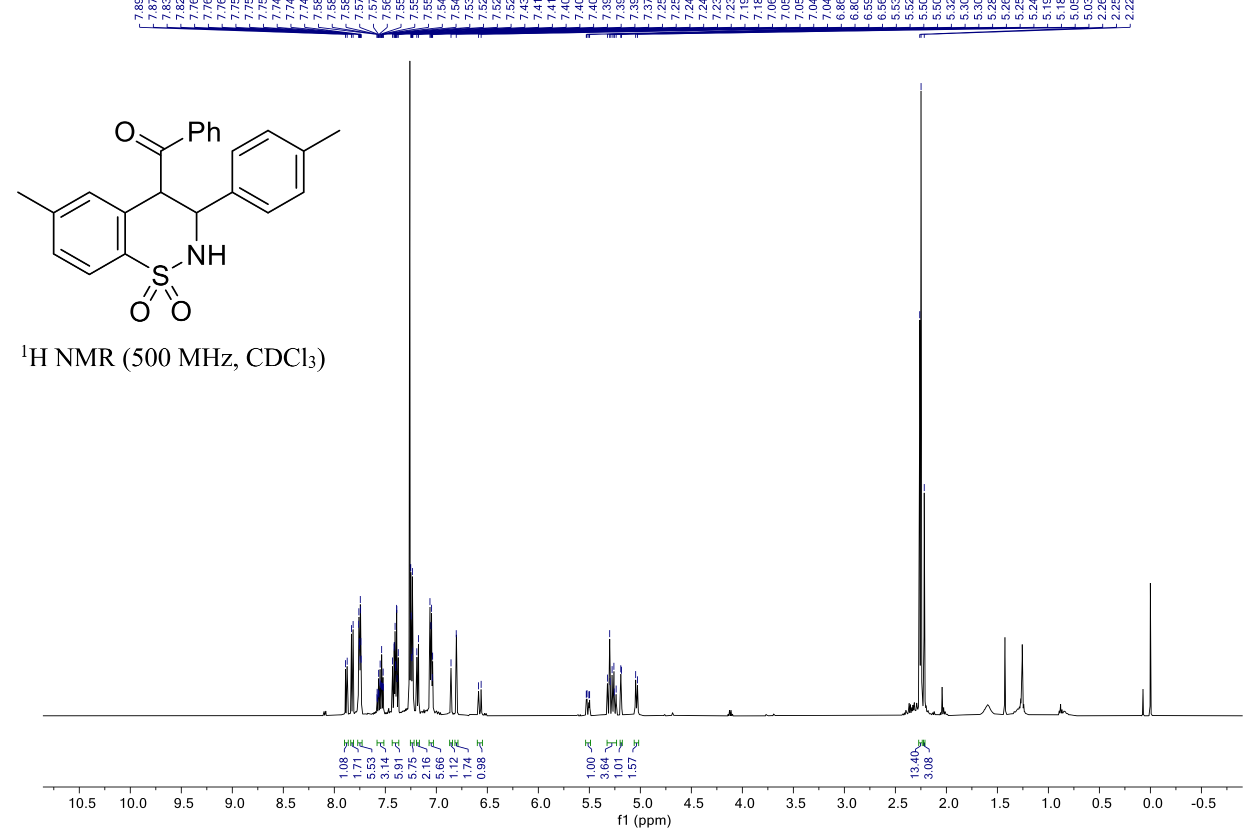

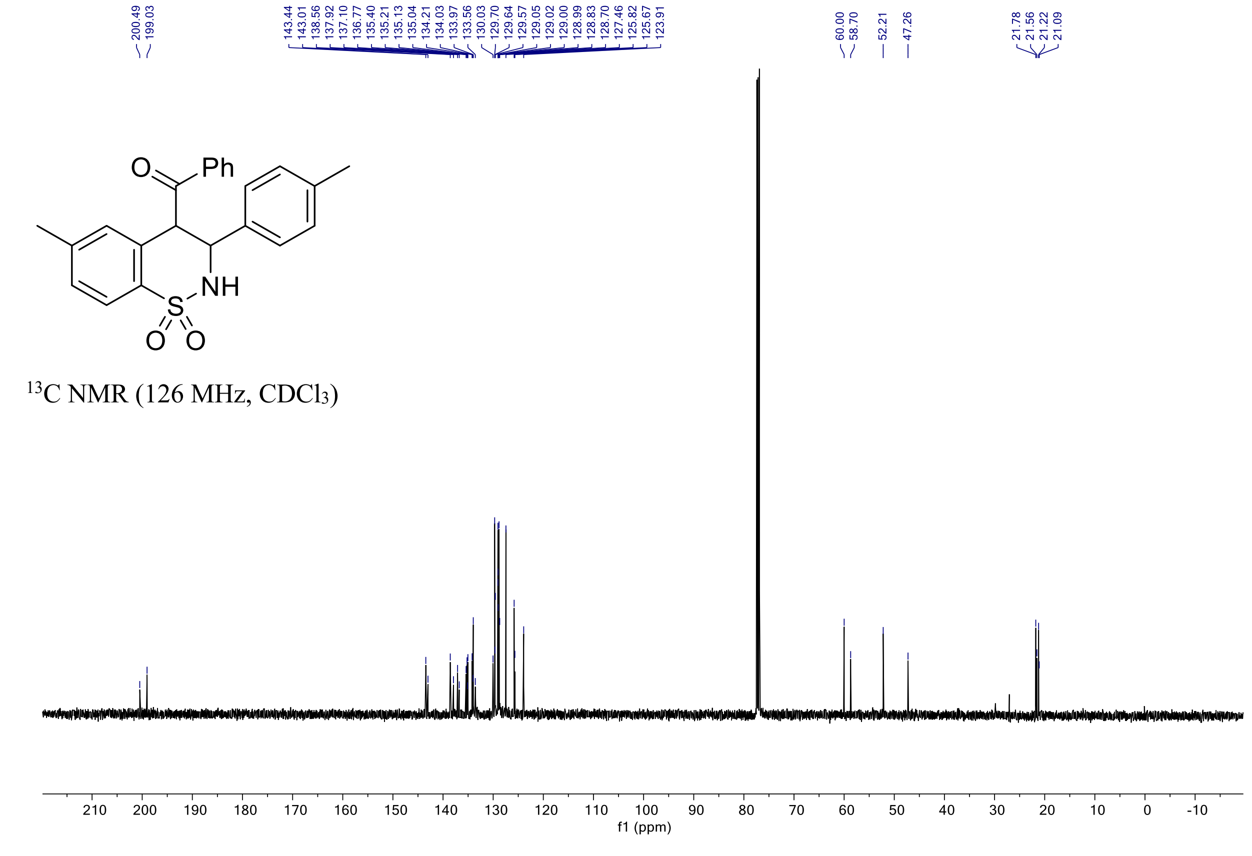

(6-Methyl-1,1-dioxido-3-(*o*-tolyl)-3,4-dihydro-2*H*-benzo[*e*][1,2]thiazin-4-yl)(phenyl)methanone (2x)

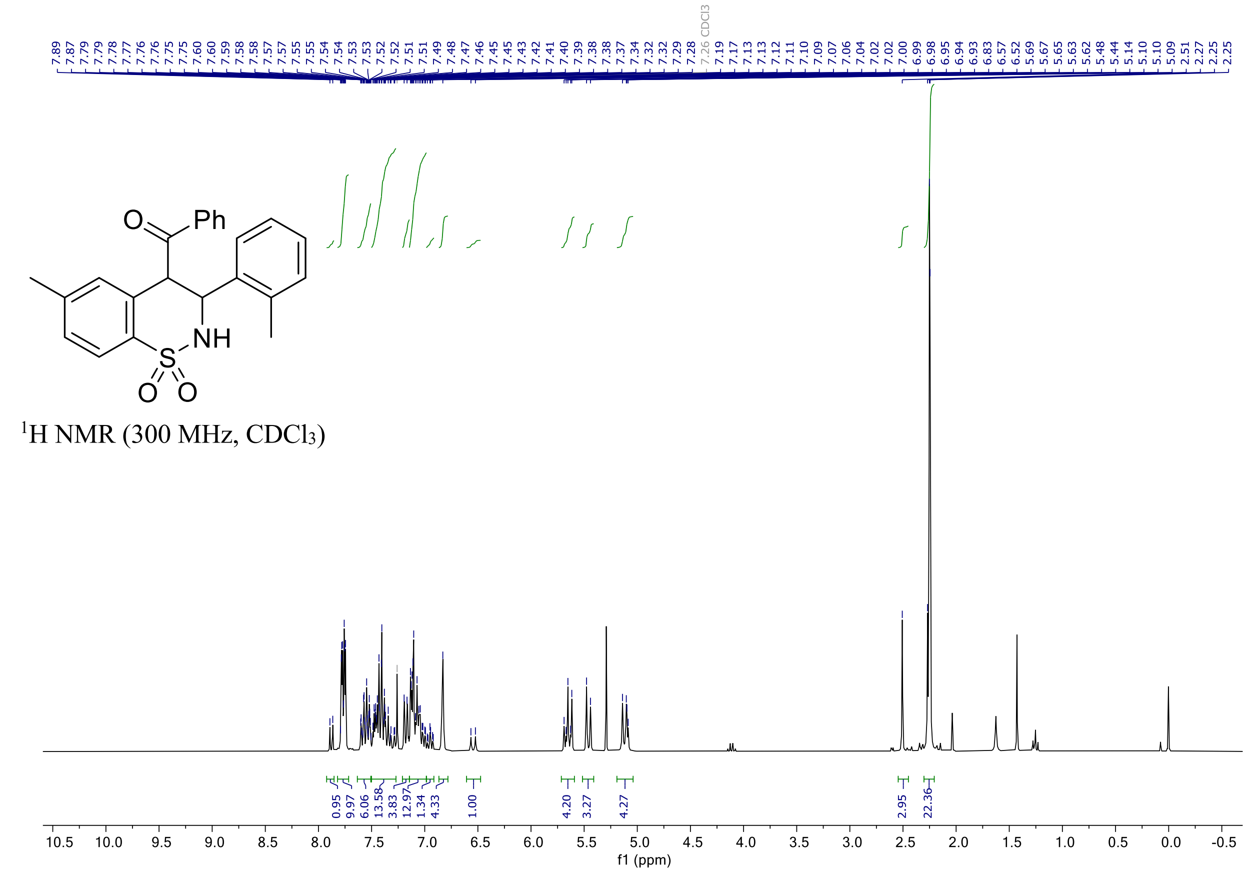

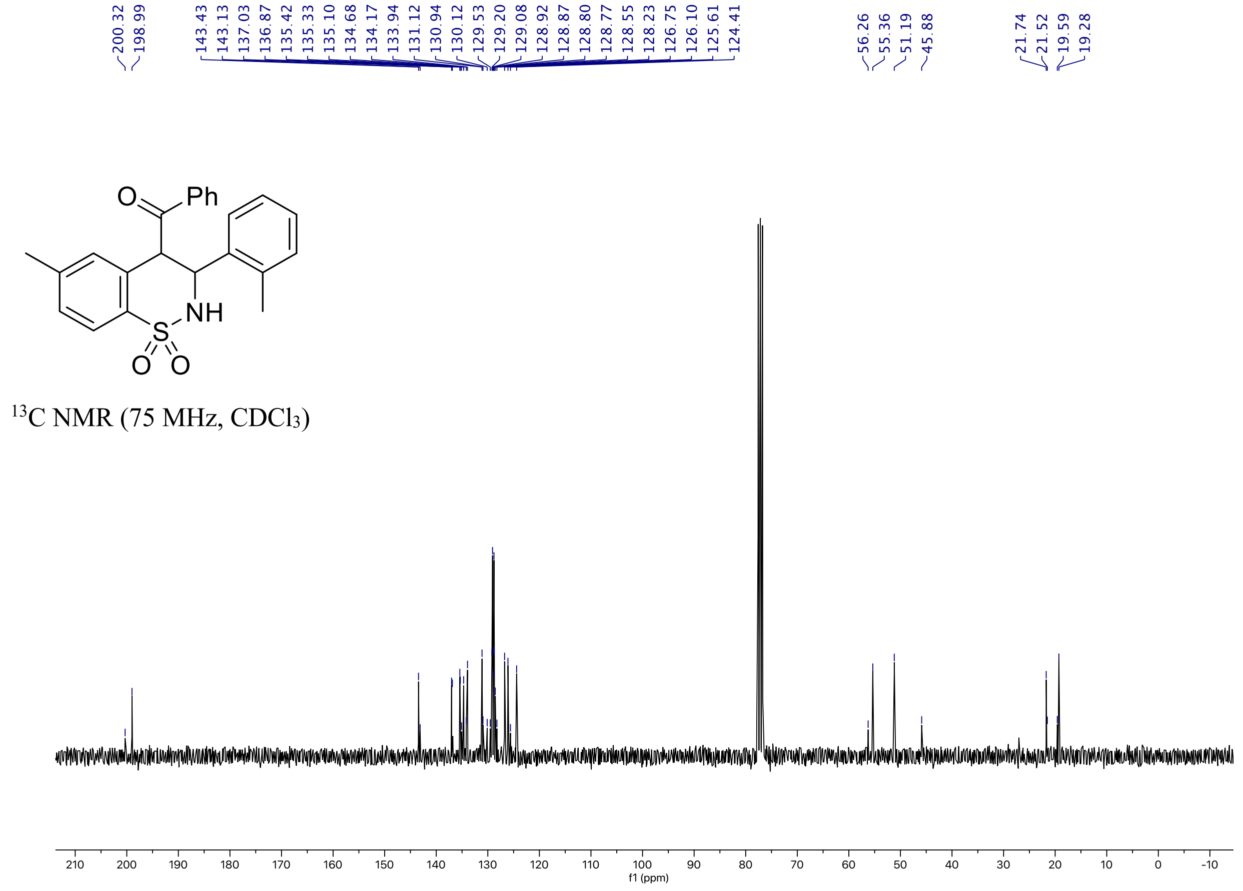

(3-(3-Methoxyphenyl)-6-methyl-1,1-dioxido-3,4-dihydro-2*H*-benzo[*e*][1,2]thiazin-4-yl)(phenyl)methanone (2y)

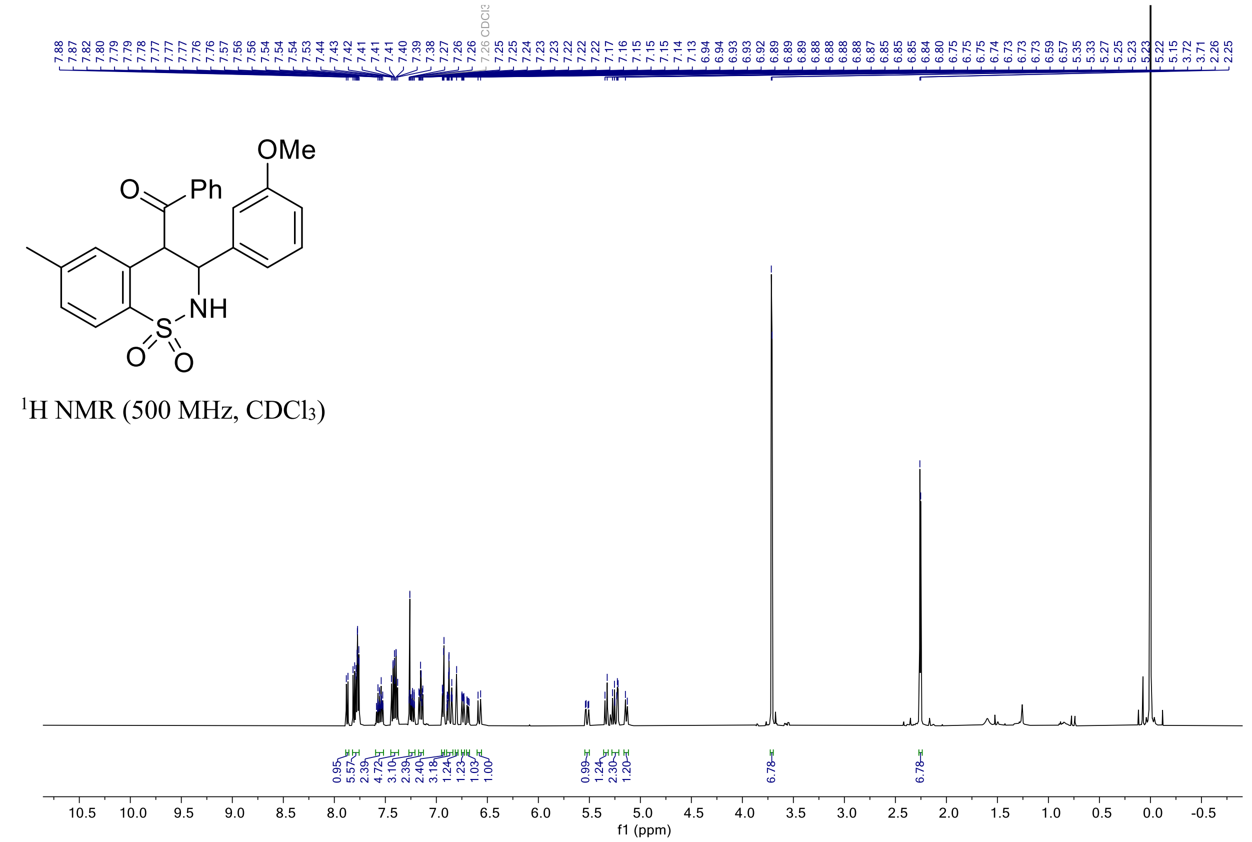

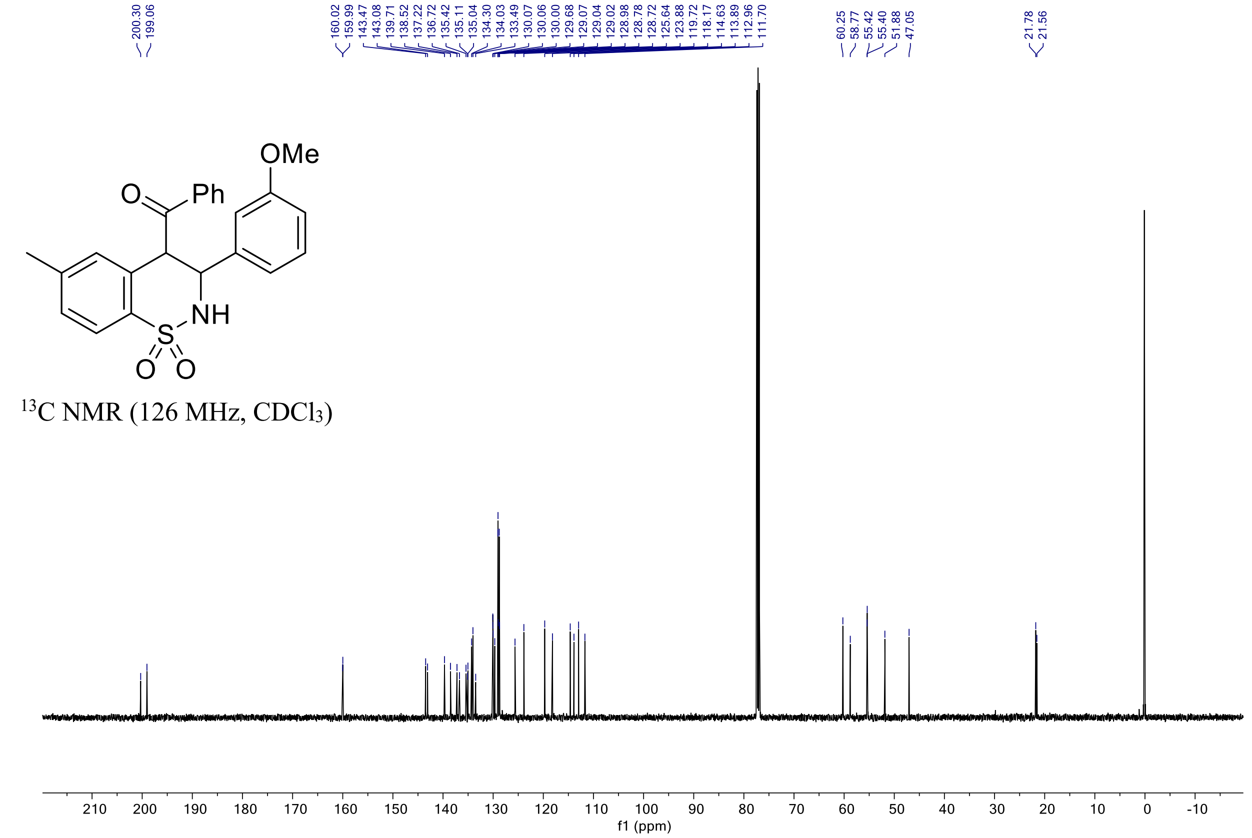

(3-(4-Fluorophenyl)-6-methyl-1,1-dioxido-3,4-dihydro-2*H*-benzo[*e*][1,2]thiazin-4-yl)(phenyl)methanone (2z)

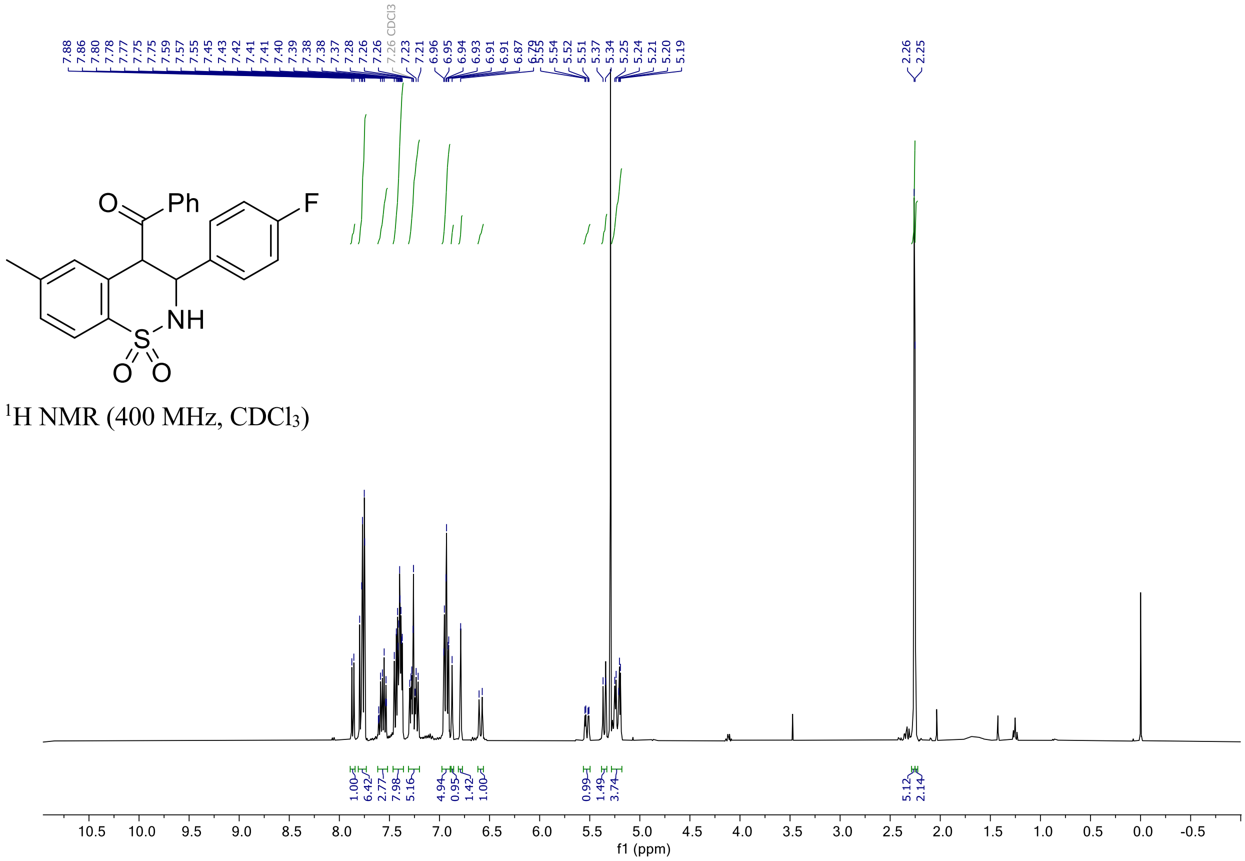

^{1}H NMR (400 MHz, $CDCl_3$)

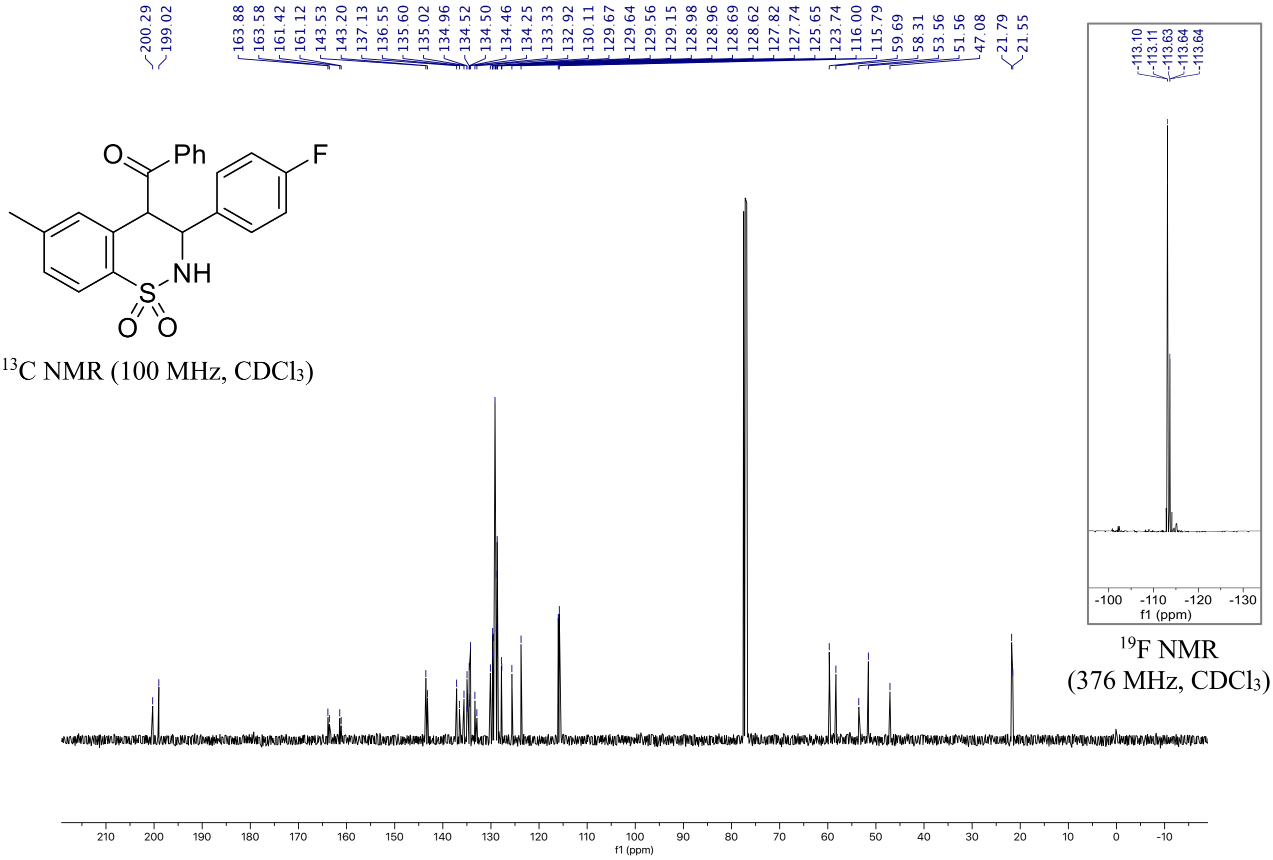

^{13}C NMR (100 MHz, $CDCl_3$)

^{19}F NMR
(376 MHz, $CDCl_3$)

(3-(4-Chlorophenyl)-6-methyl-1,1-dioxido-3,4-dihydro-2*H*-benzo[*e*][1,2]thiazin-4-yl)(phenyl)methanone (2za)

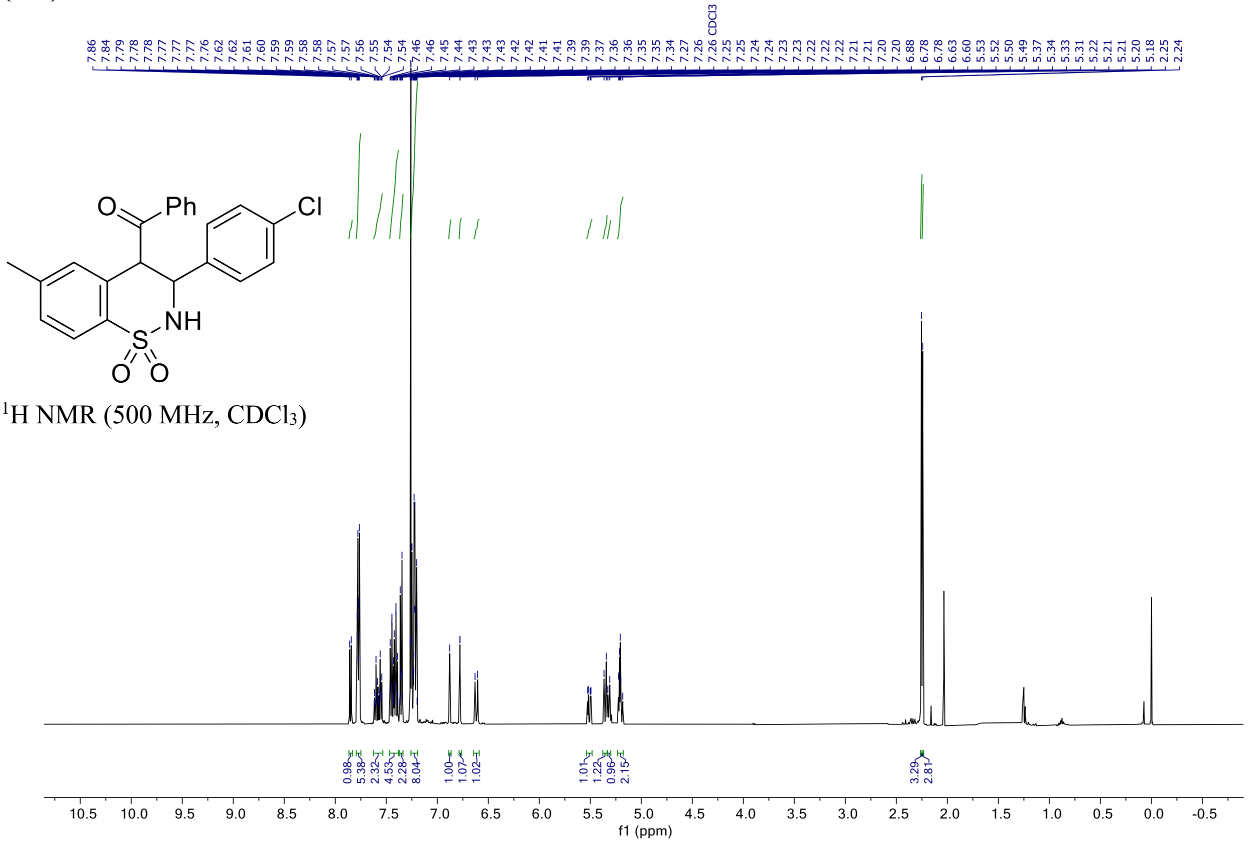

^{1}H NMR (500 MHz, $CDCl_3$)

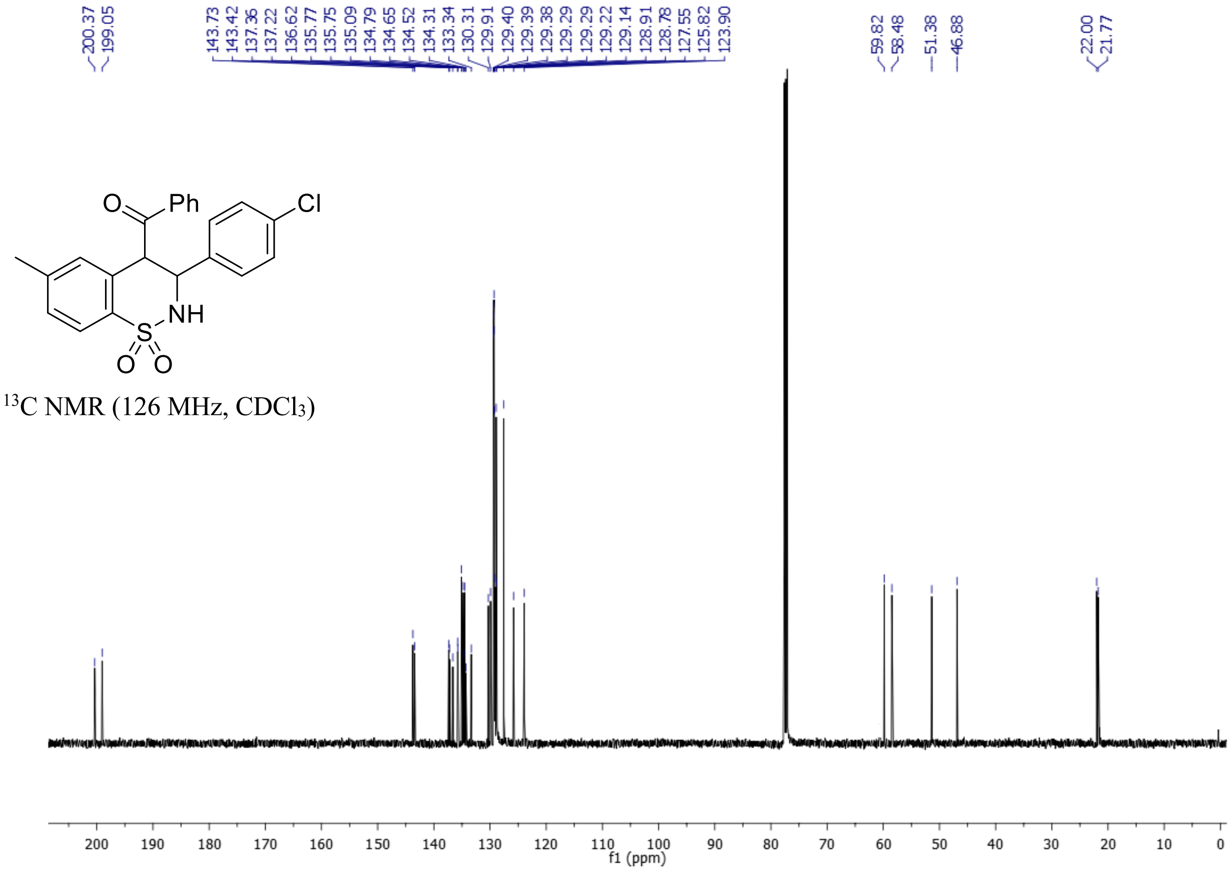

^{13}C NMR (126 MHz, $CDCl_3$)

(3*S*,4*R*)/(3*R*,4*S*)-(3-(2-Chlorophenyl)-6-methyl-1,1-dioxido-3,4-dihydro-2*H*-benzo[*e*][1,2]thiazin-4-yl)(phenyl)methanone (*cis*-2zb)

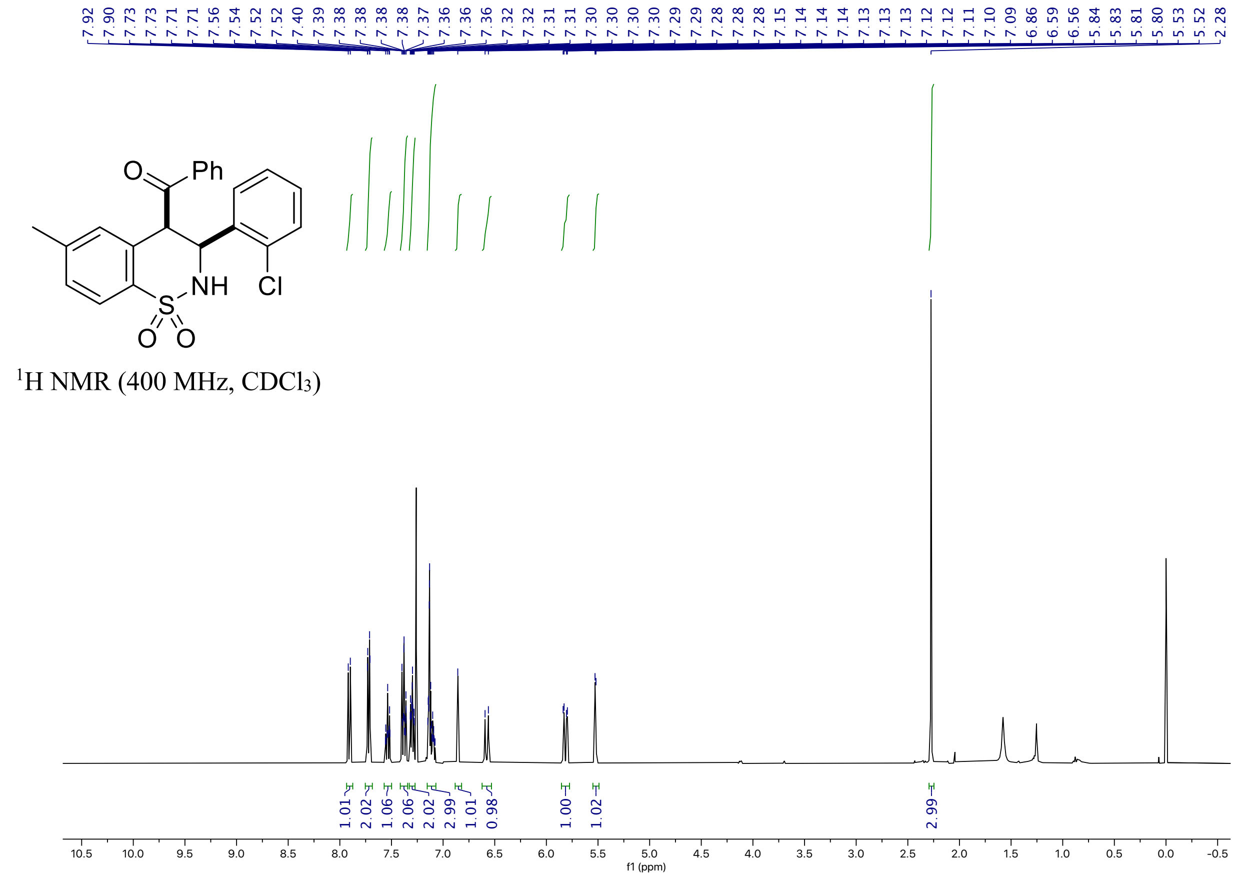

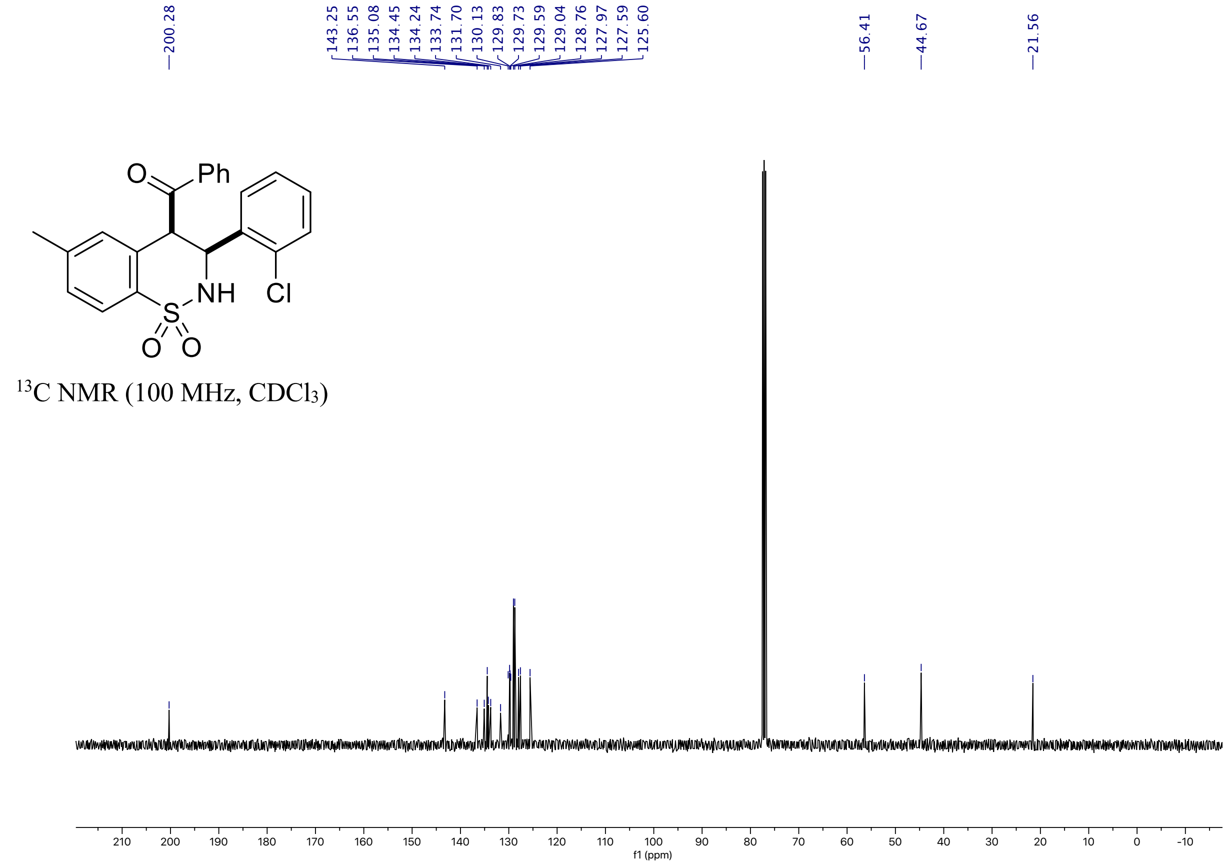

(3*R*,4*R*)/(3*S*,4*S*)-(3-(2-Chlorophenyl)-6-methyl-1,1-dioxido-3,4-dihydro-2*H*-benzo[*e*][1,2]thiazin-4-yl)(phenyl)methanone (*trans*-2zb)

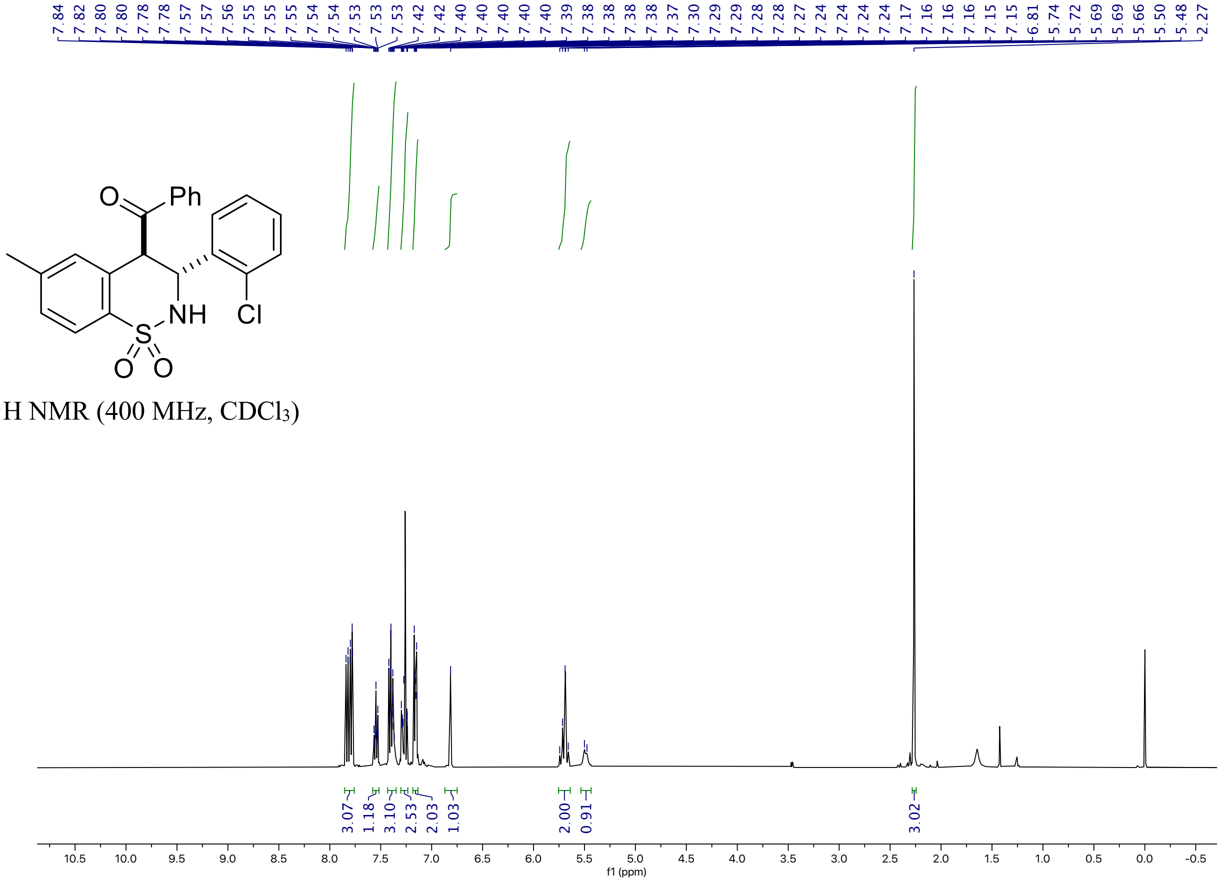

^{1}H NMR (400 MHz, CDCl$_3$)

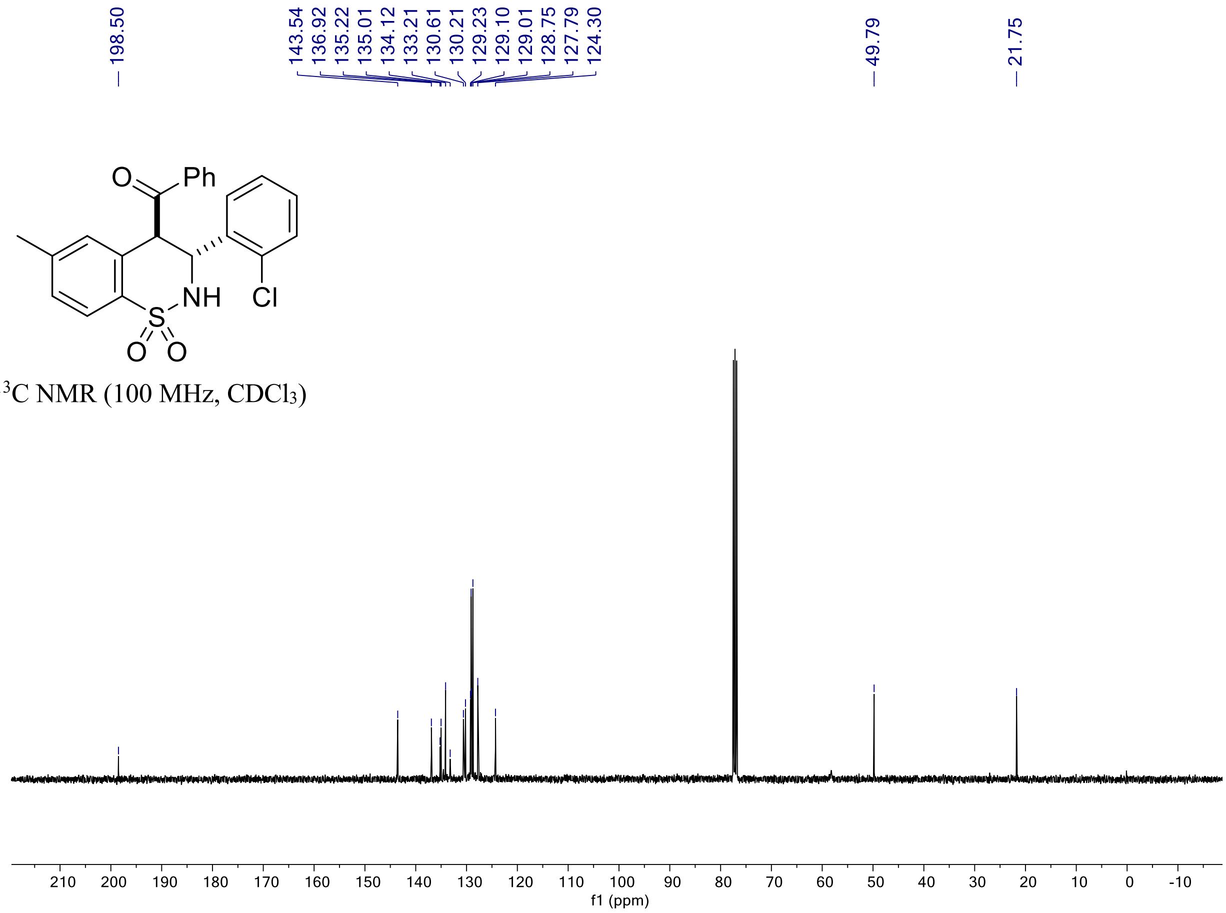

^{13}C NMR (100 MHz, CDCl$_3$)

(6-Methyl-1,1-dioxido-3,4-dihydro-2*H*-benzo[*e*][1,2]thiazin-4-yl)(phenyl)methanone (2zc)

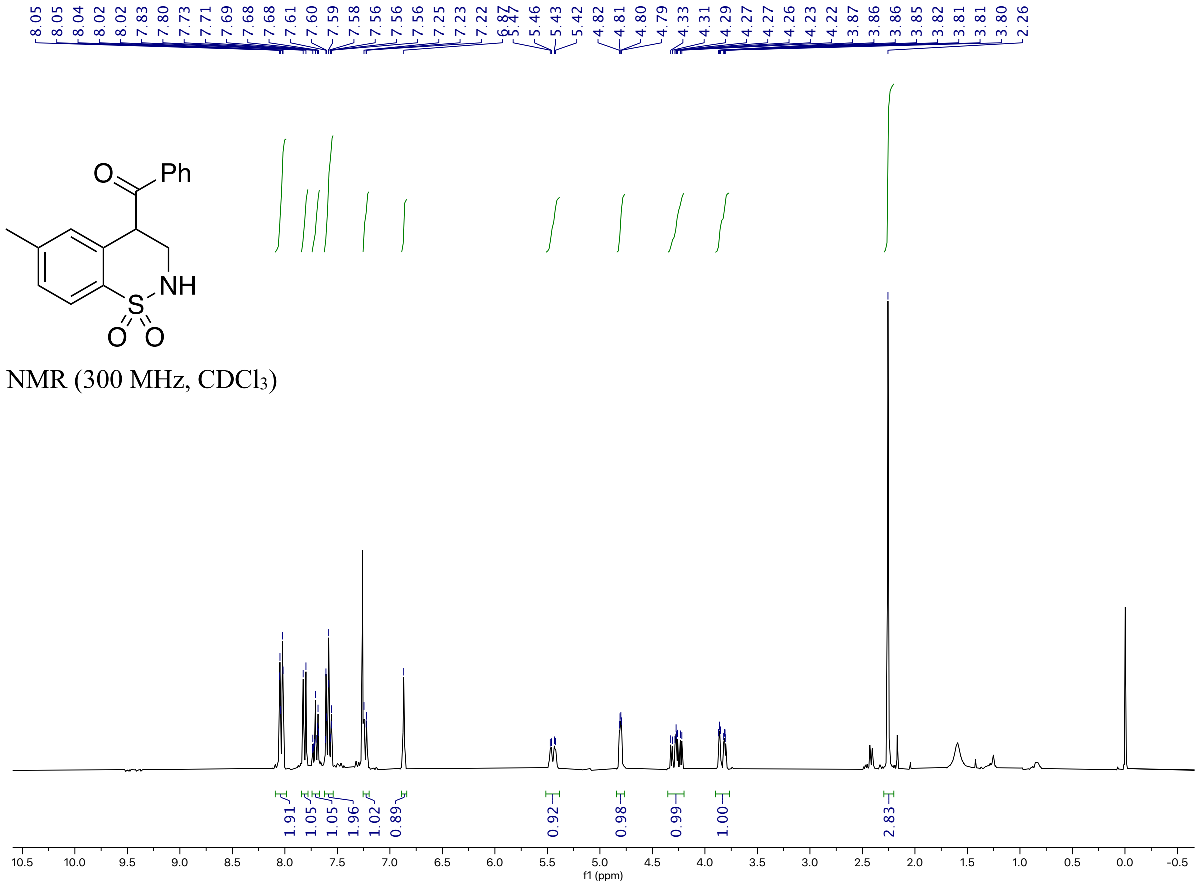

^{1}H NMR (300 MHz, $CDCl_3$)

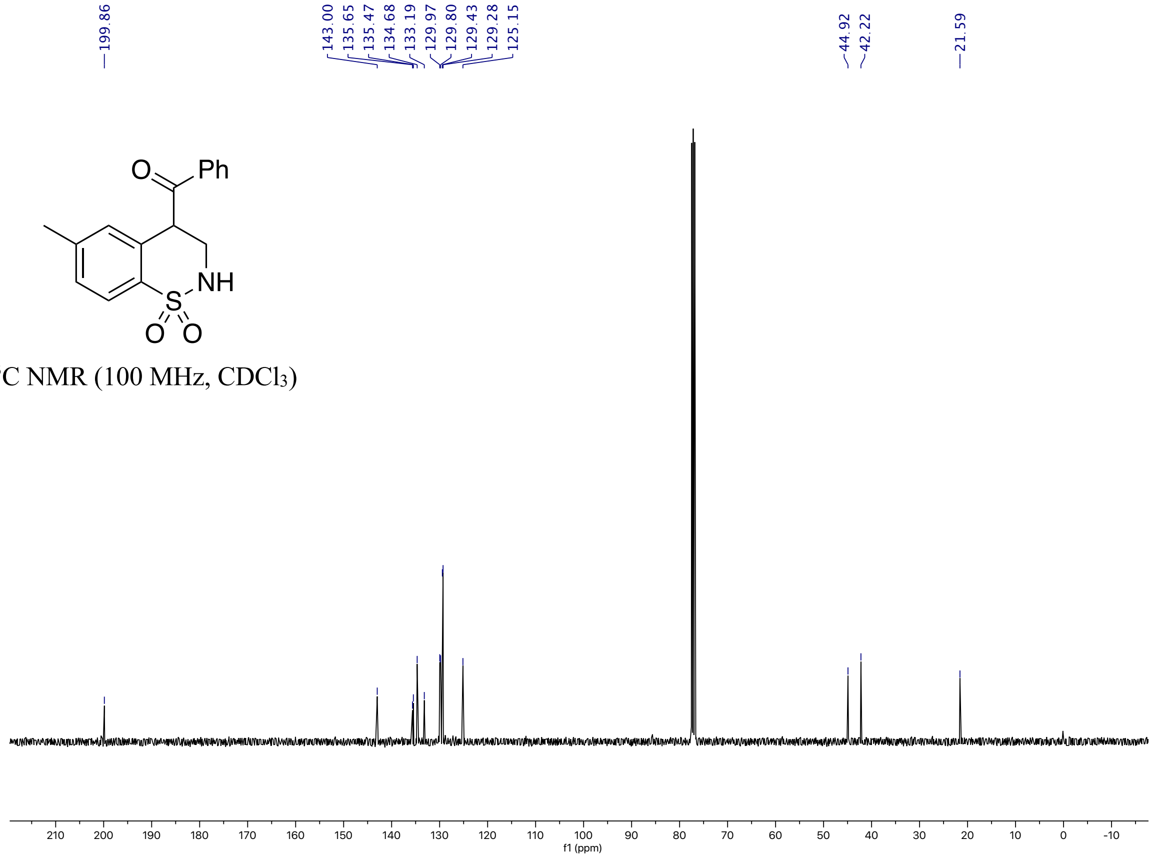

^{13}C NMR (100 MHz, $CDCl_3$)

(3*R*,4*R*)/(3*S*,4*S*)-(3,6-Dimethyl-1,1-dioxido-3,4-dihydro-2*H*-benzo[*e*][1,2]thiazin-4-yl)(phenyl)methanone (*cis*-2zd)

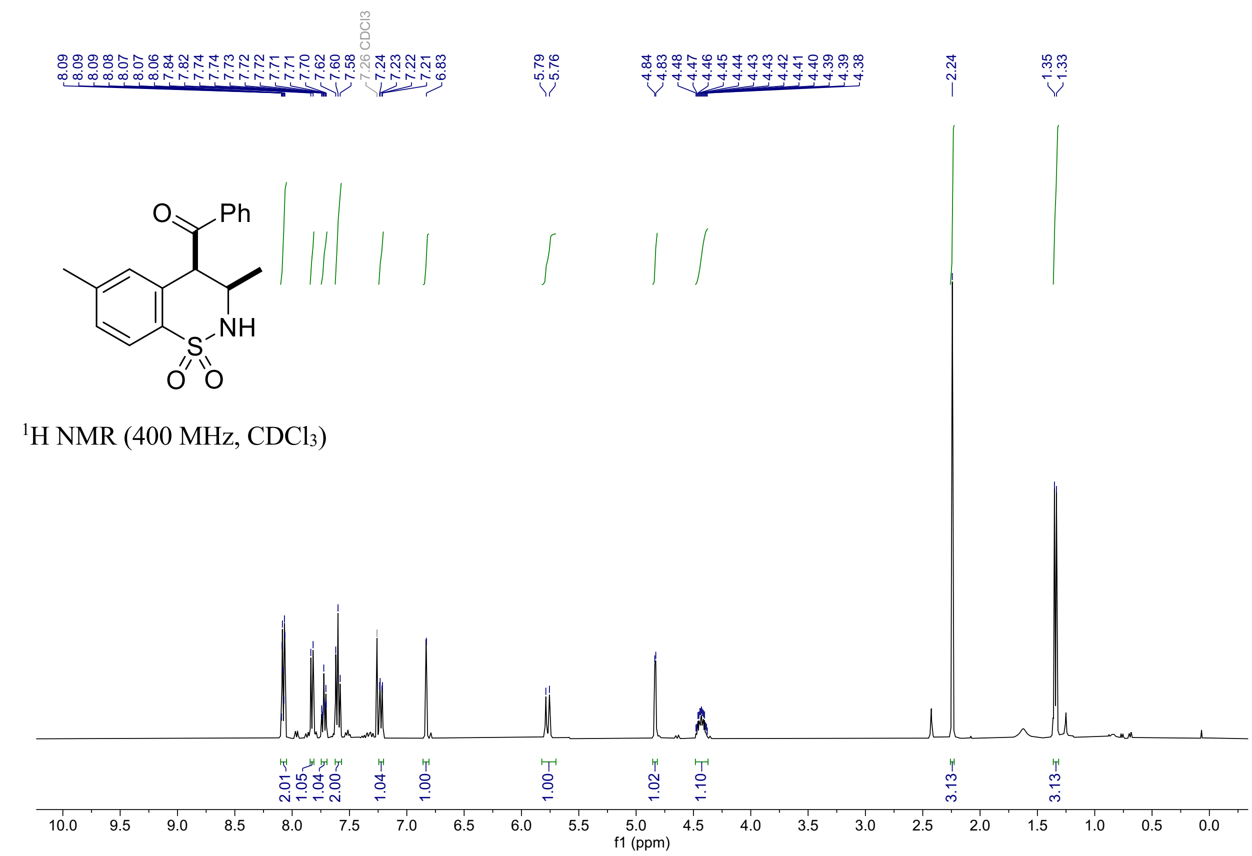

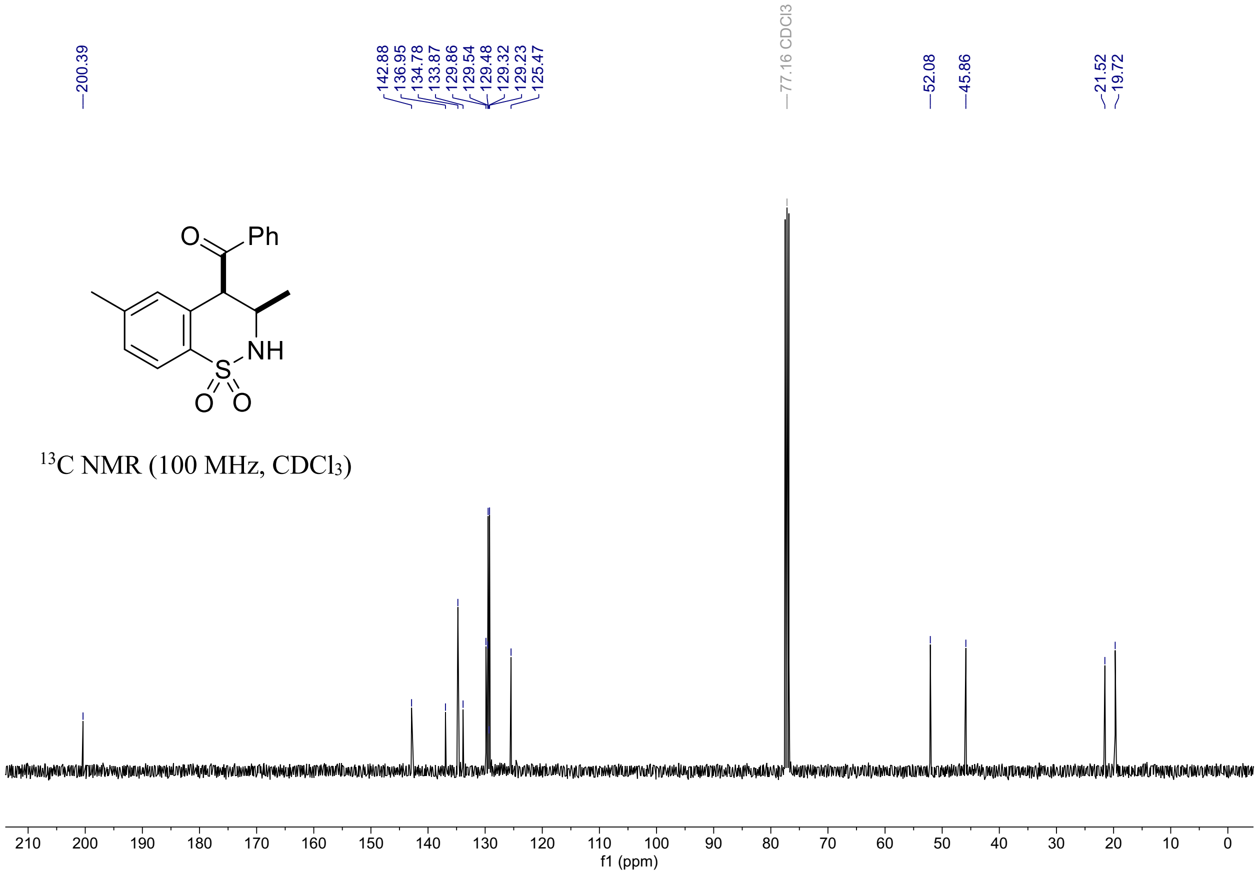

(3*S*,4*R*)/(3*R*,4*S*)-(3,6-Dimethyl-1,1-dioxido-3,4-dihydro-2*H*-benzo[*e*][1,2]thiazin-4-yl)(phenyl)methanone (*trans*-2zd)

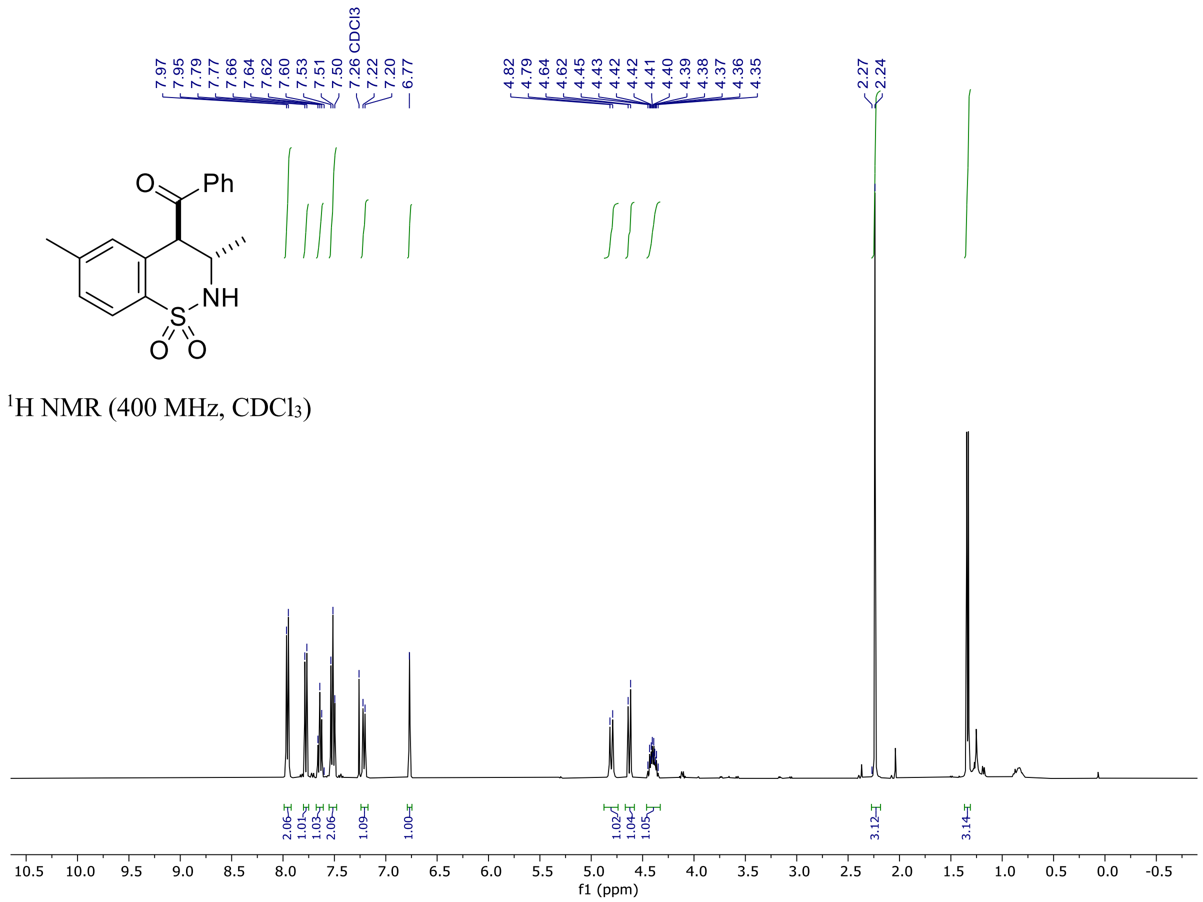

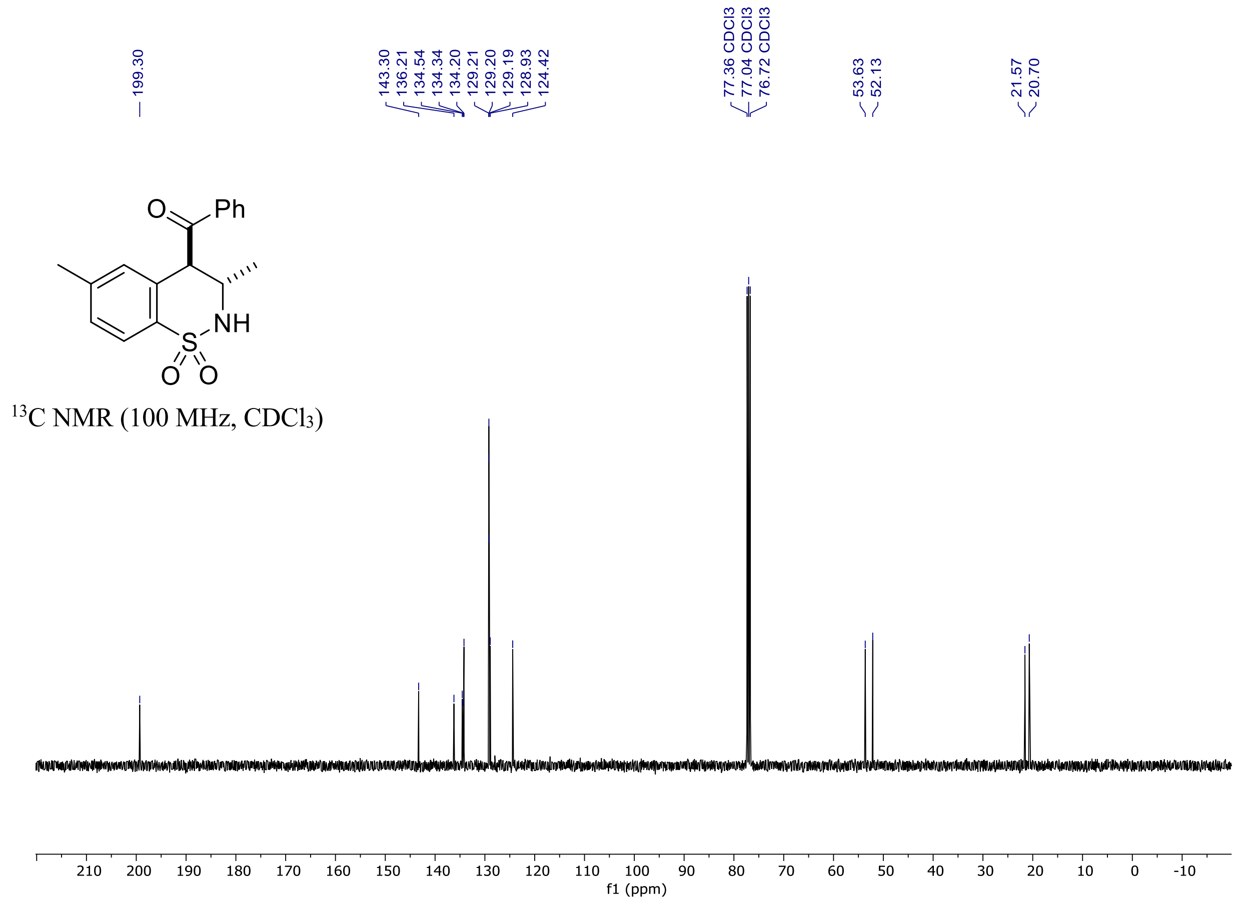

4-Methyl-2-(2-phenylquinolin-3-yl)benzenesulfonamide (2ze')

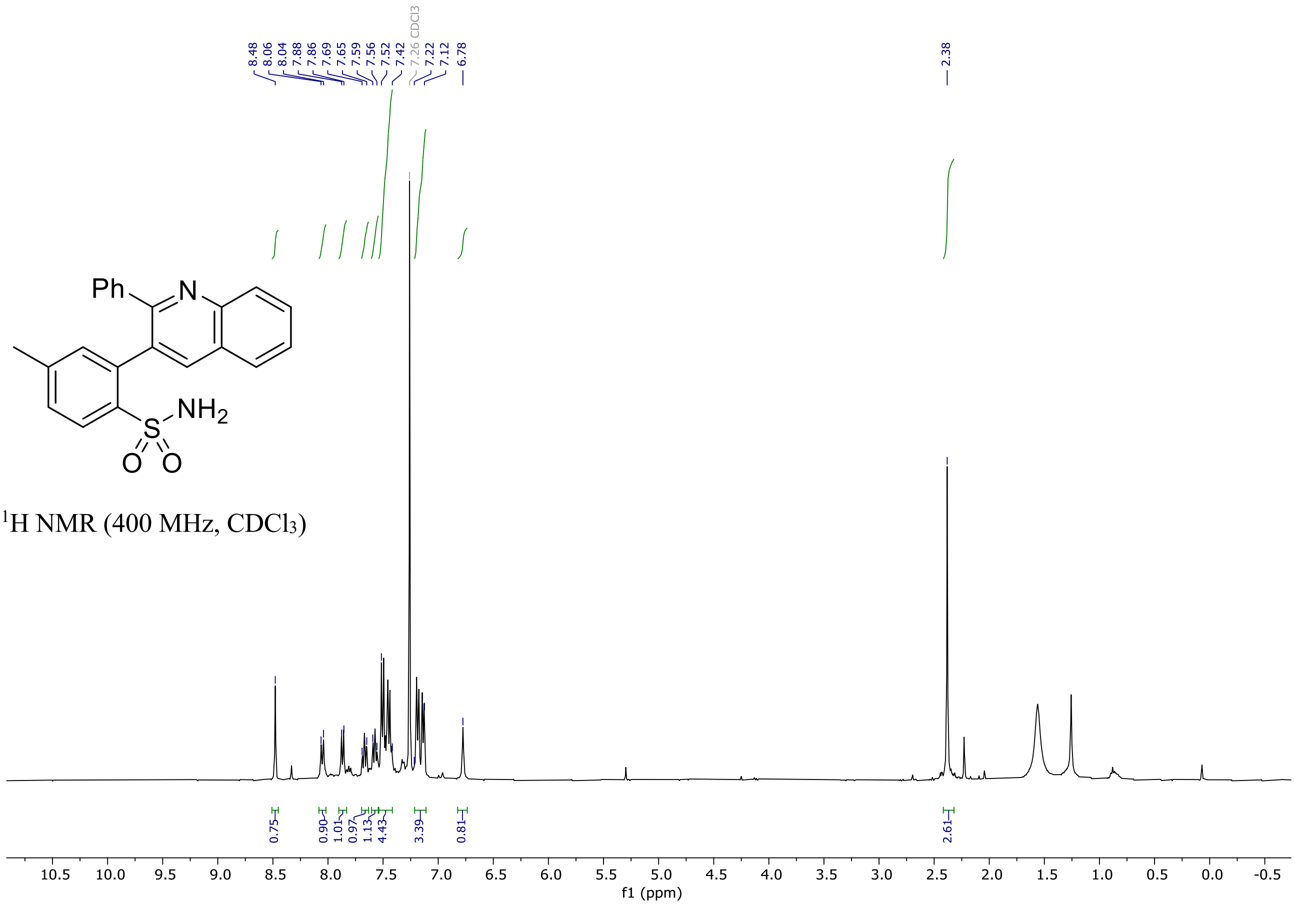

^{1}H NMR (400 MHz, $CDCl_3$)

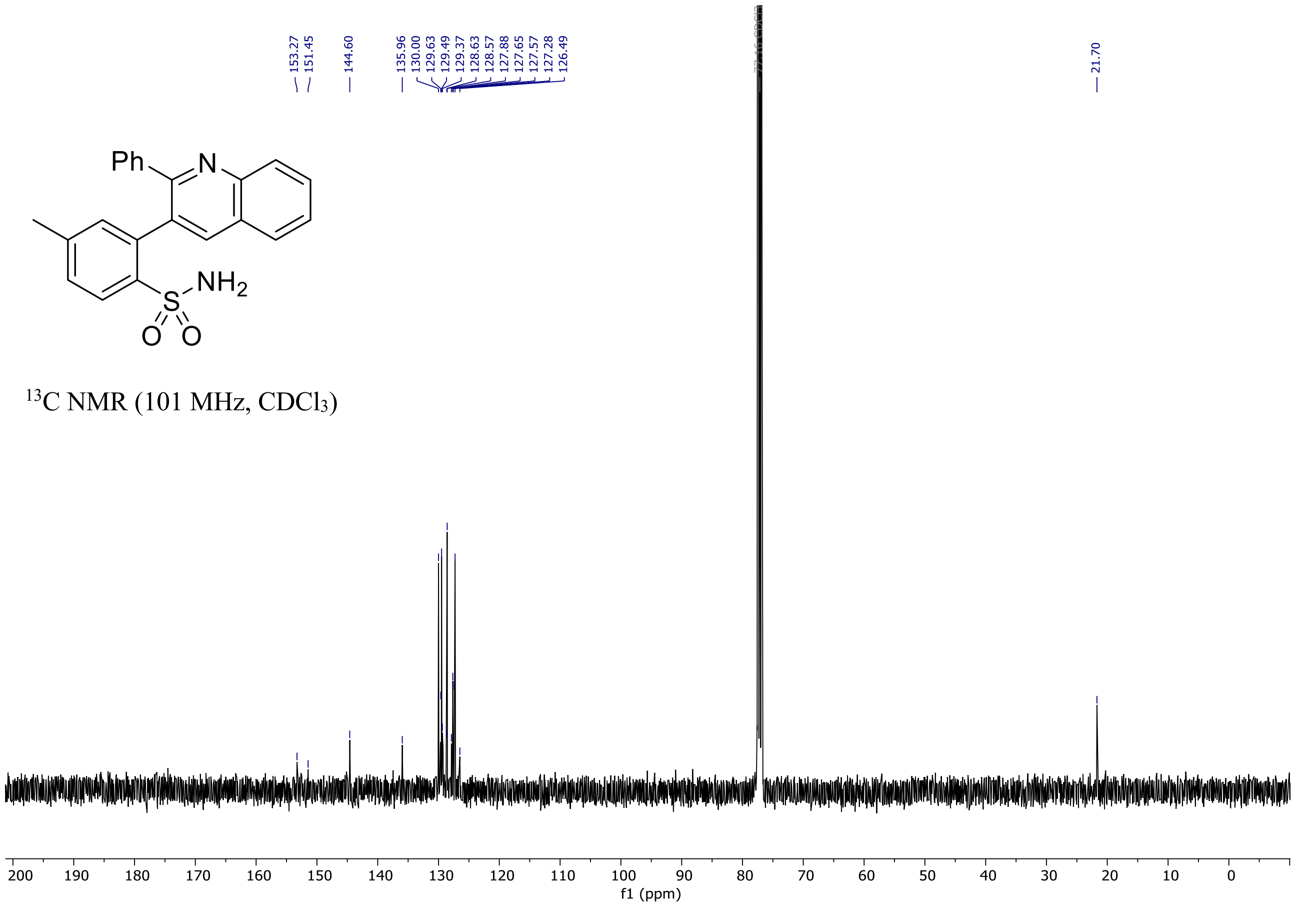

^{13}C NMR (101 MHz, $CDCl_3$)

(3*R*,4*R*)/(3*S*,4*S*)-2-Benzyl-6-methyl-1,1-dioxido-3-phenyl-3,4-dihydro-2*H*-benzo[*e*][1,2]thiazin-4-yl)(phenyl)methanone (*trans*-3a)

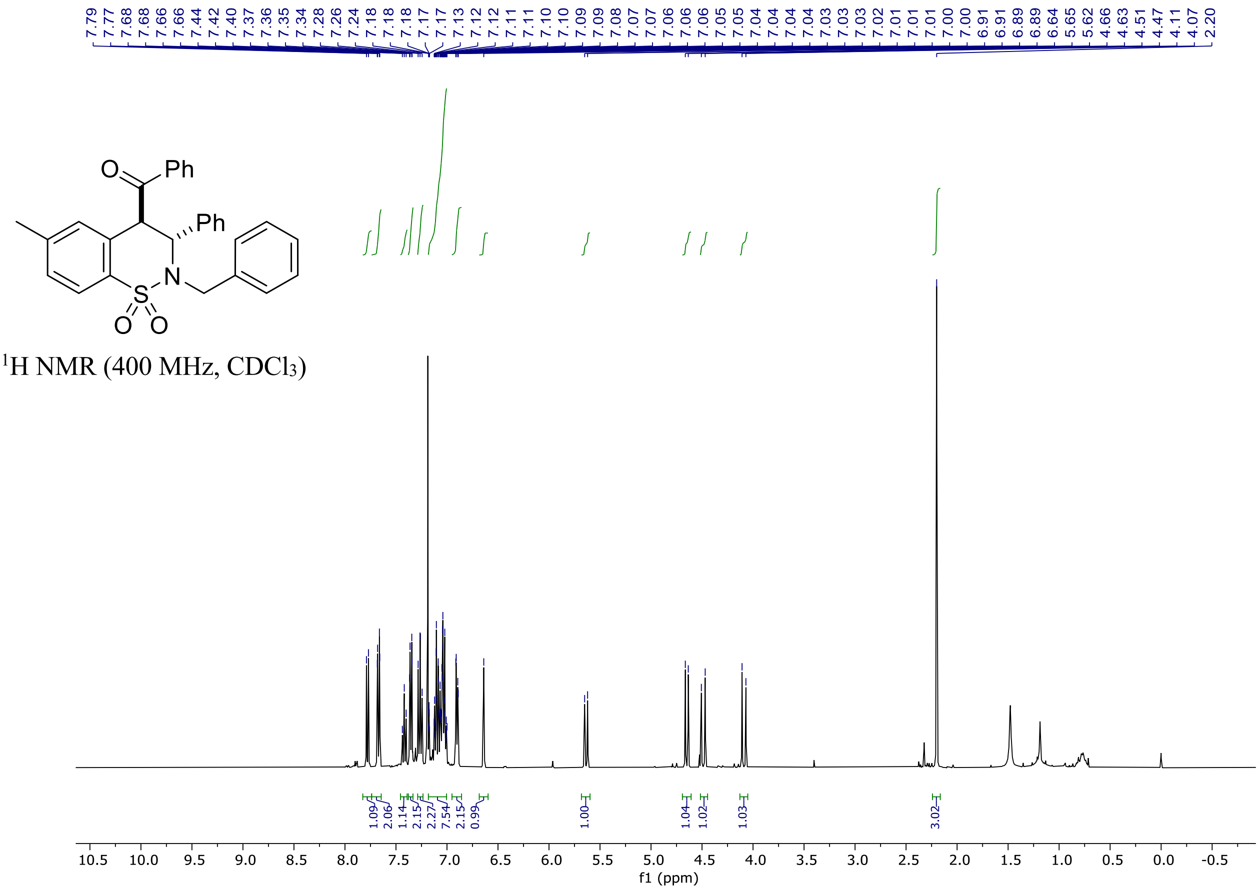

^{1}H NMR (400 MHz, $CDCl_3$)

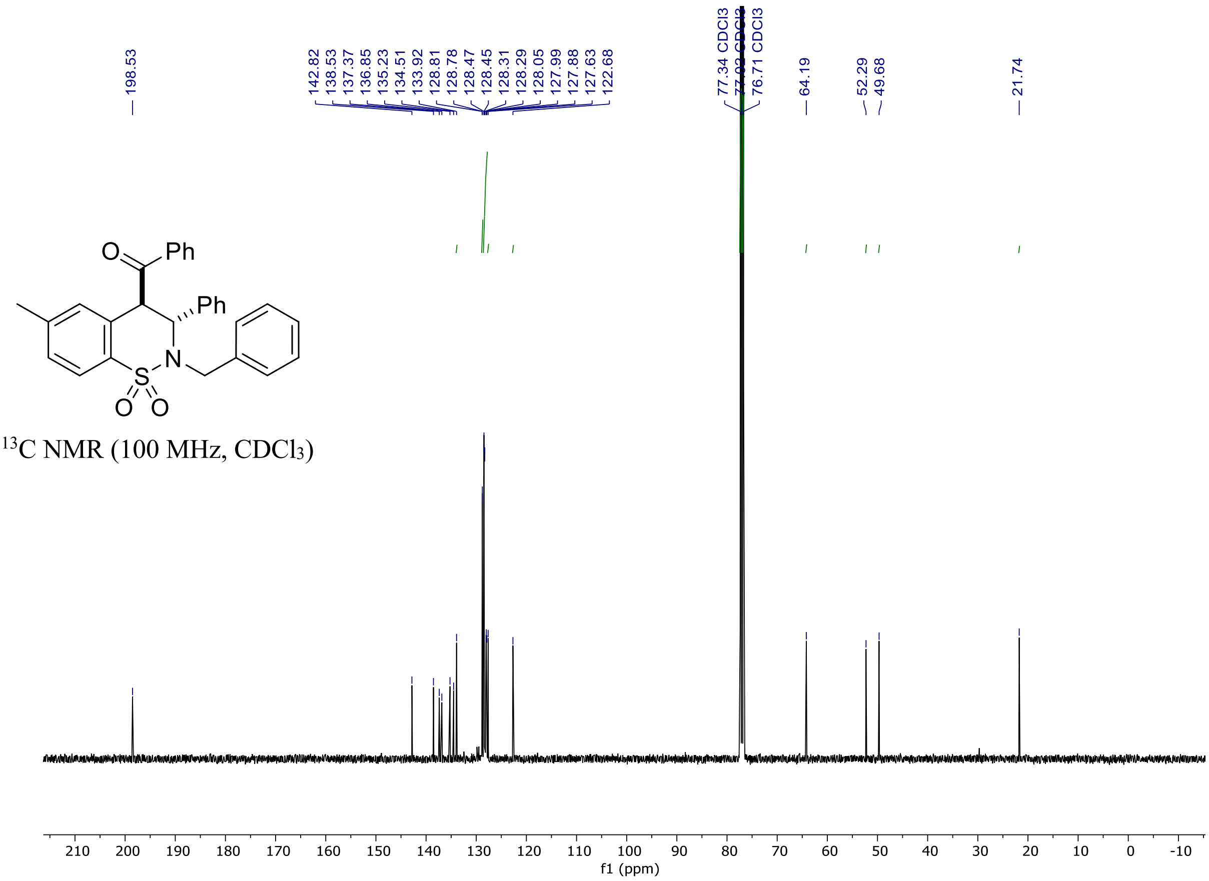

^{13}C NMR (100 MHz, $CDCl_3$)

(2,6-Dimethyl-1,1-dioxido-3-phenyl-3,4-dihydro-2*H*-benzo[*e*][1,2]thiazin-4-yl)(phenyl)methanone (4a)

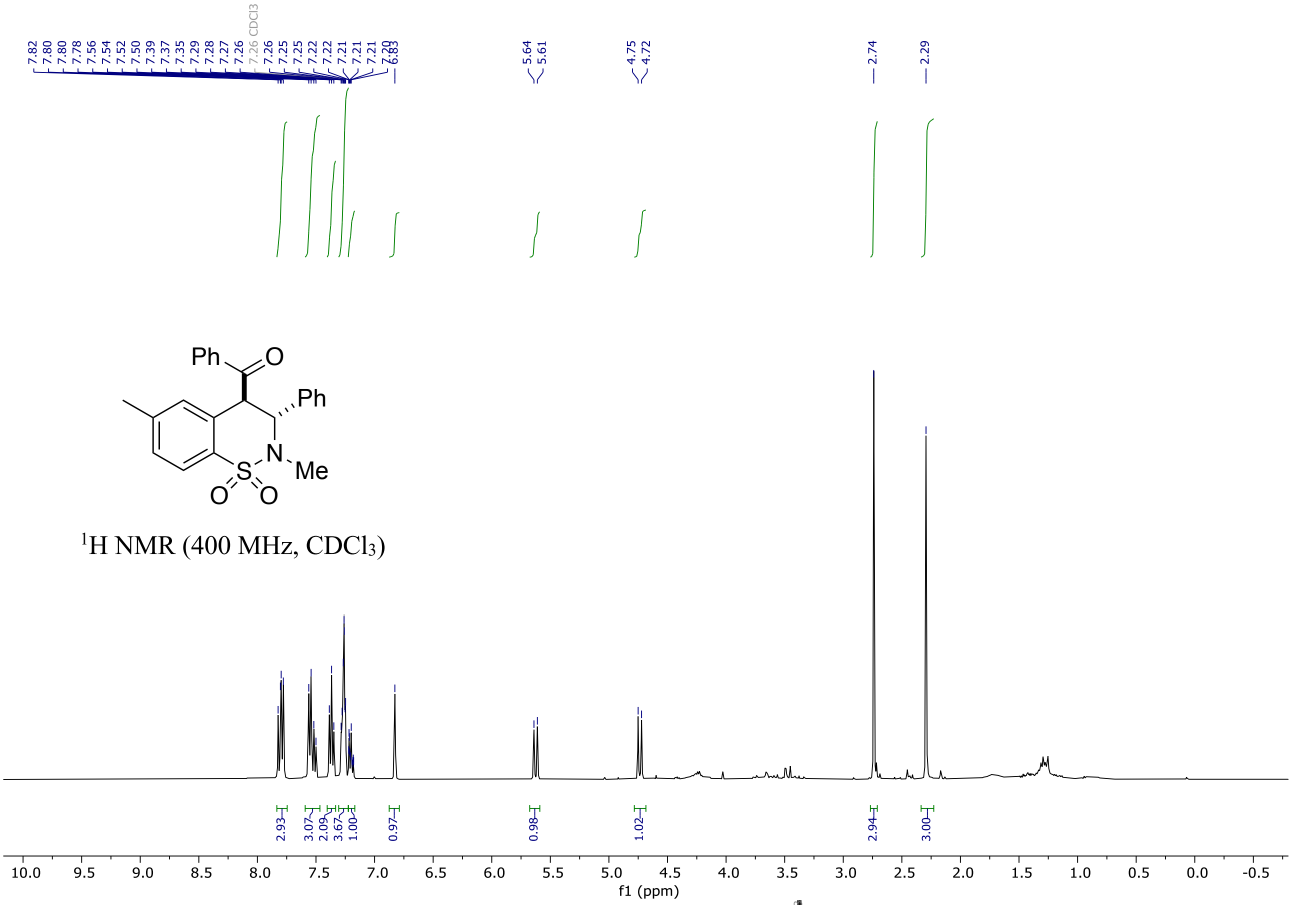

^{1}H NMR (400 MHz, $CDCl_3$)

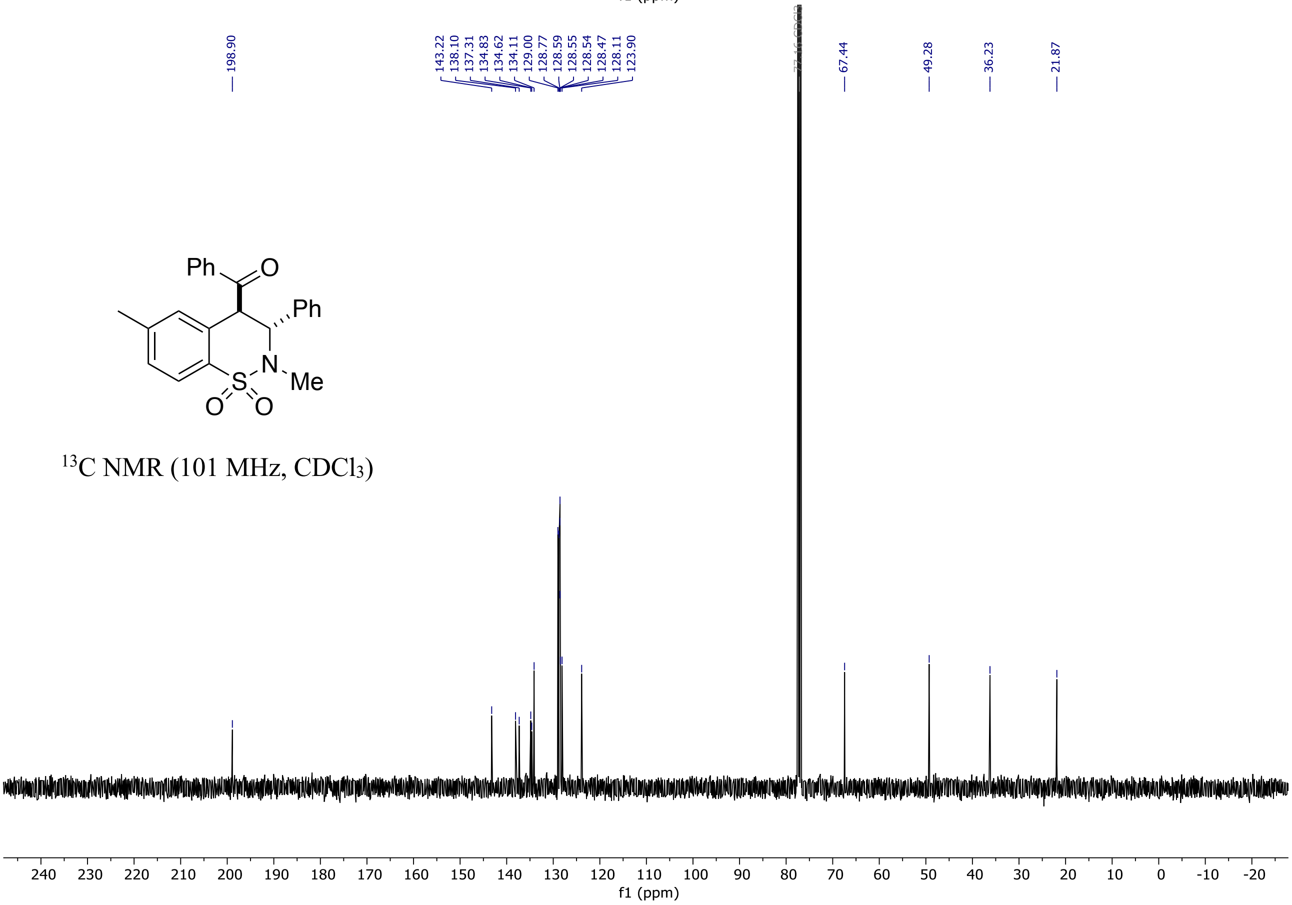

^{13}C NMR (101 MHz, $CDCl_3$)

(*E*/*Z*)-4-Methyl-2-(3-oxo-1,3-diphenylprop-1-en-2-yl)-*N*,*N*-diphenylbenzenesulfonamide (5a)

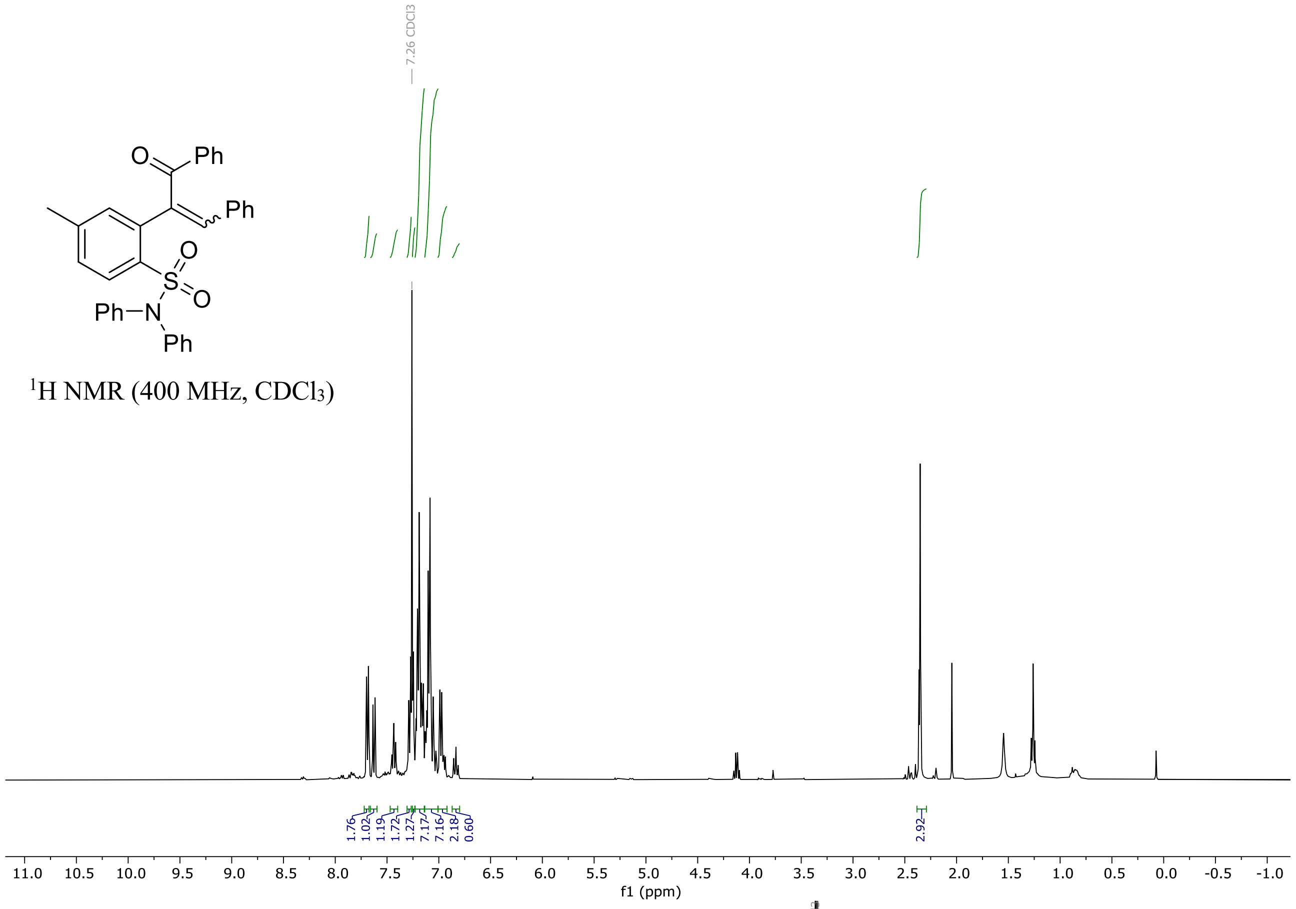

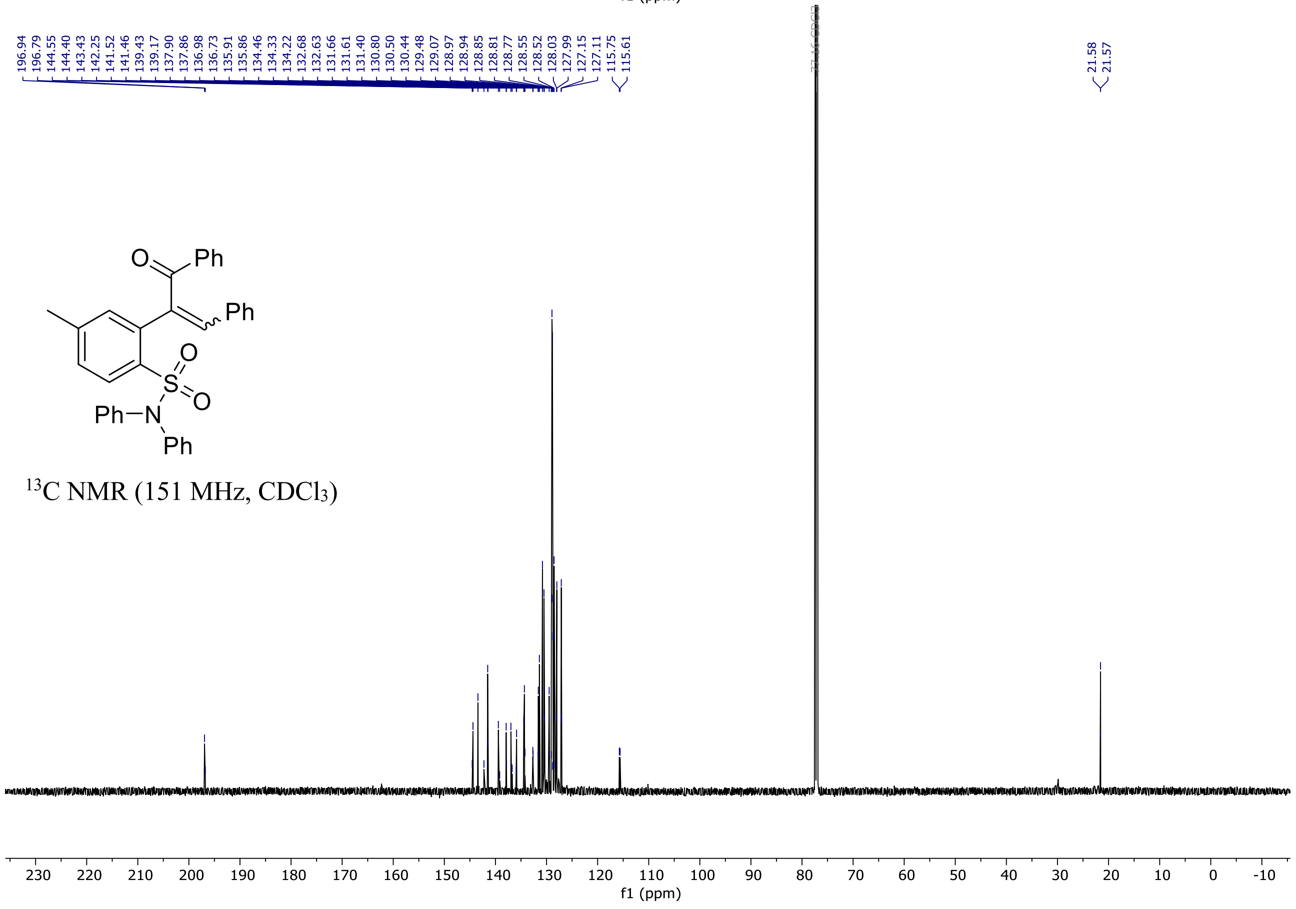

4-Methyl-*N*-(3-oxo-1,3-diphenyl-2-((2,2,6,6-tetramethylpiperidin-1-yl)oxy)propyl)benzenesulfonamide (6a)

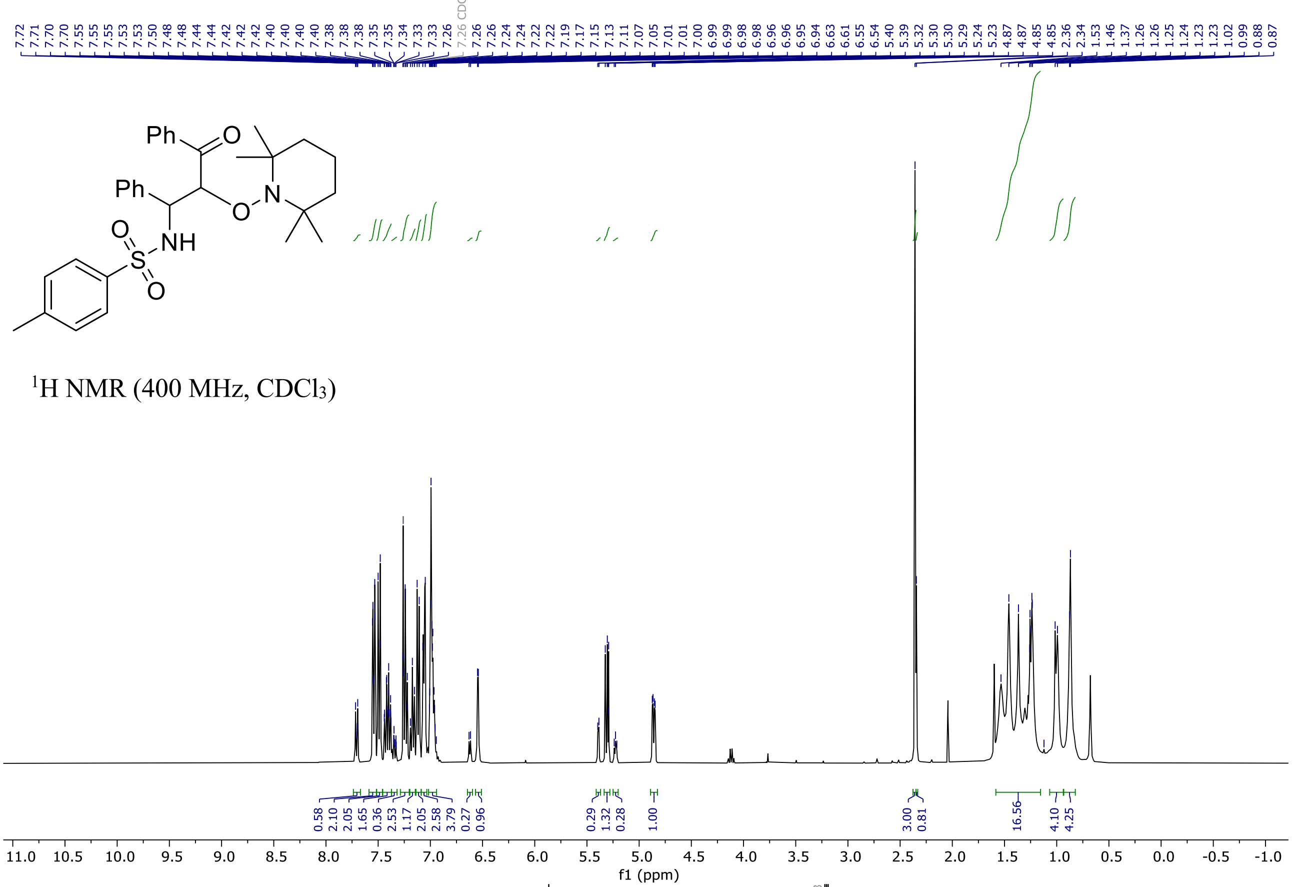

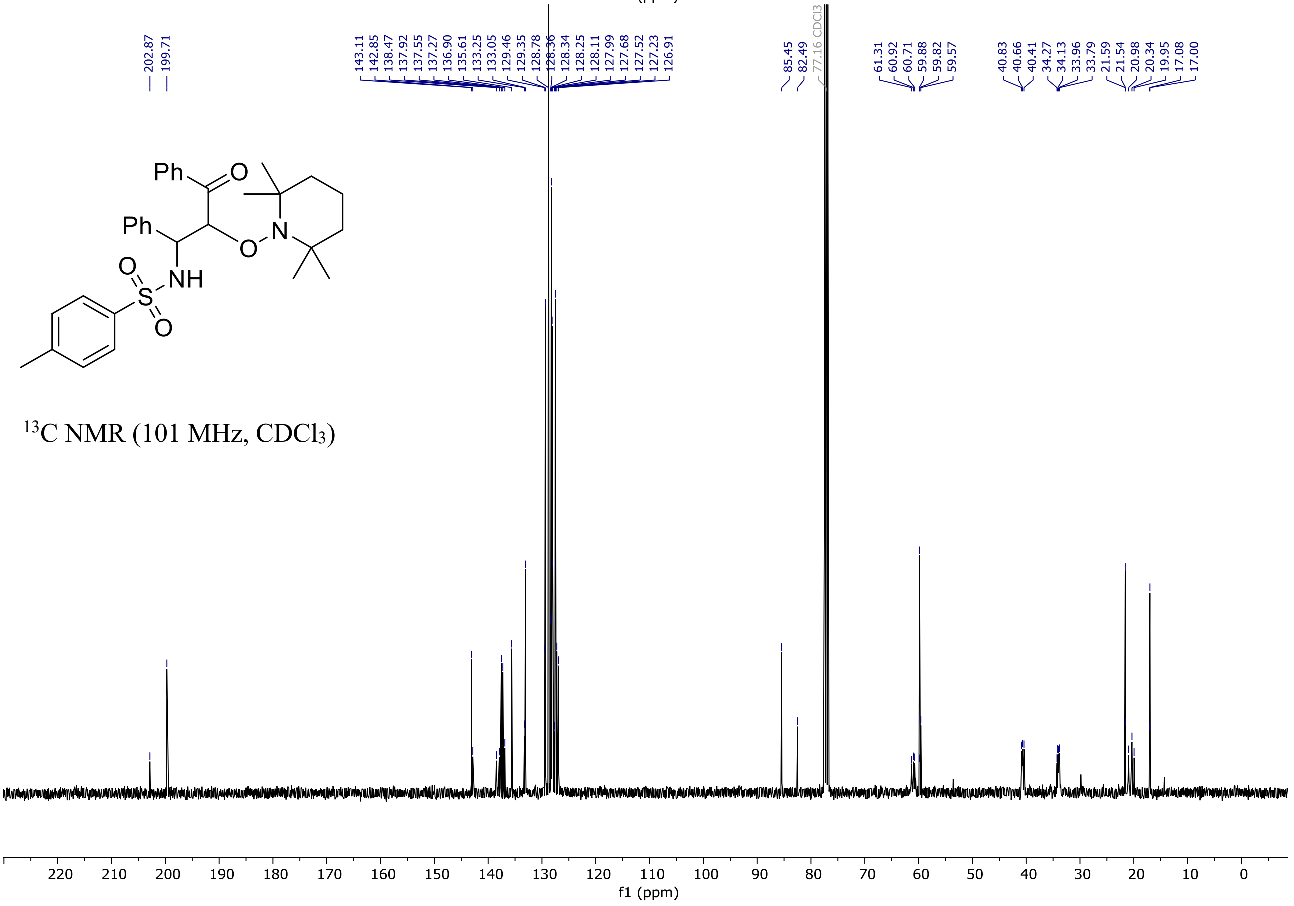

Supplement: Supplementary file 1 — Supporting Information [file ANIE-65-e16731-s002.pdf]
